
JOURNAL INFORMATION
==============================
NLM Title Abbreviation: Innov Surg Sci
Journal ID: Innov Surg Sci
NLM Journal ID: 101708165
Journal ID: iss

Innovative Surgical Sciences

EISSN: 2364-7485
Publisher: De Gruyter

ARTICLE INFORMATION
==============================
PMCID: PMC13068494
DOI: 10.1515/iss-2026-2002
Article ID: iss-2026-2002
Article version: 1
Subjects: Article

2. Visceral Surgery

Electronic publication date: 2026 Apr 10
Publication date: 2026 Apr
Volume: 11
Issue: Suppl1
First page: 77
Last page: 269
Copyright: © 2026 by Walter de Gruyter Berlin/Boston
Copyright year: 2026
Copyright holder: De Gruyter
License: This work is licensed under the Creative Commons Attribution 4.0 International License.
License URL: https://creativecommons.org/licenses/by/4.0/

Page count: 193

==============================

DE GRUYTER BRILL

**Biliary Reflux After OAGB: Effectiveness of Conversion to Roux-en-Y Gastric Bypass**
ID: E-DCK-132

**Viszeralchirurgie / Visceral Surgery**
**Adipositastherapie und metabolische Chirurgie (CAADIP) / Obesity Treatment and Metabolic Surgery (CAADIP)**

Robin Wippich[1], Johannes Sander[1]

[1]Schön-Klinik Hamburg-Eilbek, Adipositasklinik, Hamburg, Deutschland

**Background:**
One-anastomosis gastric bypass (OAGB) has become an established procedure in the surgical treatment of obesity with comparable results to Roux-Y gastric bypass in terms of weight loss and improvement of obesity-associated comorbidities.
Biliary reflux is a long-term complication of OAGB. In addition to restricted quality of life, it is feared that chronic biliary reflux promotes development of gastric or esophageal carcinomas.

**Material and methods:**
Frequency of clinically relevant biliary reflux after OAGB and its conservative and surgical therapeutic options and their effectiveness.

Retrospective evaluation of **3913** patients who underwent OAGB as primary surgery at Schoen Clinic Hamburg Eilbek (Germany) between September 2016 and September 2025. Evaluation of the frequency of clinically relevant biliary reflux, which led to surgical revision with conversion of the OAGB into a Roux-Y-situation after conservative therapy options had been exhausted.

**Results:**
In the follow-up period up to September **2025**, a total of **196** patients (**5,01**%) were converted into Roux-Y gastric bypass. In **165** patients (**4,22**%) biliary reflux was predominant and the biliary loop was distalized. In addition, **23** patients (**0,59**%) had a chronic anastomotic ulcer, which led to a resection of the gastro-jejunostomy and a new anastomosis was performed simultaneously. Postoperative early complications (< 30 days) occurred in one patient (**0,51**%) with hematemesis, two patients (**1,04**%) with bleeding at the side of the jejuno-jejunostomy which required a laparoscopic revision and **four** patients (**2.07**%) with abdominal wall hematoma. Late complications observed: persistent reflux symptoms n=2 (**1,04**%) and dumping syndrome n=**2** (**1.04**%). Recurrence of anastomotic ulcer n=**5** (**2,55**% of revisional cases resp. **0,13**% of all cases).

**Conclusion:**
Biliary reflux is a rare long-term complication after OAGB. If conservative therapy attempts fail in the long term and the level of suffering is high, conversion into a Roux-Y- situation is a successful treatment option with low complication rates and high patient satisfaction.

Open Access. © 2026 The Author(s), published by De Gruyter. This work is licensed under the Creative Commons Attribution 4.0 International License.

DE GRUYTER BRILL

**Pharmacotherapy, Prehab, Prognosis: A Systematic Review of Metabolic-Bariatric Preconditioning**
ID: E-DCK-153

**Viszeralchirurgie / Visceral Surgery**
**Adipositastherapie und metabolische Chirurgie (CAADIP) / Obesity Treatment and Metabolic Surgery (CAADIP)**

Jacques Hilbert[1], Gabriel Seifert[1]

[1]Universitätsklinikum Freiburg, Freiburg im Breisgau, Deutschland

**Background:**
Obesity has recently been defined as the presence of excess adipose tissue with an increased risk of associated local or systemic cell and tissue dysfunction leading to preclinical and clinical forms of obesity. IFSO recently embraced multimodal therapy approaches including surgical and non-surgical modalities. It is known that Metabolic bariatric surgery (MBS) still is the most effective treatment for sustained weight loss and improvement in obesity-related comorbidities. However, outcomes vary, and patients with metabolic disease face higher risks of complications and poor recovery. Presurgical weight gain is linked to suboptimal postoperative results, prompting greater focus on prehabilitation. Traditionally, this involves dietary changes, exercise, and psychological support. Recently, obesity management medications (OMMs) have emerged as potential tools to enhance preoperative outcomes, yet research on their use before MBS remains limited. Pharmacological prehabilitation may improve adipose tissue loss, glycemic control, inflammation, and respiratory function —especially in patients with complex medical profiles. This systematic review evaluates current evidence on OMMs in preoperative care for MBS, assessing their impact on surgical outcomes and identifying gaps to guide future research and individualized treatment strategies.

**Material and methods:**
We conducted a systematic search of MEDLINE, Cochrane Central, EMBASE, and Web of Science for studies on pharmacological prehabilitation before metabolic bariatric surgery (MBS), with emphasis on GLP-1 receptor agonists (GLP1RA). Search terms included “bariatric surgery,” “preoperative,” and “GLP1-agonists,” refined via Ovid MEDLINE. Studies published in English up to January 28, 2025, were screened for relevance to pre-, intra-, and postoperative outcomes. Abstracts were initially reviewed, followed by full-text screening by two independent reviewers (JH, GS), with disagreements resolved through discussion. Of 754 identified articles, 67 underwent full-text review, and 14 met inclusion criteria. Exclusions were due to topic deviation (n=16), conference abstracts (n=18), non-English language (n=15), and lack of original research (n=5). Studies were assessed using GRADE. Data extraction included study design, population, outcomes, and statistical methods. Gender parity was achieved across most studies. Due to heterogeneity and limited comparable data, meta-analysis was not feasible.

**Results:**
We analyzed 14 studies (9,587 participants), predominantly US-based cohorts (12 of 14) led by Lewis et al. (n = 4,157). Nine studies examined six preoperative outcomes, with five assessing BMI changes after GLP1RA prehabilitation. Four studies evaluated intraoperative metrics, and six studied four postoperative outcomes, including four on postoperative BMI following GLP1RA prehabilitation.

Preoperative Outcomes:
Eight studies explored how pharmacological prehabilitation shapes the pre-surgery landscape. Most focused on GLP-1 receptor agonists (GLP1RAs) and consistently showed that three to six months of therapy slashes BMI—one report even found an average drop of 5.5 kg/m² versus 2.9 kg/m² in people who didn't receive the drug. Sibutramine delivered a smaller but still significant six-week effect (–1.6 kg/m² vs +2.9 kg/m²), and exenatide drove steady weight loss (–4.1 kg in one month, –8.9 kg in three, –12.6 kg in six) alongside shrinking waistlines.
Two groups tracked blood sugar changes and found that around a 1 percent reduction in HbA1c was achievable after three or six months of GLP1RA, with no clear extra benefit from the longer course. A small proof-of-concept trial showed a combination of liraglutide, amino acids, and a low-calorie diet outperformed an intragastric balloon for both BMI and HbA1c reduction. Interestingly, nearly 70 percent of patients who lost more than 15 percent of their bodyweight on GLP1RA decided against surgery, even though most said they'd be open to medications as an extra boost.
Intraoperative Outcomes:
Four cohort studies looked at the operating room. Six weeks of sibutramine shortened Roux-en-Y bypass time by about 20 minutes without raising complication rates. By contrast, six months of liraglutide added roughly 13 minutes to sleeve gastrectomy and revealed more adhesions, possibly tied to drug-induced pancreatic inflammation. A separate investigation into semaglutide use before body-contouring surgery after MBS linked it to higher rates of wound dehiscence and delayed healing, although confounding factors weren't fully accounted for. And when teams examined pre-surgery HbA1c levels in diabetic patients, they found no consistent link to perioperative complications.
Postoperative Outcomes:
Six studies followed patients after surgery. Preoperative GLP1RA gains didn't always translate into extra pounds shed post-op—results varied by cohort. One group saw lower 12-month BMI in the liraglutide prehab arm, while another noted smaller BMI drops at one and six months. When compared directly, intragastric balloon therapy had higher excess weight loss than liraglutide but carried a higher complication rate. Phentermine–topiramate prehabilitation, on the other hand, led to better total weight loss at one year.
In type 2 diabetes, the story gets more promising: each 1 percent drop in HbA1c before surgery predicted an additional 0.3 percent reduction afterward, and insulin-dependent patients who did a GLP1RA prehab saw remission rates jump from 4 percent to 22 percent. Overall, drugs like GLP1RAs and sibutramine clearly reshape the pre- and post-surgery journey—but the impact on long-term weight outcomes is still a mixed bag.

**Conclusion:**
This review highlights pre-surgical interventions: GLP-1 receptor agonists enhance preoperative and cumulative weight loss, although current evidence remains limited. Further research should assess whether weight responses to preoperative obesity medications predict outcomes after metabolic bariatric surgery. A drop in preoperative HbA1c strongly forecasts postoperative glycemic improvement and T2D remission. No guidelines currently address combining obesity medications with surgery. These findings underscore the urgent need for early-phase adequately powered large-scale well-designed multicenter RCTs to establish optimal treatment duration and medication regimens to maximize total weight loss from enrollment and achieve early disease control before comorbidities develop.

DE GRUYTER BRILL

**RYGB vs. SG in BMI >50kg/m² - five year follow up results from an international multicentre cohort**
ID: E-DCK-24

**Viszeralchirurgie / Visceral Surgery**
**Adipositastherapie und metabolische Chirurgie (CAADIP) / Obesity Treatment and Metabolic Surgery (CAADIP)**

Lars Kollmann[1], Ilan Rosenblum[2], Reinhard Stoll[2], Robert Rosenberg[2], Erik Stenberg[3], Torsten Olbers[4], Ralph Peterli[5], Florian Seyfried[1], Anas Taha[2]

[1]Universitätsklinikum Würzburg, Department of General-, Visceral-, Transplantation-, Vascular-, and Pediatric Surgery, Würzburg, Deutschland, [2]Kantonsspital Baselland, Klinik für Chirurgie und Viszeralchirurgie, Liestal, Schweiz, [3]University Örebro, Örebro, Sweden, [4]Linköping University, Department of Surgery and Department of Clinical and Experimental Medicine, Norrköping, Sweden, [5]Universitätsspital Basel, Basel, Schweiz

**Background:**
Patients with an initial body-mass-index (BMI) >50kg/m² are vastly underrepresented in randomized controlled trails demonstrating similar weight loss and diabetes remission rates after sleeve gastrectomy (SG) and Roux-en-Y-gastric-bypass (RYGB).

**Material and methods:**
Propensity matching was used to compare outcomes regarding weight loss and diabetes control after SG and RYGB in patients with a BMI >50kg/m² in an international database from six European countries.

**Results:**
In total, 3976 of 8160 patients were used for the analysis (1988 each after SG and RYGB). Median BMI and mean age at baseline were 55.5kg/m² (range 50-100kg/m²) and 39.9 years (range 15-76), respectively. Median BMI after one- and five-years were 39.3kg/m² (22-64.7), and 41.6kg/m² (22.0 – 67.2) in the SG group and 37.5kg/m² (23.7-59.7) and 38.7kg/m² (20–63.8) in the RYGB group (p<.001 between groups). Accordingly, %TBWL at one and five years after SG was 30.2% (2.2-63.7) 25.4% (4.8 – 56.0) versus 31.2% (7.4-54.5) and 28.2% (-6.6 – 62.9) in the RYGB group (p<.001). The prevalence of a persistent BMI>40kg/m² after one and five years was 42.7% and 57.6% after SG versus 24.5 % and 39.2% after RYGB (p<.001). The prevalences of a normal HbA1c (<6.5%) were 12.9% after SG and 11.6% after RYGB group (p=0.323) after five years.

**Conclusion:**
This study suggests that Roux-en-Y gastric bypass leads to a greater weight loss than sleeve gastrectomy in patients with a BMI above 50kg/m² while improvements in diabetes appear to be similar. The large proportion of patients still having a BMI>40 kg/m² at five years after surgery suggests implementation of adjuvant treatment regimens.

DE GRUYTER BRILL

**Treatment pathways and outcomes in patients with BMI ≥ 50 kg/m²**
ID: E-DCK-240

**Viszeralchirurgie / Visceral Surgery**
**Adipositastherapie und metabolische Chirurgie (CAADIP) / Obesity Treatment and Metabolic Surgery (CAADIP)**

Sara Notz[1], Rainer Grotelueschen[1], Julia Pape[1], Björn-Ole Stueben[2 3], Louisa Stern[3], Julia Gerullies[1], Anne Lautenbach[4], Thilo Hackert[1], Philipp Busch[1], Anna Duprée[1], Dieter Weber[3], Oliver Mann[1], Gabriel Plitzko[1 3]

[1]University Medical Center Hamburg-Eppendorf, Department of General, Visceral and Thoracic Surgery, Hamburg, Deutschland, [2]Medical Center University Duisburg-Essen, Department of General-, Visceral- and Transplant Surgery, Essen, Deutschland, [3]Royal Perth Hospital, Department of General Surgery, Perth, Australia, [4]University Medical Center Hamburg-Eppendorf, III Department of Medicine, Hamburg, Deutschland

**Background:**
In the developed world, obesity is one of the greatest public health problems. The global prevalence of obesity has increased substantially over the past 40 years. Obesity is associated with an increased risk for type 2 diabetes, hypertension, dyslipidaemia, cardiovascular diseases, certain types of cancer and mortality. Treatment options for obesity include non-surgical treatment and bariatric surgery.
Bariatric surgery is the most effective treatment for patients with a BMI ≥50 kg/m², yet preoperative conservative therapy is often mandated. The benefit of such an approach remains controversial. This study aimed to compare outcomes of conservative treatment alone, immediate surgery, and stepwise surgery following failed conservative therapy in patients with BMI ≥50 kg/m².

**Material and methods:**
All patients with BMI ≥50 kg/m² presenting to a German bariatric center between 2015–2021 were retrospectively categorized into three groups: Non-Surg (conservative therapy only), Surg-First (immediate surgery), and Step-Treat (initial conservative therapy followed by surgery). Primary outcomes were assessed at 6, 12, and 24 months and included weight loss, resolution of obesity-associated comorbidities (hypertension, T2DM , dyslipidemia, OSAS), and overall outcome measured by the SF-BARI score.

Abstracts – DCK 2026 – 143rd Congress of the German Society of Surgery – Leipzig, April 22–24 • DOI 10.1515/iss-2026-2002
Innov Surg Sci 2026; 11 (Special Suppl 1): s77–s269

Figure 1

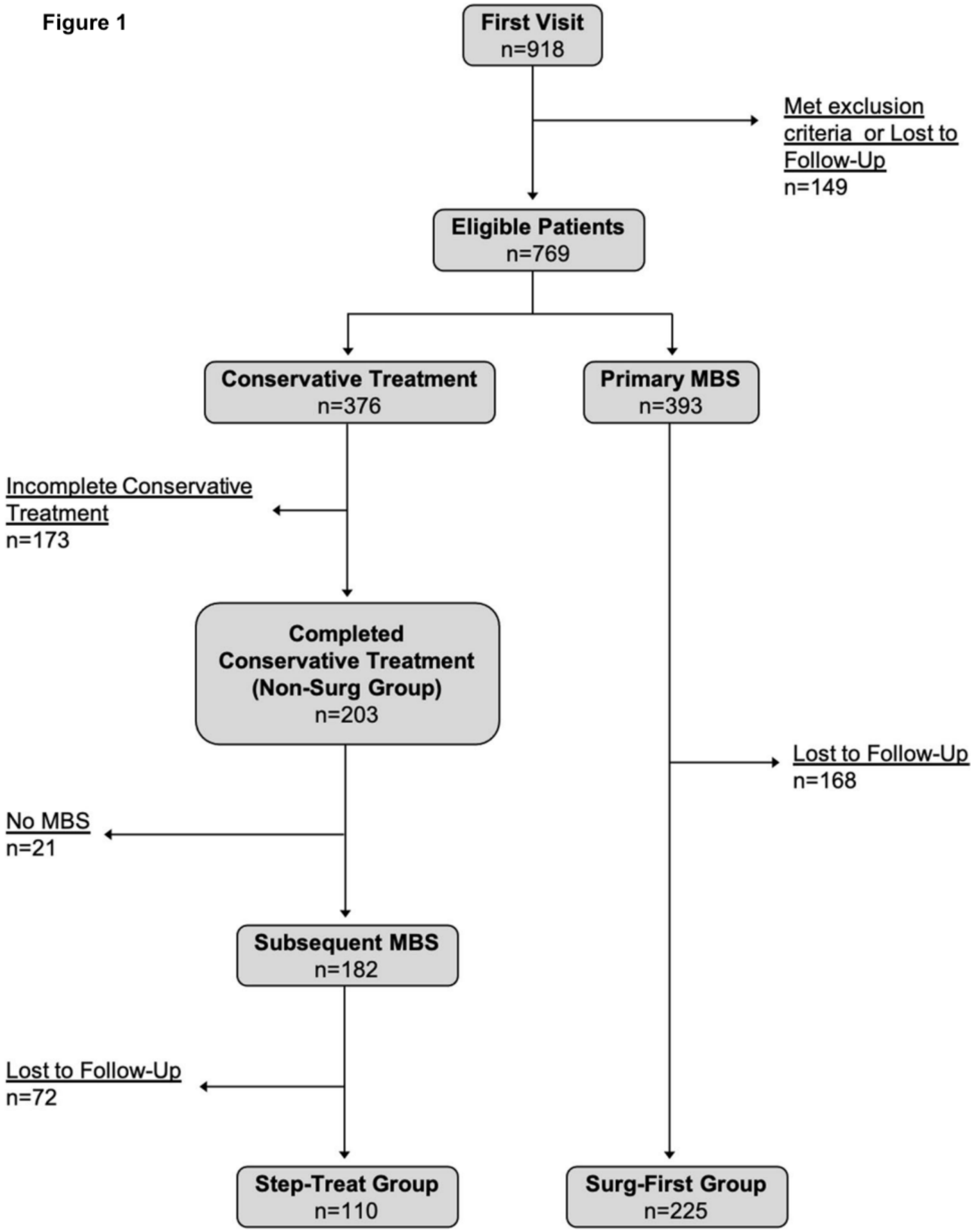

**Results:**
Of 918 screened patients, 538 met inclusion criteria and were allocated to Non-Surg (n=203), Surg-First (n=225), and Step-Treat (n=110) (Fig. 1). After 6 months, median %TWL and %EWL were significantly higher in the Surg-First group (%TWL: 23.6% vs. 0%; %EWL: 42% vs. 0%; both $P < .001$) compared to Non-Surg (Tab. 1). Only 3% of Non-Surg patients achieved adequate weight loss. Surg-First patients also showed higher remission rates of T2DM (54.1% vs. 21.2%; P < .001) and fewer de novo T2DM cases. Compared to Surg-First, the Step-Treat group showed comparable short-term weight loss, but significantly lower %TWL and %EWL at 12 and 24 months. Severe postoperative complications (Grade IIIb) were more common in Step-Treat (11.8% vs. 3.6%; P < .001), and SF-BARI scores were significantly higher in Surg-First, indicating superior overall outcomes (Tab. 2).

**Table 1:** Weight outcomes and changes in metabolic parameters in the Non-Surg and Surg-First group 6 months after treatment.

| | Non-Surg | Surg-First | P value |
|---|---|---|---|
| n | 203 | 225 | |
| **Weight Data** | | | |
| Weight loss (kg) | 0 (-4 to 6) | -37 (-47 to -31) | <0.001 |
| BMI (kg/m²) | 54.7 (51.2 to 59) | 42.6 (38.3 to 46.6) | <0.001 |
| %TWL (kg) | 0 (-3.5 to 2.6) | 23.6 (19 to 28.4) | <0.001 |
| %EWL (%) | 0 (-7 to 5) | 42 (34 to 51) | <0.001 |
| **Metabolic Parameters** | | | |
| HbA1c (%) | 6.7 (6.5 to 7.8) | 6.6 (5.8 to 7.2) | 0.15 |
| LDL-C (mg/dl) | 132 (121 to 150) | 138.5 (121.8 to 157) | 0.21 |

Normally distributed data are presented as mean ± standard deviation (SD), non-normally distributed data as median (IQR).

**Conclusion:**

In patients with BMI ≥50 kg/m², immediate bariatric surgery was associated with superior weight loss, improved comorbidity resolution, and fewer complications compared to conservative therapy alone or a stepwise approach. These findings support direct surgical treatment in this population.

**Table 2:** Operative and postoperative metrics of Step-Treat und Surg-First group.

| | Step-Treat | Surg-First | P value | OR | 95% CI |
|---|---|---|---|---|---|
| n | 110 | 225 | | | |
| **Operating time (min)** | 78.5 (64.75 to 94.5) | 79 (66.5 to 94.5) | 0.39 | N/A | N/A |
| **Length of stay (d)** | 6 (5 to 7) | 6 (5 to 7) | 0.25 | N/A | N/A |
| **Procedure (n, %)** | | | | | |
| SG | 96 (87.2) | 189 (84) | 0.43 | 0.77 | 0.39 to 1.5 |
| RYGB | 14 (12.8) | 36 (16) | | | |
| **Complications (n, %)** | 25 (22.6) | 37 (16.4) | 0.16 | 0.67 | 0.38 to 1.2 |
| I | 4 (3.6) | 16 (7.1) | 0.33 | 2.1 | 0.66 to 6.2 |
| II | 5 (4.5) | 11 (4.8) | 0.89 | 1.1 | 0.05 to 1.2 |
| IIIa | 2 (1.8) | 2 (0.9) | 0.6 | 0.48 | 0.07 to 3.5 |
| IIIb | 13 (11.8) | 8 (3.6) | <0.001 | 0.02 | 0.006 to 0.06 |
| IVa | 0 (0) | 0 (0) | 0.43 | 0.83 | 0.53 to 1.3 |
| IVb | 1 (0.9) | 0 (0) | 0.33 | 0.83 | 0.97 to 1.0 |
| V | 0 (0) | 0 (0) | N/A | N/A | N/A |
| **SF Bari Score** | 85.9 ± 28.9 | 98.9 ± 30.3 | <0.001 | N/A | N/A |
| Excellent (n, %) | 6 (5.5) | 24 (10.7) | 0.12 | 2.07 | 0.82 to 5.2 |
| Very good (n, %) | 19 (17.3) | 66 (29.4) | 0.02 | 1.9 | 1.1 to 3.5 |
| Good (n, %) | 52 (47.3) | 96 (42.7) | 0.43 | 0.83 | 0.53 to 1.3 |
| Fair (n, %) | 30 (27.2) | 33 (14.6) | 0.006 | 0.46 | 0.26 to 0.8 |
| Suboptimal (n, %) | 3 (2.7) | 6 (2.6) | 0.97 | 0.98 | 0.24 to 3.9 |

Normally distributed data are presented as mean ± standard deviation (SD), non-normally distributed data as median (IQR).
The complications are reported according to the Clavien-Dindo classification. The percentage of each category refers to the total number of complications.

DE GRUYTER BRILL

**Development of a predictive model for postoperative BMI and Diabetes mellitus outcomes**
ID: E-DCK-26

**Viszeralchirurgie / Visceral Surgery**
**Adipositastherapie und metabolische Chirurgie (CAADIP) / Obesity Treatment and Metabolic Surgery (CAADIP)**

Ilan Rosenblum[1], Vincent Ochs[2], Lars Kollmann[3], Reinhard Stoll[1], Robert Rosenberg[1], Erik Stenberg[4], Ralph Peterli[5], Anas Taha[1]

[1]Kantonsspital Baselland, Klinik für Chirurgie und Viszeralchirurgie, Liestal, Schweiz, [2]Universität Basel, Basel, Schweiz, [3]Universitätsklinikum Würzburg, Department of General-, Visceral-, Transplantation-, Vascular-, and Pediatric Surgery, Würzburg, Deutschland, [4]University Örebro, Örebro, Sweden, [5]Universitätsspital Basel, Basel, Schweiz

**Background:**
Bariatric surgery is currently the most efficient treatment option for obesity and associated comorbidities, including Type 2 diabetes mellitus (T2D). However, predicting long-term outcomes such as postoperative Body Mass Index (BMI) trajectories and T2D remission remains challenging. Accurate forecasts are essential for guiding clinical decisions and personalizing follow-up care.

**Material and methods:**
This multicenter retrospective study included 9652 adult patients who underwent bariatric surgery between 2012 and 2023 in eleven centers in Europe. A two-stage machine learning framework was developed. The first stage used ensemble-based regression models (XGBoost, Random Forest, HistGradient Boosting, Multilayer Perceptron) to predict postoperative BMI at eight intervals up to 5 years. The second stage classified T2D remission (XGBoost, Random Forest, Hist Gradient Boosting, Multilayer Perceptron) using preoperative data and predicted BMI trajectories. Data preprocessing included iterative imputation, SMOTETomek resampling, polynomial feature expansion, normalization, and one-hot encoding. Model performance was evaluated using 10-Fold Cross Validation. A web-based application was developed to deliver individually accessible predictions.

**Results:**
The regression model achieved an root mean squared error (RMSE) of 2.93 kg/m² and median absolute deviation (MAD) of 1.99 kg/m² at 1 year, and 3.60 kg/m² and 1.98 kg/m², respectively, at 5 years. The T2D classification model reached a macro-averaged F1 score of 0.92, with 5-year precision and recall of 0.93 and 0.88. Inclusion of dynamic BMI predictions significantly improved classification performance over baseline-only models. Model performance was consistent across clinical sites and surgical subtypes by assessing the difference between the predicted and actual values.

**Conclusion:**
This study demonstrates that integrating dynamic BMI trajectories with preoperative data enhances prediction of long-term metabolic outcomes after bariatric surgery. The model supports personalized clinical decision-making. A web application was constructed to enable real-time, patient-specific risk assessment. These findings highlight the potential of machine learning to improve postoperative care and long-term outcomes in obesity treatment.

DE GRUYTER BRILL

**The Treatment of Lipedema and Concomitant Obesity: A Modified Delphi Consensus Statement**
ID: E-DCK-68

**Viszeralchirurgie / Visceral Surgery**
**Adipositastherapie und metabolische Chirurgie (CAADIP) / Obesity Treatment and Metabolic Surgery (CAADIP)**

Sjaak Pouwels[1], Jeremias Schmidt[2], Chetan Parmar[3], Mojtaba Ghods[4]

[1]Universitaetsklinik OWL der Universitaet Bielefeld - Campus Klinikum Lippe, Detmold, Deutschland, [2]Helios Berlin Buch, Berlin, Deutschland, [3]Whittington Hospital, London, Großbritannien, [4]Ernst von Bergmann Klinikum, Potsdam, Deutschland

**Background:**
Despite numerous advances in the treatment of obesity and lipedema, there is no published consensus amongst experts on how to treat patients with both obesity and lipedema. The aim of this modified Delphi consensus is to generate a set of principles, which could support the diagnosis and treatment of patients with concomitant obesity and lipedema and could guide future research.

**Material and methods:**
The expert committee consisted of 44 experts on obesity and lipedema treatment from nine specialties from twenty countries. The project was coordinated by the steering committee of 3 members. Statements were based on aetiology, diagnosis and treatment of lipedema and obesity.

**Results:**
A total of 86 statements were voted upon in the two rounds. In the first round of the Delphi process, 44 experts voted on 72 statements. After the first round of voting, 55 statements reached a consensus ≥ 70.0% among the expert committee, whereas, 17 statements did not reach consensus. These 17 statements were modified based on feedback and hence there were 14 statements in the second round. Of the statements voted on in the second round, four reached consensus. After two rounds of voting consensus were reached on 59 of the in total 86 statements (68.6%).

**Conclusion:**
The treatment of patients with obesity and concomitant lipedema should be multidisciplinary with frequent reassessments of treatment effects. Regarding the pathophysiology and the possible link between the two diseases, more research is needed.

DE GRUYTER BRILL

**Diagnosis and Treatment of Chronic Abdominal Pain after Bariatric Surgery: A Systematic Review**
ID: E-DCK-69

**Viszeralchirurgie / Visceral Surgery**
**Adipositastherapie und metabolische Chirurgie (CAADIP) / Obesity Treatment and Metabolic Surgery (CAADIP)**

Sjaak Pouwels[1], Alba Zevallos[2]

[1]Universitaetsklinik OWL der Universitaet Bielefeld - Campus Klinikum Lippe, Detmold, Deutschland,
[2]Loma Linda University Medical center, Loma Linda, Vereinigte Staaten

**Background:**
Bariatric surgery effectively manages morbid obesity and related comorbidities but can result in chronic abdominal pain (CAP) for some patients, affecting their quality of life and postoperative care. CAP has an incidence rate of 30% to 50% and is a common cause of emergency visits and readmissions, increasing healthcare costs.

**Material and methods:**
The etiology of CAP is multifactorial, involving anatomical causes like adhesions and internal hernias, functional disorders, biliary pathologies, nutritional disorders, abdominal wall pathologies, and miscellaneous causes. Accurate diagnosis requires a structured approach that combines a thorough clinical history, physical examination and laboratory evaluation with targeted diagnostic modalities including imaging studies such as computed tomography (CT), upper gastrointestinal series, abdominal ultrasound and endoscopy. Management of CAP after bariatric surgery requires an individualized, multidisciplinary approach with surgical interventions, endoscopic therapies, medical management, dietary interventions, physical therapy and behavioral therapy.

**Results:**
This systematic review examines the multifactorial causes of CAP after bariatric surgery and provides a comprehensive overview of current diagnostic strategies and therapeutic approaches.

**Conclusion:**
A systematic, multidisciplinary framework is essential to improve outcomes and reduce the burden of CAP in the bariatric population.

DE GRUYTER BRILL

**The effect of rapeseed oil in patients with obesity undergoing sleeve gastrectomy**
ID: E-DCK-95

**Viszeralchirurgie / Visceral Surgery**
**Adipositastherapie und metabolische Chirurgie (CAADIP) / Obesity Treatment and Metabolic Surgery (CAADIP)**

Patricia Widmayer[1], Martina Feger[1], Emre Tanay[2], Julian Hornung[2], Tobias Meile[3], Nicklas Griese[1], Gabriele Stangl[4], Frank Hirche[4], Marvin Schmitt[5], Michael Föller[6]

[1]Universität Hohenheim, Stuttgart, Deutschland, [2]Klinikum Stuttgart, Stuttgart, Deutschland, [3]Adipositaspraxis Reutlingen, Reutlingen, Deutschland, [4]3Martin Luther University Halle-Wittenberg, Institute of Agricultural and Nutritional Sciences, Halle (Saale), Germany, Saale, Deutschland, [5]4University of Stuttgart, Cluster of Excellence SimTech, Stuttgart Center for Simulation Science, Germany, Stuttgart, Deutschland, [6]University of Hohenheim, Department of Physiology, Stuttgart, Germany, stuttgart, Deutschland

**Background:**
Obesity is associated with dysregulated ghrelin secretion, impaired nutrient sensing, and aberrant inflammatory responses.
The objective of this randomized controlled trial was to assess effects of rapeseed oil (RO) supplementation on top of a calorie-restricted (1200 kcal/day) high protein (120 g/day) diet prior sleeve gastrectomy on gastric cellular and molecular changes of ghrelin, nutrient receptors and inflammatory markers, and on circulating ghrelin and chemokines.

**Material and methods:**
Thirty-six patients with obesity (50-81.2 kg/m$^2$) were divided into two groups: ND (normal preoperative diet; n=18) and ND+RO (normal preoperative diet + RO containing α-linolenic acid 2.9 g/day; n=18).

**Results:**
After four weeks, RO supplementation enhanced ghrelin$^+$ cell density. The number of ghrelin$^+$ cells was greater in women than men receiving ND+RO. In men on ND+RO the number of ghrelin$^+$ cells expressing the fatty acid sensing receptor GPR120 was enhanced, whereas calcium sensing receptor (CaSR) expressing ghrelin$^+$ cells remained unaffected. Men receiving ND+RO had less immune cell aggregates and macrophage infiltration, lower TNF-α expression, and fewer endothelial cells compared to men consuming the ND. In contrast, female participants on ND+RO showed strong interindividual differences with only a few women exhibiting less immune cell infiltrates. RO consumption did not alter plasma acyl-ghrelin, MCP-1 and IL-8 levels.

**Conclusion:**
Four weeks of supplementation with rapeseed oil had a local beneficial effect of limiting inflammation in the gastric mucosa of men, while the dietary intervention had no effect on inflammatory cytokines in the blood. In women who received ND+RO only a few showed signs of resolving inflammation. Higher initial infiltration levels may have potentially influenced the response to the diet.

DE GRUYTER BRILL

**Staging Accuracy in Esophageal Cancer – Insights from Data-Driven Analysis and Clinical Practice**
ID: E-DCK-141

**Viszeralchirurgie / Visceral Surgery**
**Chirurgie oberer Gastrointestinaltrakt (CAOGI) / Upper Gastrointestinal Surgery (CAOGI)**

Wael Rayya[1], Ioannis Pozios[1], Christian Schineis[1], Jonas Staudacher[2], Christoph Treese[2], Swantje Malinka[1], Vanessa Pross[3], Pia Jäger[1 3 4]

[1]Charité Berlin Campus Benjami Franklin, Klinik für Allgemein- und Viszeralchirurgie, Berlin, Deutschland, [2]Medizinische Klinik für Gastroenterologie, Infektiologie und Rheumatologie (einschl. Arbeitsbereich Ernährungsmedizin), Berlin, Deutschland, [3]Charité Berlin Campus Benjamin Franklin, Allgemein- und Viszeralchirurgie - Chirurgisches Forschungslabor, Berlin, Deutschland, [4]Berlin Institute of Health at Charité – Universitätsmedizin Berlin, Berlin, Deutschland

**Background:**
Accurate staging of esophageal and AEG cancer patients is crucial in achieving the best possible outcome while avoiding the unnecessary side effects of overtreatment. This is particularly important as curative treatment mostly includes a surgical resection associated with a high operative risk.
To assign patients to the proper treatment scheme, the most important factors are the histological entity, localisation and T-stage, which are decisive in recommending neoadjuvant therapy. According to the German S3 guideline, patients with cT2 tumours may receive preoperative chemotherapy (adenocarcinoma) or radiochemotherapy (squamous cell carcinoma). In locally advanced stages (cT3/T4 or cN+), perioperative chemotherapy or preoperative radiochemotherapy is recommended, depending on histology.
However, accurate staging, particularly of the T-stage, is challenging, since preoperative diagnostic methods have limited accuracy.
This study aims to analyse staging accuracy in patients who undergo surgery for esophageal or AEG III cancer.

**Material and methods:**
The database is the German nationwide StuDoQ register, and includes patients who undergo surgical therapy for esophageal cancer or AEG cancer between 2021 and 2024. Factor analysis and multivariate regression are performed to determine the influencing factors correlated with inadequate therapy allocation, defined as advanced tumour stage in histopathology (≥ pT3) without neoadjuvant therapy. The findings are discussed in the context of recent findings. Additionally, clinical experiences and best practice approaches in the clinical practice of a university treatment centre are presented.

**Results:**
The study group comprises 1,345 people who undergo surgery for esophageal (n = 1155) or AEG (n = 190) cancer. The vast majority (98.96%) of these patients are included due to elective surgery, while 14 patients undergo emergency surgery. Data regarding neoadjuvant therapy and tumour regression is applicable in 1,332 patients (99.03%). The majority of surgical patients (n = 1,094, 81.34%) undergo preoperative neoadjuvant therapy. Lymph node infiltration (defined as N+ with at least one infiltrated lymph node) is found histologically in 44.24% (n = 511) of patients with esophageal cancer and 47.37% (n = 90) of patients with AEGIII cancer.
Conversely, 17.70% (n = 238) of surgical patients undergo surgery without receiving neoadjuvant therapy. Surgical histopathology confirms a tumour of T2 or smaller in only 53.36% (n = 127) of patients who do not receive neoadjuvant therapy, tumour invasion classified as at least T3 in 43.70% (n = 104) of patients, and a non-classifiable T stage in 1.74% (n = 7) of patients.
In multivariable logistic regression (modest fit with Nagelkerke's $R^2$ = 0.12), higher age is significantly associated with incorrect staging (OR 1.09, 95% CI 1.06–1.12, p< 0.001). Patients with poor performance status (ECOG 3) also show markedly increased odds of staging inaccuracy (OR 9.04, 95% CI 2.02–36.6, p = 0.002). The patients' BMI demonstrates a borderline inverse association (OR 0.94, 95% CI 0.89–1.00, p = 0.054), and the histological classification "squamous cell carcinoma" shows a trend without reaching significance (OR 0.51, 95% CI 0.23–1.04, p = 0.072).

**Conclusion:**

The current accuracy of pre-surgical T staging remains unsatisfactory: 104 patients (7.7% of the total cohort) are retrospectively classified as undertreated, and specifically among those who undergo primary surgery without neoadjuvant therapy, more than 40% are, according to postoperative histopathology, appropriate candidates for such treatment. Patients who are old, in poor general condition and overweight or obese might be particularly effected.
To address these shortcomings, an interdisciplinary group is established to develop research approaches, validate diagnostic strategies, and refine clinical practice recommendations, in order to improve staging accuracy and optimise treatment allocations, and consequently, therapeutic outcome.

DE GRUYTER BRILL

### Longterm Quality of Life after MIS and Open Oncological Surgery of the Upper Gastrointestinal Tract

ID: E-DCK-179

**Viszeralchirurgie / Visceral Surgery**
**Chirurgie oberer Gastrointestinaltrakt (CAOGI) / Upper Gastrointestinal Surgery (CAOGI)**

Dolores Krauss[1], Katharina Bahde[1], Jennifer Eckhoff[1], Stefanie Brunner[1], Lars Schiffmann[1], Thomas Schmidt[1], Christiane J. Bruns[1], Hans Fuchs[1]

[1]Uniklinik Köln, Klinik und Poliklinik für Allgemein-, Viszeral-, Thorax- und Transplantationschirurgie, Köln, Deutschland

**Background:**
Oncological surgery of the upper gastrointestinal tract includes complex procedures like gastrectomies, transhiatal gastrectomies and esophagectomies, changing the GI tract significantly. A reduced quality of life (QoL) and a variety of symptoms including dysphagia, reflux, bloating and pain are common and often difficult to address surgically and pharmacologically. Aim of this study was to provide an overview and comparison of the quality of life of patients after oncological surgery of the upper gastrointestinal tract including not only the different surgical approaches but also minimally invasive and robotic techniques.

**Material and methods:**
Patients undergoing oncological surgery of the upper gastrointestinal tract are postoperatively followed up in a standardized fashion, including scheduled CT scans, EGDs and assessment of QoL. QoL is evaluated using the validated and organ-specific EORTC questionnaire of the European Organisation for Research and Treatment of Cancer (EORTC-QLQ-C30). Retrospective analysis of prospective collected data in our IRB approved database was performed of all patients with complete assessment of QoL after robotic (RAMIE), minimally invasive (laparoscopic/thoracoscopic - MIS), hybrid (laparoscopic/open) and open esophagectomy, open gastrectomy and open transhiatal esophagectomy. For the EORTC-QLQ-C30 the raw score was calculated first, then linear transformation was performed to obtain score S. Unpaired t test was performed to analyse statistical significance. A p value of <0.01 was considered statistically significant.

*Table 1: Summary of QoL assessment of different oncological surgeries and mean follow up times.*

| | RAMIE* | Total MIS* | Hybrid* | Open* | Gastrectomy* | THG* |
|---|---|---|---|---|---|---|
| **Patients (n)** | 65 | 24 | 101 | 15 | 37 | 40 |
| **Mean age in years (range)** | 62.508 (43-79) | 61.021 (46-80) | 53.703 (39-84) | 65.2 (54-77) | 63.270 (21-82) | 69.525 (45-87) |
| **Gender (F/M)** | M =54 F = 11 | M = 22 F = 2 | M = 85 F = 16 | M = 11 F = 4 | M = 20 F = 17 | M = 30 F = 10 |
| **Mean time of follow up in days (range)** | 730.246 (83-1932) | 723.833 (99-1645) | 816.644 (30-3892) | 467.267 (97-2052 | 765.676 (116-2063) | 823.65 (100-3743) |

**RAMIE – robotic assisted minimally invasive Ivor Lewis esophagectomy*
*Total MIS – minimally invasive laparoscopic/thoracoscopic Ivor Lewis esophagectomy*
*Hybrid – laparoscopic abdominal part, open thoracic part Ivor Lewis esophagectomy*
*Open – open Ivor Lewis esophagectomy*
*Gastrectomy – D2 gastrectomy*
*THG – D2 transhiatal gastrectomy*

**Results:**
A total of 282 patients were evaluated. Demographics, type of surgery and mean follow-up times are depicted in table 1. Results of the EORTC-QLQ-C30 for patients undergoing an esophagectomy are shown in table 2. While no significant change in the overall health score was seen, the robotic technique was associated with a significantly better social functioning, while the hybrid technique was associated with a significantly better cognitive and emotional functioning. Table 3 shows a comparison of results for patients undergoing a D2 gastrectomy compared to patients undergoing a transhiatal gastrectomy with no significant differences in QoL between techniques.

*Table 2: Comparison of quality of life for different operative techniques of an oncological Ivor Lewis esophagectomy.*

| | RAMIE | Total MIS | Hybrid | Open | |
|---|---|---|---|---|---|
| | **Mean S score** | **Mean S score** | **Mean S score** | **Mean S score** | **P value*** |
| Overall score | 42.449 | 42.671 | 43.265 | 42.066 | 0.3305 |
| Global Health Status /QoL | 68.077 | 65.909 | 68.369 | 65.476 | 0.9358 |
| **Functional scales** | | | | | |
| Physical functioning | 79.385 | 79.638 | 74.235 | 72.857 | 0.1961 |
| Role functioning | 64.871 | 73.188 | 69.588 | 70.238 | 0.3781 |
| Emotional functioning | 62.949 | 69.697 | 72.047 | 63.690 | 0.0404* |
| Cognitive functioning | 72.821 | 76.515 | 80.351 | 67.857 | 0.0786 |
| Social functioning | 61.282 | 71.212 | 72.281 | 72.619 | 0.0363* |
| **Symptom Scales / Items** | | | | | |
| Fatigue | 41.138 | 39.130 | 38.039 | 39.683 | 0.4771 |
| Nausea and vomiting | 20.769 | 9.420 | 13.776 | 1.190 | 0.0647 |
| Pain | 24.872 | 21.014 | 21.212 | 27.381 | 0.4282 |
| Dyspnoea | 25.128 | 26.087 | 30.928 | 26.190 | 0.2486 |
| Insomnia | 35.385 | 36.232 | 36.082 | 28.571 | 0.9008 |
| Appetite Loss | 20.513 | 10.145 | 20.748 | 19.048 | 0.9575 |
| Constipation | 18.462 | 7.246 | 11.905 | 16.667 | 0.1068 |
| Diarrhoea | 21.538 | 30.303 | 24.211 | 40.476 | 0.5998 |
| Financial Difficulties | 18.974 | 27.272 | 14.539 | NA | 0.3239 |

** p value comparing the RAMIE and hybrid technique was calculated. A p value of <0.01 was considered statistically significant.*

**Conclusion:**
Long term assessment of quality of life with a mean follow up of 2 years revealed no significant differences between gastrectomy or transhiatal gastrectomy. Furthermore, no significant differences between overall QoL scores of different surgical approaches of the Ivor Lewis esophagectomy were seen, indicating that the surgical approach may not affect the long term QoL of patients undergoing this procedure. Qol should hence, not play a role when deciding for or against a surgical approach for these patients.

*Table 3: Comparison of s scores depicting quality of life of patients undergoing a gastrectomy compared to patients undergoing a transhiatal gastrectomy.*

| | Gastrectomy | THG | |
|---|---|---|---|
| | **Mean S score** | **Mean S score** | **P value** |
| Overall score | 42.67 | 42.116 | 0.7196 |
| Global Health Status /QoL | 61.429 | 63.333 | 0.7018 |
| **Functional Scales** | | | |
| Physical functioning | 74.074 | 71.333 | 0.5971 |
| Role functioning | 67.619 | 62.821 | 0.5332 |
| Emotional functioning | 55.629 | 62.736 | 0.1091 |
| Cognitive functioning | 76.190 | 72.5 | 0.5723 |
| Social functioning | 66.667 | 61.667 | 0.5336 |
| **Symptom Scales / Items** | | | |
| Fatigue | 42.222 | 45.139 | 0.6595 |
| Nausea and vomiting | 15.741 | 14.167 | 0.7667 |
| Pain | 30.180 | 19.583 | 0.1105 |
| Dyspnoea | 18.519 | 25.833 | 0.2752 |
| Insomnia | 41.667 | 37.5 | 0.6327 |
| Appetite Loss | 22.857 | 25.833 | 0.7074 |
| Constipation | 6.481 | 7.5 | 0.8105 |
| Diarrhoea | 40 | 38.333 | 0.8416 |
| Financial Difficulties | 20 | 24.167 | 0.5609 |

DE GRUYTER BRILL

**Impact of Surgical Approach on the Clinical Consequences of Anastomotic Leak in gastric cancer**
ID: E-DCK-196

**Viszeralchirurgie / Visceral Surgery**
**Chirurgie oberer Gastrointestinaltrakt (CAOGI) / Upper Gastrointestinal Surgery (CAOGI)**

Nerma Crnovrsanin[1], Marcel Schneider[2], Jeesun Kim[3], Felix Berlth[4], Yutaka Sugita[5], Takeshi Sano[5], Riccardo Rosati[6], Peter Grimminger[4], Gian Baiocchi[7], Maria Bencivenga[8], Giovanni De Manzoni[8], Souya Nunobe[5], Han-Kwang Yang[3], Christian Gutschow[2], Beat Müller-Stich[9], Henrik Nienhueser[1]

[1]Department for General-, Visceral- and Abdominal Surgery, Universitätsklinikum Heidelberg, Heidelberg, Deutschland, [2]Department of Surgery & Transplantation, University Hospital Zurich, Zurich, Switzerland, Zürich, Schweiz, [3]Department of Surgery, Seoul National University Cancer Hospital, Seoul, South Korea, Seoul, Südkorea, [4]Department of General, Visceral and Transplant Surgery, University Medical Centre Mainz, Mainz, Germany, Mainz, Deutschland, [5]Department of Gastroenterological Surgery, Cancer Institute Hospital of the Japanese Foundation for Cancer Research, Tokyo, Japan, Tokyo, Japan, [6]San Raffaele Hospital, Milan, Italy, Milan, Italien, [7]Department of Surgery, University Hospital of Brescia, Brescia, Italy, Brescia, Italien, [8]Department of Surgery, University Hospital of Verona, Verona, Italy, Verona, Italien, [9]Department of Surgery, Clarunis University Center for Gastrointestinal and Liver Disease, University Hospital and St. Clare Hospital Basel, Basel, Switzerland., Basel, Schweiz

**Background:**
Anastomotic leak (AL) remains the most severe complication after gastrectomy, contributing substantially to postoperative morbidity and mortality. Evidence from other surgical fields suggests that the surgical approach may modify the clinical impact of complications. This study aimed to assess whether the consequences of AL differ between minimally invasive gastrectomy (MIG) and open gastrectomy (OG).

**Material and methods:**
This retrospective study used the GASTRODATA registry, an international, multicenter database of patients undergoing curative-intent surgery for gastric cancer. Patients were excluded if they had ASA grade V, no lymphadenectomy, underwent HIPEC, additional resections beyond appendectomy or cholecystectomy, surgical procedures other than subtotal or total gastrectomy, or R2 resections. Differences between groups were assessed using chi-square tests. Propensity score matching (PSM) was applied to adjust for baseline imbalances in age, gender, T stage, N stage, and type of surgery.

**Results:**
Of 9,662 patients, 8,012 met inclusion criteria; 324 (4.1%) developed an AL. Among them, 152 (46.2%) underwent MIG and 172 (53.1%) OG. Prior to matching, patients in the OG group were older ($p < 0.001$), more often female ($p = 0.048$), presented with more advanced disease ($p < 0.001$), and more frequently received preoperative treatment ($p < 0.001$). AL after OG tended to be more severe (Clavien–Dindo > IIIb: 49% vs 38%, $p = 0.060$). Pulmonary complications were more common after OG (25% vs 14%, $p = 0.012$), whereas stenosis was more frequent after MIG (4.6% vs 0%, $p = 0.005$). After PSM, 89 matched pairs were analyzed. Pulmonary complications remained more frequent after OG (28% vs 15%, $p = 0.028$).

**Conclusion:**

In gastric cancer surgeries, anastomotic leaks following open surgery were associated with more pulmonary complications than after minimally invasive surgery, underscoring that surgical access shapes the clinical impact of this complication. Recognizing that the consequences of anastomotic leak differ by surgical approach may help tailor perioperative monitoring and complication management strategies after gastrectomy

DE GRUYTER BRILL

**Gastric Stump Cancer: Separate tumor entity or subtype of gastric cancer?**
ID: E-DCK-228

**Viszeralchirurgie / Visceral Surgery**
**Chirurgie oberer Gastrointestinaltrakt (CAOGI) / Upper Gastrointestinal Surgery (CAOGI)**

Congde Martin Xu[1], Helmut Friess[1], Carsten Jäger[1], Ekin Demir[1], Julius Shakhtour[1]

[1]TUM Universitätsklinikum München, Klinik für Allgemein und Visceralchirurgie, München, Deutschland

**Background:**
Gastric stump cancer (GSC) is a rare carcinoma that occurs 5 or more years after distal gastrectomy for benign disease. Its clinicopathological features and prognosis compared with primary gastric cancer (PGC) remain unclear.

**Material and methods:**
We retrospectively analyzed 95 patients with histologically confirmed GSC treated between 1982 and 2018 at TUM University Hospital, Munich. A matched cohort of 475 PGC patients (1:5 ratio) was generated based on age, sex, surgical date, TNM classification, R-status, and tumor grading. All underwent oncological resection with lymphadenectomy. Postoperative outcomes, pathology, and survival were assessed using Kaplan-Meier and Cox regression analyses. Immunohistochemistry was performed in a subset (GSC n=5, PGC n=10).

**Results:**
The mean age of GSC patients was 70 years, with a predominance of men (87%). The median interval between gastrectomy and GSC diagnosis was 32 years. Tumors were most often located in the upper third of the remnant stomach (42%). R0 resection was achieved in 74% of cases. Compared with PGC, there were no significant differences in postoperative complications, TNM stage, tumor grading, or R0 rates. Median overall survival in GSC patients was 58.4 months, with a 5-year survival of 36%. Survival did not differ significantly between GSC and PGC ($p=0.47$). Within the GSC cohort, tumor location was prognostically relevant: patients with tumors involving the complete remnant stomach had the poorest outcomes ($p<0.05$). Multivariable Cox regression identified tumor stage, lymph node and distant metastases, R0 status, and age as independent prognostic factors, but not tumor entity. Immunohistochemistry revealed predominance of tubular adenocarcinoma in both groups, with PDL1 expression (40% vs. 20%), mismatch repair deficiency (20% vs. 30%), and Claudin 18.2 expression (40% vs. 50%) observed in GSC vs. PGC.

**Conclusion:**
GSC shows comparable clinicopathological features and survival outcomes to PGC. Prognosis is determined by stage, resection status, and metastasis rather than tumor entity. Tumor location within the remnant stomach influences outcome, and selected immunohistochemical markers may represent therapeutic targets.

DE GRUYTER BRILL

**Adjuvant FLOT in minor responders of gastro-esophageal junction type I and II patients**
ID: E-DCK-247

**Viszeralchirurgie / Visceral Surgery**
**Chirurgie oberer Gastrointestinaltrakt (CAOGI) / Upper Gastrointestinal Surgery (CAOGI)**

Naita Wirsik[1], Max Krämer[2], Sabine Schiefer[3], Noel E. Donlon[4], Jara Tigges[5], Cezanne D. Kooij[6], Henrik Nienhüser[3], Matthew G. Davey[4], Hans Fuchs[1], Daniel Reim[5], Florian Seyfried[7], Christoph-Thomas Germer[7], Richard Van Hillegersberg[6], John V. Reynolds[4], Thomas Schmidt[1], Christiane J. Bruns[1]

[1]University Hospital of Cologne, Department of General, Visceral, Thoracic and Transplant Surgery, Köln, Deutschland, [2]University of Cologne, Gastrointestinal Cancer Group Cologne GCGC, Department I of Internal Medicine, Center for Integrated Oncology Aachen Bonn Cologne Duesseldorf, Köln, Deutschland, [3]University Hospital of Heidelberg, Department of General, Visceral, and Transplant Surgery, Heidelberg, Deutschland, [4]Trinity College Dublin, Trinity St James' Cancer Institute, St James's Hospital Dublin, Ireland, Department of Surgery, Dublin, Ireland, [5]Technical University of Munich, Klinikum Rechts der Isar,, Department of Surgery, München, Deutschland, [6]University Medical Center Utrecht, Department of Surgery, Utrecht, Deutschland, [7]University Hospital Wuerzburg, Department of General, Visceral, Transplantation, Vascular and Pediatric Surgery, Würzburg, Deutschland

**Background:**
Since the ESOPEC study perioperative chemotherapy according to the FLOT protocol has become standard of care in the treatment of adenocarcinomas of the esophagus (EAC) and gastroesophageal junction (GEJ) due to prolonged survival in comparison to neoadjuvant radio chemotherapy with the CROSS protocol. Nonetheless, there is a high number of patients with a minor response to FLOT and the SPACE FLOT study showed a potential benefit for minor responder undergoing FLOT adjuvantly, while including gastric cancer and GEJ tumors. However, GEJ Siewert type I/II show different biological characteristics than gastric cancer. Therefore, a multi-national high-volume center cohort study for minor responder evaluating the role of adjuvant FLOT treatment in GEJ tumors was undertaken.

**Material and methods:**
Retrospective analysis of prospectively maintained databases from six high-volume centers with patients fulfilling inclusion criteria of AC or GEJ tumor Siewert type I/II and minor response to neoadjuvant treatment with at least 3 cycles FLOT undergoing a surgical resection in a curative intend. Primary endpoint was overall survival (OS). Subgroup analyses were performed to account for selection bias.

**Results:**
Of the cohort, 70.5% (n=285) received an adjuvant treatment, while 29.5% (n=119) received only follow-up care. Only 59.3% of patients undergoing an adjuvant treatment received at least 3 cycles of FLOT without any dosage reduction. Regarding preoperative patient characteristics of both groups (± adjuvant therapy), there were no differences regarding gender, age, ASA classification or clinical TNM stage ($p>0.05$). The majority of both groups underwent esophagectomy ($p=0.48$). Postoperative morbidity was higher for patients undergoing follow-up care than adjuvant treatment ($p<0.001$), while there were no significant distinctions regarding resection rate ($p=0.07$). Histopathological outcomes were similar in

Abstracts – DCK 2026 – 143rd Congress of the German Society of Surgery – Leipzig, April 22–24 • DOI 10.1515/iss-2026-2002
Innov Surg Sci 2026; 11 (Special Suppl 1): s77–s269

regard to pT and pM-category (p>0.05) between both groups. Solely, the pN category was higher for patients receiving follow-up care (p=0.01), while all patients were minor or non-responders.
OS was longer in the adjuvant treated group with a median survival of 44.8 months (95% CI 37.6-51.9) versus 33.0 months (95% CI 21.1-44.9, p=0.003; Figure 1A). Adjuvant treatment showed also a prolonged disease-free survival (DFS) in contrast to follow-up care (p=0.22, Figure 1B).
After exclusion of patients with severe postoperative complication (Clavien-Dindo ≥3b) the survival benefit for adjuvant treatment of minor responder in comparison to the ones undergoing follow-up care was no longer significantly different (p=0.12, Figure 1C) as well as DFS (p=0.62, Figure 1D).

**Conclusion:**
It may be more beneficial for minor responders to undergo a strategy of follow-up care and treatment initiation on tumor progression instead of standardized FLOT adjuvantly as the benefit of adjuvant treatment on patient survival did not remain after exclusion of severe postoperative morbidity.

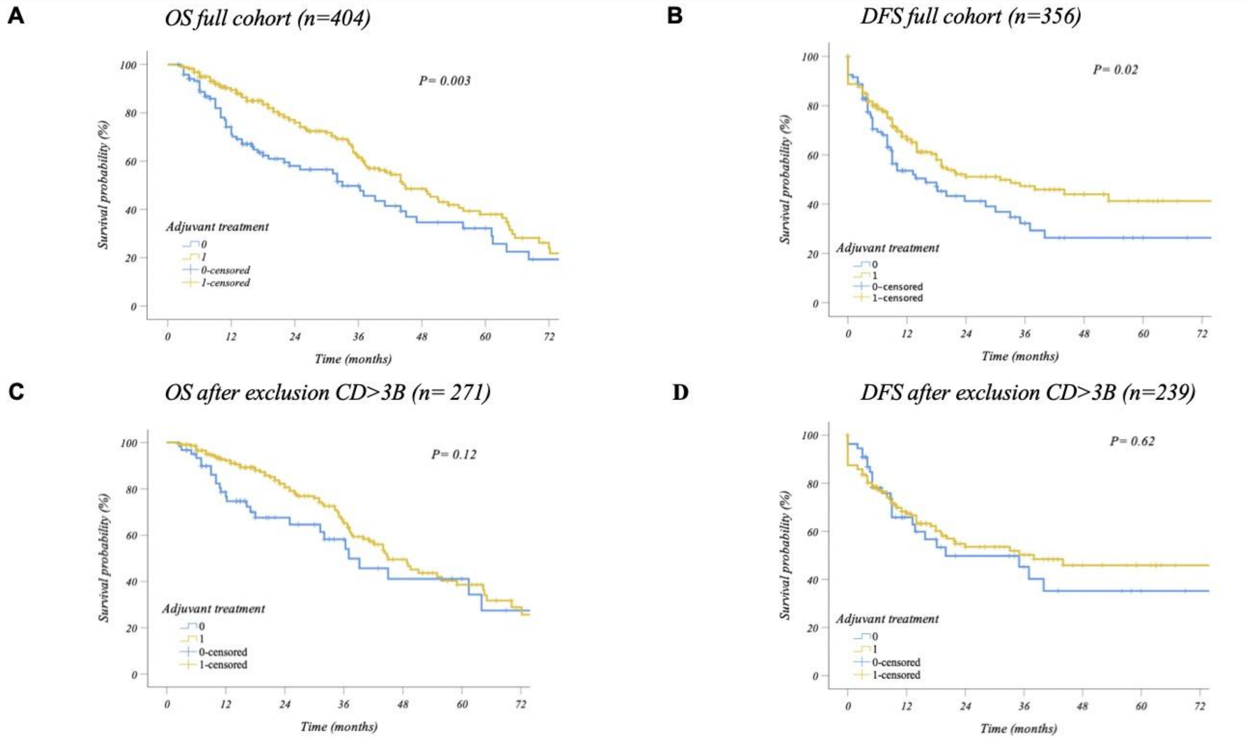

DE GRUYTER BRILL

**Activation of tumor-draining lymph nodes in EGA resistant to chemo- or radiotherapy**
ID: E-DCK-384

**Viszeralchirurgie / Visceral Surgery**
**Chirurgie oberer Gastrointestinaltrakt (CAOGI) / Upper Gastrointestinal Surgery (CAOGI)**

Loreen Sophie Rudek[1 2], Martin Thelen[1 2], Su Ir Lyu[3], Kerstin Wennhold[1 2], Alexander Quaas[3], Christiane Bruns[2], Axel Hillmer[3], Maria Garcia-Marquez[1 2], Hans Schlößer[1 2]

[1]Center for Molecular Medicine Cologne, University of Cologne, Faculty of Medicine and University Hospital Cologne, Cologne, Deutschland, [2]Department of General, Visceral, Thoracic, and Transplantation Surgery, University of Cologne, Faculty of Medicine and University Hospital Cologne, Cologne, Deutschland, [3]Institute of Pathology, University of Cologne, Faculty of Medicine and University Hospital Cologne, Cologne, Deutschland

**Background:**
Most patients with esophago-gastric adenocarcinoma (EGA) present with locally advanced or metastatic disease at first diagnosis due to the late onset of tumor-related symptoms such as dysphagia. Multimodality therapies including surgery and neoadjuvant radio-chemotherapy (CROSS) or perioperative chemotherapy (FLOT) are the standard of care with recent data demonstrating superiority of FLOT. Despite the positive results of neoadjuvant multimodality protocols, a large fraction of EGA patients show primary resistance with minor pathological response. Addition of anti-PD-(L)1 treatments to the multimodality protocols of EGAs appears promising and is already approved in specific situations.
The CheckMate-577 trial revealed a significant increase in progression-free survival (PFS) of patients with residual tumor after neoadjuvant radio-chemotherapy and lead to approval of Nivolumab (anti-PD-1) as adjuvant treatment following CROSS in EGAs. In line with this, the combination of chemotherapy and Nivolumab prolonged disease-free survival (DFS) and overall survival (OS) in previously untreated advanced or metastatic EGAs. The latest MATTERHORN trial (perioperative FLOT plus Durvalumab (anti-PD-1)) also demonstrated better event-free survival outcomes than chemotherapy alone. In contrast, adjuvant immunotherapy with Nivolumab plus Ipilimumab (CTLA-4 inhibitor) in patients with poor response to neoadjuvant FLOT was inferior to postoperative continuation of FLOT in the VESTIGE trial. Hence, resistance to immunotherapy represents a major obstacle in treatment of EGAs. Mechanisms of resistance and factors determining susceptibility to immunotherapy remain poorly understood.

**Material and methods:**
We aim to elucidate the immune microenvironment of FLOT- and CROSS-resistant patients. Tumor-draining lymph nodes (TDLN) are a functional part of the extended tumor microenvironment. We hypothesize that lymph node activation is a surrogate for tumor immunogenicity and susceptibility to immune checkpoint inhibition. We selected 135 therapy-resistant patients who underwent surgery for EGA in a multimodal setting at the University Hospital Cologne from 2017-2023. Histopathological evidence of residual vital tumor cells in the resected tumor samples was considered a failure of neoadjuvant therapy. The activation status of non-metastatic TDLNs (lymph node station 7, 8, 9, and lymph nodes from the lesser curvature) was defined based on presence of secondary follicles and sinus histiocytosis visible on Hematoxylin and Eosin (H&E) staining of formalin-fixed, paraffin-embedded (FFPE) tumor slides.

**Results:**
When comparing multiple lymph nodes from the same patients from different lymph node stations, a complete consistency of lymph node activation was observed in 80% of FLOT- and 67% of CROSS-treated patients, respectively. 113 samples from patients with >50% residual tumor cells following CROSS (n=29) or FLOT (n=84) were included in this study. Patients treated with FLOT had significantly more activated lymph nodes than patients treated with CROSS. 93% of FLOT-treated patients had activated lymph nodes in comparison to only 55% of CROSS-treated patients ($p<0.01$). The activation in TDLNs of CROSS-resistant patients was even lower in patients with <50% residual tumor cells. Analyses of samples from CROSS-resistant patients receiving adjuvant Nivolumab showed no correlation of lymph node activation and responsiveness to Nivolumab.

**Conclusion:**
In contrast to a proposed synergism of radiotherapy and immune checkpoint inhibition, activation of TDLNs appears to be higher in patients treated with chemotherapy. Deeper insights into the immune composition of resistant tumors will provide important rationales for tailored treatment strategies and hopefully superior outcomes in this very challenging patient cohort. Further investigations of the immune microenvironment (e.g., $CD3^+$ T-cell infiltration, presence of tertiary lymphoid structures) of the selected patients are ongoing and will be included in the presentation.

DE GRUYTER BRILL

**Esophageal Reconstruction After Caustic Injury: A Case Report with Postoperative Stenosis**
ID: E-DCK-498

**Viszeralchirurgie / Visceral Surgery**
**Chirurgie oberer Gastrointestinaltrakt (CAOGI) / Upper Gastrointestinal Surgery (CAOGI)**

Henrik Nienhüser[1], Leonard Raphael Klevesath[1], Marcus Kantowski[1]

[1]Uniklinikum Heidelberg, Abteilung für Viszeral- und Transplantationschirurgie, Heidelberg, Deutschland

**Background:**
Reversal of esophageal discontinuity is a complex and challenging procedure. Interposition of the colon can offer a potential reconstruction technique and is commonly used in patients with resected stomach. However, it comes with challenges, preventing anastomotic leaks, and managing long-term complications like reflux or stenosis. EGD (esophagogastroduodenoscopy) balloon dilation and stent placement are well-documented methods for treating esophageal stenosis in the literature. In this case, however, these established techniques are applied in a unique situation following a colon interposition reconstruction.

**Material and methods:**
The data presented in this case report were collected through a detailed patient history (anamnesis) and retrospectively reviewed from the patient's clinical records. All relevant clinical, surgical, and postoperative information was systematically extracted and analyzed to provide a comprehensive overview of the case.

**Results:**
A 21-year-old female patient who underwent esophagectomy and gastrectomy following caustic ingestion five years ago presented to our department with the strong wish for reversal of the discontinuity. We performed a reconstruction using the ascending colon and the terminal ileum for reconstruction. A challenging aspect was the complete stricture of the cervical esophagus requiring and anastomosis of the ileum to the pharyngeal cartilage.

Recanalisation was done by intraoperative rendez-vous endoscopy and external preparation of the ileum stump and residual pharynx. -Postoperatively, the patient developed dysphagia due to stenosis at the cervical anastomosis. This was managed through multiple balloon dilations via EGD and the placement of a partially coated biliary stent. Since then, the patient has been able to swallow fluids and soft foods (porridge food breikost) without significant difficulty

**Conclusion:**
Reversal of esophageal discontinuity is a complex and challenging procedure. Interposition of the colon can offer a potential reconstruction technique and is commonly used in patients with resected stomach. However, it comes with challenges, preventing anastomotic leaks, and managing long-term complications like reflux or stenosis. EGD (esophagogastroduodenoscopy) balloon dilation and stent placement are well-documented methods for treating esophageal stenosis in the literature. In this case, however, these established techniques are applied in a unique situation following a colon interposition reconstruction.

DE GRUYTER BRILL

**Impact of Obesity and Metabolic Disorders on Surgical Outcomes in Esophageal Cancer**
ID: E-DCK-94

**Viszeralchirurgie / Visceral Surgery**
**Chirurgie oberer Gastrointestinaltrakt (CAOGI) / Upper Gastrointestinal Surgery (CAOGI)**

Christian Schineis[1], Nataliya Vavrynchuk[1], Wael Rayya[1], Ioannis Pozios[1], Swantje Malinka[1], Vanessa Pross[2], Pia Jäger[1 2 3]

[1]Charité Berlin Campus Benjami Franklin, Klinik für Allgemein- und Viszeralchirurgie, Berlin, Deutschland, [2]Charité Berlin Campus Benjamin Franklin, Allgemein- und Viszeralchirurgie - Chirurgisches Forschungslabor, Berlin, Deutschland, [3]Berlin Institute of Health at Charité – Universitätsmedizin Berlin, Berlin, Deutschland

**Background:**
Esophageal cancer is the eighth most common cancer worldwide, classified mainly into squamous cell carcinoma (SCC) and adenocarcinoma (AC) with distinct risk factors. SCC is mainly linked to smoking and alcohol, while AC is driven by GERD, Barrett’s esophagus and obesity. In industrialised countries, AC accounts for about two-thirds of cases, driven by rising obesity and GERD prevalence. Surgical treatment includes robotic, laparoscopic and the increasingly rare conventional approach. The most frequent procedure is the Ivor Lewis procedure. Depending on tumour localisation and resection extent, surgery is challenging, and obesity can further hinder accessibility and resectability. In oncological laparoscopy, such as colorectal surgery, obesity is a well-known risk factor for limited feasibility and reduced success. However, the current state of the literature on esophageal cancer is inconsistent regarding the influence of obesity on surgical, peri-surgical (short-term) and long-term outcome. To the best of the authors' knowledge, no robust data on surgical outcomes are currently available.
This study aims to investigate the effect of body mass index (BMI), obesity and diabetes, as well as their combination, on the surgical outcomes of esophageal cancer patients in a robust database of a large, nationwide patient population, using an extended, adjusted analysis. This includes the primary outcome of R0 resection, as well as the length of hospital and ICU stay, and surgical complications.
Esophageal cancer is the eighth most common cancer worldwide, classified mainly into squamous cell carcinoma (SCC) and adenocarcinoma (AC) with distinct risk factors. SCC is mainly linked to smoking and alcohol, while AC is mainly driven by GERD, Barrett’s esophagus and obesity. In industrialised countries, AC now accounts for about two-thirds of cases, driven by rising obesity and GERD prevalence. Surgical treatment includes robotic and laparoscopic and the increasingly rare conventional approach. The most frequent procedure is the Ivor Lewis procedure. Depending on tumour localisation and resection extent, surgery is challenging, and obesity can further hinder accessibility and resectability. In oncological laparoscopy, such as colorectal surgery, obesity is a well-known risk factor for limited feasibility and reduced success.
However, the current state of the literature on esophageal cancer is inconsistent regarding the influence of obesity on surgical, peri-surgical (short-term) and long-term outcome. To the best of the authors' knowledge, no robust data on direct surgical outcomes are currently available. As obesity is increasing almost everywhere in the world, the impact is expected to grow in the future, due to both the increased prevalence and the therapeutic approach to treating affected patients.
This study aims to investigate the effect of body mass index (BMI), obesity and diabetes, as well as their combination, on the surgical outcomes of esophageal cancer patients in a robust database of a large, nationwide patient population, using an extended, adjusted analysis. This includes the primary outcome of R0 resection, as well as the length of hospital and ICU stay, and surgical complications.

**Material and methods:**
The German nationwide StuDoQ registry of the DGAV is used as the database. All patients undergoing surgery for esophageal cancer between 2021 and 2024 are included. Descriptive statistics are performed first, followed by extended multivariate regression analyses (generalised for the dichotomous outcome parameter of R0 resection and linear/ordinally scaled for the secondary outcome parameters dependent on the properties of the variables) adjusted for various oncological, surgical and patient-specific conditions.
The German nationwide StuDoQ registry of the DGAV is used as the database. All patients undergoing surgery for esophageal cancer between 2021 and 2024 are included. Descriptive statistics are performed first, followed by extended multivariate regression analyses (generalised for the dichotomous outcome parameter of R0 resection and linear/ordinally scaled for the secondary outcome parameters dependent on the properties of the variables) adjusted for various oncological, surgical and patient-specific conditions.

**Results:**
Overall, 1,155 patients undergoing surgery for esophageal cancer are included, with R0 resection achieved in 95.5% (n = 1,103). The mean BMI is significantly higher in patients with R0 compared to R1 resection (26.13 ± 4.95 vs. 24.34 ± 4.36; Welch's $t$-test, $t = 2.87$, $df = 57.4$, $p = 0.006$). In multivariable logistic regression adjusted for *histology, regression score, operative technique, comorbidity, operative approach, anastomotic localisation, ASA score, ECOG status, age,* and *sex*, higher BMI is associated with a lower likelihood of R1 resection (OR = 0.89, 95% CI 0.81–0.99, $p = 0.017$; McFadden's $R^2 = 0.26$). These findings persist after additional adjustment for diabetes (BMI effect: OR = 0.89, 95% CI 0.80–0.96, $p = 0.006$; McFadden's $R^2 = 0.27$).
Patients undergoing surgery for esophageal cancer (n = 1,155) have a mean hospital stay of 22.42 days (±27.40; range 3–433) and a mean ICU stay of 7.83 days (±11.89; range 0–132). No significant correlation between BMI and either hospital stay or ICU stay is observed in unadjusted or adjusted analyses.
In esophageal cancer patients, BMI shows no association with complications, neither in group comparisons (Clavien–Dindo, $p = 0.26$) nor in adjusted ordinal regression ($p = 0.58$).
Overall, 1,155 patients undergoing surgery for esophageal cancer are included, with R0 resection achieved in 95.5% (n = 1,103). The mean BMI is significantly higher in patients with R0 compared to R1 resection (26.13 ± 4.95 vs. 24.34 ± 4.36; Welch's $t$-test, $t = 2.87$, $df = 57.4$, $p = 0.006$). In multivariable logistic regression adjusted for *histology, regression score, operative technique, comorbidity, operative approach, anastomotic localisation, ASA score, ECOG status, age,* and *sex*, higher BMI is associated with a lower likelihood of R1 resection (OR = 0.89, 95% CI 0.81–0.99, $p = 0.017$; McFadden's $R^2 = 0.26$). These findings persist after additional adjustment for diabetes (BMI effect: OR = 0.89, 95% CI 0.80–0.96, $p = 0.006$; McFadden's $R^2 = 0.27$).
Patients undergoing surgery for esophageal cancer (n = 1,155) have a mean hospital stay of 22.42 days (±27.40; range 3–433) and a mean ICU stay of 7.83 days (±11.89; range 0–132). No significant correlation between BMI and either hospital stay or ICU stay is observed in unadjusted or adjusted analyses.
In esophageal cancer patients, BMI shows no association with complications, neither in group comparisons (Clavien–Dindo, $p = 0.26$) nor in adjusted ordinal regression ($p = 0.58$).

**Conclusion:**

Surgical and peri-surgical outcomes of esophageal cancer are surprisingly favourable (even) in obese patients. BMI and diabetes do not appear to impair surgical success as might be expected. These findings should be considered in clinical decision-making. Potential underlying reasons, such as differences in tumour biology, should be addressed in further research.

Surgical and peri-surgical outcomes of esophageal cancer are surprisingly favourable (even) in obese patients. BMI and diabetes do not appear to impair surgical success as might be expected. These findings should be considered in clinical decision-making. Potential underlying reasons, such as differences in tumour biology, should be addressed in further research.

DE GRUYTER BRILL

**Iterative Hepatic and Pulmonary Metastasectomy in Stage IV Colorectal Cancer**
ID: E-DCK-151

**Viszeralchirurgie / Visceral Surgery**
**Chirurgie unterer Gastrointestinaltrakt, Koloproktologie (CACP) / Lower Gastrointestinal Surgery and Coloproctology (CACP)**

Luisa Schäfer[1], Philipp Holzner[1], Magdalena Menzel[1], Gabriel Stöger[1], Andreas Gengenbach[1], Christian Hillebrecht[1], Francesca Reimer[1], Rebecca Kesselring[1], Uyen-Thao Le[2], Stefan Fichtner-Feigl[1], Christopher Berlin[1]

[1]Uniklinik Freiburg, Klinik für Allgemein- und Viszeralchirurgie, Freiburg, Deutschland, [2]Uniklinik Freiburg, Klinik für Thoraxchirurgie, Freiburg, Deutschland

**Background:**
Colorectal carcinoma frequently metastasizes to the liver and lungs. Despite curatively intended resections, recurrence rates remain high. While outcomes after initial hepatic and pulmonary metastasectomies are well studied, data on repeated resections for recurrent metastases in both organs are limited.

**Material and methods:**
We conducted a retrospective analysis of 97 patients who underwent iterative hepatic and pulmonary resections for recurrent colorectal metastases. Long-term outcomes with a follow-up exceeding 10 years were assessed.

**Results:**
Survival after initial hepatic metastasectomy did not differ between synchronous and metachronous liver metastases. Multiple hepatic and pulmonary metastasectomies resulted in survival comparable to that of patients undergoing single resections. Postoperative complications of Clavien-Dindo grade ≥ II following a second liver resection were associated with reduced overall survival, whereas pulmonary resections had no impact on survival. Iterative liver and lung resections did not increase postoperative morbidity, with median complication severity remaining within Clavien-Dindo grade II–IIIa across resections.

**Conclusion:**
Hepatic metastasectomy provides comparable benefit regardless of the sequence of metastasis. Iterative hepatic and pulmonary resections for recurrent colorectal metastases appear safe and may contribute to prolonged overall survival. These findings support an aggressive surgical approach in carefully selected patients with recurrent metastases.

DE GRUYTER BRILL

**Multimodal prehabilitation in colorectal cancer – the randomized controlled PREHAB-PEER trial**
ID: E-DCK-157

**Viszeralchirurgie / Visceral Surgery**
**Chirurgie unterer Gastrointestinaltrakt, Koloproktologie (CACP) / Lower Gastrointestinal Surgery and Coloproctology (CACP)**

Magdalena Holze[1], Solveig Tenckhoff[2], Eva Kalkum[2], Jens Notheis[2], Maximilian Joos[2], Manuel Feisst[3], Alexandra Balzer[3], Joachim Wiskemann[4], Till Johannes Bugaj[5], Ingeborg Rötzer[6], Erich Grohmann[7], Rosa Klotz[1]

[1]Universitätsklinikum Heidelberg, Klinik für Allgemein-, Viszeral- und Transplantationschirurgie, Heidelberg, Deutschland, [2]Universitätsklinikum Heidelberg, Studienzentrum der Deutschen Gesellschaft für Chirurgie, Heidelberg, Deutschland, [3]Universitätsklinikum Heidelberg, Institut für medizinische Biometrie, Heidelberg, Deutschland, [4]Universitätsklinikum Heidelberg, Klinisch für Medizinische Onkologie, Nationales Centrum für Tumorerkrankungen, Heidelberg, Deutschland, [5]Universitätsklinikum Heidelberg, Klinik für Innere Medizin und Psychosomatik, Heidelberg, Deutschland, [6]Universitätsklinikum, Klinik für Innere Medizin I, Heidelberg, Deutschland, [7]Deutsche ILCO e.V., Heidelberg, Deutschland

**Background:**
Multimodal prehabilitation aims to improve physical fitness, nutritional status, and psychological resilience before major surgery, including colorectal cancer surgrey. It has the potential to reduce postoperative complications, empower patients through active participation in their care, and support faster reintegration into social life and recovery to pre-surgery health status. While recent studies have strengthened the evidence for prehabilitation, existing randomized controlled trials (RCTs) show relevant methodological limitations and inconclusive results. The PREHAB-PEER trial will evaluate the effect of a personalized multimodal prehabilitation intervention on the postoperative quality of recovery in high-risk CRC patients.

**Material and methods:**
The multicenter randomized controlled PREHAB-PEER trial was developed during a 12-month conceptual phase in close collaboration with all relevant stakeholders to ensure feasibility and clinical relevance of the planned trial. Patient and scientific target groups were systematically identified, and tailored methods of stakeholder involvement were applied in four consecutive work packages (Table 1) including a patient survey, an expert workshop, and focus group meetings. Stakeholders actively shaped the trial design, including eligibility criteria, intervention components, implementation strategies, and outcome parameters. In addition, potential ethical, social, and psychosocial challenges were proactively assessed, and corresponding solution strategies were developed to optimize patient compliance and trial conduct.

Abstracts – DCK 2026 – 143rd Congress of the German Society of Surgery – Leipzig, April 22–24 • DOI 10.1515/iss-2026-2002
Innov Surg Sci 2026; 11 (Special Suppl 1): s77–s269

| Month\| Work package (WP) | 1 | 2 | 3 | 4 | 5 | 6 | 7 | 8 | 9 |
|---|---|---|---|---|---|---|---|---|---|
| WP-1: Identification of target groups | ■ | ■ | | | | | | | |
| WP-1.1: Meetings with focus groups and stakeholders | ■ | ■ | | | | | | | |
| WP-2: Methods for patient involvement | | ■ | ■ | ■ | ■ | ■ | | | |
| WP-3: Development of trial concept | | | | ■ | ■ | ■ | ■ | ■ | |
| WP-3.1: Patient and stakeholder survey | | | | ■ | ■ | | | | |
| WP-3.2: Analysis of Survey | | | | | | ■ | | | |
| WP-3.3: Statistical planning and design of the trial | | | | | | | ■ | ■ | |
| WP-3.4: Type and implementation of trial intervention | | | | | | | ■ | ■ | |
| WP-3.5: Identification of sociopsychological barriers and risks for non-compliance of patients/development of potential solutions | | | | | ■ | ■ | ■ | ■ | |
| WP-4: Conceptualization of the target group involvement for the implementation phase | | | | | | | ■ | ■ | ■ |
| WP-4.1: Identification of trial documents needed in lay language (e.g., flyer, patient information etc.) | | | | | | | ■ | ■ | ■ |

**Table 1.** Work packages of the PREHAB-PEER conceptual phase

**Results:**
From November 2023 to October 2024, the PREHAB-PEER conceptual phase defined the design of a multicenter RCT in high-risk CRC patients prior to curative surgery. Key stakeholders included the Patient Advisory Board of the Study Center of the German Society of Surgery, the Deutsche ILCO e.V., CRC patients, and experts in nutrition, physiotherapy, psychosomatics, and prehabilitation. It was chosen to include a high-risk population as elderly, frail, and malnourished CRC patients are particularly vulnerable to the adverse effects of surgical stress, and this patient population benefits most from supportive preoperative measures. The prehabilitation program consists of four components: (1) personalized strength and endurance exercise via supervised live-remote sessions, (2) individual nutritional counselling providing each patient with a personalised nutrition plan, including high-calorie and protein supplements, plus Dietitian weekly follow-up calls, (3) psychooncological care with group sessions for all patients including relaxation and meditation techniques and individual face-to-face sessions for those with higher need, and (4) structured peer support through the Deutsche ILCO e.V. The outpatient, setting with close supervision ensures flexibility and patient adherence. Quality of Recovery (QoR) was defined as the primary endpoint as it was rated most relevant for by the target patient population and directly reflects patient experience compared to functional outcomes. The primary endpoint will be assessed on postoperative day 4, secondary outcomes include surgical, functional and patient-reported outcomes with a 60-day postoperative follow-up.
Overall, 196 patients will be included in each study arm randomized either to a multimodal prehabilitation intervention (3-4 weeks if scheduled for upfront resection or up to 12 weeks prehabilitation if neoadjuvant treatment is indicated) or standard preoperative care. Patients will be recruited over a 36-months enrolment period in over 10 trial sites across Germany.

**Conclusion:**
Multimodal prehabilitation has the potential to improve the preoperative condition of high-risk CRC patients, reduce postoperative complications, and lower mortality, yet no standardized program or setting for this approach currently exists. The PREHAB-PEER trial addresses this evidence gap with a patient-centered, stakeholder-driven approach. Our conceptual phase demonstrated that active patient involvement is both feasible and highly effective, laying the foundation for a rigorous multicenter RCT design to generate high-quality evidence on prehabilitation in CRC care.

DE GRUYTER BRILL

### Clinical Obesity and Metabolic Risk in Colorectal Cancer: A Prospective Cohort Analysis
ID: E-DCK-191

**Viszeralchirurgie / Visceral Surgery**
**Chirurgie unterer Gastrointestinaltrakt, Koloproktologie (CACP) / Lower Gastrointestinal Surgery and Coloproctology (CACP)**

Lars Schmitt[1], Cornelius Struck[1], Jacques Hilbert[1], Hannes Neeff[1], Stefan Fichtner-Feigl[1], Gabriel Seifert[1]

[1]Uniklinik Freiburg, Klinik für Allgemein-/Viszeralchirurgie, Freiburg, Deutschland

**Background:**
Obesity and metabolic comorbidities drive the incidence of colorectal cancer (CRC) and associate with advanced tumor stages, more complications and worse survival. Yet, previous studies are largely retrospective and rely on automated retrieval of BMI values. This may dramatically underestimate the true prevalence of obesity and metabolic disorders in CRC patients. The recently proposed definition of *clinical obesity* (Lancet 2025) departs from the BMI and may better capture the metabolic disease burden in this population. To describe the prevalence of obesity and metabolic risk factors in patients with incident CRC and to compare traditional BMI-based classification with the new definition of clinical obesity.

**Material and methods:**
We conducted a prospective cohort study at the Comprehensive Cancer Center Freiburg (CCCF) including patients with first diagnosis of CRC. Demographics, tumor stage, anthropometric measures, and metabolic comorbidities were systematically collected. Obesity was defined both by BMI (≥30 kg/m²) and according to the Lancet 2025 clinical obesity framework. Data were analyzed descriptively; the cohort database is continuously expanding with ongoing follow-up.

**Results:**
Among 85 patients (mean age 63.8 years, 32.9% female), obesity prevalence differed substantially by definition: 32.9% were classified as obese by BMI, whereas 48.2% fulfilled the new clinical obesity criteria. Patients with clinical obesity showed higher rates of type 2 diabetes (32% vs. 13%), hyperlipidemia (37% vs. 27%), arterial hypertension (68% vs. 27%), and sleep apnea (22% vs. 0%) compared to those without clinical obesity. Across UICC stages, a high burden of metabolic dysfunction and cardiovascular comorbidities was observed.

**Conclusion:**
In this prospective CRC cohort, the new definition of clinical obesity identified a substantially larger group of patients at metabolic risk compared to the traditional WHO classification. The strong overlap between CRC and metabolic comorbidities underscores the need for integrated oncologic–metabolic management strategies. Ongoing analyses will further clarify the prognostic impact of these conditions.

DE GRUYTER BRILL

### Discordance between restaging MRI and final pathology following neoadjuvant therapy in rectal cancer

ID: E-DCK-255

**Viszeralchirurgie / Visceral Surgery**
**Chirurgie unterer Gastrointestinaltrakt, Koloproktologie (CACP) / Lower Gastrointestinal Surgery and Coloproctology (CACP)**

Michaela Ramser[1], Simon Dindo[2], Sandro Hügli[1], Ralph Fritsch[3], Matthias Turina[1]

[1]Universitätsspital Zürich, Departement für Viszeral- und Transplantationschirurgie, Zürich, Schweiz, [2]Universität Zürich, Zürich, Schweiz, [3]Universitätsspital Zürich, Klinik für Medizinische Onkologie und Hämatologie, Zürich, Schweiz

**Background:**
Accurate restaging after neoadjuvant therapy (NAT) is crucial in the management of rectal cancer, especially in centers that aim to implement organ-preserving watch-and-wait (W&W) strategies for patients achieving complete response. Reliable identification of clinical complete response (cCR) is key to safely avoiding radical surgery. Magnetic resonance imaging (MRI) is the cornerstone of restaging after NAT in patients with rectal cancer, but its diagnostic accuracy in predicting complete remission following NAT remains debated, with both overstaging and understaging reported. Such discrepancies can lead to unnecessary resections in true complete responders or to potentially detrimental undertreatment in patients with residual disease. Moreover, the accuracy of MRI restaging strongly depends on the expertise and specialization of the interpreting radiologist, which adds further variability. This study evaluated the concordance between MRI-based restaging and pathological staging in patients who underwent total mesorectal excision following NAT at our institution, with a particular focus on MRI-defined complete clinical response (cCR).

**Material and methods:**
Retrospective analysis of patients with rectal cancer who underwent NAT (chemotherapy, radiotherapy, chemoradiotherapy or immunotherapy) followed by surgical resection between 2018 and 2024 at our institution. MRI-based tumor regression grade (mrTRG) was available in 51 patients, while MRI T-stage restaging (ymrT) was documented in 38 patients. Both were compared with pathological tumor regression grade (pTRG) and pathological T-stage (ypT) in patients undergoing resection.

**Results:**
A total of 113 patients were eligible for analysis (38.9% female). Median age was 62 years (Q1 53, Q3 74), median BMI 24.5kg/m2 (Q1 21.7, Q3 27.6). Eleven patients received chemotherapy alone, 21 radiotherapy, 47 chemoradiotherapy, 33 total neoadjuvant therapy, 1 immunotherapy (with Dostarlimab). In 51 patients preoperative mrTRG and pTRG (Dworak) was available. Overall, 16 patients were staged correctly (31.4%), while 33.3% were overstaged radiologically and 35.3% radiologically understaged. mrTRG1 was attributed to 4 patients of which 2 had a pTRG4 (Dworak, complete response), while 1 patient had pTRG3 and 1 patient pTRG2. pTRG4 was reported in overall 9 patients of with 2 were preoperatively staged mrTRG1, 5 mrTRG2, 1 mrTRG3 and 1 mrTRG4.

In 38 patients, preoperative ymrT and ypT staging was available. Correct preoperative radiological staging was achieved in 36.8% (14 patients), while 47.4% were radiologically overstaged and 15.8% radiologically understaged. ymrT0 was reported in 2 patients of which one resulted in ypT0 and the other in ypT1. ypT0 was reported in 6 patients of which only 1 patient had a preoperative ymrT0 staging. Two patients were staged ymrT2, 2 patients ymrT3 and one patient ymrT4.

**Conclusion:**

MRI restaging after neoadjuvant therapy showed limited accuracy in identifying CR in rectal cancer in a modern European academic cancer center. In fact, only one of three patients was correctly staged preoperatively. Both overstaging and understaging were frequent, resulting in clinically relevant consequences: false-positive findings of CR (ymrT0 or mrTRG1) carry the risk of hazardous undertreatment in watch-and-wait protocols, while overstaging of true complete responders subjects patients to avoidable radical surgery. These limitations highlight that, although being the cornerstone of post-treatment assessment, MRI restaging remains unreliable to predict pCR in an unacceptably high percentage of patients.

In clinical practice, these findings emphasize the need for a cautious and multimodal approach to restaging. MRI results should always be interpreted in conjunction with endoscopy and digital rectal examination. For centers offering organ-preserving strategies, continuous quality assurance of radiological staging is essential, and treatment decisions should not solely rely on MRI. Furthermore, in centers with high discordance of preoperative MRI restaging, surgical resection should, when in doubt, be offered to patients at risk of understaging.

DE GRUYTER BRILL

**Centralization of Swiss colorectal surgery - a nationwide audit of 33'000 patients across >10 years**
ID: E-DCK-258

**Viszeralchirurgie / Visceral Surgery**
**Chirurgie unterer Gastrointestinaltrakt, Koloproktologie (CACP) / Lower Gastrointestinal Surgery and Coloproctology (CACP)**

Michaela Ramser[1], Werner Vach[2], Matthias Turina[1]

[1]Universitätsspital Zürich, Departement für Viszeral- und Transplantationschirurgie, Zürich, Schweiz,
[2]Basel Academy, Basel, Schweiz

**Background:**
Colorectal cancer is among the leading causes of cancer-related morbidity and mortality worldwide, posing a major public health challenge. In Switzerland alone, 28.5 (female) - 41.7 (male)/100'000 persons per year are newly diagnosed each year, with surgical resection being the mainstay of treatment. As postoperative outcomes are not only determined by quality of the individual operation, but also heavily influenced by both institutional and surgeon case numbers, political initiatives to centralize colon and rectal surgery are currently implemented, as for rectal cancer.
Over the past decade, the complexity of colorectal cancer surgery has steadily increased. Advances such as the standardization of oncologic resections, the introduction of total mesocolic and mesorectal excision and extended lymphadenectomy, as well as the dissemination of minimally invasive and robotic techniques, have raised expectations for surgical expertise and institutional infrastructure. These developments have reinforced the argument that high-quality cancer surgery is best provided in specialized centres with adequate case volumes and interdisciplinary teams.
International experiences demonstrate that centralization of complex cancer surgery can reduce postoperative morbidity, increase the use of modern techniques such as minimal-invasive and especially robotic surgery, and ultimately improve outcomes. Consequently, many healthcare systems have introduced volume-based regulations or regionalization strategies to ensure that patients benefit from specialized expertise. In Switzerland, political initiatives over recent years have also sought to implement such measures. However, nationwide data on whether these efforts have effectively changed practice patterns by concentrating colorectal cancer surgery in high-volume hospitals and facilitating access to minimally invasive surgical techniques are still scarce.
The present study therefore aims to provide a comprehensive, population-level analysis of colorectal cancer surgery in Switzerland over a ten-year period using administrative data. By examining more than 32,000 patients and stratifying surgical procedures according to complexity and hospital volume, we investigate the extent to which centralization has occurred and assess its potential impact on access to specialized oncologic care.

**Material and methods:**
We conducted a retrospective, population-based analysis using the national database of the Swiss Federal Statistical Office (Bundesamt für Statistik; BFS). All patients treated between 2012 and 2022 with a diagnosis of colorectal cancer (ICD-10 codes C18.0, C18.2–C18.7, C19, and C20) were identified. Surgical interventions were defined by a predefined set of CHOP (Swiss Classification of Surgical Procedures) codes, including right hemicolectomy, left hemicolectomy, sigmoid resection, low anterior resection and other major colorectal resections. Within this classification, procedures are distinguished according to open or minimally invasive approach, with an additional specific code available for robotic surgery.
Hospitals were identified using anonymized institutional identifiers and classified into three categories according to the official BFS scheme: K111 representing university hospitals (Versorgungsstufe 1), K112 representing large non-academic hospitals (Versorgungsstufe 2), and K12 representing primary care hospitals (Grundversorgung, including K121–K123). Performed procedures were subsequently analyzed by hospital category as well as by annual institutional case volume.

**Results:**
Between 2012 and 2022, a total of 33,418 colorectal cancer resections were performed nationwide, corresponding to 2,525–3,363 procedures annually; 44.2% of patients were female. The majority of patients were between 65 and 79 years of age. Early-onset colorectal cancer (<50 years) accounted for 6.7% of resections, with population-adjusted case numbers increasing by 57% over the study period. Most procedures (66.7%) were performed in large non-academic centres (K112). Only three hospitals achieved a median annual volume exceeding 100 resections. The proportion of patients treated in smaller hospitals (K12) declined markedly, from 38% in 2012 to less than 20% in 2022, whereas large non-academic hospitals demonstrated the strongest increase, rising from 50% to 71% of all resections. In 2012, 1,055 patients (42.6%) underwent surgery in high-volume centres with >60 cases/year; by 2022, this number had increased to 2,073 (63.0%), corresponding to a 50.9% rise in absolute numbers.
Minimally-invasive surgery demonstrated a late but notable national uptake, largely driven by robotic procedures. In 2022, 421 robotic resections were performed. University hospitals exhibited the steepest increase, with robotic cases projected to have surpassed open resections in 2023 or 2024. This shift was accompanied by a marked reduction in open procedures and, to a lesser extent, in laparoscopic surgery. In large non-academic hospitals, minimally invasive surgery also increased steadily, while open resections declined to below 40%.
When stratified by annual case volume, hospitals performing more than 60 resections per year showed a pronounced adoption of robotic surgery, paralleled by a decline in laparoscopic and open procedures. In contrast, hospitals with fewer than 60 resections per year displayed stable laparoscopic rates, little uptake of robotic surgery, but a consistent reduction in open resections across all hospital categories.

**Conclusion:**
Over the past decade, colorectal cancer surgery in Switzerland has undergone a measurable process of centralization. The proportion of patients treated in small hospitals declined substantially, while large non-academic centres absorbed the majority of procedures, and the number of patients operated in high-volume hospitals increased by more than 50%. Parallel to this structural shift, surgical practice evolved with a growing adoption of minimally invasive approaches, most notably a late but rapid increase in robotic resections that is projected to overtake open surgery at university hospitals. These trends indicate that political initiatives and increasing technical demands have gradually reshaped the delivery of colorectal cancer care, improving access to specialized centres and modern surgical techniques. Continued monitoring is warranted to evaluate whether these structural changes translate into improved oncologic and perioperative outcomes for patients.

DE GRUYTER BRILL

**Integrating PROMs into colorectal cancer care: Four-year experience of a colorectal cancer center**
ID: E-DCK-296

**Viszeralchirurgie / Visceral Surgery**
**Chirurgie unterer Gastrointestinaltrakt, Koloproktologie (CACP) / Lower Gastrointestinal Surgery and Coloproctology (CACP)**

Alexandra Claudia Klein[1], Larissa Klein[1], Hauke Lang[1], Karoline Horisberger[1]

[1]University Medical Center of the Johannes Gutenberg University, Department of General, Visceral, and Transplant Surgery, Mainz, Deutschland

**Background:**
Nowadays, the success of a treatment should also be measured in terms of functional life data and quality of life. Especially in colorectal surgery, interventions can be associated with postoperative functional limitations. However, patient-reported outcome measures (PROMs) are rarely used in clinical practice, especially after hemicolectomies. Therefore, we have initiated a project to systematically and routinely record PROMs in all patients undergoing resection of colorectal carcinoma, regardless of tumour site. PROMs are integrated into routine patient care to support clinical decision-making.

**Material and methods:**
This quality improvement project is being carried out in the Department of Colorectal Surgery of a German certified colorectal cancer center. Based on the recommendations of the International Consortium for Health Outcomes Measurement (ICHOM), a questionnaire was developed that combines validated instruments such as the MSKCC Bowel Function Instrument, the EORTC QLQ-CR29 and the Low Anterior Resection Syndrome Score (LARS Score). General symptoms, specific complaints of bowel function and continence as well as restrictions in sexual life are recorded. All patients presenting for resection of a colorectal carcinoma receive the questionnaire preoperatively for voluntary participation. The survey is repeated 3, 6, 12 and 24 months postoperatively - in each case with the offer to discuss symptoms in person. The data collection is pseudonymized.

**Results:**
Since April 2022, patients with a curative approach to colorectal cancer have been included. The postoperative response rate averages 82% across follow-up timepoints.

**Conclusion:**
Using PROMs in colorectal surgery clinics is feasible and can lead to high response rates. For patients, PROMs open up the possibility of tracking functional results after surgical treatment of colorectal carcinomas over the course of time, making the available consultation time more targeted and continuously adapting treatment to individual needs. At an institutional level, PROMs can increase the transparency of treatment quality and promote quality assurance.

DE GRUYTER BRILL

**What is the main predictor for postsurgical LARS in upper rectal cancer?**
ID: E-DCK-316

**Viszeralchirurgie / Visceral Surgery**
**Chirurgie unterer Gastrointestinaltrakt, Koloproktologie (CACP) / Lower Gastrointestinal Surgery and Coloproctology (CACP)**

Philine-Sophie Hoyer[1], Johannes Martens[1], Timotheus Ritter-Vanfloorop[1], Julie Schanz[2 3], Andreas Leha[4], Torsten Liersch[1]

[1]Klinik für Allgemein-, Viszeral- und Kinderchirurgie, Göttingen, Deutschland, [2]Klinik für Hämatologie und Medizinische Onkologie, Göttingen, Deutschland, [3]Institut für Klinische Chemie, Göttingen, Deutschland, [4]Institut für Medizinische Statistik, Göttingen, Deutschland

**Background:**
The impact of anastomotic height and loss of *ampulla recti* for low anterior resection syndrome (LARS) was assessed in participants of the GAST-05-trial (ISRCTN35198481) with locally advanced rectal cancers (≥ 12 cm above anal verge; stages II / III) after partial (PME) vs. total (TME) mesorectal excision followed by FOLFOX-CTx.

**Material and methods:**
PME / TME was performed in 98 patients (median age: 69 years; f: 33, m: 65 with good, moderate, or poor quality in 84.7 %, 14.3 % and 1.0 %, respectively. Acute / late adverse events (AEs), LARS and defecation disorders were categorized according to Dindo /Clavien-, NCI-CTC-AE-, POLARS- and Wexner-scoring systems. Data were correlated with diverse variables (e.g. gender, surgery, anastomotic height).

**Results:**
After PME / TME acute AEs (49.0 %) had been reported in 40.7 % and 59.1 %, respectively. For grade III / IV AEs, intervention was needed in 11.1 % and 22.7 %. During follow-up (85.5 months in median), LARS was diagnosed in 44.1 %, more frequently after TME (p = 0.007), predominantly in patients with anastomosis at ≤ 6 cm (from av). LARSwas mild, moderate, and severe in 20.4 %, 12.9 %, and 10.8 %, respectively. Multivariable ordinal regression models revealed the association between anastomotic height and LARS (OR = 0.73, 95%-CI [0.55; 0.96], p < 0.025). Scatter plot analysis showed a correlation between LARS and the Wexner-Score (Kendal`s $\tau$: 0.42, p < 0.0001).

**Conclusion:**
LARS occurred after PME and TME, but predominantly in patients with low anastomosis. Anastomotic height was a predictor for LARS.

DE GRUYTER BRILL

**Guideline-adherence in follow-up Colonoscopies after Colorectal Cancer Surgery**
ID: E-DCK-50

**Viszeralchirurgie / Visceral Surgery**
**Chirurgie unterer Gastrointestinaltrakt, Koloproktologie (CACP) / Lower Gastrointestinal Surgery and Coloproctology (CACP)**

Konstantin Lukas Uttinger[1], Laura Acar[2], Armin Wiegering[1]

[1]Klinik für Allgemein-, Viszeral-, Transplantations- und Thoraxchirurgie, Universitätsklinikum Frankfurt am Main, Frankfurt am Main, Deutschland, [2]Barmer Versicherung, Wuppertal, Deutschland

**Background:**
In Germany, colorectal carcinoma (CRC) is among the most frequently diagnosed cancers, with surgical resection being a cornerstone of therapy. Current S3-guidelines recommend surveillance colonoscopies after curative-intent resection.

**Material and methods:**
This is a retrospective longitudinal observational study based on health insurance claims data provided by BARMER health insurance company. All index hospitalizations between January 1st, 2009, and September 30th, 2017, due to colorectal cancer (ICD C18-C20) with a documented surgical procedure for tumor resection wwere included. The first primary outcome of interest was adherence to surveillance colonoscopy within 15 or 60 months after discharge. The second primary outcome of interest was disease-free survival (DFS).

**Results:**
A total of 62,352 patient records were analyzed, 66.5% (41,482) with colon carcinoma, 4.5% (2,826) with rectosigmoid carcinoma, and 28.9% (18,044) with rectal carcinoma. Median age was 72 (IQR 64-79). A follow-up colonoscopy was performed within 15 months in 35.4% (22,093) and/or within 60 months in 53.5% (33,361) of patients. Disease-free survival (DFS) was overall 66.0% (41,127) after 36 months and 56.4% (35,110) after 60 months. DFS was reduced among the non-followed-up in comparison to the followed-up in all time frames and in all tumor locations of CRC patients (e.g. DFS 41.3% in non-followed-up patients vs 71.5% in followed-up patients after 60 months in colon carcinoma). In a multivariable Cox hazards model for DFS, female sex was associated with reduced risk, while increased age, increased comorbidity, rectosigmoid and rectal carcinoma (vs colon carcinoma), and open index surgery (vs laparoscopic) were associated with increased risk of disease recurrence or death. Performance of at least one follow-up colonoscopy within 60 months was associated with decreased risk of disease recurrence or death (HR 0.41, p<.001). Readmissions for CRC within 60 months was more frequent among followed-up (4.9% vs 1.6% in non-followed-up). Readmissions for treatment of metastases were more frequently observed among the followed-up compared to the non-followed-up (e.g. readmissions for treatment of hepatic metastasis within 60 months: 5.5% vs 4.0%).

**Conclusion:**

In this retrospective cohort study, guideline-adherence regarding surveillance colonoscopies after curative-intent surgery in CRC patients was 53.5% regarding colonoscopies within 60 months after surgery. Adherence to colonoscopy surveillance was associated with improved disease-free survival in a multivariable Cox hazard model.

DE GRUYTER BRILL

**Enterocutaneous Fistula Following Surgery Treated by Placing a Targeted Drain: Case Series**
ID: E-DCK-511

**Viszeralchirurgie / Visceral Surgery**
**Chirurgie unterer Gastrointestinaltrakt, Koloproktologie (CACP) / Lower Gastrointestinal Surgery and Coloproctology (CACP)**

Helga Komi[1], Daniel Antonio Morales Santana[1], Reinhold Kafka-Ritsch[1]

[1]Department of Visceral, Transplant and Thoracic Surgery, Center of Operative Medicine, Medical University Innsbruck, Austria, Innsbruck, Österreich

**Background:**
Enterocutaneous fistulas (ECFs) are abnormal communications between the gastrointestinal tract and the skin that can ariseas complications of intestinal inflammation or postoperative leak. Although various treatment strategies have been described in the literature, the management of complex fistulas remains particularly demanding and continues to pose a challenge for surgeons. In this article, we present a case series of patients with complex ECFs and outline our surgical approach to their treatment.

**Material and methods:**
We present three cases of patients who underwent abdominal surgery, either for oncological indications or as emergency procedures due to ileus and/or intestinal inflammation.
All patients subsequently required multiple reoperations due to complications such as postoperative bleeding, adhesions, or intestinal perforation. Following these reinterventions, all patients developed intestinal leak, necessitating further surgical revision. Intraoperatively, all patients were found to have a frozen abdomen. Therefore, we decided against extensive adhesiolysis and performed suturing of the intestinal leak with targeted drain placement, followed by abdominal wall closure.

**Results:**
Postoperatively, the drain was gradually removed to allow controlled fistula output through the drain tract. Inflammatory markers peaked on the second postoperative day and showed rapid decline, with CRP levels ranging between 10 and 21 mg/dl. Drain output volume from the fistula decreased progressively, and ECF closure was achieved following drain removal. Imaging follow-up showed no residual fluid collections or abscess formation. The duration of targeted drain therapy varied depending on individual patient factors and comorbidities, ranging from postoperative day 2 to 45. The median length of hospital stay at our department was 63 days. However, discharge was possible at a median of 13 days after drain removal. This surgical approach helped achieve an improvement in quality of life, reduce morbidity, and enabled successful hospital discharge in all cases.

**Conclusion:**
Targeted intra-abdominal drain combined with abdominal wall closure should be considered as a treatment strategy for patients with a frozen abdomen and intestinal leaks to effectively manage ECFs. This approach facilitates patient mobility, reduces the risk of reoperation, promotes spontaneous fistula closure, improves quality of life.

DE GRUYTER BRILL

### Lymph Node Ratio added to N-Stage adds prognostic value in risk stratification for Colorectal Cancer

ID: E-DCK-64

**Viszeralchirurgie / Visceral Surgery**
**Chirurgie unterer Gastrointestinaltrakt, Koloproktologie (CACP) / Lower Gastrointestinal Surgery and Coloproctology (CACP)**

Francesca Cordera[1], Emil Bosshard[1], Charlotte Pradella[1], Jasmin Zeindler[1], Emanuel Burri[2], Robert Rosenberg[1]

[1]Kantonsspital Baselland, Klinik für Chirurgie, Liestal, Schweiz, [2]Kantonsspital Baselland, Klinik für Gastroenterologie und Hepatologie, Liestal, Schweiz

**Background:**
Lymph node metastases (LNM) are crucial in determining the prognostic outcome as well as the necessity of adjuvant treatment in colorectal cancer. Lymph node ratio (LNR), defined as the proportion of positive lymph nodes relative to the total number of resected lymph nodes, accounts for the quality of lymphadenectomy and the number of resected lymph nodes, and has been postulated to exert superior prognostic predication when added to conventional N-stage. We aimed to evaluate the predictive value and improved discriminatory power of LNR in risk stratification of 5-year overall survival in patients with colorectal cancer and to confirm existing LNR cut-offs in a cohort with a high lymph node yield.

**Material and methods:**
This is a single-centre retrospective cohort study. The study population included all patients undergoing surgical resection for colorectal cancer between 2014 and 2022 in a specialized centre in Switzerland. Predefined LNR cut-offs (LNR 0 = 0.00, LNR 1 = 0.01-0.17, LNR 2 = 0.18-0.41, LNR 3 = 0.42-0.69, LNR 4 = 0.70-1.00) were determined from literature. Survival rates according to N-stage and LNRs were analysed using uni- and multivariate Cox regression.

**Results:**
493 patients (40.2% females, stage 0: 2.6%, stage I: 23.9%, stage II: 31.0%, stage III: 24.3%, stage IV: 18.1%) were included. The median number of resected lymph nodes was 31. 5-year overall survival was 98% in N0-stage (n=301/493). Patients with LNMs (n=192/493) had worse 5-year overall survival with 86% in pN1a and 48% in pN2b. Increasing LNR showed decreasing 5-year overall survival ($p < 0.001$). Some patients in the LNR3 group showed pN1b stage. LNR emerged as independent prognostic factor in multivariate analysis (HR 1.77, 95% CI 1.53–2.04; $p < 0.001$).

**Conclusion:**
LNR resulted in a better discrimination of 5-year overall survival prediction when added to conventional N-stage compared to outcome prediction with N-stage alone. Especially LNR group 3 helps to improve risk stratification, as some patients with pN1b stage distribute into the LNR3 group. Additionally, our results were able to show, that LNR risk stratification is an independent prognostic factor predicting survival outcome in patients with CRC.

DE GRUYTER BRILL

**Surgical treatment of tailgut cysts: a single-center analysis of 23 cases**
ID: E-DCK-85

**Viszeralchirurgie / Visceral Surgery**
**Chirurgie unterer Gastrointestinaltrakt, Koloproktologie (CACP) / Lower Gastrointestinal Surgery and Coloproctology (CACP)**

Petro Zgurskyi[1], Nina Schraps[1], Baris Mercanoglu[1], Ramez Wahib[1], Jakob Izbicki[1], Thilo Hackert[1], Nathaniel Melling[1]

[1]Universitätsklinikum Hamburg-Eppendorf, Hamburg, Deutschland

**Background:**
Tailgut cysts are rare benign lesions with inflammatory and malignant potential. However, studies evaluating treatment outcomes in larger patient cohorts remain very limited; thus, clinical decision-making and treatment becomes challenging. To analyze the clinical characteristics and management strategies of patients with tailgut cysts.

**Material and methods:**
This is a retrospective, descriptive, single-center study conducted at the University Medical Center Hamburg-Eppendorf, a tertiary-level institution. The study included patients with histologically confirmed tailgut cysts or those managed under clinical suspicion of the disease between 2014 and 2025. The analysis focused on diagnostic accuracy, surgical strategy, postoperative morbidity, recurrence, and length of hospital stay.

**Results:**
This study included 23 patients (female, 73.9%; mean age, 40 years). The most frequent symptoms were lower back pain, lower abdominal pain, and anal pain (each, 17.4%). Meanwhile, 30.4% of the patients were asymptomatic. The median follow-up period was 34 (interquartile range, 5–62) months. All patients underwent magnetic resonance imaging, with 78.3% suspected with a tailgut cyst or benign cystic lesion preoperatively. The mean cyst diameter was 5.8 ± 3.07 cm, and 30.4% of the patients had a multilobular morphology. Additionally, a dorsal (Kraske) approach was used in 77.3% of patients. One patient underwent laparoscopic cyst resection, and four required rectal resection. Regarding postoperative complication, delayed wound healing was the most common (13.6%). Only two (8.7%) patients experienced recurrence.

**Conclusion:**
This study presents one of the largest single-center cohorts of resected tailgut cysts. Magnetic resonance imaging is typically sufficient for diagnosis. Multilobular cysts are associated with recurrence, often pose diagnostic challenges, and may lead to unnecessarily extensive surgical intervention, which is associated with an increased risk of complications. The dorsal (Kraske) approach combined with secondary wound healing proved safe and effective, yielding high patient satisfaction and low complication rates.

DE GRUYTER BRILL

**Risk Factors for Hypocalcemia and Vocal Cord Paralysis after Thyroidectomy in 3365 Cases**
ID: E-DCK-126

**Viszeralchirurgie / Visceral Surgery**
**Endokrine Chirurgie (CAEK) / Endocrine Surgery (CAEK)**

Carmen Kirchner[1], Sarah Krieg[2], Matthias Schott[3], Karel Kostev[4], Andreas Krieg[1]

[1]Department of General and Visceral Surgery, Thoracic Surgery and Proctology, Medical Campus OWL, Ruhr University Bochum, Herford, Deutschland, [2]Department of Inclusive Medicine, University Hospital Ostwestfalen-Lippe, Bielefeld, Deutschland, [3]Division for Specific Endocrinology, Heinrich-Heine-University and University Hospital Duesseldorf, Duesseldorf, Deutschland, [4]Epidemiology, IQVIA, Frankfurt, Deutschland

**Background:**
Paralysis of the vocal cords and larynx and hypoparathyroidism are the two main complications following thyroid surgery that can potentially affect patients in the long term. The prevention of these diseases by identifying potentially at-risk patients with a differentiated surgical approach and prompt, targeted therapy represents a major challenge in endocrine surgery.
Injury of the recurrent laryngeal nerve (RLN) resulting in VCP occurs in 1.5% to 20% of patients. The use of intraoperative nerve monitoring (IONM) during thyroid surgery has shown superior results compared to visual inspection alone with a significant up to 30-fold decrease in permanent nerve lesions. In the development of VCP after thyreoidectomy a variety of risk factors has been described including invasive malignancies necessitating extensive lymph node dissection, large thyroid volumes (>100 mL), retrosternal goiters, anatomical variations of the recurrent laryngeal nerve (RLN), reoperations and reliance on visual nerve identification without intraoperative neuromonitoring (IONM). Also advanced tumor stage, multifocal disease, and prolonged operative time have been identified as independent predictors of transient VCP.
Hypocalcemia is a frequent complication in 1.2% to 40% of patients after thyroidectomy. Risk factors include surgical technique, extent of resection and underlying disease and female sex.  In the course of gender medicine, the differentiated view of diseases is increasingly coming into focus as the risk of postoperative hypocalcemia is not equally distributed among patients and appears to be influenced by both biological sex and metabolic comorbidities. Benign thyroid nodules and autoimmune thyroid disorders are more frequently in women whereas men present more often with advanced thyroid carcinoma at an older age. The expression of estrogen receptors on thyroid epithelial and progenitor cells, may play a role in this context by promoting hormone-driven growth and cellular activity.
Apart from hormonal differences, metabolic-driven diseases like obesity, type 2 diabetes mellitus and insulin resistance seem to have an influence in the development of thyroid disorders. A higher prevalence of thyroid nodules has been recognized in obese patients possibly due to modulation of endocrine signaling pathways through peripheral estrogen production in adipose tissue. Additionally, obesity, especially in women, has been identified as an independent risk factor for the development of thyroid carcinoma.
There is few information on how sex and metabolic comorbidities influence postoperative outcomes. Therefore we focused in this large multicenter cross-sectional study of 27 German hospitals with 3365 cases on the prevalence and predictors of hypocalcemia and VCP following thyroid surgery with particular attention given to the role of sex, obesity and diabetes mellitus in influencing postoperative outcomes.

**Material and methods:**
We conducted a multicenter cross-sectional analysis using anonymized data from 3,365 patients (1,517 hemithyroidectomies; 1,848 thyroidectomies) aged 18 or older that were treated at 27 German hospitals between 2019 and 2024. Therefore we utilized data from the IQVIA hospital database, including records from 49 hospitals of approximately 2,143,071 hospitalizations between January 1, 2019, and January 31, 2024, in Germany including specialized hospitals, primary care hospitals, maximum care facilities, standard care facilities and university hospitals. According to §21 of the Hospital Compensation Act (KHEntgG), data are transmitted to the Reimbursement Institute for Hospitals (InEK) in a standardized format. To protect data, export files were anonymized (e.g., case and patient numbers) before transmission.

The primary outcomes of the study were the prevalence of two surgical complications: postprocedural hypoparathyroidism (ICD-10: E89.2) and VCP (ICD-10: J38.0). Baseline characteristics of the study patients included age groups (≤45, 46-55, 56-65, >65 years), sex, documented thyroid gland disorders (nontoxic single thyroid nodule, ICD-10: E04.1; nontoxic multinodular goitre, ICD-10: E04.2; thyrotoxicosis with diffuse goitre, ICD-10: E05.0; thyrotoxicosis with toxic multinodular goitre, ICD-10: E05.2; benign neoplasm of thyroid gland, ICD-10: D34; malignant neoplasm of thyroid gland, ICD-10: C73), obesity (ICD-10: E66), diabetes mellitus (ICD-10: E10-E14), and hypertension (ICD-10: I10).
Baseline characteristics were compared between patients who underwent hemithyroidectomy and thyroidectomy. Student's t-test was performed for continous variables, while chi-squared tests were utilized for categorical variables. To assess associations between age, sex, surgery type, diagnoses and each complication a multivariable logistic regression analysis was used. Patients who underwent hemithyroidectomy or thyroidectomy were analyzed separately. The findings of these models were expressed as odds ratios (OR) and 95% confidence intervals (CI) for each variable. Statistically significant results were defined as P-values less than 0.05. All analyses were performed using SAS 9.4 (SAS Institute, Cary, US).

**Results:**
A total of 3,365 cases (1,517 with hemithyroidectomy and 1,848 with thyroidectomy) in 27 hospitals qualified for further analysis. In patients undergoing hemithyroidectomy the mean age was 54.0 (SD: 14.1) years and 55.2 (SD: 13.8) years in patients undergoing thyroidectomy.

Patients who underwent hemithyroidectomy had slightly higher proportion of men (29.5% vs. 26.2%) and had higher rates of nontoxic single thyroid nodule (34.8% vs. 6.9%) and benign neoplasm of thyroid gland (15.6% vs. 9.1%) compared to those who had underwent thyroidectomy. In contrast, patients with thyroidectomy had higher proportion of thyrotoxicosis with diffuse goitre (18.8% vs. 1.7%), thyrotoxicosis with toxic multinodular goitre (5.9% vs. 1.3%), nontoxic multinodular goitre (52.6% vs. 42.3%) and malignant neoplasm of thyroid gland (14.2% vs. 11.2%)

In patients, who underwent hemithyroidectomy, hypoparathyroidism was prevalent in 0.3% of patients, whereas in patients, who underwent thyroidectomy this complication occured in 3.6% of patients. Among patients who underwent thyroidectomy, female gender was significantly associated with hypoparathyroidism (OR: 2.30; CI: 1.11-4.77) since obesity was negatively associated with hypoparathyroidism (OR: 0.15; 95% CI: 0.04-0.63) as shown in multivariable logistic regression.

VCP was observed in 1.7% of patients who underwent hemithyroidectomy and 1.0% of patients who underwent thyroidectomy.

Malignant neoplasm of the thyroid gland was significantly associated with an increased risk of VCP (odds ratio [OR]: 4.00; 95% confidence interval [CI]: 1.37-1.64) as well as thyrotoxicosis with diffuse goiter was strongly associated with this complication (OR: 4.94; 95% CI: 0.86-28.37) with a low level of significance (p = 0.074).

Simultaneous parathyroidectomy proved as a significant risk factor for VCP (odds ratio [OR]: 3.47; 95% confidence interval [CI]: 1.04-11.59) in patients who underwent thyroidectomy. Also the prevalence of diabetes negatively influenced the occurence of VCP (OR: 3.09; 95% CI: 0.98-9.74), although this association was only marginally significant (p = 0.055).

**Conclusion:**

In our study, our patient cohort was comparable to those reported by other others with predominantly female patients in hemithyroidectomy (70.5%) and thyroidectomy (73.8%). Also the average patient age (54.0 years in hemithyroidectomy; 55.2 years in thyroidectomy) is consistent with those reported in the literature as well as the incidence of parathyroidectomy with 7% of patients in our study population.

As expected, hypocalcemia occurred more frequently in patients who underwent thyroidectomy (3.6%) than in those who underwent hemithyroidectomy (0.3%), as the extent of the surgical procedure is significantly greater without the possibility of compensating for parathyroid injury on the contralateral side, as is the case with hemithyroidectomy. Therefore, careful consideration of individual risks and the existing pathology in relation to the planned scope of the operation appears to be particularly important.

Female sex was an independent risk factor for the occurrence of postoperative hypocalcemia. These data are also in line with other studies. Reasons for this may include differences in vitamin D metabolism, gender-specific characteristics of thyroid disorders, and variations in weight histology of the parathyroid gland. In addition, it has been shown that men are more likely to develop malignant diseases in later life, which may be due to different biological factors, but also to behavioral patterns involving earlier contact with doctors in women for cosmetic reasons. Further investigations for pre- and postoperative hormonal parameters are needed in this context.

Interestingly, obesity was a protective factor in the occurence of hypocalcemia. Compensated secondary hyperparathyroidism due to altered calcium metabolism in obese patients offers one possible explanation. Animal studies show that a high-fat diet increases intracellular calcium and is associated with altered cAMP signaling in adipose tissue. Underreporting of obesity, a more cautious approach to challenging cases, and supplementation with calcium or vitamin D also may explain this protective trend.

Vocal cord paralysis occurred more frequently in larger and more complex procedures. Risk factors here were simultaneous parathyroidectomy, malignant diseases, and diffuse goiter, possibly as a result of difficult dissections.

Diabetes mellitus also presented an increased risk of vocal cord paralysis, which has already been described in connection with peripheral neuropathy and increased latency of the recurrent laryngeal nerve (RLN) after thyroidectomy.

A slightly higher rate of vocal cord paralysis after hemithyroidectomy (1.7%) compared to thyroidectomy (1.0%) was a surprising initial finding. This may be due to the termination of surgery in some cases following loss of signal during intraoperative neuromonitoring, and emphasizes the importance of data interpretation and a patient-specific surgical approach.

Despite all limitations, we were able to show that there are certain key factors in thyroid surgery that influence postoperative outcomes. These include female gender as an independent risk factor for hypocalcemia and obesity as a protective factor in this context. Risk factors for the occurrence of vocal cord paralysis were malignancy, parathyroidectomy, diffuse goiter, and diabetes mellitus.

These results emphasize the importance of individualized surgical planning. Further prospective studies with detailed laboratory and surgical data are needed to improve thyroid surgery.

DE GRUYTER BRILL

**Preoperative malnutrition is a frequent risk factor in patients with pancreatic neurocrine tumours**
ID: E-DCK-13

**Viszeralchirurgie / Visceral Surgery**
**Endokrine Chirurgie (CAEK) / Endocrine Surgery (CAEK)**

Alina Ritter[1], Freya Brodersen[1], Antonie Willner[1], Jelte Poppinga[1], Kira Steinkraus[1], Feline Ockenga[1], Fabrice Viol[2], Faik Uzunoglu[1], Tania Amin[2], Thorben Fründt[2], Samuel Huber[2], Jakob Izbicki[1], Thilo Hackert[1], Anna Nießen[1]

[1]Universitätsklinikum Hamburg-Eppendorf, Klinik für Allgemein-, Viszeral- und Thoraxchirurgie, Hamburg, Deutschland, [2]Universitätsklinikum Hamburg-Eppendorf, I. Medizinische Klinik und Poliklinik, Hamburg, Deutschland

**Background:**
Pancreatic neuroendocrine tumours (pNETs) are rare neoplasms. Surgical resection remains the only curative option. In order to reduce perioperative stress, ERAS (*Enhanced Recovery After Surgery*) programs are applied nowadays. One aspect of the ERAS concept involves screening patients for their risk of malnutrition (ROM) preoperatively. However, little is known about the relevance of ROM in pNET patients. The prevalence of ROM in pNET patients and its influence on postoperative outcome was assessed at an ERAS and ENETS (*European Neuroendocrine Tumor Society*) Center of Excellence.

**Material and methods:**
Patients that were operated on between 03/2017 and 01/2025 due to a pNET or pNET liver metastasis at the University Medical Center Hamburg-Eppendorf and participated in the local ERAS program, were retrospectively analysed from a prospectively maintained database. ROM was defined by ≥ 2 points in the Nutritional Risk Screening 2002. Clinical data was analysed using univariate analysis.

**Results:**
Within the examined time span, 58 pNET patients participating in the institutional ERAS program, were screened for malnutrition. A ROM was evident in 26 patients (44.8%) and was more frequent in poorly differentiated pNETs. In patients with ROM, open operations, total pancreatectomies and venous resections were more common. There was no statistically significant difference regarding age, comorbidities and ERAS-compliance between patients with and without ROM. In patients with ROM, pre- and postoperative nutrition treatment was significantly more frequently applied. Patients with and without ROM did not significantly differ regarding 30-day-mortality, major complications and intensive care unit stay.

**Conclusion:**
This study shows, for the first time, that preoperative malnutrition affects more than one third of pNET-patients. Introduction of a structured ERAS program with preoperative malnutrition screening and perioperative nutrition treatment positively affects postoperative outcome especially in extensive tumour resections.

DE GRUYTER BRILL

### A Novel Nomogram for Predicting Clinical Success after retroperitoneoscopic Adrenalectomy in PHA

ID: E-DCK-469

**Viszeralchirurgie / Visceral Surgery**
**Endokrine Chirurgie (CAEK) / Endocrine Surgery (CAEK)**

Eslam Elmaghraby[1], Shahrooz Babaei[1], Wolfram Trudo Knoefel[1], Andreas Krieg[2]

[1]Heinrich-Heine-University and University Hospital Duesseldorf, Department of Surgery (A), Duesseldorf, Deutschland, [2]University Hospital Herford, Medical Campus OWL, Ruhr University Bochum, Department of General and Visceral Surgery, Thoracic Surgery and Proctology, Herford, Deutschland

**Background:**
Retroperitoneoscopic adrenalectomy is the standard treatment for unilateral primary hyperaldosteronism (PHA), resulting in biochemical cure in most patients. However, resolution of hypertension remains less predictable. This study aimed to evaluate clinical and biochemical outcomes and to develop a nomogram predicting the probability of clinical success after surgery.

**Material and methods:**
We retrospectively analyzed data from 122 patients who underwent retroperitoneoscopic adrenalectomy for PHA between 2006 and 2021. Preoperative and postoperative clinical, biochemical, and medication data were collected. Multivariate logistic regression analysis was performed to identify independent predictors of clinical success. These predictors were then incorporated into a nomogram to estimate the probability of complete clinical success.

**Results:**
Postoperatively, patients showed significant improvements, including marked reductions in aldosterone levels ($p<0.001$), aldosterone/renin-ratio ($p<0.001$), systolic and diastolic blood pressure ($p<0.001$ and $p<0.01$), and daily antihypertensive medication requirements ($p<0.001$) as well as the Defined Daily Dose ($p < 0.001$). Serum potassium levels ($p < 0.001$) and renin levels increased significantly ($p < 0.001$). Overall, biochemical success was achieved in 82.5% of patients. Complete clinical success was attained in 20.8% of patients, while 61.7% achieved partial success. The nomogram predicting complete clinical success included several preoperative variables—age, duration of hypertension, serum potassium, and antihypertensive medication dosage—and demonstrated excellent discrimination, with an area under the curve (AUC) of 0.925, outperforming existing scoring systems.

**Conclusion:**
Retroperitoneoscopic adrenalectomy is a safe and effective treatment for PHA, providing high rates of biochemical cure and substantial clinical improvement. The novel nomogram developed in this study serves as a highly accurate tool for predicting complete clinical success and may be invaluable for preoperative patient counseling and postoperative management. Such a nomogram-based scoring system can also aid in risk stratification and in minimizing preventable complications.

DE GRUYTER BRILL

**Perioperative outcome of pNET in the context of different body composition phenotypes**
ID: E-DCK-489

**Viszeralchirurgie / Visceral Surgery**
**Endokrine Chirurgie (CAEK) / Endocrine Surgery (CAEK)**

Fabiola Bechtiger[1], Mariah Herbrich[1], Hazem Karim Anwar Abdelgawad Hassan[1], Jörg Kaiser[1], Thomas Hank[1], Magdalena Lewosinska[1], Miriam Klauß[2], Christoph Kahlert[1], Deniz Uluk[1], Henrik Nienhüser[1], Ralph Brecheisen[3], Steven Olde Damink[3], Martin Loos[1], Christoph Michalski[1], Franck Billmann[1], Markus W. Büchler[1], Zoltan Czigany[1]

[1]Universitätsklinikum Heidelberg, Klinik für Allgemein-, Viszeral- und Transplantationschirurgie, Heidelberg, Deutschland, [2]Universitätsklinikum Heidelberg, Klinik für diagnostische und interventionelle Radiologie, Heidelberg, Deutschland, [3]Maastricht University Medical Centre, Department of Surgery, Maastricht, Niederlande

**Background:**
Pancreatic neuroendocrine neoplasms (pNENs) are rare and heterogeneous tumors with increasing incidence. While surgical resection remains the only curative treatment, perioperative outcomes are influenced by patient-related risk factors. Recent research highlights body composition (BC) as a prognostic marker in oncological surgery. This study evaluates the impact of BC on perioperative morbidity in patients undergoing surgery for pNENs.

**Material and methods:**
All consecutive patients who underwent surgery for pNENs at the University Hospital Heidelberg between 2010 and 2023 were retrospectively analyzed. BC was assessed using preoperative CT scans at the L3 level with AI-based software (Mosamatic™). Parameters included skeletal muscle index (SMI), muscle radiation attenuation (SM-RA), visceral and subcutaneous fat, and derived definitions of sarcopenia, myosteatosis, adipopenia, sarcopenic obesity, and visceral obesity. Postoperative morbidity was classified according to Clavien-Dindo and the Comprehensive Complication Index (CCI). Logistic regression was used to identify predictors of 90-day morbidity.

**Results:**
A total of 622 patients were included, of whom 480 had evaluable CT scans. Median age was 59.1 years and 53.8% were male. Obesity was present in 59.3%, sarcopenia in 49.2%, myosteatosis in 50.0%, adipopenia in 48.6%, sarcopenic obesity in 21.5%, and visceral obesity in 60.1%. Obesity, visceral obesity, sarcopenic obesity, and myosteatosis were associated with significantly higher complication rates, longer hospital stays, or increased CCI, while adipopenia showed some association with reduced postoperative pancreatic fistula and morbidity. In multivariate analysis, ASA ≥3 and prolonged surgery remained independent predictors of major complications.

**Conclusion:**
Impaired body composition is highly prevalent in pNEN patients. Obesity-related phenotypes and myosteatosis negatively impact perioperative outcomes, underscoring the relevance of BC assessment in preoperative risk stratification and surgical decision-making.

DE GRUYTER BRILL

**Case Report: Five year old forgotten Common Bile Duct Stent forms a stable colic-jejunal Fistula**
ID: E-DCK-72

**Viszeralchirurgie / Visceral Surgery**
**Endoskopie und Sonographie (CAES) / Endoscopy and Sonography (CAES)**

Andreas Felix Lührs[1], Anne Glitsch[1], André Schreiber[1], Stephan Kersting[1]

[1]Universitätsmedizin Greifswald, Klinik für Allgemeine Chirurgie, Viszeral-, Thorax- und Gefäßchirurgie, Greifswald, Deutschland

**Background:**
The implantation of plastic or metallic stents is a common and widely practiced intervention in modern medicine. Medical indications for the implant are various such as obstruction of the common hepatic duct (CBD) because of stones or due to a malignant disease or a leaking bile duct after cholecystectomy. As with all implanted, non-permanent foreign bodies, the risk of dislocation is always prevalent. Although the majority of stents are either removed or pass without complications, this case presents an unusual outcome—a stable colic-jejunal fistula forming five years after the initial CBD stent implantation. To the best of our knowledge, this is the first documented instance of such a delayed complication, highlighting a rare but significant risk.

**Material and methods:**
Endoscopy (EGD and Colonoscopy) were used for diagnostics and to extract the foreign body

**Results:**
Case Report
The 50-year-old male patient with known history of Crohn´s disease presented himself in the emergency department with headaches, fever and radiating pain from his flanks to his abdomen. The Patient has a wide history of operative procedures already performed such as open cholecystectomy (2019), Ileocecal resection as well as multiple anorectal abscesses due to Crohn´s Disease. Regarding his Crohn´s disease, the patient was on Mesalazine orally 3g/day  in a stable disease status. Laboratory tests revealed septic findings, with a white blood cell count of 31.1 Gpt/l, C-reactive protein elevated to 171 mg/l and mildly elevated creatinine to 108 µmol/l. Liver and bile parameters were all normal. Urinalysis showed nitrite-negative leucocyturia (52-250 cells/µl), proteinuria (660-2000 mg/l) and a microhematuria. Point-of-care ultrasound examination ruled out hydronephrosis and free intraabdominal fluid. The patient was set on i.v. antibiotics, and a Foley catheter was inserted. Following positive blood cultures with *Klebsiella pneumoniae*, the antibiotics were adjusted accordingly. Further diagnostics included a CT-Scan, which revealed numerous small abscesses within both kidneys and, surprisingly, a CBD-stent forming a fistula between the colon and small intestine. The patient reported that the CBD-Stent has been placed 2019 in a neighbouring hospital following a complicative cholecystitis. However, he was unaware that the foreign body had to be extracted after certain time. Interestingly, the patient denied any abdominal pain or discomfort, despite the presence of the fistula. His only symptom was a noticeably reduced time between eating and the need for bowel movements. This lack of acute symptoms underscores the insidious nature of the stent migration and fistula formation. To assess the damage firstly an oesophagogastroscopy was first performed, but no fistula could be identified in the accessible duodenale regions. During a subsequent colonoscopy the stent was found impacted at the right colonic flexure. Adjacent to the stent, the ascending colon appeared highly stenosed and could not be intubated with the colonoscope. We decided to extract the CBD-Stent which was easily accomplished. As no insufficiency of the fistula was evident the patient was discharged in good health after sufficient treatment of the pyelonephritis with an appointment scheduled to discuss surgical exploration. As of now the patient, being in good health, declined any surgical intervention and agreed to a yearly follow-up including colonoscopy and CT imaging.

**Conclusion:**

The migration of implanted foreign bodies can lead to acute, life-threatening conditions. Many case reports describe devices such as a CBD-stent leading to duodenal perforation and, in some cases, severe pancreatitis (1,2). A similar problem is seen in urology, where forgotten or displaced **double-J stents** can cause serious complications.

In this case, however, the patient was apparently unaware of the fistula forming in his intestines following the **likely spontaneous dislocation of the stent.** There are reports of CBD stents perforating the duodenum, but these typically occur shortly after implantation, with patients seeking immediate medical attention (4). He et al. recently reported a case of a duodenocolic fistula caused by a migrating CBD stent that had been in situ for approximately two years. They successfully treated the fistula through endoscopic clipping of both the colon and duodenum (5). **In contrast to similar reports** (6)**,** in our case, only the aboral end of the fistula was accessible via colonoscopy. Due to significant anastomosis stenosis of the ascending colon, which poses a high risk for developing a subsequent ileus, no clipping was performed ensuring bowel passage. This case underscores the critical need for establishment of a robust registry system in endoscopic departments, ensuring that patients are reminded of the need for stent removal. It also emphasizes the importance of clear communication between physicians and patients regarding the long-term management of implanted devices. Particularly in patients with chronic conditions, or those at risk of being lost to follow-up—as seen during the disruptions of the COVID-19 pandemic—such measures could help prevent complications like the one described here. While CBD stents are typically well tolerated, this case serves as a reminder of the risks associated with implants subjected to timely replacement, especially when regular follow-up is missed. Verifying proper positioning and functionality of these devices is essential to avoid rare but possibly severe complications.

DE GRUYTER BRILL

**Cost comparison of robotic colorectal surgery with and without a clinical pathway**
ID: E-DCK-239

**Viszeralchirurgie / Visceral Surgery**
**Gesundheitspolitik & Ökonomie / Health Policy and Economics**

Ulrich Ronellenfitsch[1], Alfonso Valenzuela Hurtado[2], Manuela De Allegri[2], Onur Bayram[1], Johannes Klose[1], Jörg Kleeff[1]

[1]Universitätsklinikum Halle (Saale), Universitätsklinik und Poliklinik für Viszerale, Gefäß- und Endokrine Chirurgie, Halle (Saale), Deutschland, [2]Universitätsklinikum Heidelberg, Heidelberg Institute of Global Health, Heidelberg, Deutschland

**Background:**
Clinical pathways (CP) are guidelines to standardize processes, improve quality of care and maximize resources through the implementation of evidence-based care for a medical procedure, thus reducing variation and risk. Our aim was to evaluate cost of care for robotic colorectal surgery using a CP compared to surgery without a CP.

**Material and methods:**
This was a non-interventional micro-costing study with a hospital level perspective. It was carried it out using the three standard steps. First, all resources involved in the implementation of the CP were identified. Second, resource consumption was measured. Third, the resources identified were valued and measured to estimate the average cost per group. This included the costs related to perioperative care and treatment of complications. Finally, a sensitivity analysis was conducted based on the hypothetical capacity utilization of the robotic equipment, costs of CP development and a discount rate of 3% over a period of five years.

**Results:**
The study populationcomprised 63 patients, 21 from group 1 (robotic colorectal surgery with CP) and 42 from group 2 (robotic colorectal surgery without CP). The mean cost per patient in group 1 was €12,663 (variation: €8,093 to €26,475); while the mean cost per patient in group 2 was €11,128 (variation: €8,357 to €17,628). The cost drivers for group 1 were materials and disposables, and staff, with 33% and 20% of total costs respectively. For group 2 they accounted for 38% and 20% of total costs. Group 1 was used for the sensitivity analysis. In year 1, at 100% utilization the mean cost per patient was €12,364, at 20% utilization it was €17,883. In year 5, at 100% utilization the mean cost per patient was €13,986, at 20% utilization it was €20,519.

**Conclusion:**
The results of our study indicate that using a CP for perioperative care of patients undergoing robotic colorectal surgery does not reduce cost. Staff expenses and materials are the main cost drivers in the care of patients regardless of CP usage.

DE GRUYTER BRILL

**Rare germ cell tumor as suspicious lesion of pancreas parenchyma in chronic pancreatitis**
ID: E-DCK-123

**Viszeralchirurgie / Visceral Surgery**
**Hepato-Pankreato-Biliäre Chirurgie (CALGP) / Hepato-Pancreato-Biliary Surgery (CALGP)**

Seong Jeong[1], Sara Acciuffi[1], Frank Meyer[1], Roland Croner[1], Sara Al-Madhi[1]

[1]Otto-von-Guericke-Universität mit Universitätsklinikum, Klinik für Allgemein-, Viszeral-, Gefäß- und Transplantationschirurgie, Magdeburg, Deutschland

**Background:**
Rare (cystic) tumor (Tu) lesions of the pancreas can be considered challenging w/ regard to diagnostic & therapeutic management.

**Material and methods:**
Scientific report on an uncommon case

**Results:**
Case description:
*Medical history*: The 41-years old male patient was transferred because of an unclear cystic lesion of the pancreas corpus in known chronic pancreatitis.
*Clinical finding*: General/nutritional status - good, slight obesity; abdominal wall: soft
*Diagnostics*: - Laboratory parameters - white blood cell count: 11.4 Gpt/L; blood glucose: 7.06 mmol/L
- Tu markers: CEA: 1.83 µg/L; CA19-9: 22.1 U/mL
- EUS: Cyst w/ echo-rich inhomogeneous content w/o flow of appr. 4 cm in size, solidly appearing, caseating smear - transgastric puncture (19-G needle): No hint for inflammation or malignant Tu growth (according to Pappenheim staining)
- MRI: Encapsulated, septate, fluid-filled Tu lesion of the pancreas corpus reaching the liver hilus
*Treatment*: Surgical intervention (roboter-assisted cystic enucleation at the pancreas corpus): Intraoperatively, opening of the lesser sac & preparation of the cystic Tu lesion (5 cm in size), well-defined at the upper margin of the pancreas corpus & adherent to the common hepatic artery; macroscopically, R0 resection status
*Histopathological investigation*:
- Mature cystic teratoma (so-called dermoid cyst) of 6 cm in size - considered germ cell Tu, w/o malignization
- Lymph nodes (LN, obtained from coeliac trunk): Four Tu-free LN w/ accentuated sinushistiocytosis
*Clinical course*: Discharge on the 2nd postop. day
*Outlook*/*prognosis*: After complete removal (as achieved), local Tu lesion seems to be reliably cured.

Diagnosis: Rare germ cell Tu presented as suspicious cystic pancreas parenchyma lesion in chronic pancreatitis

**Conclusion:**
Germ cell Tu of the pancreas extends the spectrum of suspicious Tu(-like) lesions of pancreas, the meticulous verification of which is essential for a finding-specific decision-making w/ regard to an adequate therapeutic approach w/ good prospects (e.g., ”watch & wait“ *vs*. resection).

**A new scoring system for preoperative risk evaluation for HCC using the Studoq|Liver registry**
ID: E-DCK-146

**Viszeralchirurgie / Visceral Surgery**
**Hepato-Pankreato-Biliäre Chirurgie (CALGP) / Hepato-Pancreato-Biliary Surgery (CALGP)**

Sebastian Knitter[1], Eriselda Keshi[1], Nils Haep[1], Philipp Haber[1], Nathanael Raschzok[1], Wenzel Schöning[1], Johann Pratschke[1], Felix Krenzien[1]

[1]Chirurgische Klinik, Charité – Universitätsmedizin Berlin, Berlin, Deutschland

**Background:**
Major postoperative complications remain a key driver of morbidity, hospital stay and resource use in patients undergoing liver surgery for hepatocellular carcinoma (HCC). Surgeons need a pragmatic and easy-to-use tool that stratifies risk for complications preoperatively and communicates it clearly to patients. Building on routinely available parameters, we aimed to derive and internally validate a simple point-based scoring system for predicting major complications after liver resection for HCC using the German StuDoQ|Liver registry.

**Material and methods:**
Clinicopathological data on patient-, tumor- and procedure-related factors from patients who underwent surgery at 13 German centers from 2017 to 2022 was extracted from the StuDoQ|Liver registry of the German Society of General and Visceral Surgery. Postoperative complications were graded according to the Clavien-Dindo classification. Clavien-Dindo grade ≥3a defined major postoperative complications. The dataset was randomly split, stratified by major complications, into training (70%) and validation cohorts (30%). Univariate analyses used Fisher's exact test or the chi-square test for categorical variables and the Mann–Whitney U test for continuous variables, as appropriate. Variables with $p$ <0.05 were entered in a multivariable logistic regression model. Odds ratios of parameters with $p$ <0.05 from the multivariate analysis were used for a weighted point score. The resulting total score was grouped in low, medium and high-risk groups. Model performance in the validation cohort was assessed by the area under the receiver-operating characteristic curve (AUC) and by observed event rates within risk strata. All statistical analyses were conducted using R software (version 4.5.1, The R Foundation for Statistical Computing, Vienna, Austria).

**Results:**
Of all 6,763 patients in the registry, 1,056 patients underwent surgery for HCC. The whole cohort was randomly split into training (n = 739) and validation (n = 319) cohorts in a 70:30 proportion, stratified by the primary outcome major complications to maintain similar event rates in both sets. In the training set, patients with major complications (11%) differed from those without (89%) in several baseline and operative characteristics. In univariate analysis, the following parameters were found to be different between the two groups: ASA classification ≥3 (77% vs. 65%, p = 0.043), immunosuppressive therapy (6% vs. 1%, p = 0.035), portal vein thrombosis (10% vs. 4%, p = 0.046), open surgery (64% vs. 48%, p = 0.012), major liver resection (47% vs. 21%, p < 0.001), and median duration of surgery (223 vs. 187 minutes, p = 0.001). In multivariate analysis, ASA classification ≥3 (OR 2.00, 95% CI [1.11, 3.78], p = 0.026), immunosuppressive therapy (OR 4.05, 95% CI [0.99, 14.1], p = 0.034), and major liver resection (≥ 3 liver segments; OR 2.77,

95% CI [1.58, 4.87], $p < 0.001$) were found to be independently associated with the development of major complications (Table 1).
A simple risk score was derived from these coefficients and weighted according to the odds ratio: ASA ≥3 = 1 point, immunosuppressive therapy = 2 points, and major liver resection = 2 points, resulting in a scoring from 0-5 points. Based on observed event rates, we defined risk strata as low (0 points), medium (1–2 points), and high (3–5 points). Applied to the validation cohort, the score showed a clear monotonic increase in observed major complication rates from low to high risk (Cochran–Armitage trend test Z = -2.06, p = 0.020), with modest discrimination (AUC 0.60; 95% CI 0.50–0.70; Figure 1).

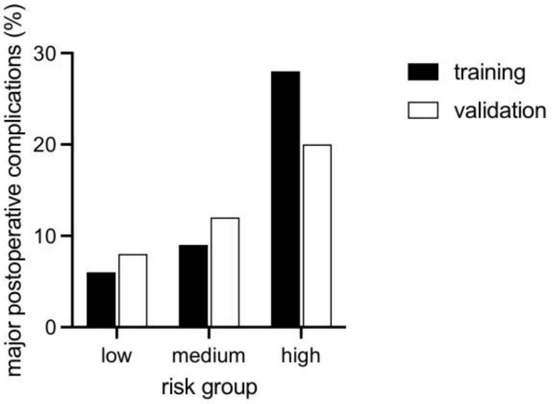

***Figure 1.*** *Incidence of postoperative major complications in training and validation cohorts*

**Conclusion:**
We propose a transparent three-item score (ASA ≥ 3, immunosuppressive therapy, major resection) that stratifies patients planned for liver surgery for HCC into low, medium, and high risk for major postoperative complications. The tool is simple to compute at the bedside and demonstrates a clear gradient in observed risk, though overall discrimination is modest. External validation is warranted, and future refinements such as incorporation of additional routinely available predictors may improve performance without compromising usability.

***Table 1.*** *Logistic regression analysis of demographic and surgical parameters associated with major postoperative complications*

| Characteristic | OR | 95% CI | p-value |
|---|---|---|---|
| **ASA ≥3** | 2.00 | 1.11, 3.78 | **0.026** |
| **Immunosuppressive therapy** | 4.05 | 0.99, 14.1 | **0.034** |
| **Portal vein thrombosis** | 2.19 | 0.53, 7.61 | 0.2 |
| **Open surgery** | 1.59 | 0.92, 2.81 | 0.10 |
| **Major resection (≥3 segments)** | 2.77 | 1.58, 4.87 | **<0.001** |
| **Duration of surgery ≥258 min** | 1.75 | 0.98, 3.07 | 0.053 |

Abbreviations: CI = Confidence Interval, OR = Odds Ratio

DE GRUYTER BRILL

**MRE-based prediction of HCC recurrence with liver and tumor shear stiffness – a meta-analysis**
ID: E-DCK-148

**Viszeralchirurgie / Visceral Surgery**
**Hepato-Pankreato-Biliäre Chirurgie (CALGP) / Hepato-Pancreato-Biliary Surgery (CALGP)**

Agnes Böhm[1], Felix Strobl[2], Theresa Keller[3], Anna Speth[4], Philipp Haber[1], Felix Krenzien[1 5], Ingolf Sack[4 6], Johann Pratschke[1 6], Igor Maximilian Sauer[1 6], Karl Hillebrandt[1 5]

[1]Charité – Universitätsmedizin Berlin, corporate member of Freie Universität Berlin and Humboldt-Universität zu Berlin, Department of Surgery, Augustenburger Platz 1, 13353 Berlin, Germany, Berlin, Deutschland, [2]Charité – Universitätsmedizin Berlin, corporate member of Freie Universität Berlin and Humboldt-Universität zu Berlin, Department of Vascular Surgery, Hindenburgdamm 30, Berlin, Deutschland, [3]Charité – Universitätsmedizin Berlin, Corporate Member of Freie Universität Berlin and Humboldt-Universität zu Berlin, Institute of Biometry and Clinical Epidemiology, Charitéplatz 1, 10117, Berlin, Germany, Berlin, Deutschland, [4]Charité – Universitätsmedizin Berlin, corporate member of Freie Universität Berlin and Humboldt-Universität zu Berlin, Department of Radiology, Charitéplatz 1, 10117, Berlin, Germany., Berlin, Deutschland, [5]Berlin Institute of Health at Charité – Universitätsmedizin Berlin, BIH Biomedical Innovation Academy, BIH Charité Clinician Scientist Program, Charitéplatz 1, 10117, Berlin, Germany., Berlin, Deutschland, [6]Cluster of Excellence Matters of Activity. Image Space Material funded by the Deutsche Forschungsgemeinschaft (DFG, German Research Foundation) under Germany's Excellence Strategy – EXC 2025, Berlin, Deutschland

**Background:**
Tumor recurrence remains a major challenge after surgical treatment of hepatocellular carcinoma (HCC). Magnetic resonance elastography (MRE) enables non-invasive quantification of both liver and tumor shear stiffness, which have been evaluated as potential risk factors for HCC recurrence. However, their prognostic value for recurrence-free survival (RFS) has not been conclusively established. Our first of its kind meta-analysis aimed to determine the association of liver parenchymal and intratumoral shear stiffness measured by MRE with RFS in patients with HCC.

**Material and methods:**
A comprehensive search of MEDLINE was conducted up to August 2025. Studies reporting hazard ratios (HR) for liver and/or tumor shear stiffness in relation to RFS after curative-intent treatment of HCC were eligible. Data extraction and study selection were performed independently by two reviewers. Hazard ratios were pooled using a random-effects model with Hartung–Knapp adjustment and the restricted maximum likelihood (REML) estimator. Subgroup analyses were performed for recurrence pattern (intrahepatic vs. overall), and inclusion vs. exclusion of necrotic tumor areas. Risk of bias was assessed using the QUIPS tool. The review was conducted in accordance with PRISMA guidelines.

**Results:**
Eight studies comprising 989 patients were included. The overall recurrence rate was 39.6 ± 8.0%. Both higher liver parenchymal stiffness and intratumoral stiffness were significantly associated with shorter RFS, with a HR of 1.23 (95%CI 1.00, 1.51, p = 0.049) and a HR of 1.17 (95%CI: 1.08 to 1.28; p = 0.004)

respectively. However, substantial between-study heterogeneity was present. Risk of bias across studies was rated as low to moderate.

**Conclusion:**
Both increased liver stiffness and elevated intratumoral stiffness measured noninvasively by MRE are associated with shorter RFS of HCC after curative-intent hepatectomy. However, substantial heterogeneity and methodological differences limit firm conclusions. Further prospective studies are warranted to validate these findings.

DE GRUYTER BRILL

**Characteristics and Outcomes of Resected Pancreatic Invasive Mucinous Cystic Neoplasms**
ID: E-DCK-175

**Viszeralchirurgie / Visceral Surgery**
**Hepato-Pankreato-Biliäre Chirurgie (CALGP) / Hepato-Pancreato-Biliary Surgery (CALGP)**

Jannis Duhn[1], Lennart von Fritsch[1], Kim Honselmann[1], Louisa Bolm[1], Nina Bestmann[1], Kees Kleihues-van Tol[2], Maria Lacruz[3], Constanze Schneider[4], Fabian Reinwald[5], Andrea Sackmann[6], Bianca Franke[2], Bernd Holleczek[7], Anna Krauß[8], Steffen Deichmann[1], Thaer Abdalla[1], Monika Klinkhammer-Schalke[2], Sylke Zeissig[9 10], Tobias Keck[1], Ulrich Wellner[1], Rüdiger Braun[1]

[1]Department of Surgery, University Medical-Center Schleswig-Holstein, Campus Lübeck, Lübeck, Deutschland, [2]Network for Care, Quality and Research in Oncology, German Cancer Registry Group of the Association of German Tumor Centers (ADT), Berlin, Deutschland, [3]Cancer Registry Saxony-Anhalt, Halle, Deutschland, [4]Clinical-epidemiological Cancer Registry Berlin-Brandenburg, Berlin, Deutschland, [5]Cancer Registry of Rhineland-Palatinate in the Institute for Digital Health Data, Mainz, Deutschland, [6]Hessian Cancer Registry, Frankfurt am Main, Deutschland, [7]Cancer Registry Saarland, Saarbrücken, Deutschland, [8]Cancer Registry Mecklenburg-Western Pomerania, c/o Institute for Community Medicine, University Medicine Greifswald, Greifswald, Deutschland, [9]Institute of Clinical Epidemiology and Biometry (ICE-B), University of Würzburg, Würzburg, Deutschland, [10]Bavarian Cancer Registry, Bavarian Food and Health Safety Agency, Würzburg, Deutschland

**Background:**
Mucinous cystic neoplasms (MCN) are rare premalignant neoplasms of the pancreas, primarily occurring in middle-aged women. Due to likelihood of progression into invasive carcinoma (iMCN), European guidelines recommend surgical resection in cyst sizes >40mm, presence of symptoms e.g. jaundice or high-risk stigmata. However, the outcomes of patients with iMCN, and especially the impact of adjuvant chemotherapy after resection remains poorly defined.

**Material and methods:**
Data from patients within the pooled, pseudonymized dataset of the German Cancer Research Group of the Association of German Tumor Centers (GCRG/ADT) with pancreatic cancer (diagnosed between 2000 and 2023) were analyzed. Histological subtypes wereidentified by ICD-O3 histology codes, as pancreatic ductal adenocarcinoma (PDAC) (8500/3 or 8140/3) or iMCN (8470/3). Epidemiology, histopathology and survival rates were compared between surgically resected PDAC and iMCN. Prognostic impacts were assessed using uni- and multivariable regression analyses in R.

**Results:**
22235 patients diagnosed with PDAC and 116 with iMCN were included. iMCN patients were significantly younger (median age: 67 vs. 70y, $p<0.001$) and predominantly female (71.6 vs. 47.6%, $p<0.001$) compared to PDAC. Due to tumor localization in the pancreatic tail, iMCN patients received distal pancreatectomy more often (48.3 vs. 15.2%, $p<0.001$). Rates of adjuvant therapy were comparable (40.9 vs. 47.2%, $p=0.304$). Of resected specimen, iMCN showed less advanced tumor stages compared to PDAC (UICC IIB/III: 36.7 vs. 69.6%, $p<0.001$). iMCN histology was associated with improved overall survival (OS) (median OS: 22.1 vs. 17.3 months, $p<0.001$), but not proven as independent positive prognostic factor for OS in a multivariable analysis (HR 0.81, p=0.154). Presence of lymphatic invasion (HR 4.03, p=0.02) and undifferentiated Grading (G3/4; HR 3.03, p=0.006) were independent negative prognostic factors in iMCN patients. Interestingly, reception of adjuvant therapy was not associated with OS in iMCN patients (HR 0.92, p=0.739).

**Conclusion:**
We present a nationwide analysis, showing superior long-term outcomes after resection of iMCN compared to PDAC. This likely results from less advanced tumor stages at time of resection, as iMCN was not proven as prognostic factor after adjustment for confounding variables. This highlights the relevance of surveillance programs and early resection of MCN in the presence of high-risk stigmata. Interestingly, adjuvant therapy was not associated with survival in iMCN patients, warranting further studies.

DE GRUYTER BRILL

**Outcomes following Adjuvant Chemotherapy for Distal Bile Duct Carcinoma - A GCRG/ADT Analysis**
ID: E-DCK-176

**Viszeralchirurgie / Visceral Surgery**
**Hepato-Pankreato-Biliäre Chirurgie (CALGP) / Hepato-Pancreato-Biliary Surgery (CALGP)**

Jannis Duhn[1], Lennart von Fritsch[1], Kim Honselmann[1], Rüdiger Braun[1], Lars Peters[1], Kees Kleihues-van Tol[2], Maria Lacruz[3], Constanze Schneider[4], Fabian Reinwald[5], Andrea Sackmann[6], Bianca Franke[2], Bernd Holleczek[7], Anna Krauß[8], Steffen Deichmann[1], Thaer Abdalla[1], Monika Klinkhammer-Schalke[2], Sylke Zeissig[9 10], Tobias Keck[1], Ulrich Wellner[1], Louisa Bolm[1]

[1]Department of Surgery, University Medical-Center Schleswig-Holstein, Campus Lübeck, Lübeck, Deutschland, [2]Network for Care, Quality and Research in Oncology, German Cancer Registry Group of the Association of German Tumor Centers (ADT), Berlin, Deutschland, [3]Cancer Registry Saxony-Anhalt, Halle, Deutschland, [4]Clinical-epidemiological Cancer Registry Berlin-Brandenburg, Berlin, Deutschland, [5]Cancer Registry of Rhineland-Palatinate in the Institute for Digital Health Data, Mainz, Deutschland, [6]Hessian Cancer Registry, Frankfurt am Main, Deutschland, [7]Cancer Registry Saarland, Saarbrücken, Deutschland, [8]Cancer Registry Mecklenburg-Western Pomerania, c/o Institute for Community Medicine, University Medicine Greifswald, Greifswald, Deutschland, [9]Institute of Clinical Epidemiology and Biometry (ICE-B), University of Würzburg, Würzburg, Deutschland, [10]Bavarian Cancer Registry, Bavarian Food and Health Safety Agency, Würzburg, Deutschland

**Background:**
Distal Bile Duct Carcinomas (DBDC) are rare, yet highly malignant, neoplasms of the extrahepatic bile duct from the insertion of the cystic duct to the Ampulla of Vater. Currently, radical surgical resection by means of pancreatic head resection followed by adjuvant chemotherapy (AC) remains the only curative treatment option. However, previous studies showed conflicting results regarding the impact of AC in DBDC, warranting further studies. Here, we aim to delineate the association of AC with survival of DBDC patients in a nationwide cohort study.

**Material and methods:**
Due to lacking specific ICD-10 codes, patients with DBDC were identified from the pseudonymized dataset of the German Cancer Research Group of the Association of German Tumor Centers (GCRG/ADT) being diagnosed with extrahepatic BDC (ICD-10: C24.0; also encompassing perihilar carcinomas), receiving a pancreatic resection with or without AC. Patients receiving neoadjuvant therapy, radiotherapy and patients with 90-day postoperative mortality were excluded. A multiple imputation by chained equations (MICE) was performed to account for missing data. We analyzed prognostic factors, and outcomes following AC in subgroups using uni- and multivariable analyses in R.

**Results:**
We identified 736 patients receiving upfront surgical resection for DBDC, of which 150 (20.4%) received AC. Patients receiving AC were significantly younger (67 vs.72y, $p<0.001$), and showed more often lymph node metastases, higher grading and more lymphovascular invasion (each $p<0.05$). Capecitabine-based therapy was the most frequent regime (63.9%) followed by Gemcitabine (33.3%). Age ≥ 70y, advanced pT-status, lymph node metastases and advanced grading were independent negative prognostic factors for overall survival (OS). Overall, AC was not associated with improved OS (median OS: 26.9 vs. 24.9 months, p=0.072) compared to surgery alone, but was proven as independent positive prognostic factor after adjustment for confounding variables in a multivariable Cox regression (HR 0.76, p=0.03). Patients were stratified into subgroups according to certain histopathological stigmata. In patients with pT1-2 tumors, presence of lymph-node metastases or lymphovascular invasion, AC was associated with improved OS, but not in other subgroups.

**Conclusion:**

This study shows that AC is associated with improved survival in certain subgroups of DBDC patients only, thereby confirming previous studies observing less chemotherapy responsiveness in DBDC compared to perihilar or intrahepatic BDC. In this regard, careful selection of patients for AC after resection might be crucial, and prospective trials focusing on DBDC are needed, yet challenging due to the rarity of the tumor.

DE GRUYTER BRILL

**Comparison of radiologic and intraoperative assessment of vascular involvement in pancreatic cancer**
ID: E-DCK-177

**Viszeralchirurgie / Visceral Surgery**
**Hepato-Pankreato-Biliäre Chirurgie (CALGP) / Hepato-Pancreato-Biliary Surgery (CALGP)**

Andreas Andreou[1], Gala Nacul Mora[2], Benjamin Strücker[1], Haluk Morgül[1], Shadi Katou[1], Felix Becker[1], Andreas Pascher[1], Michael Köhler[2], Gesa Pöhler[2]

[1]University Hospital Münster, Department of General, Visceral and Transplant Surgery, Münster, Deutschland, [2]University Hospital Münster, Department of Radiology, Münster, Deutschland

**Background:**
Evaluation of vascular involvement during staging for pancreatic ductal adenocarcinoma (PDAC) is crucial for decision making regarding multimodal treatment strategy. The aim of this study was to compare the radiological vascular tumor involvement grading of the National Comprehensive Cancer Network (NCCN) with the binary intraoperative infiltration assessment during resection for PDAC.

**Material and methods:**
Our retrospective cohort study included 103 patients (mean age 68 years, male 61%) with CT-based diagnosis of PDAC undergoing primary resection between 2012 and 2023. Patients with borderline resectable or locally-advanced PDAC were excluded from the analysis. CT-based vascular tumor involvement was blinded classified following NCCN criteria by two abdominal radiologists. By discrepancies, the statement of the more experienced radiologist was chosen. Radiological vascular tumor involvement was defined as any contact vs. no contact and was compared to surgical findings using McNemar test, Spearman correlation, sensitivity (how well CT detects actual infiltration) and specificity (how well CT rules out infiltration when there is none). Subgroup analysis of CT interval to operation ≤4 weeks (n=83) vs. >4 weeks (n=20) was compared using ROC-Analysis.

**Results:**
Overall vascular tumor involvement was radiologically assessed to be higher than the intraoperative finding (radiological tumor involvement 51% vs. surgical tumor involvement 39%, p= 0.041). Arterial infiltration was interdisciplinary comparable (radiological 7% vs. surgical 5%, p= 0.625), but venous infiltration was radiologically higher than the intraoperative finding (radiological 44% vs. surgical 34%, p= 0.019). The overall interdisciplinary agreement was 83.5%, but systematic radiological overestimation of venous involvement was identified. Radiological arterial infiltration sensitivity was 99% and specificity 98%. Radiological venous infiltration sensitivity was 88% and specificity 81%. Furthermore, the patient subgroup with CT ≤4 weeks showed enhanced correlations (arterial AUC: 0.731, venous AUC: 0.888).

**Conclusion:**
This interdisciplinary study revealed the complexity of vascular tumor involvement evaluation in NCCN based grading, resulting in radiological overestimation of venous infiltration, which may lead to

DE GRUYTER BRILL

exaggerated recommendation for neoadjuvant therapy in interdisciplinary tumor boards. CT-surgery interval ≤4 weeks should be prioritized.

DE GRUYTER BRILL

**Preoperative bile duct stenting and biliary colonization in pancreatic surgery**
ID: E-DCK-213

**Viszeralchirurgie / Visceral Surgery**
**Hepato-Pankreato-Biliäre Chirurgie (CALGP) / Hepato-Pancreato-Biliary Surgery (CALGP)**

Jara Tigges[1], Marie-Christin Weber[1], Patrick Hofmockel[1], Nina Wantia[2], Helmut Friess[1], Ihsan Ekin Demir[1], Florian Scheufele[1]

[1]TUM University Hospital Rechts der Isar, Department of Surgery, München, Deutschland, [2]TUM University Hospital Rechts der Isar, Institute of Medical Microbiology, Immunology and Hygiene, München, Deutschland

**Background:**
Surgical procedures such as pancreaticoduodenectomy or total pancreatectomy remain associated with high morbidity and mortality. Recent studies have demonstrated that preoperative bile duct drainage (PBD) leads to a high rate of bacterial contamination of the bilary sytem. PBD was associated with alterations in the bilary microbiome and an increased risk of postoperative complications such as surgical site infections. This study aimed to generate an up-to-date status of the influence of PBD on bacterial bile duct contamination and postoperative outcome after pancreatic surgery.

**Material and methods:**
This retrospective single-centre study included 504 patients undergoing pancreaticoduodenectomy or total pancreatectomy between 2017 and 2024. Intraoperative bile duct swabs were analysed for microbial colonization and antibiotic resistance patterns according to standard clinical microbiology procedures. Data on PBD procedures, pre- and postoperative complications and clinical data were assessed. Patients with PBD routinely received Piperacillin/Tazobactam for at least 5 days postoperatively as per in-house standard operating procedures.

**Results:**
Bacterial bile duct contamination was significantly more frequent in patients with PBD (n=236) than in those without (n=268) (97% vs 18.3%; p<0.001). Patients with PBD also showed a higher mean number of bacterial species (mean 3.7; s.d.=1.6 vs. mean 1.61; s.d.=1.04; p<0.001). Major postoperative complications (Clavien-Dindo ≥ III) occured in 26.8% of patients, without significant differences between those with or without positive cultures. Fascial dehiscence was significantly more common in culture-positive patients (2.4% vs. 20.3%; p=0.008). Resistance to Ampicillin/Sulbactam (5.6% va. 64%; p<0.001), Piperacillin/Tazobactam (1.5% va. 30.1%; p<0.001), and Ciprofloxacin (25% vs. 78%; p<0.001) was significantly higher in PBD patients, while the combination of Meropenem and Linezolid remained a therapeutic option (0% va. 1.3%; p=0.151).

**Conclusion:**
This study highlighted the strong association between PBD and microbiological biliary colonization. Which was associated with a prominent increase in antibiotic resistance of bacteria in the bile fluid. Bacterial contamination of the bile duct was an indipendet predictor of fascial dehiscense, while other complications remained unaffected. This finding may be explained by a progressively adapted postoperative antibiotic strategy based on previous results, with PBD patients routinely receiving prolonged perioperative therapy with Piperacillin/Tazobactam.

DE GRUYTER BRILL

**Major Liver Resection in Cirrhotic HCC patients in the era of robotic surgery: Expanding Boundaries**
ID: E-DCK-231

**Viszeralchirurgie / Visceral Surgery**
**Hepato-Pankreato-Biliäre Chirurgie (CALGP) / Hepato-Pancreato-Biliary Surgery (CALGP)**

Patryk Kambakamba[1], Viktoria Schmuck-Schleper[1], Maximilian Wenz[1], Christoph Anthoni[1], Stefan A. Farkas[1]

[1]St. Josefs Hospital, Department of Surgery, Wiesbaden, Deutschland

**Background:**
Cirrhosis and has traditionally been considered relative contraindications for hepatic resection due to the risk of post-hepatectomy liver failure (PHLF). With the rising incidence of HCC and persistent organ shortage, alternative curative approaches are urgently required. Robotic surgery may provide improved outcomes, less invasiveness, and be particularly beneficial for cirrhotic patients. The aim of this study is, to evaluate the safety and outcomes of robotic liver resection in cirrhotic patients with hepatocellular carcinoma (HCC).

**Material and methods:**
We retrospectively analyzed all cirrhotic patients with HCC undergoing liver resection between 2020–2025. Patients were stratified by approach (open, laparoscopic, robotic). Outcomes included perioperative results, PHLF (ISGLS), morbidity, mortality, and oncologic feasibility.

**Results:**
Fifty-seven (n=57) cirrhotic patients underwent liver resection for HCC: open (n=28), laparoscopic (n=7), and robotic (n=21). Whereas initially major resections accounted only for 17% (n=6/35) of open and laparoscopic resections, the initiation of the robotic program significantly increased the rate of major resections up to 43% (n=9/21). Despite a learning curve, overall median operative time was comparable between open and robotic resections (242 vs. 252 minutes, p=0.64), whereas robotic procedures significantly reduced operation time compared with equivalent minor laparoscopic resections from 214 to 198 minutes (p=0.03). With proportional increase of major liver resections, a trend to bigger tumor size and frameshift from BCLC 0 to BCLC A patients was observed for the robotic group. In that context, PHLF occurred in n=3/6 robotic major resections (all ISGLS grade A, resolved with supportive care) versus n=2/9 severe grade C cases after open surgery. Despite more complex resections, robotic patients had shorter ICU stay (1 vs. 3 days, p=0.01), reduced hospital stay (7 vs. 11 days, p=0.02), and lower ascites/aldactone requirements. All robotic procedures were technically feasible, with no conversions. R0 rates were comparable (98% vs. 96%, p=0.89). No 90-day mortality occurred.

**Conclusion:**
Robotic liver resection is safe and feasible in cirrhotic HCC patients, even for major hepatectomies. Although mild PHLF was common, it is reversible, and recovery outcomes are favorable. The improved recovery from liver dysfunction ín this high risk population, may be partially attributed to the less traumatic benefits of robotic surgery. Therfore, in the era of increasing HCC incidence and organ shortage, robotic surgery can expand resectional options and complement transplantation strategies.

DE GRUYTER BRILL

### Preoperative Semaglutide Before Robotic Major Liver Resection in Obese patients– Preliminary Results

ID: E-DCK-232

**Viszeralchirurgie / Visceral Surgery**
**Hepato-Pankreato-Biliäre Chirurgie (CALGP) / Hepato-Pancreato-Biliary Surgery (CALGP)**

Patryk Kambakamba[1], Maximilian Wenz[1], Nora Hagenmüller[1], Schäfers Ole[1], Christoph Anthoni[1], Stefan A. Farkas[1]

[1]St. Josefs Hospital, Department of Surgery, Wiesbaden, Deutschland

**Background:**
Obesity and hepatic steatosis are increasingly prevalent in hepatopancreatobiliary (HPB) surgery and are associated with prolonged operative time and higher morbidity. In minimally invasive major hepatectomy, these factors can further increase technical complexity. Pharmacological weight loss with GLP-1 receptor agonists may optimize surgical candidates, but data in major HPB surgery remain limited. Moreover, preliminary translational studies in rodents, support a potential role of GLP-1 in liver regeneration. The aim of this pilot study, is to evaluate the impact of preoperative semaglutide (GLP-1) therapy on weight loss, hepatic steatosis, and perioperative outcomes in obese patients undergoing robotic major liver resection.

**Material and methods:**
All consecutive high-BMI patients (median BMI 38 kg/m²) scheduled for robotic major liver resection between February and September 2025 received preoperative semaglutide for a median of 4 weeks. Endpoints included percentage weight loss, MRI-documented steatosis (before and after GLP-1), operative time, postoperative morbidity (Clavien–Dindo ≥III), and feasibility.

**Results:**
Ten patients were included and compliance with GLP-1 was >100%. Median preoperative weight loss was 7.2% (IQR 4.1–10%), corresponding to 11.4 kg. All patients showed signs of steatosis in MRI, with a major proportion of severe steatotic patients (n=6). After GLP-1 repeated MRI prior to surgery documented a descrease of steatosis in n=5/10 patients. n=7 patients underwent double vein embolization prior to hepatectomy and GLP-1 treatment was started after the intervention. Postinterventional GLP-1 treatment did not impair liver regeneration and completion hepatectomy could be performed in all patients. Indications included hepatocellular carcinoma (n=5), colorectal liver metastases (n=3), and cholangiocarcinoma (n=2). Overall, Robotic right hemihepatectomy was performed in n=8 patients, left hemihepatectomy in n=1, and parenchyma-sparing resection (≥3 Segments) in n=1. No conversions occurred. Median operative time was 210 min (IQR 180–330). Only one patient experienced a major complication (Clavien–Dindo ≥IIIa).

**Conclusion:**
Preoperative GLP-1 therapy is feasible, safe, and well tolerated in obese patients undergoing robotic major liver resection. It induces clinically relevant weight loss, reduces hepatic steatosis, and facilitates technically demanding procedures without impairing liver regeneration. Larger studies are warranted to confirm these preliminary findings and to further define the role of GLP-1 receptor agonists in optimizing surgical candidates.

DE GRUYTER BRILL

**The impact of cardiac comorbidities on postoperative outcome in pancreatic head resections**
ID: E-DCK-249

**Viszeralchirurgie / Visceral Surgery**
**Hepato-Pankreato-Biliäre Chirurgie (CALGP) / Hepato-Pancreato-Biliary Surgery (CALGP)**

Patricia Wyzlic[1], Isabel Kraemer[1], Nikolai Schleussner[1], Naita Wirsik[1], Karl Knipper[1], Jin-On Jung[1], Lars Schiffmann[1], Hans Fuchs[1], Christiane J. Bruns[1], Alexander Damanakis[1], Thomas Schmidt[1]

[1]Uniklinikum Köln, Köln, Deutschland

**Background:**
Patients undergoing major surgery for pancreatic disease are older and often present with pre-existing medical conditions. Our aim was to assess the potential influence of cardiac comorbidities on postoperative morbidity in patients undergoing pancreatic head resections to estimate the perioperative risk in this group of patients.

**Material and methods:**
We included 417 patients who underwent pancreatic head resections from 2016-2024 at the university hospital in Cologne and had well documented pre-existing cardiac conditions. Included cardiac comorbidities and risk factors were a body mass index > 25 kg/m2, arterial hypertension, congestive heart failure, heart valve dysfunction or replacement, cardiac arrhythmia, cardiac stenting, a myocardial infarction in the past and the presence of a pacemaker.

**Results:**
Out of all 417 included patients, 43% were female and 57% were male. Median age at surgery was 68 years. The vast majority had relevant comorbidities, which is reflected in the ASA score: 48% were classified ASA 2 and 47% were classified ASA 3. Only 25% of all included patients had no cardiac risk factors or comorbidities. 36% had one risk factor and 28% had two risk factors. For further analysis, we stratified two cohorts, 106 patients having no cardiac comorbidity and 311 patients having cardiac comorbidities. In patients with at least one cardiac comorbidity significantly more wound infections (13% vs. 23%, p=0.025), more sepsis (2% vs. 7.4%, p=0.039) and more episodes of postoperative tachyarrhythmia absoluta (2% vs. 7.7%, p=0.032) occured. Overall Clavien-Dindo Score was significantly higher in patients with at least one pre-existing cardiac condition (Clavien -Dindo 3b-4b: 8.5 % vs. 20%, Clavien-Dindo 5: 2.8% vs. 4.5%, p=0.015) and the overall hospital stay of those patients was significantly longer (16 vs. 21 days, p<0.001). In logistic regression analysis, an increased number of pre-existing cardiac comorbidities, led only to significant higher rates of postoperative tachyarrhythmia absoluta (OR 1.58, 95% CI 1.17-2.12, p=0.002) and wound infections (OR 1.28, 95% CI 1.05-1.57, p=0.016).

**Conclusion:**
In conclusion, already one assessed cardiac risk factor or comorbidity significantly elevates postoperative complication rates with, amongst others, a significantly higher Clavien-Dindo Score. Increasing prevalence of cardiac risk factors does not seem to lead to worse outcomes. Patients should be informed about the higher postoperative risk and perioperative management should be ameliorated in order to provide the best standard-of-care possible in this high-risk profile group of patients.

DE GRUYTER BRILL

**A retrospective cohort analysis of 102 patients who underwent ALLPS**
ID: E-DCK-250

**Viszeralchirurgie / Visceral Surgery**
**Hepato-Pankreato-Biliäre Chirurgie (CALGP) / Hepato-Pancreato-Biliary Surgery (CALGP)**

Katrin Zimmermann[1], Mohammad Fard-Aghaie[1], Amitis Pourian[1], Tim Reese[2], Jörg Böcker[1], Kim Caroline Wagner[1], Karl Oldhafer[1]

[1]Asklepios Klinik Barmbek, Viszeralchirurgie, Hamburg, Deutschland, [2]Karolinska Universitätskrankenhaus, Division of Hepatobiliary- and Pancreatic Surgery, Solna, Sweden

**Background:**
Associating Liver Partition and Portal Vein Ligation for Staged Hepatectomy (ALPPS) was first described in 2012 and has since been increasingly adopted as a strategy to expand resectability in advanced hepatic malignancies. Concerns regarding early morbidity and mortality prompted refinement of both technique and patient selection. We report one of the largest single-center series worldwide, analyzing more than 100 consecutive ALPPS procedures performed at a high-volume German HPB unit.

**Material and methods:**
A retrospective cohort analysis was conducted including all patients who underwent ALPPS at our institution over a period of more than ten years. Demographics, comorbidities, tumor entity, ALPPS variant (classic, partial, hybrid, RFA), volumetric and functional liver parameters, perioperative morbidity (Clavien–Dindo), 90-day mortality, and survival were assessed. Cox regression models explored the association of standardized future liver remnant (sFLR) with survival. Outcomes before and after the implementation of stricter selection criteria at a consensus meeting were compared.

**Results:**
The cohort comprised 102 patients (74% male, mean age 66 years). Colorectal liver metastases represented the leading indication (≈70%), followed by cholangiocarcinoma, hepatocellular carcinoma, and non-colorectal non-neuroendocrine tumors. The median interval between the two stages was approximately three weeks, with an average relative sFLR increase of more than 70%. Classic ALPPS accounted for 45% of cases, partial ALPPS for 34%, and hybrid procedures for 21%.

After stage 1, more than half of patients remained complication-free, while post-hepatectomy liver failure occurred in about 16%. After stage 2, only 15% remained free of complications, with major events (≥IIIa) occurring in over a quarter of patients. The most frequent complications were renal dysfunction, hemorrhage, and infections. Overall 90-day mortality was below 9%, with lower rates after implementation of stricter selection (<5%).

Volumetric analyses did not show a linear correlation with survival. Hazard ratio modeling suggested optimal survival within defined sFLR ranges rather than at very low or excessively high values. Long-term survival varied substantially by tumor entity: biliary tumors showed the poorest outcomes, whereas non-colorectal non-neuroendocrine tumors demonstrated a more favorable trend.

DE GRUYTER BRILL

**Conclusion:**
This large single-center experience confirms that ALPPS can be performed with acceptable early mortality when delivered in a specialized unit, even in older and comorbid patients. Functional testing (LiMAx, ICG) appears essential alongside volumetry for appropriate selection. The high incidence of renal dysfunction following stage 2 underscores the physiologic burden of two major operations in close succession and highlights the need for perioperative nephroprotection. While stricter indications have improved perioperative safety, long-term results remain driven by tumor biology.
ALPPS is a valid strategy to expand curative resection in advanced hepatic tumors, particularly colorectal liver metastases, when performed in experienced centers. This series, among the largest reported from a single institution, underscores the importance of refined selection, functional assessment, and complication prevention to optimize outcomes.

DE GRUYTER BRILL

**ICG Fluorescence with 4K overlay advances laparoscopic liver resection: a propensity score study**
ID: E-DCK-264

**Viszeralchirurgie / Visceral Surgery**
**Hepato-Pankreato-Biliäre Chirurgie (CALGP) / Hepato-Pancreato-Biliary Surgery (CALGP)**

Laura-Ann Sahan[1], Omid Ghamar Nejad[1], Hamraz Javaheri[2], Mahmoud Jabal[1], Rizky Widyaningsih[1], Gregor Alexander Stavrou[1]

[1]Klinikum Saarbrücken, Department of General, Visceral, Thoracic and Oncologic Surgery, Saarbrücken, Deutschland, [2]Department of Embedded Intelligence, German Research Center for Artifcial Intelligence (DFKI), Kaiserslautern, Deutschland

**Background:**
Laparoscopic liver resection (LLR) has become a standard approach for the treatment of liver malignancies, offering significant perioperative advantages over conventional open surgery. Nevertheless, traditional laparoscopic liver resection (C-LLR) remains technically challenging due to the lack of tactile feedback, limited depth perception, and reliance on intermittent intraoperative ultrasound, all of which can compromise accurate tumor localization and resection margins. Real-time navigation techniques such as indocyanine green (ICG) fluorescence imaging enable intraoperative visualization of tumors and biliary structures. When combined with advanced 4K imaging technology, these methods have the potential to enhance surgical navigation and precision. In this context, the Liver-Light study evaluated the feasibility and clinical benefits of integrating ICG fluorescence with 4K overlay (ICG4K-LLR) to improve surgical accuracy as well as postoperative surgical and oncologic outcomes compared to conventional techniques.

**Material and methods:**
This single-center prospective study included a total of 113 patients who underwent laparoscopic liver resection between 2017 and 2024. Of these, 42 patients received ICG fluorescence-guided 4K overlay navigation (ICG4K-LLR) between 2021 and 2024, while 71 patients underwent conventional laparoscopic liver resection (C-LLR). Patients were matched 1:1 using propensity scores with a control group from a retrospective cohort who underwent C-LLR. Matching factors included age, sex, BMI, tumor type, and procedural complexity. Perioperative and short-term oncological outcomes were analyzed after matching.

**Results:**
ICG fluorescence with 4K overlay in LLR significantly reduced operative time (155.2 min vs. 196.6 min, p=0.002) and intraoperative blood loss (300.0 ml vs. 501.2 ml, p=0.006), with a markedly lower rate of blood transfusions (0 % vs. 14.3 %, p = 0.011). Importantly, no positive resection margins were observed in the ICG4K-LLR group, compared to 14.3 % in the control group (p = 0.011). Clinically relevant complications like major complications and posthepatectomy liver failure were significantly less frequent with ICG4K-LLR (major morbidity: 7.1 % vs. 23.8 %, p = 0.035; PHLF grade B/C: 0 % vs. 9.5 %, p = 0.040;). The 1-year disease-free survival rate was substantially higher in the ICG4K-LLR group (92.3 % vs. 64.3 %, p = 0.004). Benefits were particularly pronounced for complex resections, where both overall and disease-free survival were significantly improved.

**Conclusion:**
ICG fluorescence navigation with 4K overlay represents a powerful advancement in laparoscopic liver surgery. By enabling continuous, high-resolution, real-time tumor tracking, it helps overcome key limitations of conventional laparoscopy, leading to shorter procedures, reduced blood loss, improved margin status, and superior short-term oncological outcomes. These findings support the broader adoption of ICG4K technology in minimally invasive liver surgery and justify further evaluation in larger, randomized trials.

DE GRUYTER BRILL

**Perioperative transfusion and the effect on postoperative morbidity in pancreatic head resections**
ID: E-DCK-276

**Viszeralchirurgie / Visceral Surgery**
**Hepato-Pankreato-Biliäre Chirurgie (CALGP) / Hepato-Pancreato-Biliary Surgery (CALGP)**

Isabel Kraemer[1], Patricia Wyzlic[1], Saeed Torabi[2], Nikolai Schleußner[1], Naita Wirsik[1], Karl Knipper[1], Jin-On Jung[1], Lars Schiffmann[1], Hans Fuchs[1], Christiane Bruns[1], Alexander Damanakis[1], Thomas Schmidt[1]

[1]Universitätsklinikum Köln, Klinik für Allgemein-, Viszeral-, Thorax- und Transplantationschirurgie, Köln, Deutschland, [2]Universitätsklinikum Köln, Klinik für Anästhesiologie und Operative Intensivmedizin, Köln, Deutschland

**Background:**
Perioperative transfusions of blood products are associated with poorer postoperative outcome as prior data indicates. Given the high-risk profile of pancreatic surgery, our aim was to assess postoperative morbidity after pancreatic head resections regarding the administration of blood products.

**Material and methods:**
We performed a retrospective analysis of 269 patients with well annotated clinical information including anesthesia protocols who underwent pancreatic head resections between 2016 and 2024 at the university hospital of Cologne. Clinical pre- and postoperative parameters, including postoperative complications, in-hospital mortality and reoperations were assessed.

**Results:**
Out of all included patients, 32% received at least one packed red blood cell concentrate (PRBC) during their hospital stay. Intraoperative transfusions were given in 11% of cases and 25% of patients received more than two PRBC transfusions in the overall hospital stay. Perioperative transfusions correlate significantly with postoperative complications like intraabdominal abscesses and subsequent CT-guided drainage (17% vs. 3.3% for intraabdominal abscesses, p=<0.001; 14% vs. 4.4% for CT-guided drainage, p=0.006).
Intraoperative transfusions alone correlate with a significantly higher Clavien Dindo score in the univariable analysis (p=0.008; OR 1.89, CI 1.15, 3.02) and multivariable analysis (p=0.045; OR 2.3, CI 1.02, 5.40). However, higher intraoperative blood loss (>500ml) or lower intraoperative haemoglobin levels were not associated with higher postoperative morbidity and mortality.

**Conclusion:**
Intraoperative PRBC transfusion during pancreatic head resections is associated with specific postoperative complications like pancreatic fistulae, intraabdominal abscesses and a higher Clavien Dindo score, independent of intraoperative blood loss or low intraoperative haemoglobin-levels. In case of intraoperative transfusions, patients remain in the hospital for 7 days longer, on average.
Potentially, further research is needed to evaluate the impact of interdisciplinary collaboration between anaesthesia and surgery on decision-making regarding intraoperative transfusion.

DE GRUYTER BRILL

### Emergency Salvage Pancreatectomy following Pancreatic Head Resection Single Center Experience

ID: E-DCK-283

**Viszeralchirurgie / Visceral Surgery**
**Hepato-Pankreato-Biliäre Chirurgie (CALGP) / Hepato-Pancreato-Biliary Surgery (CALGP)**

Simon-Alexander Kammer[1], Andreas Andreou[1], Felix Becker[1], Shadi Katou[1], Michael Bartels[2], Andreas Pascher[1], Benjamin Strücker[1], Haluk Morgül[1]

[1]Universitätsklinik Münster, Klinik für Allgemein-, Viszeral- und Transplantationschirurgie, Münster, Deutschland, [2]Helios Park-Klinikum Leipzig, Klinik für Allgemein, - Viszeral- und Thoraxchirurgie, Leipzig, Deutschland

**Background:**
Grade C pancreatic fistula following pancreatic head resection is associated with significant morbidity and mortality rates. The timing of intervention is critical; however, management currently relies on clinical judgment, as no standardized, evidence-based work-up exists. In this report, we present a series of salvage pancreatectomies performed for the treatment of this severe complication.

**Material and methods:**
We retrospectively analyzed patients who underwent pancreatectomy at our tertiary HPB center between 2012 and 2024. Patients were stratified according to the need for and timing of salvage pancreatectomy. Factors that might influence complications as well as short-term morbidity and mortality were analyzed.

**Results:**
During this period, 234 pancreatectomies were performed. Twenty patients who underwent emergency salvage pancreatectomy due to peritonitis or hemorrhage induced by grade C fistulas were included in the study. The 90 days mortality rate was 25%. The median overall hospital and ICU stays were 41 and 34 days, respectively. Timing of revision was a significant factor for mortality (5 days vs. 10 days, $p<0.05$)

**Conclusion:**
Our data demonstrate lower mortality than published data following salvage pancreatectomy; however, the indication and timing remain crucial. Although emergency salvage pancreatectomy is a highly demanding procedure, it can be considered a damage control procedure in experienced centers in the early postoperative phase.

DE GRUYTER BRILL

**PAncreatic Surgery and Length Of Hospital Stay - A Systematic Review and Meta-Analysis (PALOS study)**
ID: E-DCK-298

**Viszeralchirurgie / Visceral Surgery**
**Hepato-Pankreato-Biliäre Chirurgie (CALGP) / Hepato-Pancreato-Biliary Surgery (CALGP)**

Laetitia Hampe[1], Klaudia Narbekovas[2], Amila Cizmic[1], Anas Preukschas[1], Anna Nießen[1], Jan Bardenhagen[1], Faik Uzunoglu[1], Chenchen Li[1], Ulla Klaiber[2], Oliver Strobel[2], Pascal Probst[3], Thilo Hackert[1], Felix Nickel[1], Carl-Stephan Leonhardt[2]

[1]University Medical Center Hamburg-Eppendorf, Department of General, Visceral and Thoracic Surgery, Hamburg, Deutschland, [2]Medical University of Vienna, Department of General Surgery, Wien, Österreich, [3]Cantonal Hospital Thurgau, Department of Surgery, Frauenfeld, Schweiz

**Background:**
Length of hospital stay (LOS) is a commonly reported postoperative outcome measure in pancreatic surgery. LOS has also been proposed as an element of composite outcome measures, textbook outcome and benchmarking. However, LOS may be influenced by a wide variety of factors, including healthcare systems, cultural traditions, and readmission rate. This systematic review and meta-analysis aimed to investigate LOS in pancreatic surgery.

**Material and methods:**
A systematic review and meta-analysis was conducted following the PRISMA guidelines to identify all randomized controlled trials (RCTs) of pancreatic surgery and reporting LOS. Medians were converted to means for the meta-analysis according to standard methodology. Subsequently, a random-effects model using mean differences as effect estimator for the continuous outcome LOS was conducted. The protocol was preregistered at PROSPERO (CRD42024601296).

**Results:**
A total of 183 RCTs, published between 1994 and 2024, met the inclusion criteria, comprising a total of 27,585 patients. The mean LOS differed significantly by type of pancreatic resection ($p = 0.0019$), with studies on pancreaticoduodenectomy (PD) reporting a mean LOS of 20 days (95% CI: 18 to 21) and studies on distal pancreatectomy (DP) reporting a mean LOS of 13 days (95% CI: 11 to 16). LOS also varied by country ($p<0.0001$) and country remained a significant factor after adjusting for readmission rate, year of study, and type of pancreatic resection. The shortest LOS was observed in the USA, with a pooled value of 11 days (95% CI: 10 to 13) and the longest in Japan, with a pooled value of 30 days (95% CI: 26 to 34). Subgroup analysis of studies comparing minimally invasive and open surgery showed a mean LOS difference of 2 days (95% CI: 1 to 3). However, this effect varied significantly across countries, with mean LOS differences ranging from 1 to 6 days.

**Conclusion:**
LOS after pancreatic surgery exhibited significant statistical and clinical heterogeneity based on the country of study, type of operation and access. This highlights the importance of considering contextual factors when evaluating and comparing LOS as an outcome measure in pancreatic surgery and incorporating it into composite scores.

DE GRUYTER BRILL

**Proteomic Profiling of CRLM reveals Response-Specific Molecular Signatures of Chemotherapy Efficacy**
ID: E-DCK-302

**Viszeralchirurgie / Visceral Surgery**
**Hepato-Pankreato-Biliäre Chirurgie (CALGP) / Hepato-Pancreato-Biliary Surgery (CALGP)**

Agnes Böhm[1], Lisa Skrip[1], Oliver Klein[2], Felix Strobl[3], Jonas Wieland[1], Alexander Arnold[4], Yijun Zhou[1], Björn Papke[4], Christine Sers[4], Dominik Paul Modest[5], Simon Moosburner[1 6], Philipp Haber[1], Felix Krenzien[1 6], Nathanael Raschzok[1], Wenzel Schöning[1], David Horst[4], Thomas Malinka[1], Ingolf Sack[7 8], Johann Pratschke[1 8], Igor Maximilian Sauer[1 8], Karl Hillebrandt[1 6]

[1]Charité – Universitätsmedizin Berlin, corporate member of Freie Universität Berlin and Humboldt-Universität zu Berlin, Department of Surgery, Experimental Surgery, Augustenburger Platz 1, Berlin, Deutschland, [2]Berlin Institute of Health at Charité – Universitätsmedizin Berlin, Core Facility Imaging Mass Spectrometry, Berlin, Deutschland, [3]Charité – Universitätsmedizin Berlin, corporate member of Freie Universität Berlin and Humboldt-Universität zu Berlin, Department of Vascular Surgery, Hindenburgdamm 30, Berlin, Deutschland, [4]Institute of Pathology, Charité – Universitätsmedizin Berlin, Corporate Member of Freie Universität Berlin and Humboldt-Universität zu Berlin, Chariteplatz 1, berlin, Deutschland, [5]Charité – Universitätsmedizin Berlin, Corporate Member of Freie Universität Berlin and Humboldt- Universität zu Berlin, Department of Hematology, Oncology, and Cancer Immunology (CVK/CCM), Charitéplatz 1, Berlin, Deutschland, [6]Berlin Institute of Health at Charité – Universitätsmedizin Berlin, BIH Biomedical Innovation Academy, BIH Charité Clinician Scientist Program, Charitéplatz 1, Berlin, Deutschland, [7]Charité – Universitätsmedizin Berlin, corporate member of Freie Universität Berlin and Humboldt-Universität zu Berlin, Department of Radiology, Charitéplatz 1, 10117, Berlin, Germany., Berlin, Deutschland, [8]Cluster of Excellence Matters of Activity. Image Space Material funded by the Deutsche Forschungsgemeinschaft (DFG, German Research Foundation) under Germany's Excellence Strategy – EXC 2025, Berlin, Deutschland

**Background:**
Chemoresistance in treatment of colorectal liver metastases (CRLM) poses a major challenge in preventing disease relapse, with up to 80% of patients developing drug resistance over the course of treatment. Proteomic signatures of responsive vs. non-responsive metastases can provide insights into functional expression patterns, potentially identifying biomarkers for therapy efficacy.

**Material and methods:**
A total of 33 CRLM tissue samples from 31 patients were subjected to histopathological and proteomic analysis. The patients were included in the study after undergoing preoperative treatment (n = 28), including platinum-based chemotherapy (n = 19), non-platinum-based chemotherapy (n = 8), as well as targeted therapies (n = 20), or without preoperative therapy (n = 5). Based on Rubbia-Brandt criteria and vital tumor cell percentage, CRLM were categorized to major (MR), partial (PR) and no response (NR) groups. Proteomic analysis was conducted using label-free mass spectrometry (LFQ-MS), followed by clustering according to histological response and distinct treatment regimens.

**Results**
Proteomic analysis revealed significant differential protein expression of 607 proteins linked to distinct histopathological response types. CRLM responsive to chemotherapy displayed marked enrichment ($p \leq 0.01$) in pathways associated with immune infiltration, ECM matrix organization, the complement system, and apolipoprotein-associated processes, indicative of distinct stromal and immune invasion patterns with multimodal importance of cell adhesion proteins. In contrast, attenuated expression of proteins enriched in pathways of mitochondrial translation initiation, elongation and termination was detectable.

**Conclusion:**
CRLM exhibited distinct proteomic phenotypes based on their histopathological response to preoperative systemic therapy largely independent of chemotherapy regimens. This proteomic profiling establishes a foundation for identifying critical biomaker profiles by nominating protein markers for major chemotherapy response.

DE GRUYTER BRILL

### Simultaneous Resection of Colorectal Liver Metastases and Extrahepatic Abdominal Disease
ID: E-DCK-333

**Viszeralchirurgie / Visceral Surgery**
**Hepato-Pankreato-Biliäre Chirurgie (CALGP) / Hepato-Pancreato-Biliary Surgery (CALGP)**

Janine Baumgart[1]

[1]Universitätsmedizin Mainz, Allgemein-, Viszeral- und Transplantationsmedizin, Mainz, Deutschland

**Background:**
The aim of the study was to describe the perioperative and oncological outcome of patients who underwent simultaneous resection of colorectal liver metastases (CRLM) and concomitant extrahepatic abdominal disease (EHAD).

**Material and methods:**
Data of 1312 patients undergoing liver surgery due to CRLM from January 2008 to December 2024 were analysed from our prospective liver data base. Analysis included patients' demographics, perioperative and oncological outcome.

**Results:**
Combined resection of CRLM and EHAD was performed in 147 patients. EHAD was one-sited in 92 and multiple sited in 55 patients. EHAD tumour invasion was detected in 74 patients (50.3%) and in 97 resected specimens. Median RFS was superior in patients without tumour invasion in the EHAD (8.8 versus 5.6 months, p = 0.007). Positive distant lymph node invasion had worse median RFS (4.1 versus 7.4 months, p = 0.001). The median Overall survival (OS) was 39.5 months. One sited EHAD with tumour infiltration achieved a median OS of 48.8 and multiple sited EHAD with tumour infiltration 29.9 months (p=0.005).

**Conclusion:**
Resection of CRLM and EHAD can achieve prolonged survival in well selected patients. Only in cases of distant lymph node, peritoneum involvement and multiple sited EHAD, the benefit of surgery remains debatable.

DE GRUYTER BRILL

### Malignant biology drives short term outcomes following pancreatic resection in patients with IPMNs

ID: E-DCK-336

**Viszeralchirurgie / Visceral Surgery**
**Hepato-Pankreato-Biliäre Chirurgie (CALGP) / Hepato-Pancreato-Biliary Surgery (CALGP)**

Athanasios Zamparas[1], Ughur Aghamaliyev[1], Yannick Meyer[1], Simon Sirtl[2], Georg Beyer[2], Markus Guba[1], Hanno Niess[1], Jens Werner[1], Bernhard Renz[1]

[1]LMU Klinikum, Allgmein, Viszeral & Transplantationschirurgie, München, Deutschland, [2]LMU Klinikum, Innere Medizin, München, Deutschland

**Background:**
IPMNs can serve as precursors for pancreatic ductal adenocarcinoma (PDAC), with IPMN-derived carcinomas (IPMN-Ca) representing a malignant progression. While poorer long-term survival in IPMN-Ca compared to non-invasive IPMNs is expected given their malignant nature, data comparing short-term postoperative outcomes between these two entities remain limited. This study compares postoperative outcomes between IPMN and IPMN-Ca, applying the Ideal Outcome (IO) metric for the first time in this context.

**Material and methods:**
We analyzed 148 patients from our prospective database undergoing pancreatoduodenectomy (PD) or distal pancreatectomy (DP) for IPMN or IPMN-Ca from 2014 to 2024. Pre-, intra-, and postoperative variables were compared. IO was defined as the absence of six adverse events: in-hospital mortality, major complications (Clavien-Dindo ≥ III), postoperative pancreatic fistula (POPF) grade B/C, reoperation, prolonged length of stay (≥75th percentile), and 30-day readmission.

**Results:**
IPMN-Ca patients showed higher CA 19-9 and lower albumin and hemoglobin levels, reflecting a systemic malignant burden. POPF B/C was more frequent in IPMN (25% vs. 13%, p=0.04), while delayed gastric emptying (DGE) B/C and major complications were higher in IPMN-Ca. Mortality was higher in IPMN-Ca (4% vs. <1%, p=0.03). IO was achieved in 55% of cases (56% IPMN vs. 52% IPMN-Ca, p=0.3). DP had better outcomes than PD, particularly in IPMN. Multivariate analysis identified ASA ≥3, PD, and operative time ≥280 min as negative predictors of IO in IPMN, with ischemic heart disease as the sole predictor in IPMN-Ca.

**Conclusion:**
Although IO rates were similar, IPMN and IPMN-Ca differ in complication profiles. The IO metric offers a valuable tool for benchmarking outcomes and guiding perioperative strategies.

DE GRUYTER BRILL

**Switching Strategies in Neoadjuvant Chemotherapy for Pancreatic Adenocarcinoma**
ID: E-DCK-381

**Viszeralchirurgie / Visceral Surgery**
**Hepato-Pankreato-Biliäre Chirurgie (CALGP) / Hepato-Pancreato-Biliary Surgery (CALGP)**

Veronika Elisabeth Lummer[1], Florian Oehme[1 2], Anke Rentsch[3], Sebastian Hempel[1 3], Marius Distler[1 3], Jürgen Weitz[1 3], Olga Radulova-Mauersberger[1]

[1]Department of Visceral-, Thoracic and Vascular Surgery, University Hospital Carl Gustav Carus, Technische Universität Dresden, 01307 Dresden, Germany., Dresden, Deutschland, [2]National Center for Tumor Diseases (NCT/UCC), Faculty of Medicine and University Hospital Carl Gustav Carus, Technische Universität Dresden; German Cancer Research Center (DKFZ), Helmholtz-Zentrum Dresden-Rossendorf (HZDR), 01307 Dresden, Germany., Dresden, Deutschland, [3]Universitäts KrebsCentrum (NCT/UCC), Klinisches Krebsregister, Universitätsklinikum Carl Gustav Carus, Dresden., Dresden, Deutschland

**Background:**
Neoadjuvant chemotherapy (NAC) has become a standard of care in the treatment of borderline resectable and locally advanced pancreatic adenocarcinoma (PDAC), as it improves R0 resection rates and overall survival according to current evidence. Multiagent regimens such as FOLFIRINOX or gemcitabine/nab-paclitaxel are preferred to facilitate downstaging and enable secondary resectability. Despite these advances, prediction of individual response remains challenging, and in many cases NAC does not lead to measurable tumor shrinkage. Patients may thus suffer chemotherapy-related adverse events without oncological benefit. Switching chemotherapy due to poor response, disease progression or treatment-related toxicity is frequent, yet evidence regarding the impact of switching to an alternative chemotherapy regimen in such situations is scarce. In clinical practice, individualized treatment approaches may therefore include a regimen switch.

**Material and methods:**
We retrospectively analyzed a cohort of 214 patients with pancreatic ductal adenocarcinoma (PDAC) who received neoadjuvant chemotherapy (NAC) at a high-volume pancreatic cancer center between 2012 and 2024. In 40 patients (18.7%), chemotherapy was discontinued prematurely due to either disease progression or chemotherapy-related toxicity.

**Results:**
In 22 patients (10.2%), NAC was discontinued because of disease progression. Fourteen of these patients had received FOLFOXIRI/FOLFIRINOX, seven gemcitabine/nab-paclitaxel, and one olaparib. Chemotherapy was discontinued after a mean of 3.91 cycles for FOLFOXIRI/FOLFIRINOX and 4.0 cycles for gemcitabine-based therapy. Nine patients (40.9%) were switched to an alternative regimen, all to gemcitabine/nab-paclitaxel. Secondary operability was achieved in five patients (55.6%), and four patients (44.4%) underwent R0 resection. In contrast, none of the eleven patients without a regimen switch underwent surgery, and all were transitioned to palliative treatment.
Another 18 patients (8.4%) discontinued NAC due to chemotherapy-related toxicity. Ten of these patients had initially received FOLFOXIRI/FOLFIRINOX, and eight a gemcitabine-based regimen. Chemotherapy was discontinued after a median of 4.0 cycles for FOLFOXIRI/FOLFIRINOX and 2.4 cycles for gemcitabine.

Six patients (33.3%) were switched to an alternative regimen: four to gemcitabine, one to FOLFOXIRI, and one to FOLFIRINOX. All six underwent surgery, and in every case R0 resection was achieved. Among the 12 patients without a regimen switch, operability was achieved in 50% (6/12), with R0 resection in 41.7% (5/12).

**Conclusion:**
These findings suggest that switching to an alternative chemotherapy regimen after discontinuation of NAC may improve the likelihood of achieving secondary operability and R0 resection, both in cases of tumor progression and chemotherapy-related toxicity. However, it is unclear which patient subgroups may benefit from CTx switch.  Especially the role of biochemical markers such as CA19-9 and the lack of standardized criteria for radiological response complicate patient selection. The sequential use of multiple chemotherapy regimens carries a higher risk of cumulative toxicity and must be weighed carefully in clinical decision-making. The Efficacy and the optimal timing of CTx switch for increasing R0 resection rate is still unclear and should be subject for further trials.

DE GRUYTER BRILL

**Robotic ALPPS: A Single-Center Experience**
ID: E-DCK-387

**Viszeralchirurgie / Visceral Surgery**
**Hepato-Pankreato-Biliäre Chirurgie (CALGP) / Hepato-Pancreato-Biliary Surgery (CALGP)**

Charlotte Pollmann[1], Andreas Andreou[1], Felix Becker[1], Andreas Pascher[1], Haluk Morgul[1], Benjamin Strücker[1], Shadi Katou[1]

[1]Universitätsklinikum, Allgemein-, Viszeral- und Transplantationschirurgie, Münster, Deutschland

**Background:**
The ALPPS procedure (Associating Liver Partition and Portal Vein Ligation for Staged Hepatectomy) remains controversial due to reported high morbidity and mortality.
The robotic approach in liver surgery adds further advantages: greater precision, reduced tissue trauma, shorter hospital stays, and faster patient recovery. These features increase safety in complex procedures. However, evidence on robotic ALPPS is still limited, especially regarding perioperative outcomes and complications.

**Material and methods:**
A total of 21 patients who underwent robotic ALPPS at the University Hospital Münster between May 2023 and July 2025 were included. Demographic and perioperative data were retrospectively analyzed. Length of hospital stay after ALPPS stage one and two, and postoperative complications were evaluated.

**Results:**
The series included 57% men (n = 12) and 43% women (n = 9), with ages ranging from 19 to 74 years (mean 57 years). The indications for resection included liver metastases from colorectal cancer (n = 8) or anal cancer (n=1), intrahepatic cholangiocarcinoma (n = 4), hepatocellular carcinoma (n = 3), hepatic echinococcosis (n = 2), and three other conditions.
Mean surgical time of ALPPS stage one was 286 minutes. The average hospital stay after stage one was six days; with the exception of two cases, all patients were discharged between the two stages. The median interval between ALPPS stage one and stage two was 15 days. During this period, one patient showed progressive disease, and one patient died of sepsis. ALPPS stage two was performed in 19 cases, with a mean surgical time of 384 minutes. The average hospital stay after stage two was 11 days. Among all 21 patients, the mortality rate was 4,8% (n = 1). Of the 16 patients with a malignant disease, 75% (n = 12) achieved R0 resection. Major complications (Clavien-Dindo >3b) occurred in 21% (n = 4).

**Conclusion:**
This is the largest case series of robotic ALPPS to our knowledge, demonstrating that the approach is feasible and can be established at an experienced center. We observed acceptable stages completion and major morbidity rates, while discharging most patients between both stages.

DE GRUYTER BRILL

### International Reference Values for Surgical Outcomes of Total Pancreatectomy from 25 Expert Centers

ID: E-DCK-394

**Viszeralchirurgie / Visceral Surgery**
**Hepato-Pankreato-Biliäre Chirurgie (CALGP) / Hepato-Pancreato-Biliary Surgery (CALGP)**

Philip Müller[1], Christoph Kümmerli[1], Caroline Berchtold[1], Janina Eden[1], Philipp Dutkowski[1], Adrian Billeter[1], Beat Müller[1]

[1]Clarunis - University Digestive Health Care Center Basel, Basel, Schweiz

**Background:**
Total pancreatectomy (TP) is indicated for advanced pancreatic cancer or multifocal tumors. Furthermore, TP may be performed to avoid the risk of pancreatic fistula in selected patients to improve the perioperative risk profile. The aim of this international multicenter study was to define reference values for TP based on a low-risk cohort treated at expert centers.

**Material and methods:**
This multicenter study analyzed outcomes from patients undergoing primary TP for malignant or benign lesions from 25 international expert centers from 01/2017 to 11/2023. Low-risk TP (LR-TP) patients were without vascular resections or significant comorbidities. Twenty reference values were derived from the 75$^{th}$ or the 25$^{th}$ percentile of the median values of all centers. Outcomes of LR-TP were compared to a cohort of TP with vascular resection, TP due to high-risk pancreatic anastomosis and the benchmark values for low-risk pancreatoduodenectomy (PD).

**Results:**
Of 994 patients, 333 (33.5%) qualified as the LR-TP cohort. Reference values included blood loss (≤1000ml), major complications (≤37%), 3 months postoperative mortality (<6%) and retrieved lymph nodes (≥29). Compared to TP with vascular resections, reference cutoffs were not met for major complications (51% vs LR-TP ≤37%) and 90-day mortality (10.9% vs LR-TP ≤6%). For TP due to high-risk anastomosis, failure to rescue rate (38% vs ≤6.3%) and 90-day mortality (11.1% vs LR-TP ≤6%) were not met. Compared to PD, reference values for postoperative mortality were four times higher for LR-TP (≤1.6 vs. ≤6.3%) and less for resected lymph nodes (≥16 vs. ≥29).

**Conclusion:**
This analysis sets the first global reference values for TP, indicating significantly higher postoperative morbidity and mortality compared to PD. Perioperative morbidity of TP was especially increased in patients with vascular resections. These reference values can serve for quality control of pancreatic surgery.

DE GRUYTER BRILL

**Spleen preservation versus splenectomy during left pancreatectomy for IPMN: a meta-analysis**
ID: E-DCK-396

**Viszeralchirurgie / Visceral Surgery**
**Hepato-Pankreato-Biliäre Chirurgie (CALGP) / Hepato-Pancreato-Biliary Surgery (CALGP)**

Ingmar F Rompen[1], Anouk van Bodegraven[2], Charlotte Leseman[2], Thomas Hank[1], Henrik Nienhüser[1], Christoph Michalski[1], Martin Loos[1], Giovanni Marchegiani[3], Marc Besselink[2]

[1]Department of General, Visceral and Transplantation Surgery, Heidelberg University Hospital, Heidelberg, Deutschland, [2]Department of Surgery, AmsterdamUMC, Amsterdam, Niederlande, [3]Department of Pancreatic Surgery, University Hospital of Padova, Padova, Italien

**Background:**
The value of splenectomy for patients undergoing left pancreatectomy for intraductal papillary mucinous neoplasm (IPMN) is unclear. This was identified as a topic of interest in the ongoing international update of the IPMN guidelines. This systematic review addresses the oncological safety of spleen preserving left pancreatectomy (SPLP) versus left pancreatectomy with splenectomy (LPS) for IPMN.

**Material and methods:**
A systematic review was conducted adhering to the Cochrane Handbook for Systematic reviews of Interventions within the electronic databases of PubMed, Embase, and Cochrane. The primary outcome was oncological safety of SPLP for IPMN measured by IPMN recurrence rate and overall survival. Secondary outcomes included postoperative morbidity and mortality.

**Results:**
Overall, 12 observational studies with 3,325 patients undergoing left pancreatectomy were included of which 1,265 with IPMN. One study included only patients with IPMN. No difference was observed in Clavien-Dindo ≥3a complications (OR 0.89, 95% CI 0.56 to 1.42; p = 0.56) and mortality (OR 1.12, 95% CI 0.04 to 32.43; p = 0.90) between …. and …. Overwhelming post-splenectomy infection syndrome (OPSI) was rarely reported with 1/330 (0.3%) observed case in the splenectomy group. No difference in IPMN-specific recurrence/secondary progression rate (OR 0.56, 95% CI 0.00 to 195.67; p = 0.42) or overall survival (adjusted-HR 0.50, 95%CI: 0.22 to 1.18) was observed between SPLP and LPS for IPMN.

**Conclusion:**
During left pancreatectomy for IPMN, the oncological safety and surgical outcome are not compromised by spleen preservation. Spleen preservation may therefore be considered in patients undergoing left pancreatectomy for IPMN without preoperatively suspected overt malignancy.

DE GRUYTER BRILL

**3D visualization in liver surgery – a necessary technique?**
ID: E-DCK-408

**Viszeralchirurgie / Visceral Surgery**
**Hepato-Pankreato-Biliäre Chirurgie (CALGP) / Hepato-Pancreato-Biliary Surgery (CALGP)**

Esther Giehl-Brown[1], Frederic Konrad[2], Steffen Löck[2], Johannes Schweipert[2], Felix Schön[2], Jens Kühn[2], Jürgen Weitz[2], Carina Riediger[3]

[1]Universitätsklinikum Heidelberg, Heidelberg, Deutschland, [2]TU Dresden, Dresden, Deutschland, [3]Marienhospital Stuttgart, Stuttgart, Deutschland

**Background:**
Successful liver resection in oncologic surgery depends on safety, precision, and efficacy, all of which require a thorough understanding of liver anatomy. Contrast-enhanced computed tomography (CT)-generated 3D models have been proposed as a valuable tool to enhance this understanding. However, a systematic comparison of different display modalities across professional groups has not yet been performed.

**Material and methods:**
In this prospective, monocentric randomized trial, high-resolution 2D CT images of liver malignancies were compared with their corresponding standardized, non-colored 3D virtual and printed models in facilitating anatomical and spatial understanding as well as surgical decision-making. A total of 91 study participants, including 40 surgeons, 10 radiologists, and 41 students, evaluated six clinical cases (three centrally and three peripherally located liver malignancies). Each participant assessed one central and one peripheral case per display modality, presented in a random order.

**Results:**
Objectively, both 3D virtual and printed models significantly improved the correct identification of tumor location ($p<0.001$), enhanced the comprehension of spatial relationships with adjacent liver and portal veins ($p<0.001$ and $p=0.019$, respectively), and facilitated clinical decision-making ($p<0.001$) compared to CT images. No significant difference was observed between virtual and printed models in terms of effectiveness. Within the different professional subgroups, surgeons and students showed superiority applying 3D models, while no meaningful differences within the subgroups of radiologists were identified between display modalities. Subjectively, the majority of surgeons and students preferred 3D printed models over virtual models and CT images.

**Conclusion:**
This study demonstrates that standardized, non-colored 3D virtual and printed models equally enhance preoperative anatomical understanding and decision-making, particularly for surgeons and students. By isolating the influence of display modality, our findings clarify prior inconsistent results and support the integration of cost-effective 3D visualization by applying virtual models into surgical planning and education. Preference for printed models despite comparable efficacy highlights the importance of user-centered implementation strategies.

DE GRUYTER BRILL

**Long-term quality of life after pancreatic resection for malignancy: a prospective study**
ID: E-DCK-446

**Viszeralchirurgie / Visceral Surgery**
**Hepato-Pankreato-Biliäre Chirurgie (CALGP) / Hepato-Pancreato-Biliary Surgery (CALGP)**

Florian Schepp[1], Florian Oehme[2], Sandra Korn[2], Marius Distler[2], Jürgen Weitz[2], Olga Radulova-Mauersberger[2]

[1]Klinik für Allgemein-, Viszeral- und Transplantationschirurgie, Universitätsklinikum Augsburg, Augsburg, Deutschland, [2]Klinik und Poliklinik für Viszeral-, Thorax- und Gefäßchirurgie, Universitätsklinikum Dresden, Dresden, Deutschland

**Background:**
Pancreatic surgery for malignant diseases is associated with substantial morbidity and is known to negatively affect short-term postoperative quality of life. However, data on long-term QoL beyond the early postoperative phase—particularly among patients who survive five years or longer—remain scarce due to the aggressive nature of these malignancies. This study aimed to evaluate cross-sectional quality of life in patients after pancreatic resection across different postoperative time intervals of up to 12 years.

**Material and methods:**
We conducted a prospective cross-sectional study including all patients who underwent pancreatic resection for malignant disease at the University Hospital Dresden (Technische Universität Dresden) between 2013 and 2024. The hospital's clinical information system was screened to identify patients who were still alive. Eligible patients were contacted by telephone and asked to provide informed consent to participate in the study. After verbal consent, the EORTC QLQ-C30 and PAN26 questionnaires were mailed to the participants for completion and return by post. Quality of life was assessed once based on the returned questionnaires. The primary endpoint was the global health status score, which was analyzed for each postoperative year up to 12 years and subsequently grouped into three categories: <5 years, 5–10 years, and >10 years after surgery.

**Results:**
A total of 216 patients were identified as alive in the clinical information system (CIS) and were subsequently contacted by telephone. Of these, 62 patients (28.7%) had invalid contact information, and 21 patients (9.7%) were confirmed deceased after inquiry with relatives. Seven patients (3.2%) declined to participate in the study, while 126 patients (58.3%) agreed to participate and consented to complete the questionnaires. Of these, 95 patients (75.4%) returned the questionnaires fully completed and were included in the final analysis.
Among the study cohort, 52.6% had pancreatic ductal adenocarcinoma. The majority underwent pancreatic head resection (64.3%), followed by distal pancreatectomy (25.3%) and total pancreatectomy (8.4%).
The global health status (GHS) showed the most pronounced improvement during the first three years after surgery. The mean GHS was 47.7 (SD 20.9) one year after resection and normalized to 68.9 (SD 16.3) after three years. Interestingly, patients who had major postoperative complications initially reported significantly higher pain scores within the first five years after surgery (31 (SD 25)). Beyond this period, however, the difference in pain perception between patients with and without severe complications diminished and was no longer clinically relevant.

**Conclusion:**
This prospective cross-sectional study represents one of the largest contemporary datasets assessing quality of life after pancreatic surgery. Our findings demonstrate a continuous improvement of quality of life over time, with stabilization approximately five years after resection. Notably, even long-term survivors report meaningful and sustained quality of life levels, highlighting that recovery after pancreatic surgery extends well beyond the early postoperative phase.

DE GRUYTER BRILL

### Spleen Preservation in Distal Pancreatectomy for Benign and Low-Malignant Lesions

ID: E-DCK-493

**Viszeralchirurgie / Visceral Surgery**
**Hepato-Pankreato-Biliäre Chirurgie (CALGP) / Hepato-Pancreato-Biliary Surgery (CALGP)**

Alexander Werba[1], Julia Raba[1], Mohammadamin Shahrbaf[1], Ulf Hinz[1], Ali Ramouz[1], Andrea Schuh[1], Fabian Schuh[1], Till Seiboldt[1], Markus Büchler[2], Rosa Klotz[1], Martin Loos[1], Frank Pianka[1]

[1]Universitätsklinikum Heidelberg, Klinik für Allgemein-, Viszeral- und Transplantationschirurgie, Heidelberg, Deutschland, [2]Champalimaud Foundation, Lissabon, Portugal

**Background:**
While splenectomy is standard in oncologic distal pancreatectomy (DP), its necessity is unclear for benign or low-malignant disease. Spleen preservation may reduce long-term risks of asplenia but could increase perioperative morbidity. Evidence from large homogeneous cohorts is scarce.

**Material and methods:**
This retrospective cohort study analyzed all consecutive adults undergoing elective distal pancreatectomy at a high-volume tertiary referral center. To ensure sufficient sample size, spleen-preserving distal pancreatectomy (SPDP) cases were included from 2002 onward, whereas DP cases with splenectomy were included from 2011. The study period ended in 2022. Patients with pancreatic ductal adenocarcinoma or other high-grade malignancies were excluded. Outcomes were defined by International Study Group of Pancreatic Surgery (ISGPS) criteria. Associations with postpancreatectomy hemorrhage (PPH) were examined using multivariable logistic regression adjusted for potential confounders.

**Results:**
A total of 943 patients were included (302 SPDP, 641 DP). The Kimura technique accounted for 90.1% of SPDP. Clinically relevant postoperative pancreatic fistula (POPF) occurred in 23.5% overall, severe morbidity (Clavien–Dindo ≥III) in 20.6%, and 90-day mortality in 0.4%. The incidence of PPH was higher after SPDP than DP (8.6% vs. 5.1%, $p = 0.041$). PPH risk increased stepwise with fistula severity (2.6% with no/biochemical leak, 14.3% with grade B, 57.9% with grade C; $p < 0.0001$). In multivariable analysis, SPDP was independently associated with PPH (OR 1.98, 95% CI 1.14–3.43), while female sex was protective (OR 0.54, 95% CI 0.31–0.93).

**Conclusion:**
SPDP for benign and low-malignant lesions is safe but carries an increased risk of hemorrhage, largely mediated by POPF. Careful patient selection and vigilant postoperative monitoring are essential to balance early morbidity against the long-term sequelae of splenectomy.

Abstracts – DCK 2026 – 143rd Congress of the German Society of Surgery – Leipzig, April 22–24 • DOI 10.1515/iss-2026-2002
Innov Surg Sci 2026; 11 (Special Suppl 1): s77–s269

DE GRUYTER BRILL

**Improving outcomes in patients after total pancreatectomy following multimodal ERAS protocols**
ID: E-DCK-503

**Viszeralchirurgie / Visceral Surgery**
**Hepato-Pankreato-Biliäre Chirurgie (CALGP) / Hepato-Pancreato-Biliary Surgery (CALGP)**

Matthäus Felsenstein[1], Dou MA[1]

[1]Charité – Univversitätsmedizin Berlin, Chirurgische Klinik, Berlin, Deutschland

**Background:**
Standardized protocols from the Enhanced Recovery after Surgery (ERAS) society are widely implemented across institutions. Evidence have shown clear benefits in patients after major hepato-pancreatobiliary surgeries (e.g. reduced morbidity and shorter in-hospital stay). Still, data on ERAS items and their impact on the clinical outcome following major pancreatic resection remain scarce and sometimes ambigous. After prospective evaluation of the ERAS program at our high-volume, pancreatic carcinoma center, we retrospectively compared this cohort with patients prior implementation of ERAS guided concepts.

**Material and methods:**
A prospective observational study comparing outcome in patients after total pancreatecomty prior (2014-2018) and after (2019-2022) establishement of the ERAS program at the Charité – Universitätsmedizin Berlin. ERAS items were applied according to the ERAS society recommendations.

**Results:**
During the entire observational period, a total of 238 PDAC patients underwent total pancreatectomy, while 100 prior and 138 after ERAS study initiation. During the ERAS trial we identified patients that were allocated into ERAS care (n=90) and non-ERAS care (n=48). Comprehensive follow-up data for oncologic indications were available for 133 patients, with a mean follow-up duration of 392 days (+/-26.3d). Stage adjusted Cox-regression analysis revealed a relevant survival benefit for PDAC patients in the ERAS group (HR=0.517, 95%CI [0.53-1.36]) compared to standard care and pre-ERAS trial. The ERAS group demonstrated a significantly shorter hospital stay (15.7d vs 25.5d), ICU stay (4.8d vs 6.9d) and operative time (289.8 min vs 409.9min) compared to the pre-ERAS group ($p<0.001$). When comparing with a non-ERAS group, overall postoperative complication rates decreased from 84.7% to 62.5% in patients strictly following the ERAS concept, along with a reduction in the number of patients experiencing major complications (10.3% vs 35.7%) classified as Clavien-Dindo grade IIIB or higher ($p < 0.05$). No significant differences were observed between the two groups in terms of complexity of the surgery, 30-day mortality, ASA score and PPH. We characterized the adherence on specific ERAS items relevant for pancreatic surgery. We herein present recommendations for specialized perioperative care of patients undergoing total pancreatectomy.

**Conclusion:**
The implementation of ERAS protocols at high volume pancreatic cancer centers likely improves the patients outcome after total pancreatectomy. Special perioperative care is needed for patients experiencing total gland removal, while the ERAS society offers protocols for optimized surveillance and enhanced recovery.

DE GRUYTER BRILL

**Continous real time glucose assessment as a predictor for complications after pancreatic resection**
ID: E-DCK-57

**Viszeralchirurgie / Visceral Surgery**
**Hepato-Pankreato-Biliäre Chirurgie (CALGP) / Hepato-Pancreato-Biliary Surgery (CALGP)**

Patryk Kambakamba[1]

[1]St. Josefs Hospital, Department of Surgery, Wiesbaden, Deutschland

**Background:**
Postoperative hyperglycaemia has been described as an early marker of complications after pancreatic resection. However, evidence is based on retrospective assessment of arbitrary serum glucose measurements. In contrast, continuous glucose monitoring (CGM) systems allow real-time monitoring of glucose fluctuations. Therefore, this study investigates continuous perioperative glucose changes after pancreatic resection and the impact on postoperative complications.

**Material and methods:**
Twenty (n=20) consecutive patients undergoing pancreatic resection were prospectively enrolled. In addition, n=10 patients undergoing other major abdominal surgery served as control group. Dexcom G6 CGM system was used. Time in euglycemic range (TIR) and peak glucose levels were analyzed. Routine serum glucose measurements and daily C-reactive protein (CRP) levels were also assessed. Comprehensive Complication Index (CCI) was used to quantify postoperative complications.

**Results:**
No adverse events related to CGM devices were observed. Glucose levels increased significantly from a median of 7mmol/l (IQR 6-8mmol/l) to 9 mmol/l (IQR 8-1mmol/l, P=0.026) after pancreatic resection. Correspondingly, the TIR decreased from 86.5% (IQR 85-96%) to 78.1% (IQR 34-89%, p=0.042). Perioperative glucose levels (p=0.623) and TIR (p=0.408) remained unchanged in the control group. Linear regression showed a significant correlation between peak glucose levels on day 1, measured by CGM (*R*=0.738, p=0.004, Figure 1A), and CRP levels on day 2 (*R*=0.528, p=0.034, Figure 1B) with CCI. In contrast, routine serum glucose levels did not to predict complications.

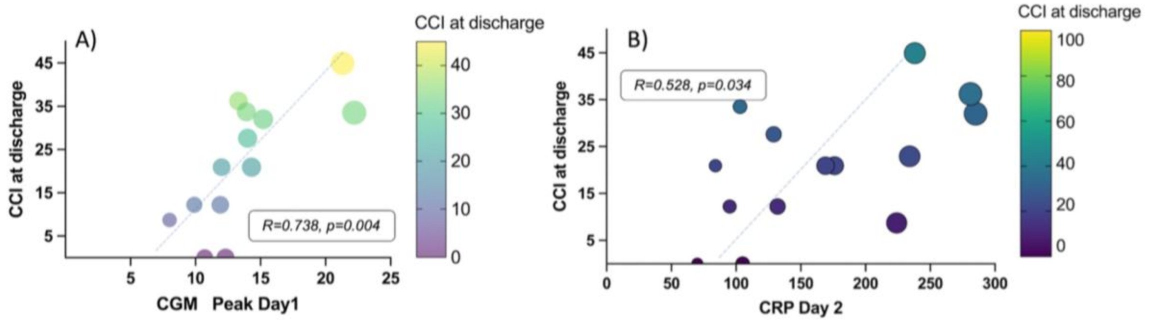

**Conclusion:**
In this pilot study, peak glucose levels on day 1 after pancreatic resection were associated with adverse events. CGM may be a valuable tool to identify patients at risk of complications.

DE GRUYTER BRILL

**Initial experiences with single-port robotic-assisted eTEP-Sublay-Herniotomy**
ID: E-DCK-225

**Viszeralchirurgie / Visceral Surgery**
**Hernien und Bauchwandchirurgie (CAH) / Hernia and Abdominal Wall Surgery (CAH)**

Julian Hipp[1], Robin Klewitz[1], Hannes Neeff[1], Stefan Fichtner-Feigl[1], Philipp Holzner[1]

[1]Universitätsklinikum Freiburg, Klinik für Allgemein- und Viszeralchirurgie, Freiburg, Deutschland

**Background:**
Robotic-assisted minimally-invasive extended totally extraperitoneal plasty (eTEP)-Sublay-Herniotomy is one of the most promising novel techniques for the management of ventral hernia [1]. While several techniques for laparoscopic and multiport-robotic-assisted eTEP have been described, technical developments of the available robotic systems offer novel innovative approaches to single-port robotic-assisted eTEP-Sublay-Herniotomy (SP-eTEP) with the introduction of the Da Vinci-SP-system [1,2,3]. A suprapubic [2,3] and a lateral-dock [4] access for single-port robotic eTEP-Sublay-Herniotomy have been described. We would like to share our initial experiences with this novel robotic approach in the treatment of ventral hernia.

**Material and methods:**
The clinical data of the first 21 consecutive patients undergoing SP-eTEP procedures from 03-08/2025 were analyzed. Results are shown either as number (percent) or median (interquartile range).

**Results:**
21 patients (12 male, 9 female, age 63 years (53-70), BMI 30 kg/m² (25-35)) with ventral hernia were treated by SP-eTEP using the Da Vinci SP-system from 03-08/2025. 17 (81%) had an incisional hernia, 2 (9.5 %) had parastomal hernia and 2 (9.5%) had a primary ventral hernia. The defect width was W1 in 3 (14%), W2 in 17 (81%) and W3 in 1 (5%) patient according to the EHS-classification for incisional hernia. We used a suprapubic approach in 8 patients (38%) while in 13 patients (62%) the lateral-dock access to the retrorectus space was used for SP-eTEP. Operation time was 170 minutes (152-216) for all patients. For eTEP-Rives-Stoppa-Sublay-Herniotomy (n=16) operationtime was 167 minutes (129-203), while for eTEP with simultaneous transversus-abdominis-release or Sugarbaker/Pauli-Repair for parastomal hernia the operationtime was 214 minutes (179-224). Median defect size was 37 cm² (21-78) and median mesh size was 352 cm² (305-498). Mesh-to-Defect-ratio was 9.2 (5.1-13.6). Length of stay was 4 days (3-4) with 0 days (0-0.75) ICU stay. Re-admission rate to ICU was 0%. Complications according to Clavien-Dindo-classifications were 0% grade III-V. 30-day-surgical-site occurrence requiring procedural intervention (SSOPI)-rate was 0%.

**Conclusion:**
SP-eTEP is technically feasible for a wide variety of hernia defects with promising short-term results. The future evaluation of the novel technique according to the IDEAL-framework for surgical innovation monitoring patient safety (short- and long-term-complications, recurrence rate, trocar-hernia-rate), and comparative effectiveness to laparascopic and multiport-robotic eTEP will be necessary.

**References:**

[1] Belyansky I, Reza Zahiri H, Sanford Z, Weltz AS, Park A. Early operative outcomes of endoscopic (eTEP access) robotic-assisted retromuscular abdominal wall hernia repair. Hernia. 2018 Oct;22(5):837-847. doi: 10.1007/s10029-018-1795-z.
[2] Kim CH, Al-Sawat A, Lee CS (2022) Single-port robot-assisted extended totally extraperitoneal repair for ventral hernia using the Da Vinci single-port platform: A video vignette. Asian J Surg 45:1751–1752. 10.1016/j.asjsur.2022.01.042
[3] Kim IK, Han S-R (2023) Single-port robotic extended totally extraperitoneal (eTEP) with transverse abdominis release (TAR) for lateral ventral hernia repair using the Da Vinci SP platform. Asian J Surg 46:2829–2830. 10.1016/j.asjsur.2023.01.064
[4] Hipp J, Klewitz R, Neeff H, Fichtner-Feigl S, Holzner PA. Lateral-dock single-port robotic-assisted extended totally extraperitoneal plasty (eTEP)-Sublay-Herniotomy-Procedure- presentation of a novel technique for robotic-assisted ventral hernia surgery. Hernia. 2025 Jul 12;29(1):231. doi: 10.1007/s10029-025-03420-w.

DE GRUYTER BRILL

**Meta-analysis of different neurectomy techniques for chronic postoperative inguinal pain**
ID: E-DCK-27

**Viszeralchirurgie / Visceral Surgery**
**Hernien und Bauchwandchirurgie (CAH) / Hernia and Abdominal Wall Surgery (CAH)**

Emmanouil Charitakis[1], Lisa-Marie Schupp[2], Farah Al Hasani-Pfister[2], Robert Christian Bauer[2], Sebastian H. Lamm[2], Robert Rosenberg[2], Anas Taha[2]

[1]Faculty of Medicine, University of Basel, Basel, Schweiz, [2]Cantonal Hospital Baselland, Department of Visceral Surgery, Liestal, Schweiz

**Background:**
Chronic postoperative inguinal pain (CPIP) is a rare, but challenging long-term complication of inguinal hernia repair. Its incidence varies widely in the literature, ranging from 0.7% to 43.3%. In this meta-analysis, we aim to analyze the outcomes of neurectomy for treating this condition, with a focus on the efficacy of various surgical approaches (open, laparoscopic transabdominal, laparoscopic retroperitoneal and combined approach), and types of neurectomy—single, double and triple.

**Material and methods:**
The research was registered with PROSPERO (CRD42023475401). We conducted a search in Medline, Embase, Scopus, and Cochrane Central databases from the inception to November 2023. All patients aged 18 years and older who underwent neurectomy for treating CPIP were included. We selected only randomized controlled trials, cohort studies and case series, according to PRISMA guidelines. A random-effects model was used for single-arm analyses. The inverse variance and Mantel-Haenszel methods were employed for two-arm analyses.

**Results:**
The analysis includes fifteen studies involving 701 patients. After neurectomy, 90% of the patients experienced an improvement in pain overall. Only 9.4% of the patients had postoperative complications. Among the surgical types, triple neurectomy demonstrated the highest overall pain improvement rate at 98.2%. Double neurectomy showed the highest rate of complete pain relief at 80.1%, but it also had a higher complication rate of 15.3%. With regard to surgical approach, the laparoscopic retroperitoneal method not only had the highest overall improvement rate of 95.5% compared to other approaches, but also the highest complication rate of 28.7%.

**Conclusion:**
Neurectomy was shown to be an effective treatment for neuropathic CPIP across various surgical techniques. Our meta-analysis revealed an improvement in pain in 90% of patients, with 61.4% experiencing complete relief, as evidenced by significant reductions in Visual Analogue Scale (VAS) scores. Both double and triple neurectomy approaches have shown efficacy; however, the triple neurectomy does show an increased overall improvement, albeit it incurs some surgical challenges. Open, combined, and laparoscopic retroperitoneal approaches all produced good results, but with different rates of improvement and complication profiles. Consequently, complications like skin hyperesthesia and wound infections, while infrequent, should still be considered in patient management. Other factors contributing to improvement in pain levels include the removal of mesh, thus advocating individualistic treatment methods. The assessments give support to the safety and effectiveness of neurectomy in the management of chronic postherniorrhaphy pain, but more studies, ideally randomized controlled trials with a focus on patient reported outcome measures (PROMs), would be important to clarify the choice of surgical strategy and improve patient outcome.

DE GRUYTER BRILL

**Minimally-invasive management of a recurrent hernia in eTEP-Re-Sublay-technique - a case report**
ID: E-DCK-335

**Viszeralchirurgie / Visceral Surgery**
**Hernien und Bauchwandchirurgie (CAH) / Hernia and Abdominal Wall Surgery (CAH)**

Gergana Nenova[1], Robin Klewitz[1], Stefan Fichtner-Feigl[1], Julian Hipp[1]

[1]Universitätsklinikum, Freiburg, Deutschland

**Background:**
Surgery for recurrent hernias after mesh augmentation remains a significant challenge for abdominal wall surgeons. Despite rapid advances in minimally invasive and robotic techniques, most recurrent hernias are still managed with open surgery or intraperitoneal onlay mesh (IPOM). Current guidelines provide no clear recommendations on how to address recurrence after hernia repair with mesh reinforcement in the sublay position. The sublay position generally appears favorable for its low recurrence rates and the extraperitoneal placement of the mesh.

**Material and methods:**
We present the case of a 41-year-old woman who underwent open right hemicolectomy for a neuroendocrine tumor (NET) of the appendix and developed a large midline incisional hernia (M2-3W3 according to the EHS-classification of incisional hernia, 11 × 9 cm) two years postoperatively. Chemical component separation with botulinum toxin followed by open Rives-Stoppa-sublay-hernioplasty with intraoperative fascial traction had been performed with a 25x12 cm polypropylene mesh at another hospital. One year after the open hernia repair with retromuscular mesh, she presented to our outpatient clinic with a recurrent hernia cranial and caudal to the mesh (M2,M4W2). After thorough evaluation and discussion with the patient, we planned an extended totally extraperitoneal (eTEP) repair with mesh augmentation in the sublay position. The patient had no relevant comorbidities, and preoperative imaging was available from a recent PET-CT obtained for oncological follow-up.

**Results:**
After initial creation of a retromuscular space along the upper left quadrant cranial to the old mesh and along the left EIT-ambivium, the cross-over was performed over the previous mesh. After that, the cranial and caudal hernia were reduced, the retroxyphoidal and retropubic spaces were developed, and the abdominal wall was reconstructed by suturing of the anterior and posterior sheaths. A new polypropylene mesh of 42x15 cm was positioned in the retromuscular plane from the retroxiphoid to the retrosymphysial region anterior to the prior mesh. The integrity of the rectus muscle and the neurovascular bundles was preserved. The postoperative course was uneventful and the patient was discharged on postoperative day 4.

**Conclusion:**
From this case, we learned that eTEP is a feasible technique for recurrent hernias after prior retromuscular repair, if an initial retromuscular space can be created for at least one camera trocar and one additional working trocar. With careful preoperative planning and performed by an experienced laparoscopic surgeon, this approach represents a suitable option for selected patients with recurrent hernias after sublay hernioplasty.

DE GRUYTER BRILL

**Rare Complication After Modern Minimally Invasive Techniques for Incisional Hernia Repair**
ID: E-DCK-445

**Viszeralchirurgie / Visceral Surgery**
**Hernien und Bauchwandchirurgie (CAH) / Hernia and Abdominal Wall Surgery (CAH)**

Movsar Borshchigov[1]

[1]Franziskus Krankenhaus Berlin, Chirurgie, Berlin, Deutschland

**Background:**
Minimally invasive techniques for ventral hernia repair, such as the *extended-view totally extraperitoneal* (eTEP) approach and the *Mini- or Less-open Sublay* (MILOS) technique, have gained increasing popularity in recent years. These methods aim to reduce the complications associated with intraperitoneal onlay mesh (IPOM) placement, such as adhesions, chronic pain, and bowel erosion (1–3).
Despite these advances, rare and often underestimated complications have been observed in the retromuscular plane. One such complication is the **interparietal hernia (IH)**, in which bowel loops become trapped between the layers of the abdominal wall. The underlying cause is typically a postoperative deformation or partial dehiscence of the posterior rectus sheath, which creates a potential interparietal space for herniation (2, 4). Clinical presentation is frequently nonspecific, including abdominal distension, anorexia, and signs of subileus, which can delay diagnosis and treatment.

**Material and methods:**
Over a five-year period, more than **175 ventral hernia repairs** were performed using minimally invasive retromuscular techniques (eTEP, MILOS, and eMILOS) at a specialized hernia center. Postoperative outcomes were retrospectively analyzed to identify rare complications and evaluate their clinical presentation, diagnostic pathway, and management.

**Results:**
Two patients (incidence 1.1%) developed **interparietal retrorectal hernia (iRRH)** following minimally invasive sublay repair. Both patients presented with delayed return of bowel function and repeated loss of drain vacuum. Abdominal CT scans revealed bowel distension but failed to identify the cause of obstruction. Diagnostic laparoscopy ultimately confirmed the presence of small bowel loops entrapped between the posterior rectus sheath and the mesh, secondary to partial fascial dehiscence.
Surgical revision was performed laparoscopically, with reduction of the bowel and tension-free closure of the posterior fascial defect using absorbable inlay mesh reinforcement. Neither case required bowel resection, and both patients recovered without further complications.

Interparietal hernia is a **rare but clinically significant complication** of modern retromuscular hernia repair (2, 5–7). The key factor in prevention is a **tension-free closure of the posterior rectus sheath**. In patients at high risk for fascial tension—such as those with prior IPOM explantation, rigid abdominal wall, or obesity—**preoperative strategies** should be considered. These may include **chemical component separation** with *Botulinum toxin A* and, intraoperatively, **posterior component separation (TAR)** or **cross-stitching techniques** of the fascia to reduce lateral traction.

Drain management may provide early diagnostic clues: repeated loss of suction or vacuum can indicate early fascial dehiscence and should prompt careful clinical assessment. Conventional imaging modalities, such as CT and ultrasound, are often inconclusive in the early postoperative period, as subtle posterior sheath deformations may not be visible. In patients with unexplained postoperative symptoms, **early diagnostic laparoscopy** is recommended to avoid delayed recognition and strangulation (3, 8–10). Although fixation of retromuscular mesh remains a matter of debate, minimal fixation or the use of self-gripping mesh may reduce fascial tension and decrease the risk of postoperative displacement. Surgeons should maintain a high index of suspicion for interparietal entrapment, especially when postoperative ileus symptoms persist without radiological evidence of bowel injury.

**Conclusion:**
The growing adoption of minimally invasive hernia techniques has introduced new and sometimes underrecognized complication patterns. The **interparietal retrorectal hernia** represents one such rare event, underscoring the importance of meticulous posterior sheath closure and vigilance during postoperative monitoring.
Early recognition of atypical postoperative symptoms and incorporation of **tension-reducing strategies**—including preoperative botulinum toxin use and, when indicated, TAR—are essential to minimize the risk of fascial failure. A proactive diagnostic approach, including early laparoscopy in uncertain cases, can prevent serious outcomes and ensure optimal recovery.

**References**

1. Conze J, Klinge U, Schumpelick V. *Hernias.* In: Holzheimer RG, Mannick JA (eds). **Surgical Treatment: Evidence-Based and Problem-Oriented.** Munich: National Library of Medicine; 2001.
2. Kalmar CL, Bower CE. Laparoscopic repair of interparietal abdominal wall hernias. *J Surg Case Rep.* 2019;2019:rjz319.
3. Lower WE, Hicken NF. Interparietal hernias. *Ann Surg.* 1931;94:1070–87.
4. Narayanan S, Davidov T. Peritoneal pocket hernia: a distinct cause of early postoperative small bowel obstruction and strangulation. *J Minim Access Surg.* 2018;14:154–7.
5. Bittner R, Schwarz J. Inguinal hernia repair: current surgical techniques. *Langenbecks Arch Surg.* 2012;397:271–82.
6. Vanden Heuvel B, Dwars BJ, Klassen DR, Bonjer HJ. Is surgical repair of an asymptomatic groin hernia appropriate? A review. *Hernia.* 2011;15:251–9.
7. Sartori A, De Luca M, Noaro G, Piatto G, Pignata G, Di Leo A, et al. Rare intraoperative and postoperative complications after transabdominal laparoscopic hernia repair: results from the Multicenter Wall Hernia Group Registry. *J Laparoendosc Adv Surg Tech A.* 2021;31:290–5.
8. Kapiris SA, Brough WA, Royston CM, O’Boyle C, Sedman PC. Laparoscopic transabdominal preperitoneal (TAPP) hernia repair: a 7-year two-center experience in 3017 patients. *Surg Endosc.* 2001;15:972–5.
9. Köhler G, Mayer F, Lechner M, Bittner R. Small bowel obstruction after TAPP repair caused by a self-anchoring barbed suture device for peritoneal closure: case report and review of the literature. *Hernia.* 2015;19:389–94.
10. Tagliaferri EM, Wong TS, Abad DJJ, Bergmann H, Hammans S, Seidlmayer CM. Small bowel obstruction after TAPP repair caused by self-anchoring barbed suture device for peritoneal closure. *J Surg Case Rep.* 2018;2018:rjy165.

DE GRUYTER BRILL

**Endoscopic Assessment Following Descendo-Rectostomy – Do We Really Need to Know Anastomotic Leakage?**
ID: E-DCK-140

**Viszeralchirurgie / Visceral Surgery**
**Komplikationsmanagement / Complication Management**

Aydar Khabipov[1], Andre Schreiber[1], Stephan Kersting[1], Johannes Höhn[1], MacIej Patrzyk[1], Anne Glitsch[1], Wolfram Kessler[1]

[1]Universitätsmedizin Greifswald, Klinik und Poliklinik für Allgemeine Chirurgie, Viszeral-, Thorax- und Gefäßchirurgie, Greifswald, Deutschland

**Background:**
Anastomotic leakage remains a significant complication following rectal resection, contributing to increased morbidity and prolonged hospitalization. This study aims to evaluate the impact of early endoscopic intervention on the management of anastomotic leaks, focusing on detection time, re-operation rates, hospitalization duration, and the need for standardized post-operative endoscopy protocols.

**Material and methods:**
A retrospective analysis of 124 patients who underwent rectal or sigmoid resection or left hemicolectomy was conducted, dividing them into two groups: those who received endoscopic evaluation for anastomosis control and those who did not. Baseline characteristics were comparable. Outcomes assessed included rates of anastomotic leakage, re-operations, and hospitalization duration.

**Results:**
Our findings demonstrate that the use of endoscopy significantly reduced re-operation rates and hospitalization time for patients with anastomotic leaks. The optimal timing for endoscopic evaluation was identified as day 5-6 post-operation, with a marked increase in leak detection compared to evaluations performed on day 9 or later. Notably, the rate of anastomotic leakage was found to be twice as high in the endoscopy group, suggesting that without endoscopic assessment, the leakage rate may be underestimated.

**Conclusion:**
This study underscores the critical role of early endoscopic intervention in managing anastomotic leaks after rectal resection, highlighting the need for standardized post-operative endoscopy guidelines to optimize decision-making and enhance patient outcomes. Establishing protocols that include optimal timing for endoscopy and a classification system for anastomotic leaks could significantly improve the quality of care for patients undergoing rectal resection.

DE GRUYTER BRILL

**Impact of a Structured Quality Improvement Program on Postoperative Outcomes in Visceral Surgery**
ID: E-DCK-285

**Viszeralchirurgie / Visceral Surgery**
**Komplikationsmanagement / Complication Management**

Lisa Puntigam[1], Hoffmann Wolfgang[1], Valeri Nikolov[1], Thomas Rath[1], Petro Tasellari[1], Alexander Habrom[1], Elke von Haeften[1], Patrick Schneider[1], Sarah Braun[1], Carolin Michael[1], Steffen Güth[1], Fadhl Al Daheri[1], Thilo Skanda[1], Franck Billmann[1 2]

[1]Klinikum Heidenheim, Klinik für Visceral-, Thorax- und Gefäßchirurgie, Heidenheim an der Brenz, Deutschland, [2]Universität Heidelberg, Im Neuenheimer Feld 672, 69120 Heidelberg, Deutschland

**Background:**
Improving postoperative outcomes after standard surgical procedures such as low anterior resection (LAR), gastrectomy and cholezystectomy remains an essential goal in daily surgical practice. Alongside technological advances, quality improvement measures playy a central role in improving post-operative outcomes. Our study aims to examine the impact of these measures.

**Material and methods:**
In a retrospective analysis, we examined peroperativ outcomes. The primary outcome was postoperative complications following three procedures (LAR, gastrectomy and cholecystectomy). The study population was divided into two chronologically successive cohorts: patients operated on between January and December 2024 (cohort A) and patients operated on between January and December 2025 (cohort B). In 2025 s structured quality-improvement program was implemented, including weekly internal morbidity and complication review meetings and the introduction of standard operating procedures (SOPs) for all standard interventions. After matching both cohorts (e.g. for age, comorbidity procedure) a comparative analysis investigated the impact of these measures on perioperative outcomes.

**Results:**
In our final study design we included 40 patients with LAR, 13 patients that received a gastrectomy and overall 500 patients that underwent cholezystectomy. Among the main findings, our analysis revealed a marked reduction in the overall complication rate for LAR (45% vs 28%) and gastrectomy (83% vs 14%) as well as a slight decrease for complications rates following cholecystectomy (7% va 6,2%) (respectively in cohort A vs B). In particular, the rate of anastomotic leakage after low anterior resection was significantly reduced (13.6% va 5.6%) and the total number of wound infections also declined as a result of the implemented measures.

**Conclusion:**
These findings sugest that the structured quality-improvement strategies - including regular complications reviews, implementation of SOPs and increased use of robotic-assisted surgery - may contribute to improved postoperativ outcomes, particularly for major procedures such as low anterior resection and gastrectomy. The limitations of our study are discussed.

DE GRUYTER BRILL

**Postpancreatectomy Hemorrhage: Risk factors and outcomes**
ID: E-DCK-470

**Viszeralchirurgie / Visceral Surgery**
**Komplikationsmanagement / Complication Management**

Karam T. Alhalabi[1], Susanne Roth[1], Ingmar F Rompen[1], Salim Wayandoui[1], Ulf Hinz[1], Jörg Kaiser[1], Benedict Kinny-Köster[1], Mohammed Al-Saeedi[1], Martin Schneider[1], Thilo Hackert[1], Martin Loos[1], Mw Büchler[1], Thomas Hank[1]

[1]Universitätsklinikum Heidelberg, Klinik für Allgemein-, Viszeral- und Transplantationschirurgie, Heidelberg, Deutschland

**Background:**
Although pancreatic surgery has become safer over the past decades, post pancreatectomy hemorrhage (PPH) remains one of the most harmful events after pancreatic resection. While early bleeding is of technical nature, late bleeding events have been associated with vessel arrosion as sequelae of postoperative complications. Studies investigating PPH after complex pancreatic surgery and in patients undergoing total pancreatectomy are lacking. The aim of this study was to compare outcomes of patients with or without PPH and to identify risk factors associated with postoperative mortality.

**Material and methods:**
This analysis was conducted from a prospectively maintained database of patients who underwent elective pancreatic resection at Heidelberg University Hospital from October 1, 2001 to December 21, 2022. PPH rates and associated factors were detected and compared across different resection types. PPH was classified according to the ISGPS classification, including time of occurrence (early vs late), bleeding site and grade of severity (B/C). Additionally, risk factors for mortality in PPH patients were identified using univariate and multivariate analysis.

**Results:**
Of 10,467 patients, 5,711 were men (54.6%); median age was 63 years (IQR 54-71 years). The overall major morbidity rate was 22.7% with an in-hospital mortality of 3.2%. A total of 610 patients (5.9%) experienced PPH. Most patients had late-onset PPH (86.8%) and were detected with extraluminal bleeding (74.6%). According to the ISGPS grading, 196 (32.1%) were classified as Grade B and 414 (67.9%) as Grade C. Independent predictors of PPH were age >70 years (OR 1.36; P=0.002), pancreatoduodenectomy or total pancreatectomy (OR 1.57; P<0.001), intraoperative blood loss ≥1500 mL (OR 1.79; P<0.001), clinically relevant POPF (OR 5.93; P<0.001) and intraabdominal infection (OR 4.03; P<0.001). Both the incidence and the associated mortality of PPH increased with higher surgical complexity. Overall, PPH was associated with prolonged hospital stay (IQR 27.5 vs 12 days; P<0.001) and an almost 10-fold increase in mortality (20.7% vs 2.2%; P<0.001). Independent risk factors for mortality in the PPH group after pancreatic head or distal resections were higher age (OR: 2.79; P=0.001), relaparotomy for bleeding (OR: 6.35; P<0.001), bleeding from the SMA (OR: 3.92; P=0.007) or the hepatic arteries (OR: 3.05; P =0.002), sepsis (OR: 4.10; P<0.001) and very late (POD ³16) bleeding (OR: 3.95; P=0.003). After total pancreatectomy, only recurrent bleeding (OR: 2.36; P=0.007) and intraabdominal infection (OR: 3.10, P=0.022) were identified as independent predictors for mortality.

**Conclusion:**
The occurrence of PPH represents the most severe complication after pancreatic resection and is associated with an almost 10-fold increase in mortality. This effect is even more pronounced in patients undergoing more complex resections. Consequently, strategies to prevent and manage PPH as well as the reduction of risk factors remain a major challenge in modern pancreatic surgery.

DE GRUYTER BRILL

**Robotic Extrahepatic Glissonean Pedicle Versus Dissection Approach for right or left hepatectomy.**
ID: E-DCK-282

**Viszeralchirurgie / Visceral Surgery**
**Minimalinvasive Chirurgie (CAMIC) / Minimally Invasive Surgery (CAMIC)**

Jan Heil[1], Leonard Bopp[1], Elisabeth Miller[1], Nuh Rahbari[1], Emrullah Birgin[1]

[1]Universitätsklinik Ulm, Klinik für Allgemein- und Viszeralchirurgie, Ulm, Deutschland

**Background:**
In recent years, robotic liver surgery has increasingly gained acceptance as an alternative to laparoscopic and open approaches for major hepatectomy by providing enhanced visualization and precise dissection. The optimal technique for pedicle control in robotic right or left hepatectomy, however, remains controversial. In particular, the safety and efficacy of the extrahepatic Glissonean pedicle approach (GA) compared with the dissection approach (DA) are still under discussion. Therefore, this study aimed to evaluate perioperative outcomes of GA versus DA in robotic left and right hepatectomy.

**Material and methods:**
We performed a retrospective analysis of our prospectively maintained institutional database. All robotic left or right hepatectomies conducted between March 2021 and May 2025 were included. Two-stage procedures, sectionectomies, minor hepatectomies (<3 liver segments), and non-anatomical resections were excluded. Depending on the resection technique performed, i.e., GA or DA, perioperative outcomes were assessed up to 90 days postoperatively.

**Results:**
Forty-five patients were included, of whom 22 (49%) underwent GA and 23 (51%) DA. Right hepatectomy was performed in 31 (69%) and left in 14 (31%) patients, with comparable distributions across groups. Baseline characteristics and comorbidities, assessed by the Charlson Comorbidity Index, did not differ. Compared with DA, intraoperative blood loss was significantly lower (500 ml [200–775] vs. 800 ml [300–1500], p=0.022) and operative time was shorter (235 min [163–285] vs. 281 min [223–349], p=0.032) in GA. Resection margin status and postoperative, including liver-specific, outcomes were comparable between the two techniques.

**Conclusion:**
GA resulted in reduced intraoperative blood loss and shorter operative time compared with DA, without differences in postoperative outcomes.

DE GRUYTER BRILL

**Seeking the best parenchymal transection device for MILS systematic review and Network Meta-Analysis**
ID: E-DCK-499

**Viszeralchirurgie / Visceral Surgery**
**Minimalinvasive Chirurgie (CAMIC) / Minimally Invasive Surgery (CAMIC)**

Simon-Alexander Kammer[1], Felicia Kneifel[1], Carsten Szardenings[1], Victor Fu[1], Shadi Katou[1], Andreas Andreou[1], Haluk Morgül[1], Benjamin Strücker[1], Andreas Pascher[1], Felix Becker[1]

[1]Universitätsklinik Münster, Klinik für Allgemein-, Viszeral- und Transplantationschirurgie, Münster, Deutschland

**Background:**
Minimally invasive liver surgery (MILS), including laparoscopic and robotic approaches, has become an established alternative to open hepatectomy, offering reduced morbidity, blood loss, and hospital stay. Parenchymal transection, however, remains a pivotal and technically demanding step. A wide range parenchymal transection devices, such as CUSA, ultrasonic scalpel, monopolar and bipolar instruments, and water jet dissectors, are in use, yet no consensus exists on the optimal technique. This network meta-analysis aimed to systematically compare parenchymal transection methods in MILS regarding intraoperative performance and postoperative outcomes to provide evidence-based recommendations for device selection.

**Material and methods:**
A PRISMA-compliant systematic search identified all English-language studies on MILS published up to 2025, excluding transplant hepatectomy. Parenchymal transection devices were categorized into eight groups (crush-clamp, CUSA, stapler, ultrasonic scalpel, radiofrequency sealing device, water jet, monopolar electrocautery device, and bipolar radiofrequence device). Perioperative outcomes (hospital stay, blood loss, operative time, conversion rate, bile leak and complications) were evaluated in a Bayesian arm-based network meta-analysis.

**Results:**
Overall, 58 studies comprising 3,985 patients who underwent robotic and laparoscopic liver resection were included. Of these, 33 studies investigated purely laparoscopic procedures, 21 focused on robotic resections, and four directly compared the two approaches. The largest groups were the ultrasonic scalpel group, reported in 30 studies with 995 patients, and the stapler group, investigated in four studies with 1,076 patients. The smallest cohort comprised the combined use of bipolar and radiofrequency devices, which was evaluated in two studies, including a total of 64 patients. No significant differences were observed in any of the evaluated outcomes, including hospital stay, blood loss, operative time, conversion rate, bile leakage, and postoperative complications.

**Conclusion:**
Based on the findings of this network meta-analysis, no clear recommendation can be made regarding the optimal device for parenchymal transection during minimally invasive liver surgery. These results underscore the need for well-designed prospective studies to provide robust evidence and enable future generations of surgeons to make objective, evidence-based decisions rather than relying solely on institutional preferences or established local standards.

DE GRUYTER BRILL

**Gallstone Ileus without Evidence of a Cholecystoenteric Fistula – A Case Report**
ID: E-DCK-349

**Viszeralchirurgie / Visceral Surgery**
**Notfallchirurgie und Notfallmanagement (CAMIN) / Emergency Surgery and Emergency Management (CAMIN)**

Christian Benignus[1 2], Anna Merscher[1], Christoph Marquardt[1], Steffen Retter[1], Thomas Schiedeck[1]

[1]Klinikum Ludwigsburg, Klinik für Allgemein-, Viszeral-, Thorax- und Kinderchirurgie, Ludwigsburg, Deutschland, [2]Paracelsus Medical University Salzburg, Salzburg, Österreich

**Background:**
Gallstone ileus is a rare complication of cholelithiasis, occurring in 0.15–1.5% of patients and accounting for less than 0.1% of mechanical bowel obstructions. Typically, a bilioenteric fistula enables stone migration into the intestinal tract, most often between gallbladder and duodenum. Exceptionally, gallstones may pass via the common bile duct and papilla without fistula formation. We present an unusual case of gallstone ileus without evidence of a cholecystoenteric fistula.

**Material and methods:**
This case report was compiled from clinical data extracted from the hospital’s electronic medical records. The patient’s presentation, diagnostic imaging, surgical management, and postoperative course were reviewed and summarized to provide a concise overview of this rare condition.

**Results:**
A 65-year-old male patient presented with acute vomiting and abdominal pain. Clinical examination revealed localized tenderness and sparse bowel sounds. Laboratory tests showed elevated inflammatory markers and lactate. CT imaging demonstrated a gallstone in the terminal ileum with small bowel obstruction. Given the patient’s history of multiple prior laparotomies, an open surgical approach was chosen. Intraoperatively, four closely located ileal stenoses and a mobile gallstone proximal to these strictures were identified. Approximately 30 cm of ileum, including the stenotic segments, were resected and an end-to-end ileo-ileostomy performed. The stone measured 1.5 cm in diameter. No fistula or gallbladder pathology was found, supporting spontaneous passage of the stone via natural anatomical pathways. The postoperative course was uneventful, and the patient was discharged on day 8.

**Conclusion:**
Gallstone ileus is a rare entity usually associated with bilioenteric fistula formation. This case illustrates an unusual mechanism with spontaneous stone migration and subsequent obstruction due to pre-existing small bowel stenoses. Awareness of such atypical presentations is important to guide timely surgical management.

DE GRUYTER BRILL

**Complex Self-Inflicted Thoracoabdominal Injuries with Scissors in a Patient with PTSD: A Case Report**
ID: E-DCK-483

**Viszeralchirurgie / Visceral Surgery**
**Notfallchirurgie und Notfallmanagement (CAMIN) / Emergency Surgery and Emergency Management (CAMIN)**

Baco Stanko[1], Mitric Milos[1]

[1]Public General Hospital "Dr Mladen Stojanovic", Prijedor, Bosnia and Herzegovina

**Background:**
Suicide attempts by self-stabbing are rare, particularly when combined with bilateral venous lacerations of the extremities and multiple penetrating thoracoabdominal injuries. Such cases are scarcely reported in the literature, and they represent a major therapeutic challenge. This report highlights the importance of the rudimentary yet effective diagnostics, and rapid surgical intervention. Despite the severity of the injuries, prompt operation can be life-saving.

**Material and methods:**
The patient was a 63-year-old male with a history of post-traumatic stress disorder (PTSD), paralysed for 20 years due to war-related injuries, and experiencing severe constipation. His dependence on his wife for care and dressing led to melancholy, diminished self-confidence, and a feeling of dishonour. He was hospitalised in a state of shock, unconscious, and haemodynamically unstable, with incisions on the inner aspects of both wrists and both lower extremities veins. Furthermore, he sustained stab wounds inflicted by huge scissors to the chest, namely in the left precordial region, along with two stab wounds to the belly, one of which was a laparotomy incision in the lower left quadrant, orientated transversely and measuring approximately 16 cm in length, accompanied by a protrusion of small bowel loops.
After basic diagnostics, comprising a chest X-ray and a complete blood count, the patient underwent emergency surgery. During the operation, the patient received two units of packed red blood cells. A left thoracic drain was placed producing no more than 200 ml of blood. A midline laparotomy was performed showing no substantial intra-abdominal haemorrhage. The sigmoid colon sustained injury, necessitating the execution of a Hartmann procedure. The patient was admitted to the intensive care unit postoperatively.

**Results:**
The postoperative period was unremarkable. The wound healed without complications. The patient was hospitalised for seven days and was discharged without complications. During the first postoperative examination, the stoma was functioning, the wound appeared normal, and the patient was in a positive mood. Notably, there was a marked enhancement in mood; once he was capable of managing his private hygiene, notably concerning his stool, he underwent a transformation, and his feelings of depression completely disappeared.

**Conclusion:**

Combined injuries caused by scissors — including venous lacerations, penetrating stab wounds to the chest, and abdominal laparotomy wounds — are extremely rare and potentially lethal." immediate attention is necessary, and surgeries in these situations are frequently carried out as damage-control or life-saving operations.  Unexpectedly, in this case, after the patient regained control over her stool production and personal hygiene, there was a substantial rise in self-confidence, resulting in the complete remission of depression and the development of a renewed positive perspective on life.

**Training Level Matters: Learning Curve Analysis with the HUGO™ RAS System**
ID: E-DCK-137

**Viszeralchirurgie / Visceral Surgery**
**Passion, Training, Weiterbildung in der Viszeralchirurgie (CAJC) / Passion, Training and Continuing Education in Visceral Surgery (CAJC)**

Stefanie Brunner[1], Dean Müller[2], Dolores Krauss[1], Rabi Raj Datta[1], Jennifer Eckhoff[1], Thomas Schmidt[1], Christiane J. Bruns[1], Hans Fuchs[1]

[1]Uniklinik Köln, Klinik und Poliklinik für Allgemein-, Viszeral-, Thorax- und Transplantationschirurgie, Köln, Deutschland, [2]Universität zu Köln, Medizinische Fakultät, Köln, Deutschland

**Background:**
Robotic-assisted surgery has become an integral part of minimally invasive procedures, offering improved visualization, dexterity, and surgical precision. The introduction of new platforms such as Hugo™ Robotic-Assisted Surgery (RAS) System requires evaluation of their implementation and training needs. A key aspect of adoption is the learning curve, which reflects the number of procedures required to achieve proficiency. Understanding this curve is essential for structured training, quality assurance, and optimizing patient outcomes. This study investigates the learning curve of the Hugo™ RAS System among participants with varying levels of robotic experience.

**Material and methods:**
This prospective, single-center study assesses the learning curve of the Hugo™ RAS System using a simulation-based training program. Fifty-three participants were divided into four groups—laypeople, medical students, surgical residents, and experienced robotic surgeons—based on their surgical background. All underwent standardized training on the robotic console, followed by three repetitions of simulation tasks (Peg Board 1, Rope Walk, and Ring Walk 1). These tasks were designed to evaluate basic robotic skills. Objective performance metrics such as task completion time, motion economy, force application, instrument collisions, and drops were automatically recorded for each trial. Learning curves were analyzed by tracking changes across repeated exercises. Data were analyzed descriptively, and the study was approved by the local ethics committee.

**Results:**
Laypeople showed the longest completion times with high variability, often exceeding 60 seconds, while medical students demonstrated shorter times on average but still displayed wide ranges. Surgical residents achieved faster and more consistent times, typically clustering around 30–40 seconds. The expert group recorded the shortest completion times overall, with minimal variation, and consistently outperformed the other groups.
Regarding the **metric score**, laypeople achieved the lowest median values, with broad dispersion across individuals. Residents showed further improvement and greater consistency. Expert surgeons achieved the highest metric scores with the narrowest distribution, indicating both proficiency and reproducibility of performance.
For **economy of motion**, laypeople and medical students exhibited the highest values, reflecting inefficient and excessive instrument movements. Residents demonstrated marked improvements with every task completed, while experts consistently achieved the lowest economy of motion values, reflecting precise and efficient handling.
With regard tot he learning effects, residents required an average of **109 seconds** in the first repetition of the rope walk exercise, followed by **105 seconds** in the second trial. In the third repetition, their average completion time further decreased to **90 seconds** (Figure 1).

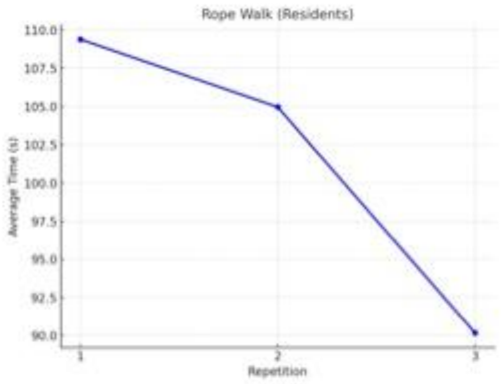

**Conclusion:**
Our findings demonstrate a clear, experience-dependent gradient in robotic surgical performance, with laypeople showing the slowest, least precise, and least efficient task execution, and expert surgeons achieving the fastest, most accurate, and most economical performance. Progressive improvements observed across medical students and residents underscore the impact of prior surgical training on speed, precision, and instrument efficiency. These results highlight the critical role of structured, simulation-based training in accelerating skill acquisition, promoting reproducibility, and minimizing unnecessary movements before live surgical application. Incorporating such training programs, along with mentorship and proficiency-based progression, can effectively shorten learning curves, enhance operative performance, and support safer adoption of robotic surgery across institutions.

DE GRUYTER BRILL

### Impact of sleep deprivation on laparoscopic performance and skill acquisition: the SLEEPLESS Trial

ID: E-DCK-259

**Viszeralchirurgie / Visceral Surgery**
**Passion, Training, Weiterbildung in der Viszeralchirurgie (CAJC) / Passion, Training and Continuing Education in Visceral Surgery (CAJC)**

Wilma Werner[1], Eleni Felinska[1], Felix Müller[1], Marielle Schobbert[1], Emre Karakas[1], Frederic Dürr[1], Felix Nickel[2], Amila Cizmic[2], Philipp Romero[3], Franck Billmann[1], Frank Pianka[1]

[1]Department of General, Visceral and Transplantation Surgery, Heidelberg University Hospital, Heidelberg, Deutschland, [2]Department of General, Visceral and Thoracic Surgery, University Medical Centre Hamburg-Eppendorf, Hamburg, Deutschland, [3]Division of Pediatric Surgery, Department of General, Visceral and Transplantation Surgery, Heidelberg University Hospital, Heidelberg, Deutschland

**Background:**
Sleep deprivation and its effect on physical and mental health, as well as concentration and psychomotor skills is a known problem for surgeons and surgical residents. However, little is known about its impact on medical students at the beginning of their surgical training. This trial investigated the influence of acute sleep loss on performance and learning of students undergoing minimally invasive surgery training in a virtual reality (VR) simulation setting.

**Material and methods:**
In this single-center randomized controlled trial, 40 medical students were allocated either to a sleep deprivation group (24 h awake) or the control group (regular sleep). All participants first completed a session of seven basic laparoscopic VR tasks, followed by a second session with the same basic tasks and three repetitions of a laparoscopic Roux-en-Y gastric bypass (LRYGB) with the Simbionix LAP Mentor simulator. Performance metrics included task completion time, instrument path length, error parameters, and procedure-specific OSATS and GOALS scores. Secondary outcomes included mental health, attention anf sleep health and were assessed with the d2-R test of attention, the NASA-TLX, and the GSE-3 scale.

**Results:**
After basic training, there were no significant differences between groups except for improvement in task A3 (cutting time), which was greater in the control group (–24.0 ± 23.2 s) than in the sleep-deprived group (–4.3 ± 18.3 s; $p = 0.021$). During the LRYGB, both groups improved significantly between the first and third attempt. In step 1 (gastric pouch creation), completion times decreased by –434.9 ± 305.2 s in the control group and –424.8 ± 240.9 s in the sleep-deprived group ($p = 0.552$), while instrument path length tended to remain longer in the sleep-deprived group ($p = 0.088$). Median OSATS 1 improvement was +13.0 (IQR 6.3–14.8) in controls and +7.0 (IQR 0.0–14.0) in the sleep-deprived group ($p = 0.222$). OSATS 2 (+3.4 ± 4.2 vs. +3.4 ± 3.6; $p = 0.957$) and GOALS (+2.4 ± 2.3 vs. +2.1 ± 2.5; $p = 0.537$) improved comparably. Secondary outcomes showed similar trends: concentration performance (d2-R) increased by +4.8 ± 4.3 vs. +3.4 ± 5.4 ($p = 0.497$), workload (NASA-TLX) rose by +17.9 ± 13.5 vs. +16.9 ± 9.2 ($p = 0.925$), and self-efficacy (GSE-3) decreased slightly (–0.8 ± 2.6 vs. –2.2 ± 3.2; $p = 0.239$).

**Conclusion:**
Laparoscopic novices improved substantially in laparoscopic performance during VR training, regardless of sleep deprivation. Observed differences between groups were small and inconsistent, suggesting that one night of sleep loss did not substantially limit short-term learning progress.

DE GRUYTER BRILL

**Integrating Laparoscopic Skills Training into Surgical Routine: A Simplified Open-Box Concept**
ID: E-DCK-33

**Viszeralchirurgie / Visceral Surgery**
**Passion, Training, Weiterbildung in der Viszeralchirurgie (CAJC) / Passion, Training and Continuing Education in Visceral Surgery (CAJC)**

Mukhammad Davliatov[1], Ludger Staib[1], Clemens Poth[1], Florian Schilcher[1]

[1]Klinikum Esslingen, Allgemein- und Viszeralchirurgie, Esslingen, Deutschland

**Background:**
Minimally invasive surgery (MIS), particularly laparoscopy, is an essential part of visceral surgery. Due to its technical complexity, it demands refined surgical skills. Common challenges such as limited field of view, the fulcrum effect, reduced depth perception, limited haptic feedback, and demanding visual-motor coordination increase the learning curve. Studies suggest that up to 200 procedures may be required to safely master complex laparoscopic procedures such as hemicolectomy [1]. This is often unachievable for surgical trainees during residency.
Simulation-based training (e.g., Lübeck Toolbox, FLS program, LapSim®, LAP Mentor®) effectively reduces complication rates and accelerates learning [2–4]. However, these systems are expensive, technically demanding, and difficult to integrate into everyday clinical routines. Neurophysiological evidence supports the superiority of regular, distributed training over sporadic intensive sessions for developing fine motor skills.

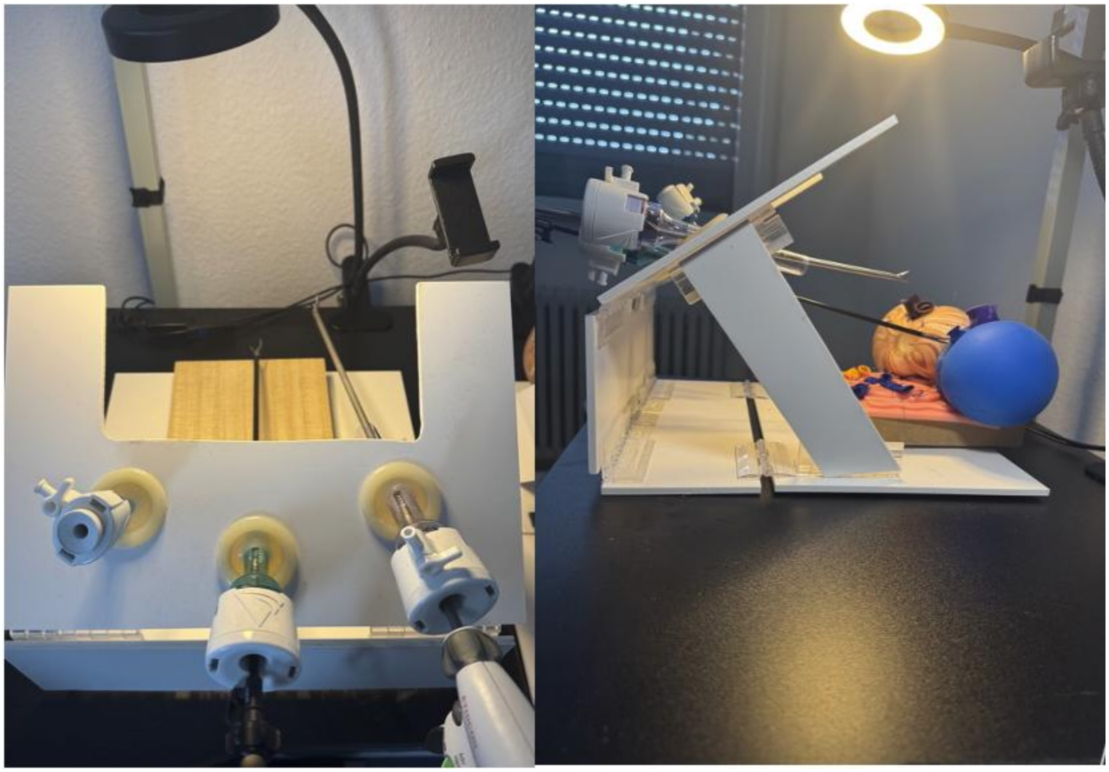

**Material and methods:**
The aim of this study was to design a cost-effective, low-threshold, and schedule-compatible training concept suitable for surgical trainees at Klinikum Esslingen. Through testing commercial and self-built trainer boxes, we identified practical thresholds that hinder consistent training. Commercial systems, such as the Lübeck Toolbox, required 3–5 minutes to set up and showed suboptimal image quality despite high costs. These barriers significantly reduced usability in everyday routines.
With financial support from "Förderverein ProKlinikum Klinikum Esslingen," we developed a custom trainer box using polystyrene panels and acrylic hinges. The setup mimics standard trocar placement and includes a large opening that allows direct visualization of the training surface - eliminating the need for camera, monitor, or computer (Fig. 1). This simplicity enables surgical trainees to complete short training sessions spontaneously during free moments.
*Fig. 1: Our custom-built trainer allows direct line-of-sight access to the working area for simple, spontaneous practice.*
**Training Methodology:**
Validated programs such as the FLS and Lübeck Toolbox were quickly mastered by trainees, which led to decreased motivation for repetition. To address this, we developed a flexible training concept that allows variation in difficulty and task design to maintain engagement. Each exercise targets a specific laparoscopic challenge (Table 1).
*Table 1. Challenges in Laparoscopy – and Targeted Solutions:*

| **Challenge** | → Targeted Training Solution |
|---|---|
| **Fulcrum effect** | → Repetition-based motion under constrained conditions |
| **Limited range of motion** | → Box-constrained tasks to improve control |
| **Bimanual coordination** | → Developed via origami and suturing tasks |
| **Depth perception limitations** | → Practicing under direct vision for spatial awareness |
| **Visual field restriction** | → Awareness training without camera reliance |
| **Reduced tactile feedback** | → Visual cue-based tissue manipulation |
| **Stress & multitasking** | → Frequent, low-pressure repetition enhances resilience |

The program is based on three structured, repeatable exercises:
**1. Origami folding (e.g., crane)**
→ Trains bimanual coordination, fine tissue-sparing technique through visual cue-based manipulation, spatial awareness, and fine motor control.

→ The difficulty level of the training can be adjusted by using origami folding paper of different sizes (Fig. 2).
*Fig. 2: Origami training using standard laparoscopic tools and folding paper of different sizes to adjust task difficulty.*

**2. Precise Needle Handling**
→ Developed using inexpensive structured surfaces (e.g., silicon mats, balloons, stress balls).
→ Needle maneuvers range from simple linear motions to complex anastomoses with varying diameters (Fig. 3)
*Fig. 3: Balloon segments and structured silicone mats simulate tubular and elastic anatomical structures. The setup allows trainees to practice needle insertion, angulation, and precision suturing in a realistic and variable environment.*

**3. Intracorporeal Knot Tying**
→ Performed on structured surfaces at various angles and depths using everyday materials (Fig. 4).
*Fig. 4: Structured surface for laparoscopic knot tying with variable angles*
Each task is designed for 10–15 minutes of training and can be performed independently, without supervision. The direct visual access eliminates setup time and facilitates frequent short sessions of low-pressure practice.

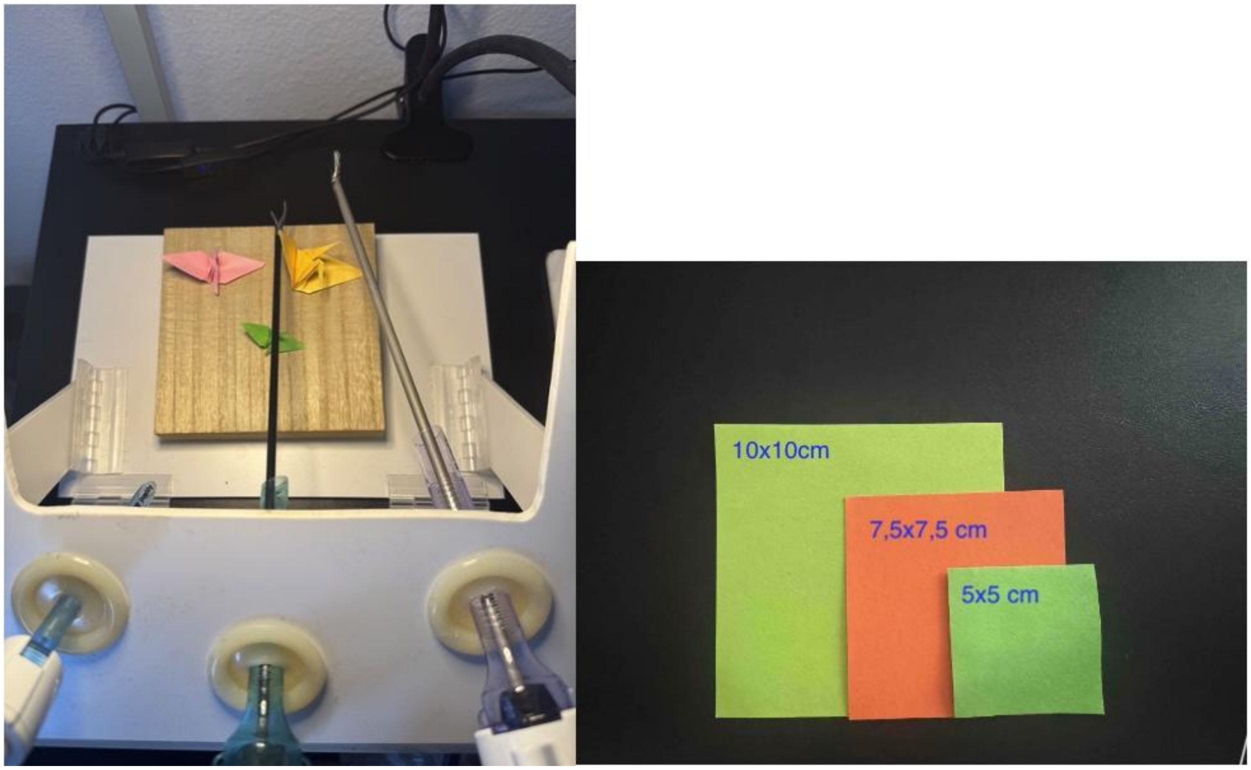

**Results:**
**Planned Evaluation:**
We plan a prospective study involving surgical residents. Each participant will perform a laparoscopic cholecystectomy before and after a 4-week training phase. Video recordings will be assessed using validated scoring tools (e.g., OSATS), and objective parameters such as operative time and error rates will be documented.

**Evidence Base:**

- **Origami improves operative performance**: Noda et al. showed significantly shorter pneumoperitoneum times in the origami-trained group during laparoscopic kidney surgery (129 vs. 208 min;*p* < 0.001) [5].
- **Competitive origami training enhances speed & accuracy**: The "Kaminote Challenge" demonstrated superior performance in those with >1,000 folded cranes [6].
- **Simulation training improves real surgical outcomes**: Multiple meta-analyses confirm improvements in operative time, technical error rates, and validated OSATS/GOALS scores post-training [3,4,7].

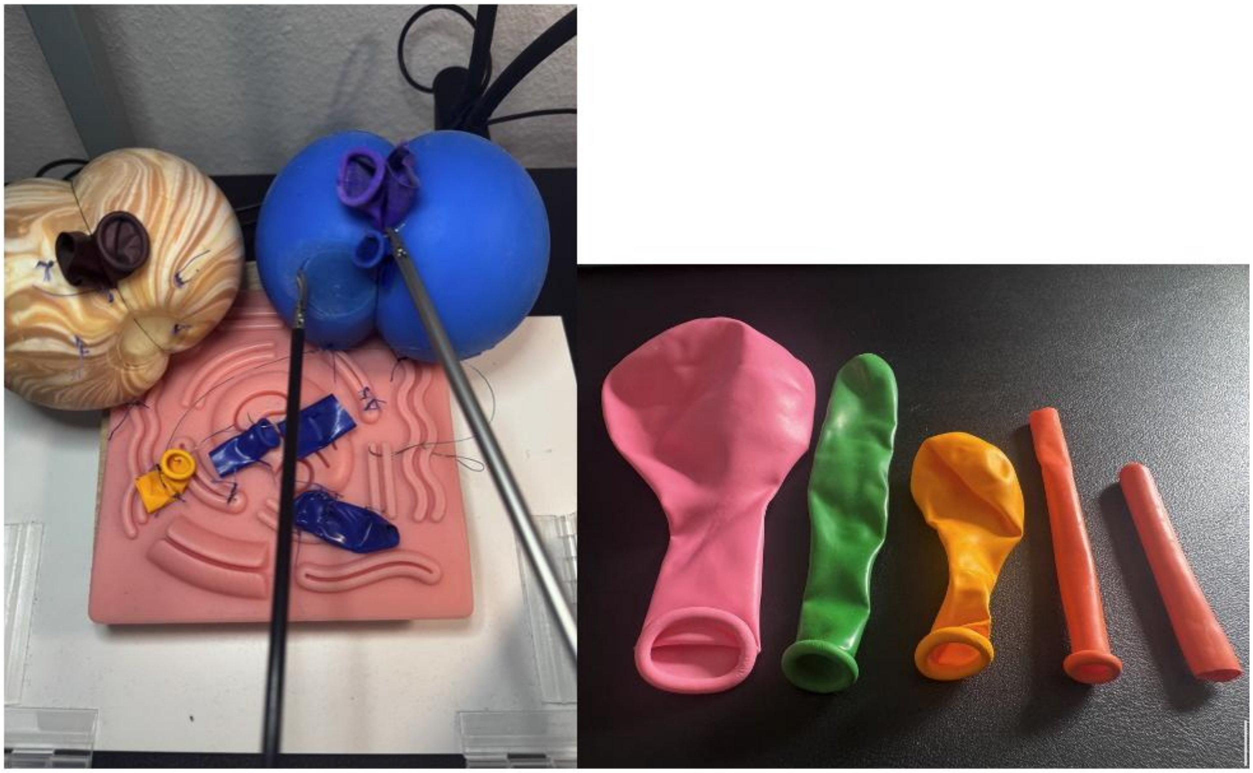

**Conclusion:**

Our proposed concept enables an affordable, time-efficient, and neurophysiologically sound training method for laparoscopic skills. The custom trainer allows frequent, spontaneous use in clinical routines without digital infrastructure. By focusing on key MIS competencies through simplified, repeatable tasks, the program addresses real-world constraints while promoting safe and skillful surgical practice. A pilot study is currently underway to validate its clinical transferability.

**References:**

1. Böttger TC et al. (2011). "Laparoscopic colon resections – learning curve and outcomes."
2. Laubert T. et al. (2015). "The Lübeck Toolbox: Validation of a structured MIC training."
3. Sroka G. et al. (2010).*Ann Surg*, 252(1): 37–42.
4. Seymour NE et al. (2002).*Ann Surg*, 236(4): 458–463.
5. Noda M. et al. (2024).*Cureus*, 16(2): e62098. https://doi.org/10.7759/cureus.62098

6. Noda M. et al. (2024). *Cureus*, 16(3): e72014. https://doi.org/10.7759/cureus.72014
7. Buckley CE et al. (2014). "Meta-analysis of simulation-based training in laparoscopic surgery."

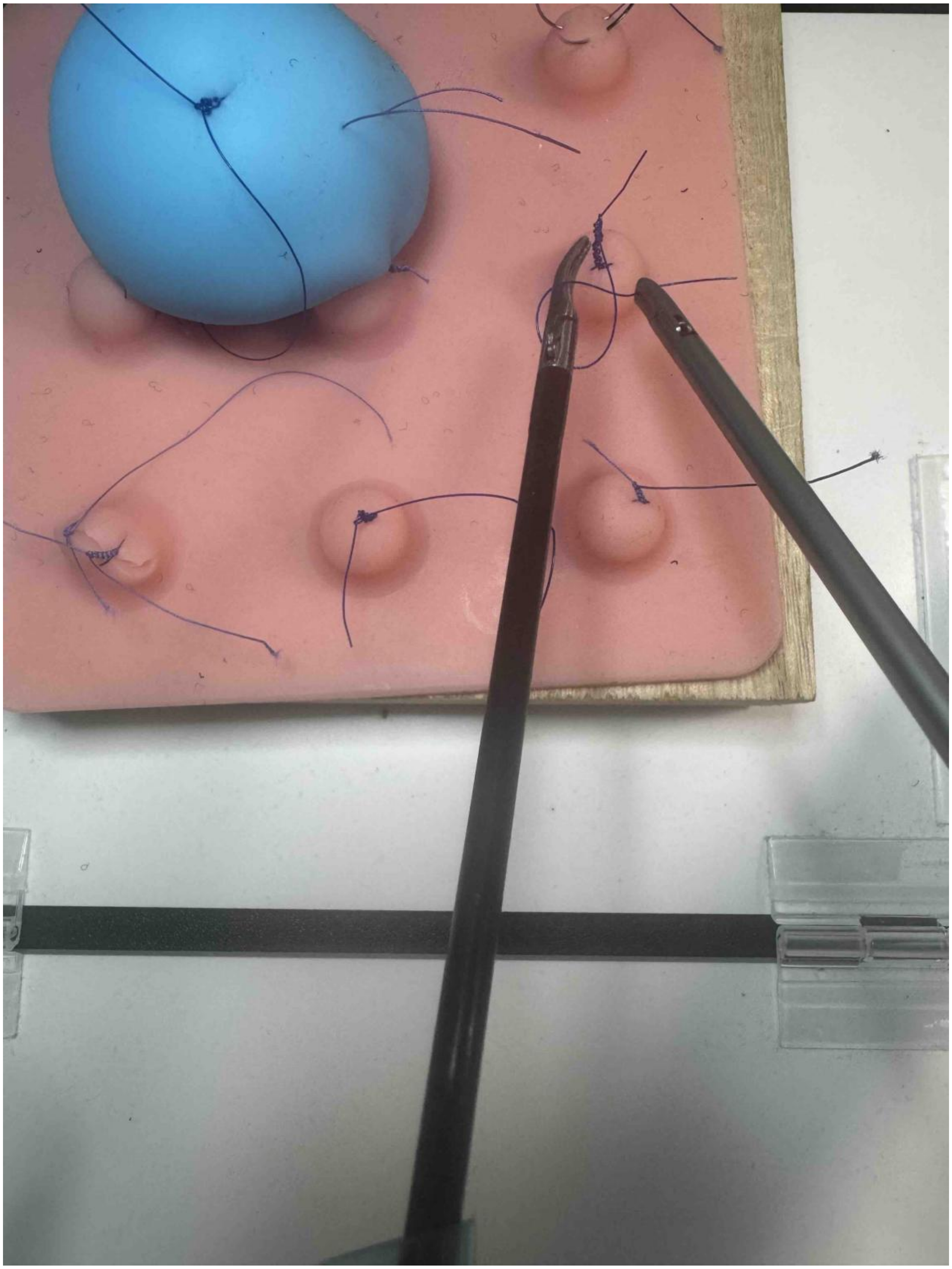

DE GRUYTER BRILL

**Learning from Errors in Surgery: Trainees' Perceptions on Morbidity and Mortality Meetings**
ID: E-DCK-93

**Viszeralchirurgie / Visceral Surgery**
**Passion, Training, Weiterbildung in der Viszeralchirurgie (CAJC) / Passion, Training and Continuing Education in Visceral Surgery (CAJC)**

Hélène Gros[1], Jessica Düblin[1 2], Nadshathra Varathan[3], Stephanie Taha-Mehlitz[1], Suna Erdem[1], Adrian Billeter[1], Beat Müller[1], Jennifer Klasen[1]

[1]Clarunis - University Digestive Health Care Center Basel, Department of Visceral Surgery, Basel, Schweiz, [2]Spital Rheinfelden, Medizinische Klinik, Rheinfelden, Schweiz, [3]Kantonsspital Olten, Departement Chirurgie, Olten, Schweiz

**Background:**
Morbidity and Mortality Meetings (M&Ms) provide opportunities to review adverse events, share experiences, and promote patient safety through collective learning. However, their structure still varies across institutions. Additionally, undergraduate medical curricula rarely include preparation for attendance or participation in M&Ms. This research aimed to explore if M&Ms fulfill their intended educational goals focusing on the perspectives of surgical learners', medical students on clinical rotation and residents.

**Material and methods:**
We observed 18 M&Ms in three surgical teaching hospitals in Switzerland and field notes were taken during the meetings. Following each M&Ms, we conducted 1-3 semi-structured interviews, for a total of 32 (15 residents and 17 medical students). The interview guide explored learners' expectations, experiences, perceived barriers, and reflections on the role of M&Ms. Using a constructivist grounded theory approach, data were analyzed using software-assisted coding, constant comparison, and categorization to develop themes.

**Results:**
Of the 32 participants, 47% were residents and 53% medical students. With one exception, all had studied in Swiss or German medical universities. From the semi-structured interviews, five key themes were developed. (1) <u>M&Ms as a suboptimal learning space</u> due to time constraints and seating arrangements that mirrored surgical hierarchy, reinforcing passive attendance. (2) <u>Lack of preparation</u> as most students reported attending their first M&M without prior introduction or guidance. They described not knowing what their role should be, or how to engage. As a result, they remained silent observers. Residents, although more familiar with the format, also described uncertainty about how they could contribute. (3) <u>Learning gains evolved through attendance</u>: medical students first distanced themselves intellectually when confronted with errors, whereas residents distanced themselves emotionally when complications carried potential personal responsibility. After reflection, however, M&Ms were seen as a valuable forum to discuss and normalize errors. (4) <u>Trainees noticed horizontal violence.</u> These confrontational exchanges were a barrier to trainees' learning. Therefore, trainees underscored the importance of a supporting team culture. (5) Finally, <u>the chief surgeon</u> consistently emerged as a pivotal educational leader, shaping tone, meaning, and value of the sessions.

**Conclusion:**

Although the explored M&Ms met their goal of facilitating the discussions on errors, improvements are still needed from learners' perspectives. Suggested improvements include small structural changes (e.g., seating arrangements, more time, greater interaction) and targeted interventions such as workshops or mock M&Ms to prepare learners. Additionally, promoting a supportive peer culture early and discouraging horizontal violence may better equip future surgeons to handle the emotional toll of complications. Finally, ensuring that moderators focus on errors analysis and causative factors is crucial to support a learning environment.

DE GRUYTER BRILL

**Gender-specific impact of body composition on the prognosis of colorectal liver metastases**
ID: E-DCK-166

**Viszeralchirurgie / Visceral Surgery**
**Perioperatives Management, Ernährung, Risikoreduktion (CA PeriVis) / Perioperative Management, Nutrition and Risk Reduction (CA PeriVis)**

Marc Reschke[1], Mathias Holtkamp[2], Alexandra Sacu[1], Daniel Heise[1], Jan Bednarsch[1], Tom Florian Ulmer[1], Arzu Özcelik[1], Johannes Haubold[2], Ulf Neumann[1], Sven Lang[1], Sophia Schmitz[1]

[1]Klinik für Allgemein-/Viszeral-/Gefäß- und Transplantationschirurgie, Universitätsklinikum Essen, Essen, Deutschland, [2]Institut für diagnostische und interventionelle Radiologie und Neuroradiologie, Universitätsklinikum Essen, Essen, Deutschland

**Background:**
Body composition has emerged as a critical factor influencing outcomes in various malignancies, yet its impact in the context of colorectal liver metastasis (CRLM) resection remains incompletely understood, especially regarding sex-specific differences.

**Material and methods:**
In this retrospective study, we analyzed patients who underwent liver resection (including re-resections) for CRLM between 2019 and 2024. We evaluated the prognostic value of sarcopenia indices derived by fully volumetric body composition analysis based on computed tomography (CT) imaging. The following indices were determined: Sarcopenia Index (SI = Muscle volume [mL] / Bone volume [mL]), Fat Index (FI = Total Adipose Tissue [mL] / Bone volume [mL]), Myosteatotic Fat Index (MFI = Intra- and intermuscular adipose tissue [mL] / Total Adipose Tissue [mL]), and Abdominal Fat Index (AFI = Visceral Adipose Tissue [mL] / Subcutaneous Adipose Tissue [mL]).

**Results:**
A total of 155 patients were included. The median body mass index (BMI) differed between male (27 kg/m$^2$, IQR 24 - 31) and female patients (25 kg/m$^2$ IQR 20 - 29; p<0,05). Compared to men, women exhibited less sarcopenia (SI: 2.3 [CI 2.1−2.6] vs. 2.6 [CI 2.4−2.9]; p<0.001) and less abdominal fat (AFI: 0.3 [CI 0.2−0.4] vs. 0.6 [CI 0.5−0.8]; p<0.001).
FI as well as MFI showed no significant sex differences. The five-year overall survival rate was 40%, with no significant difference between sexes. Sarcopenia also did not influence overall survival. However, in multivariate analysis, a higher MFI was found to be protective against recurrence in men (OR 0,83 [CI 0.72 – 0.95]; p < 0.05).

**Conclusion:**
Our findings suggest that myosteatosis may decrease the risk of recurrence in men. Therefore, a preoperative, sex-specific body composition assessment may have a prognostic role for CRLM resection.

DE GRUYTER BRILL

### Immunonutrition in Minimally Invasive Abdominal Surgery: Meta-Analysis of Randomized Clinical Trials

ID: E-DCK-20

**Viszeralchirurgie / Visceral Surgery**
**Perioperatives Management, Ernährung, Risikoreduktion (CA PeriVis) / Perioperative Management, Nutrition and Risk Reduction (CA PeriVis)**

Matthias Pfister[1], Nadja von Massow[2], Eva Kalkum[2], Pia Antony[1], Alexander Dullenkopf[3], Fabian Hauswirth[4], Pietro Renzulli[4], Markus Müller[1], Pascal Probst[1]

[1]Spital Thurgau AG, Kantonsspital Frauenfeld, Klinik für Chirurgie, Frauenfeld, Schweiz, [2]Studienzentrum der Deutschen Gesellschaft für Chirurgie (SDGC), Heidelberg, Deutschland, [3]Spital Thurgau AG, Kantonsspital Frauenfeld, Institut für Anästhesie, Frauenfeld, Schweiz, [4]Spital Thurgau AG, Kantonsspital Münsterlingen, Klinik für Chirurgie, Münsterlingen, Schweiz

**Background:**

Immunonutrition is recommended in major abdominal surgery because of its reduced rates of infectious and overall complications, as well as shorter length of hospital stay (LOS). However, evidence is sparse on its effect in minimally invasive surgery (MIS). Given the widespread adoption of MIS as the standard of care for most abdominal procedures, there is a critical need to examine the evidence for immunonutrition in this setting. Therefore, this study aimed to assess the effect of immunonutrition on postoperative outcomes in minimally invasive major abdominal surgery.

**Material and methods:**

This systematic review and meta-analysis were conducted according to PRISMA guidelines. CENTRAL, PubMed, EMBASE, and Web of Science were systematically searched (last update: May 28, 2025). All randomized clinical trials (RCTs) investigating immunonutrition versus standard nutrition in patients undergoing minimally invasive major abdominal surgery were included. Mortality, overall and infectious complications, and LOS were analyzed using odds ratios (OR) or mean differences (MD) in a random-effects model. Sensitivity analysis was conducted excluding all studies at overall high risk of bias. Subgroup analyses were performed for different immunonutrition protocols (nutrient, route and time of administration), nutritional state, type of disease, surgical site, and funding source. Risk of bias (Cochrane 2.0) and certainty of evidence (GRADE) were assessed.

**Results:**

Eleven RCTs comprising 890 patients were included. The median sample size across studies was 62 patients (interquartile range: 30-124). All but one RCT had two study arms. A total of 429 patients were analyzed in the immunonutrition and 428 patients in the control group. The control group consisted of normal oral or enteral diet in five studies, protein-enriched oral nutritional supplements in three studies, and low-fiber and low-caloric diet in one study each. One study used intravenous saline as placebo control.

**Risk of Bias Assessment**

The overall risk of bias was high in eight RCTs, with some concerns in two and low in one. Assessment of publication bias was not performed due to the inclusion of fewer than 10 studies per outcome, precluding reliable interpretation of asymmetry.

**Quantitative Analysis of Postoperative Outcomes**
Quantitative results on postoperative outcomes are summarized in Figure 1. Immunonutrition may not reduce mortality (OR 1.01, 95%-CI: 0.2 to 5.08, p=0.99, $I^2$= 0%, GRADE: *low*). It may have little to no effect on overall (OR 1.25, 95%-CI: 0.65 to 2.4, p=0.5, $I^2$= 60%, GRADE: *very low*) or infectious complications (OR 0.75, 95%-CI: 0.44 to 1.28, p=0.3, $I^2$= 32%, GRADE: *very low*), and LOS (MD +0.03 days, 95%-CI: -0.65 to +0.71, p=0.93, $I^2$= 47%, GRADE: *very low*) but the evidence is very uncertain. The results remained unchanged after excluding eight RCTs at overall high risk of bias.
**Subgroup Analysis**
In studies restricted to patients with malignant disease, the evidence suggests that immunonutrition results in little to no difference in outcomes. There were not enough data to analyze the subgroup of exclusively malnourished patients. Subgroup analysis by surgical site suggests for patients undergoing UGI surgery that immunonutrition may result in a reduction in infectious complications (OR 0.53, 95%-CI: 0.29 to 0.96; p = 0.04; $I^2$ = 0 %) and may reduce LOS (MD -1.19 days, 95%-CI: -2.07 to -0.32; p = 0.01; $I^2$ = 0 %) but the evidence is very uncertain. Regarding the source of funding (industry, non-industry), the evidence suggests that immunonutrition results in little to no difference in outcome in any subgroup.

**Certainty of Evidence**
The certainty of evidence was assessed for each outcome individually. The overall certainty of evidence for mortality was downrated to *low* because of high risk of bias and imprecision. The overall certainty of evidence for overall and infectious complications as well as LOS was rated *very low*. Reasons for downrating were high risk of bias, inconsistency in results, and imprecision. Excluding studies at high risk of bias lead to increased overall certainty across all outcomes with unchanged results.

**Conclusion:**
In conclusion, the beneficial effects of immunonutrition observed in open major abdominal surgery do not appear to be present in the minimally invasive approach. The current evidence particularly for high-risk surgical subgroups remains insufficient to draw reliable conclusions. Therefore, well-powered, rigorously conducted RCTs focusing on nutritionally at-risk patients are warranted to define patient-specific benefit. Until such high-quality evidence emerges, the continued use of immunonutrition in contemporary minimally invasive abdominal surgical cohorts is not justified.

DE GRUYTER BRILL

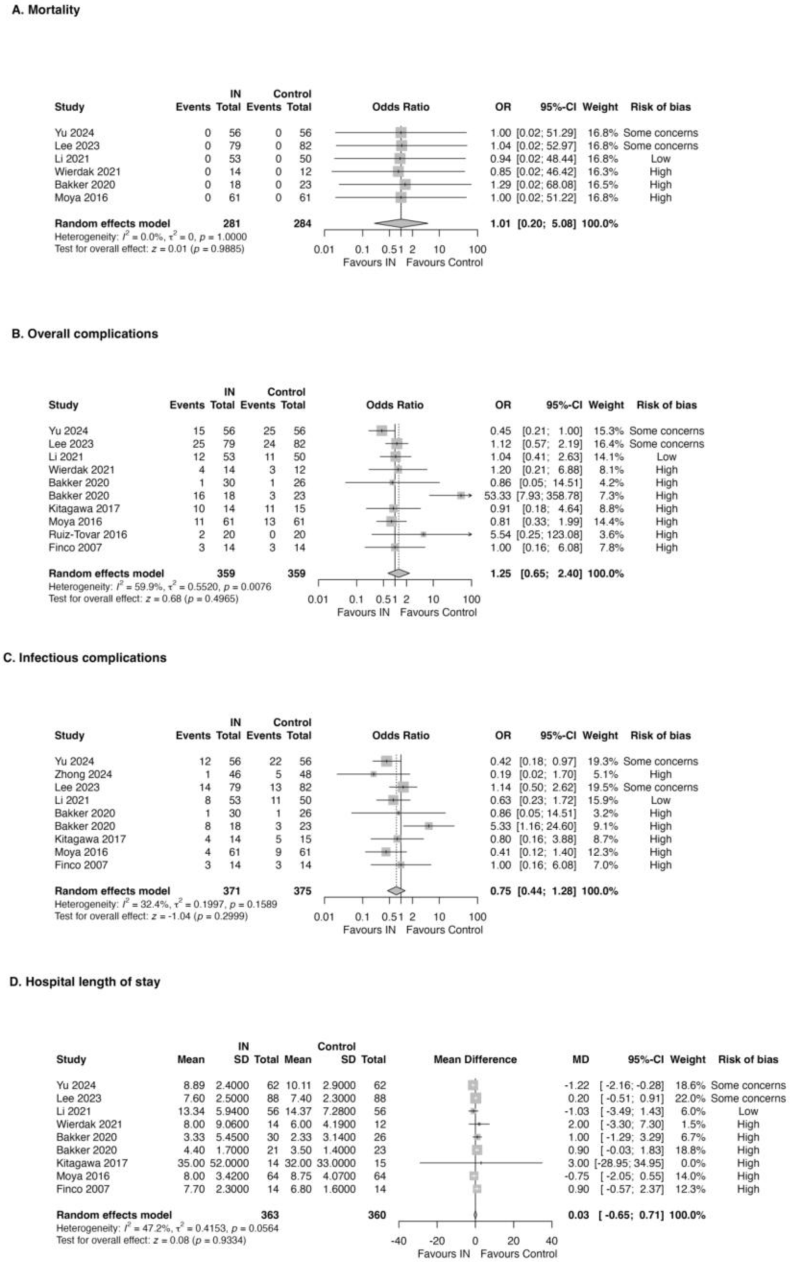

**Figure 1.** Meta-analysis of the effect of immunonutrition in minimally invasive major abdominal surgery. Forest plots summarizing the effect of immunonutrition (IN) versus control on (A) postoperative mortality, (B) overall complications, (C) infectious complications, (D) hospital length of stay. Pooled estimates are displayed as odds ratios (OD) for binary outcomes and mean differences (MD) for continuous outcomes with corresponding 95% confidence intervals (CI). Random-effects models were used for analyses.

DE GRUYTER BRILL

**Impact of Frailty and Malnutrition on Postoperative Outcomes in Upper GI Surgery**
ID: E-DCK-362

**Viszeralchirurgie / Visceral Surgery**
**Perioperatives Management, Ernährung, Risikoreduktion (CA PeriVis) / Perioperative Management, Nutrition and Risk Reduction (CA PeriVis)**

Malte Lang[1], Olga Kopeleva[1], Thorben Fedders[1], Ben Gubitz[1], Julius Pochhammer[1], Benedikt Reichert[1], Thomas Becker[1]

[1]UKSH Kiel, Klinik für Allgemeine, Viszeral-, Thorax-, Transplantations- und Kinderchirurgie, Kiel, Deutschland

**Background:**
Frailty and malnutrition are increasingly recognized as key determinants of perioperative risk in oncological surgery. A national S3 guideline on the definition and screening of frailty is currently under development, while uniform standards are still lacking. Patients with tumors of the gastroesophageal junction are particularly vulnerable to malnutrition due to dysphagia.
Previous studies suggest that frailty may be associated with adverse postoperative outcomes. At the same time, prehabilitation is being discussed as a promising approach to improve resilience, yet evidence in upper GI surgery remains limited. As part of the implementation of a structured prehabilitation program, we established a comprehensive screening tool for frailty and malnutrition at our center. The aim of this analysis was to investigate whether these screening parameters correlate with clinically relevant outcomes after Ivor Lewis esophagectomy.

**Material and methods:**
Since 2024, all patients undergoing oncological upper GI surgery at our center have been systematically screened for frailty and malnutrition. Parameters include Clinical Frailty Scale (CFS), SARC-F, 2-minute step test, Short Physical Performance Battery, handgrip strength, one-leg balance, and Nutritional Risk Screening (NRS 2002). For this analysis, we included only patients with adenocarcinoma of the gastroesophageal junction who underwent Ivor Lewis esophagectomy.
Frailty was defined by fulfilling at least one of the following criteria: CFS ≥ 4, oral intake < 60 %, BMI < 18 kg/m², performance in the lowest quartile of either 2-minute step test or handgrip strength. Patients were classified as *frail* if ≥ 1 criterion applied, and as *non-frail* otherwise. Endpoints included anastomotic leakage, pneumonia, postoperative bleeding, reoperation, myocardial infarction, delirium, pulmonary embolism, postoperative mortality, Clavien-Dindo classification, ICU and overall length of stay, as well as type and duration of endoscopic therapy. Chi² or Fisher's exact tests were applied for categorical outcomes, Welch's t-tests for continuous variables.

**Results:**
A total of 45 patients (mean age 64.9 years; 88.9 % male, 11.1 % female) were analyzed. The most distinct difference was observed for endoscopic stent placement, which was considerably more frequent in non-frail patients (OR 0.22, 95 % CI 0.04–1.17, p = 0.089). Anastomotic leakage occurred numerically more often in the frail group (+6 %; OR 1.45, 95 % CI 0.32–6.61, p = 0.62), but did not reach significance.

All myocardial infarctions occurred exclusively in the frail group (12 % vs. 0 %). Frail patients had a prolonged ICU stay (+4 days on average, 9.8 vs. 5.7 days; p = 0.07) and longer overall hospital stay (+6 days on average, 25.1 vs. 19.2 days; p = 0.08). No relevant differences were observed for reoperations, pneumonia, delirium, pulmonary embolism, or Clavien-Dindo classification. The latter is prone to limited sensitivity, as even diagnostic endoscopies are automatically scored as grade IIIa, even if no pathologies are detected.

**Conclusion:**
Frailty and malnutrition were associated with prolonged ICU and hospital stays and with specific postoperative complications. The markedly lower rate of stent therapy in frail patients suggests that anastomotic leakages in this group are more severe and less amenable to endoscopic management via stent. A structured frailty screening is feasible in daily practice and allows the identification of vulnerable patients. The main limitation of this study is the small sample size, which restricts statistical power and leads to predominantly descriptive findings. A strength of the study is the highly uniform design: all patients were screened by the same unit, treated within a short time frame (2024–2025), and operated on by the same two senior surgeons, with identical perioperative processes, ensuring excellent internal comparability. Prospective studies with larger cohorts are warranted to confirm the prognostic impact of frailty and to evaluate the effectiveness of targeted prehabilitation in upper GI surgery.

DE GRUYTER BRILL

**Beyond Survival: Insights Into Quality of Life Following Surgery for Pancreatic Cancer**
ID: E-DCK-67

**Viszeralchirurgie / Visceral Surgery**
**Perioperatives Management, Ernährung, Risikoreduktion (CA PeriVis) / Perioperative Management, Nutrition and Risk Reduction (CA PeriVis)**

Benedikt Johannes Wagner[1], Laura Lemberger-Viehmann[2], Anda Mayer[2], Hans Jürgen Schlitt[1], Silke Härteis[3], Thiha Aung[2], Christina Hackl[1]

[1]University Hospital Regensburg, Department of Surgery, Regensburg, Deutschland, [2]Deggendorf Institute of Technology, Faculty of Applied Healthcare Science, Deggendorf, Deutschland, [3]University of Regensburg, Institute for Molecular and Cellular Anatomy, Regensburg, Deutschland

**Background:**
Pancreatic ductal adenocarcinoma (PDAC) is among the most aggressive solid tumors and ranks as the seventh leading cause of cancer-related death worldwide. Global incidence continues to rise, particularly in high-income countries, reflecting demographic shifts, lifestyle risk factors, and improved diagnostic capabilities. Curative treatment is limited to complete surgical resection, followed by adjuvant chemotherapy. New therapeutic concepts, including neoadjuvant regimens and targeted approaches, are increasingly explored. Despite these advances, prognosis remains poor, with a five-year survival of 10%.[1,2,3]
While survival data dominate clinical research, health-related quality of life (HRQoL) has emerged as an equally important outcome. Complete surgical resection remains the cornerstone of curative treatment in PDAC; however, not every technically feasible operation is oncologically meaningful, and patient-related factors must also be taken into account. Beyond tumor removal, patients frequently face complex postoperative challenges such as pain, weight loss, pancreatic exocrine insufficiency, diabetes, and psychological distress. These aspects highlight the importance of balancing oncologic benefit with postoperative recovery and long-term QoL. Furthermore, adjuvant chemotherapy, although crucial for disease control, adds additional toxicity.[4,5] However, systematic evidence describing HRQoL outcomes after PDAC resection remains scarce.[6]
This retrospective single-center study aimed to explore HRQoL in a small cohort of postoperative PDAC patients. By focusing on physical and psychological domains of HRQoL the study provides preliminary insights and generates hypotheses for future research. Although limited in size, it emphasizes the relevance of integrating HRQoL into postoperative follow-up and supportive care.

**Material and methods:**
We applied a retrospective cohort design within the framework of a currently running study on PDAC at the University Hospital Regensburg (UKR). Inclusion criteria of this study comprised histologically confirmed PDAC and surgical intervention performed with curative intent. We excluded patients with non-PDAC malignancies or incomplete follow-up. Ethics committees' approval and informed consent of the patient was secured.
Of 57 patients screened, 21 (36.8%) completed HRQoL assessments and were included. Seventeen patients (29.8%) had deceased by the time of this study, and another 17 (29.8%) declined participation. Two patients (3.6%) were excluded as the final diagnosis was not PDAC.

Data collection consisted of two questionnaires: a general survey addressing health status, comorbidities, and subjective well-being, and a PDAC-adapted version of the EORTC QLQ-C30 questionnaire. Additional variables such as age, sex, diabetes status, smoking, and alcohol consumption were extracted from medical records.
Composite scores were created for the physical and psychological HRQoL. The physical score was based on six items (fitness, independence in daily activities, ability to pursue leisure activities, tolerance of exertion, fatigue, difficulties with eating/drinking). The psychological score incorporated eight items (sleep, mood, anxiety, stress, loneliness, sense of control, social support, participation in social activities). Both were scaled 0–100 and categorized as low (0–33), moderate (34–67), or high (68–100).

**Results:**
The 21 patients analyzed were predominantly male (61.9%, n=13), with the same proportion aged above 65 years. Diabetes mellitus was highly prevalent, affecting 66.7% (n=14) of the cohort, and in 42.9% (n=9) the diagnosis was made only after surgery, highlighting the metabolic consequences of pancreatic resection.
With respect to subjective well-being, patients reported a mean reduction of 2.6 points on a 0–6 scale. Nearly half (47.6%, n=10) attributed these limitations directly to their tumor disease and treatment.
When HRQoL domains were analyzed separately, physical scores were high in 47.6% (n=10), moderate in 28.6% (n=6), and low in 23.8% (n=5). Psychological HRQoL outcomes appeared more favorable as 61.9% (n=13) reached the high range, 28.6% (n=6) the moderate range, and only 9.5% (n=2) reported low values.
Treatment-related side effects were mainly associated with adjuvant chemotherapy. Fatigue, gastrointestinal complaints, and neuropathy were most frequently reported, with an average severity of 3.5 on a 0–5 scale. In addition, postoperative weight loss was a common finding, observed in 85.7% (n=18) of all patients, with a mean reduction in body weight of 9 kg.
Exploratory analyses revealed no significant correlations between HRQoL outcomes and demographic or lifestyle-related factors. Age, sex, diabetes, smoking status, alcohol consumption, and time since surgery showed no measurable influence on either physical or psychological HRQoL (all $p > 0.05$).

**Conclusion:**
This study provides insights into postoperative HRQoL following operation in patients with PDAC. Overall, patients reported surprisingly favorable outcomes, particularly in psychological well-being. Despite weight loss and chemotherapy-related burden, many maintained satisfactory physical functioning and strong psychosocial resilience.
Two important implications emerge:

1. **Supportive care is critical.** While surgery itself caused relatively few long-term complaints, chemotherapy toxicity and postoperative weight loss were major issues. Tailored supportive strategies, including nutritional counseling, pancreatic enzyme replacement, symptom management, and psychological support, are therefore essential to optimize HRQoL.
2. **Resilience factors matter.** Demographic and lifestyle factors did not correlate with HRQoL, suggesting that other factors such as psychosocial adaptation and social support networks may play a more decisive role than traditional risk variables.

This study is limited by its small sample size, retrospective design, and single-center setting. Larger prospective studies are currently underway to validate these findings and to better define determinants of QoL in PDAC.

**Bibliography**

8. Sung H, Ferlay J, Siegel RL, et al. Global Cancer Statistics 2020: GLOBOCAN Estimates of Incidence and Mortality Worldwide for 36 Cancers in 185 Countries. *CA Cancer J Clin*. 2021;71(3):209-249. doi:10.3322/caac.21660
9. Rahib L, Smith BD, Aizenberg R, Rosenzweig AB, Fleshman JM, Matrisian LM. Projecting cancer incidence and deaths to 2030: the unexpected burden of thyroid, liver, and pancreas cancers in the United States. *Cancer Res*. 2014;74(11):2913-2921. doi:10.1158/0008-5472.CAN-14-0155
10. Seufferlein T, Mayerle J, Boeck S, et al. S3-Leitlinie Exokrines Pankreaskarzinom – Version 3.1. *Z Gastroenterol*. 2024;62(10):1724-1785. doi:10.1055/a-2338-3716
11. Park W, Chawla A, O'Reilly EM. Pancreatic Cancer: A Review. *JAMA*. 2021;326(9):851-862. doi:10.1001/jama.2021.13027
12. Schmidt-Hansen M, Berendse S, Hamilton W. Symptoms of Pancreatic Cancer in Primary Care: A Systematic Review. *Pancreas*. 2016;45(6):814. doi:10.1097/MPA.0000000000000527
13. Cipora E, Czerw A, Partyka O, et al. Quality of Life in Patients with Pancreatic Cancer—A Literature Review. *Int J Environ Res Public Health*. 2023;20(6):4895. doi:10.3390/ijerph20064895

DE GRUYTER BRILL

**Bedside assistance in robotic visceral surgery: performance, workload, satisfaction, outcome**
ID: E-DCK-105

**Viszeralchirurgie / Visceral Surgery**
**Robotische Chirurgie, Innovationen, KI, Virtual Reality (CA-Robin) / Robotic Surgery, Innovations, AI and Virtual Reality (CA-Robin)**

Nazarii Polishchuk[1], Mihailo Andric[1], Sara Al-Madhi[1], Roland S. Croner[1], Jessica Stockheim[1]

[1]Universitätsklinikum Magdeburg A. ö. R., Otto-von-Guericke-Universität Magdeburg, Deutschland, Klinik für Allgemein-, Viszeral-, Gefäß- und Transplantationschirurgie, Magdeburg, Deutschland

**Background:**
Robotic-assisted surgery is increasingly important in visceral surgery, offering better patient outcomes, enhanced precision and better working conditions. However, the safe and effective use of robotic surgical systems requires a structured pathway for young surgeons to gain proficiency in this technology.

The “Robotic Curriculum for Young Surgeons” (RoCS) was developed at the German University Hospital Magdeburg in 2020 as a multimodal training program for novice surgeons. Bedside assistance is intentionally assigned to surgical residents at the outset of their robotic training. It functions as transitional phase prior to console training.

The objective of this pilot study was to provide detailed insights into bedside assistance in robotic visceral surgery by analyzing performance, workload, trainees’ satisfaction and patient outcome.

**Material and methods:**
This study was conducted within the RoCS framework between 2020 and 2024. Eligible cases required complete postoperative documentation of the bedside assistant, while implausible or incomplete datasets were excluded. Data from adult patients were included for assessment. Data collection was performed prospectively, non-randomized and electronically using RedCap. Bedside assistance was defined by its specific procedural components, with evaluation including both trainee self-assessment and trainer assessment, pre- and postoperatively, generating multiple entries per operation.

1. **Performance:** Evaluated by the console surgeon using the Ottawa Surgical Competency Operating Room Evaluation (O-SCORE), a validated 5-point scale assessing procedural independence.
2. **Workload:** Assessed postoperatively using the NASA Task Load Index (NASA-TLX) on six subscales (0–20), combined into an overall 0–100 score; participants also rated whether the procedure was easier, as expected, or more difficult; assessment was completed postoperatively by trainer and trainee.
3. **Trainee satisfaction:** Measured via a non-validated 5-point Likert questionnaire on self-confidence and teaching quality.
4. **Patient outcomes:** Including intraoperative and postoperative complications (30- and 90-day), morbidity (Clavien-Dindo grade III), and mortality.

Data analysis was performed using IBM SPSS Statistics v29. Descriptive statistics are reported as means ± SD for continuous variables and frequencies (%) for categorical variables. Statistical significance was defined as $p < 0.05$.

DE GRUYTER BRILL

**Results:**
A total of 240 surgeries were included, encompassing procedures of the upper gastrointestinal tract (n = 125, 52.1%), colon (n = 37, 15.4%), liver (n = 27, 11.3%), pancreas (n = 27, 11.3%), rectum (n = 20, 8.3%) and other sites. In 48.3% of cases (116/240) the indication for surgery was oncological. Procedural components of bedside assistance comprised establishing a pneumoperitoneum, trocar placement, docking, and intraoperative assistance. Nineteen surgical trainees participated as bedside assistants, with a mean of 12.6 procedures per trainee.
Workload
The overall workload was NASA TLX 21.5 ± 21.1. Mental demand and physical demand were 5.1 ± 5.0 and 4.0 ± 4.2, respectively. Frustration was 3.8 ± 4.7. The perceived task difficulty was most frequently reported as “as expected” (n = 196, 81.7%).
Trainees’ satisfaction
In 74.6% of bedside assistances (179/240), trainees reported the highest confidence rating (5/5), indicating that they felt capable of independently performing the assigned procedural steps in subsequent procedures.
Patient outcomes
In 92.9% of procedures, no complications occurred. The most frequently documented intraoperative complication was bleeding (2.5%; 6/240). When bedside assistants performed intraoperative tasks such as establishing a pneumoperitoneum (80% of cases, 192/240) or trocar placement (33.3% of cases, 80/240), surgical complications remained infrequent (pneumoperitoneum-related complications: 5/192, 2.6%; trocar placement-related complications: 2/80, 2.5%).
Performance
O-SCORE ratings were available in 75.4% of cases (181/240). The bedside assistant was evaluated as able to perform the procedure safely and independently in 71.8% of cases (130/181), while 28.2% did not meet this criterion (51/181). Bedside assistance was considered to have no impact on the intraoperative course in 72.9% of cases (132/181), a positive impact in 26.5% (48/181), and a negative impact in 0.6% (1/181).

**Conclusion:**
This pilot study provides structured insights into bedside assistance in robotic visceral surgery using a multiperspective assessment and real-world clinical data. Bedside assistance proved a valuable training opportunity for surgical residents, offering a safe, low-workload and supportive environment and fostering technical competence and confidence. High trainee satisfaction and perceived task achievability support its formal integration into robotic surgery curricula as an initial step in developing basic robotic competence. Low complication rates indicate patient safety was maintained. O-SCORE ratings show that novices performed tasks without disrupting procedures, demonstrating that structured early experience fosters both technical skill and confidence. Limitations include the inability to attribute complications causally to bedside tasks, the absence of a laparoscopic comparison group, and the single-center design, which may limit generalizability.

Bedside assistance in robotic visceral surgery represents a safe and effective introductory training step, promoting skill acquisition and confidence in novice surgeons. Trainee performance and satisfaction indicate that early engagement does not compromise procedural integrity or patient safety. These results support its structured inclusion in robotic surgery curricula as a foundational component of surgical education.

DE GRUYTER BRILL

**Sigmoid Resections- Comparison of Robotic-Assisted vs. Laparoscopic Surgery for Diverticular Disease**
ID: E-DCK-108

**Viszeralchirurgie / Visceral Surgery**
**Robotische Chirurgie, Innovationen, KI, Virtual Reality (CA-Robin) / Robotic Surgery, Innovations, AI and Virtual Reality (CA-Robin)**

Nora Hagenmüller[1], Patryk Kambakamba[1], Sabine Schrödel[1], Christoph Anthoni[1], Maximilian Wenz[1], Stefan Farkas[1]

[1]St. Josefs Hospital, Allgemein- und Viszeralchirurgie, Wiesbaden, Deutschland

**Background:**
Robotic systems are increasingly used in colorectal surgery and offer potential technical advantages over laparoscopy. Their effect on patient-centered outcomes such as morbidity, recovery, length of stay and quality of life remains insufficiently defined, and routine use in benign disease is limited. Comparative analysis of robotic-assisted versus laparoscopic sigmoid resections is therefore of high clinical relevance.

**Objectives**
To compare outcomes of robotic-assisted versus laparoscopic sigmoid resections for Diverticular Disease.

**Material and methods:**
This investigator-initiated, retrospective, single-center cohort study analyzed consecutive patients treated at St. Josefs-Hospital Wiesbaden who underwent sigmoid resection for benign conditions between 2022 and June 2025. The Da Vinci Xi robotic system was implemented in our department since May 2022. Data were obtained retrospectively from the hospital information system, surgical and nursing documentation, and standardized digital follow-up tools. Collected parameters include demographic data (age, sex, BMI), ASA classification, comorbidities, intraoperative and postoperative complications (CCI), operative time, conversion rates, reoperation rates, and patient-centered outcomes (e.g., analgesic consumption, anastomotic insufficiency, stoma rate, time to first bowel movement).

**Results:**
A total of *235* patients met inclusion criteria, of whom *134* (57%) underwent robotic-assisted resection and *101* (43%) laparoscopic resection. Groups were balanced for age (median 61 und 64 years) and sex (46% male, 54% female; $p = 0{,}9$). Mean BMI was comparable (robotic-assisted 26 kg/m² vs. laparoscopic 27 kg/m²). ASA classification showed a higher proportion of low-risk patients in the robotic-assisted group (ASA I: 17% vs 6%), whereas more ASA III patients were observed in the laparoscopic cohort (26% vs. 13%). Still robotic population suffered from more advanced stages of diverticulitis as compared to the laparoscopic cohort, with a significantly higher proportion of Stage III Hansen& Stock disease. Nevertheless, required conversion rate was lower in the robotic group (n=1 (1%) vs. n=3 (3%)) to open surgery. Robotic-assisted sigmoid resections demonstrated significantly shorter operative times compared to laparoscopy (204 vs. 238 min, $p < 0.001$).

Patients undergoing robotic-assisted procedures had a shorter mean length of stay in hospital (5.7 vs. 8.5 days, $p < 0.001$) and required less opioid analgesia (WHO III: 37% vs. 45%). The first postoperative bowel movement occurred earlier after robotic surgery (1.5 vs. 2.1 postoperative days, $p < 0.001$).
Rates of anastomotic leakage were lower in the robotic-assisted group (5.2% vs. 6.9%), even if this did not reach statistical significance. The intra- and postoperative stoma rate was more favorable in the robotic-assisted group (1.5% to 5%, $p < 0.001$).
Overall complication rates were notably lower with robotic-assisted (17% vs. 43%), particularly for Clavien-Dindo grade II and III events. The comprehensive complication index (CCI) was lower in the robotic-assisted cohort (4.9 vs. 11.8).

**Conclusion:**
Robotic-assisted surgery appears to provide shorter operative times, favorable risk profile of stoma and re-operation-rate compared to conventional laparoscopy, particularly in relation to the length of hospital stay and postoperative analgesic use. Given these promising results, robotic resection for diverticular disease appears to be safe and, if confirmed by further prospective studies, holds the potential to become a future gold standard.

DE GRUYTER BRILL

**Robotic right hemicolectomy with CME: Surgical platform strategies for the new Hugo™ RAS System**
ID: E-DCK-147

**Viszeralchirurgie / Visceral Surgery**
**Robotische Chirurgie, Innovationen, KI, Virtual Reality (CA-Robin) / Robotic Surgery, Innovations, AI and Virtual Reality (CA-Robin)**

Stefanie Brunner[1], Lorenzo MacOne[1], Alexander Hapfelmeier[2], Dolores Krauss[1], Jennifer Eckhoff[1], Matthias Heldwein[1], Lars Schiffmann[1], Thomas Schmidt[1], Christiane J. Bruns[1], Georg Dieplinger[1], Hans Fuchs[1]

[1]Uniklinik Köln, Klinik und Poliklinik für Allgemein-, Viszeral-, Thorax- und Transplantationschirurgie, Köln, Deutschland, [2]Technical University of Munich, Institute of AI and Informatics in Medicine, 81675 Munich, Deutschland

**Background:**
Complete mesocolic excision (CME) during right hemicolectomy is a technically demanding surgical procedure that can benefit from the enhanced precision and dexterity offered by robotic assistance. The recently introduced Hugo™ Robotic-Assisted Surgery (RAS) System presents novel requirements for trocar placement and robotic cart orientation in order to optimize surgical workflow and instrument reach. This study aimed to evaluate the feasibility, reproducibility, and efficiency of the “open book setup” for robotic right hemicolectomy with CME using a 3D anatomical model.

**Material and methods:**
A standardized approach for trocar placement and robotic cart docking, defined by specific angles and tilt parameters, was implemented on a 3D anatomical model using the Hugo™ RAS System. The study involved two groups: experienced robotic surgeons with no prior exposure to the Hugo™RAS platform, and junior surgeons with limited robotic experience. For each participant, docking and undocking times were recorded. Additionally, we monitored time required at the console to achieve predefined critical views corresponding to key surgical steps in right hemicolectomy with CME. This setup allows assessment of both the technical feasibility of the proposed configuration and the learning curve for users with varying levels of robotic experience.

**Results:**
To date, six junior surgeons have completed the study protocol. Data from five experienced surgeons are planned to be collected in the coming months. Preliminary findings indicate that the mean docking time for junior surgeons decreased consistently across five trials. Docking times were reduced from 4:36 minutes in the initial trial to 1:38 minutes in the fifth trial.
Additionally, the surgical steps of right hemicolectomy were performed faster in each trial. T
o obtain the first view (mobilization of the cecum), mean times decreased substantially, from 2:08 minutes to 0:49 minutes. The preparation of the ileocolic vessels decreased from 1:06 minutes to 0:36 minutes. Configuration of the anastomosis decreased from 1:13 to 0:40 minutes in our experimental setting. Finally dedocking was improved from 01:38 minutes in the first trial to 1:09 minutes in the last trial.

**Conclusion:**

The findings of this ongoing study demonstrate that the proposed configuration for trocar placement and robotic cart docking using the Hugo™ RAS System is both feasible and reproducible for robotic right hemicolectomy with CME. By providing a structured and standardized approach to docking and intraoperative visualization, this study contributes practical guidance for adopting the new robotic platform and may support the learning process for novice surgeons. Data from experienced robotic surgeons will further clarify the system's applicability and efficiency across different levels of surgical expertise.

Abstracts – DCK 2026 – 143rd Congress of the German Society of Surgery – Leipzig, April 22–24 • DOI 10.1515/iss-2026-2002
Innov Surg Sci 2026; 11 (Special Suppl 1): s77–s269

DE GRUYTER BRILL

**Virtual Reality in Clinical Teaching and Diagnostics for Liver Surgery: Prospective Cohort Study**
ID: E-DCK-152

**Viszeralchirurgie / Visceral Surgery**
**Robotische Chirurgie, Innovationen, KI, Virtual Reality (CA-Robin) / Robotic Surgery, Innovations, AI and Virtual Reality (CA-Robin)**

Joshua Preibisch[1]

[1]Pius Hospital Oldenburg, Oldenburg, Deutschland

**Background:**
Learning and applying anatomy are essential but are studied and done through 2D tools and imaging teehniques. This study aims to vcrify the uscfulncss of an additional 3D tcchniquc and cnsure an improvcmcnt in thc visualisation of anatomical structurcs and pathological findings.
Thc study aims to cxaminc thc uscfulncss of virtual rcality (VR) lcchnology as an additional tool in mcdical diagnostics. Groups of studcnts, rcsidcnts, and spccialists in surgcry, radiology, and intcrnal mcdicinc cvaluatcd rnagnctic rcsonance imaging (MRI) by answering a multiplc-ehoiec qucstionnairc. Subscqucntly, a virtual 3D display was used for processing. The questionnaire focuscd on topographical conditions and the transfer of acadcrnic knowlcdgc into clinical application. Thc main objcctivc was to dctcrminc anatomical undcrstanding in a comparison bctwccn scctional irnagc (20) prcscntation and additional VR (3D) prescntation, mcasurcd through correctly answcred questions and proccssing time. The systcm usability scale (SUS) was intcgrated as another critcrion for VR usability.

**Material and methods:**
The cross-over study assessed 63 participants regarding their knowledge of livcr anatomy and pathologies based on an intcrindividual comparison. Group formation according to thc rcspectivc levcl of mcdical training was as follows: studcnts (n=35), rcsidents (n= 15), and spccialists (n=l 3). Par1ieipan1s answcred 25 multiplc-choicc questions first using scctional imaging (MRI) in a 2D environment (computer screen) and aftcrward with thc rcspcctivc scgmcnted 3D model visualizcd in a VR simulation. Thc main criteria for thcanalyscs wcre thc numbcrof correctly answcrcd qucstions and proccssing time. A customized SUS was uscd to analyze VR usability. Missing data analysis showcd that thcrc werc noaccountcd missing data.

**Results:**
Thc rate of eorrcet answers improvcd significantly with the additional use of VR ($F_{1,59}$=314.376; P<.001). Using MRI, a significanl differenec was obscrved betwcen slll dents and residenls (P=.04) and bctwecn students and specialists (P<.00 l). In the VR condition, no significant differences bctwecn groups werc found. In the MRI condition, significant differcnccs in proccssing time wcrc obscrvcd betwccn students and spccialists (P=.02) and betwccn rcsidcnts and spccialists (P=.04). No differenccs exisled betwcen studcnls and rcsidcnts. With VR, proccssing time dccrcascd significanlly in all groups ($F_{1,5g}$=280.700; P<.001). Significanl diffcrcnces betwcen studcnts and spccialists (P=.02) and between students and rcsidents (P=.004) remaincd. No notablcdiffcrcnccs bctwecn residents and spccialists (P=.72) werc found. Thc SUS showcd a subjcctively simplificd answerability of thcqucstions with additional usc of VR. Thc usefulncss and bcncfits f or an additional useof VR were statcd.

**Conclusion:**
Thc additional usc of VR suggcsts statistically significant improvemcnts across all groups. VR secms to cnablc studcnts and residcnts to participatc in diagnostics and crcatc trcatmcnt pi ans at an early stage. Transfcrred to clinical practicc, this may lead to improvcmcnt in diagnostics and intcrvcntions. Thc Jack of randomization and a potential learning effcct are thc main limitations tobe addrcssed in futurc studies.

DE GRUYTER BRILL

**From SOP to bedside: A Clinical Decision Support System (CDSS) for surgical oncology**
ID: E-DCK-169

**Viszeralchirurgie / Visceral Surgery**
**Robotische Chirurgie, Innovationen, KI, Virtual Reality (CA-Robin) / Robotic Surgery, Innovations, AI and Virtual Reality (CA-Robin)**

Thomas M. Pausch[1 2 3], Simon Meisetschläger[2], Georgios Polychronidis[1], Rosa Klotz[1 3], Matthias Ganzinger[2]

[1]Universitätsklinikum Heidelberg, Klinik für Allgemein-, Viszeral- und Transplantationschirurgie,, Heidelberg, Deutschland, [2]Universität Heidelberg, Institut für Medizinische Informatik (IMI), Heidelberg, Deutschland, [3]Universitätsklinikum Heidelberg, Studienzentrum der Deutschen Gesellschaft für Chirurgie (SDGC), Heidelberg, Deutschland

**Background:**
In everyday surgical oncology, translating static guidelines and hospital **Standard Operating Procedures (SOPs)** into reliable, point-of-care actions is hard. Surgeons face time pressure, complex staging and comorbidity patterns, and fragmented information across the Electronic Health Record (EHR). We set out to operationalize local SOPs so that they become **executable, explainable, and easy to use at the bedside**—without requiring programming skills from clinical staff and while adhering to the **European Union Medical Device Regulation (EU MDR)**. Our objective was to develop and implement a **Clinical Decision Support System (CDSS)** that (i) encodes hospital-specific SOPs as machine-readable, auditable artifacts using **Health Level Seven (HL7) Fast Healthcare Interoperability Resources (FHIR)**; (ii) provides an **interactive user interface (UI)** that displays only clinically relevant steps and data at a given moment; (iii) is engineered under an ISO 13485-aligned Quality Management System (QMS) toward MDR-compliant technical documentation; and (iv) enables **low-threshold maintenance** of SOP content by clinicians.

**Material and methods:**
We followed an iterative, surgeon-centred process:

1. **Clinical requirements and prioritization.** Four multidisciplinary workshops (hepatopancreatobiliary surgery, oncology, medical informatics) captured clinical user needs and prioritized 18 functional requirements. Emphasis was placed on bedside usability (e.g., minimal clicks, clear “met/not met” feedback) and on typical surgical variables (e.g., Tumor-Node-Metastasis [TNM] stage, bilirubin, performance status, coagulopathy, American Society of Anesthesiologists [ASA] class).
2. **Knowledge representation.** SOP content was transformed into **Event-Condition-Action (ECA)** rules and encoded as FHIR **PlanDefinition/ActivityDefinition** resources. To keep authoring accessible for non-programmers, we used **FHIRPath** for executable logic. A small custom FHIR extension captures datatype, operator, allowed range/set, and unit to drive UI behavior.
3. **Explainable, interactive UI.** The UI is generated dynamically from the FHIR artifacts: it shows only the data elements and checks actually needed for the current SOP step, provides immediate graded-match feedback (e.g., “TNM = T2N1M0 satisfies step X; bilirubin above threshold - review step Y”), and preserves the clinical wording from the SOP to reduce cognitive friction.

4. **System architecture and interoperability.** We implemented a containerized stack with a Java Spring backend, Angular frontend, PostgreSQL database, and an embedded **HAPI FHIR** server to enable standards-based exchange. Authentication via **Keycloak** is supported by the architecture for future deployments. Interfacing with the EHR is planned through FHIR APIs to import existing patient data and persist decisions.
5. **MDR-oriented engineering under QMS.** Within the institutional QMS (aligned to **ISO 13485**), we maintained explicit **design-control documentation**: user needs, design inputs/outputs, a traceability matrix linking SOP requirements to implementation and tests, verification plans, and preliminary **risk analysis** aligned with **MDR Annex I** (identification of hazards, foreseeable misuse, and mitigations). We also planned formative usability activities consistent with **IEC 62366-1** principles (usability engineering) prior to clinical piloting.

**Results:**

We delivered the **core functionality** required for pilot use:

14. **Executable SOPs** for surgical oncology encoded as HL7 FHIR artifacts with ECA logic and FHIRPath expressions.
15. **Dynamic, step-wise forms** generated from the SOP definition (no manual UI wiring), including datatype/operator/unit awareness, minimizing irrelevant fields and clicks.
16. **Immediate, legible feedback** at the bedside indicating which conditions are met or not met for a given step, making the rationale transparent to surgeons.
17. **Clinician-editable knowledge base:** surgeons can update thresholds, value sets, and step logic through a domain-specific editor **without programming**; changes are versioned and auditable at the artifact level.
18. **Initial use case:** an SOP for **pancreatic cancer** (e.g., preoperative staging and treatment pathway) was fully modeled and executed, demonstrating accurate handling of common variables such as TNM stage, bilirubin, cholestasis, and neoadjuvant therapy flags.

For the surgical team, the system functions like a **living SOP**: it surfaces exactly the data points needed for the current decision, shows why a recommendation applies (or not), and might reduce time lost to document hunting. Because the logic is deterministic and grounded in the hospital's own SOPs, it is **predictable and explainable,** closer to a surgical checklist that adapts to the case rather than a "black box." Importantly, clinicians can **maintain** SOP content themselves (e.g., thresholds, contraindications), supporting rapid updates as evidence or local practice evolves.

**Limitations and next steps**

This first implementation focuses on deterministic rule execution and interactive guidance; we have not yet deployed to production EHR environments nor completed summative usability testing. The upcoming milestones are: EHR integration via FHIR APIs; **role-based access control** and **provenance/audit**; expanded SOP libraries beyond pancreatic cancer; and formal usability and safety evaluations (task success, time-on-task, error rates) under real-world conditions.

**Conclusion:**

We demonstrate the feasibility of an **MDR-aligned, FHIR-native CDSS** that turns hospital SOPs into **executable, explainable bedside guidance** for surgical oncology. By combining FHIR knowledge artifacts, FHIRPath logic, and a dynamic UI that matches surgical workflows, the approach lowers adoption barriers for clinicians, keeps content ownership with the surgical service, and provides a clear pathway to compliant clinical piloting and EHR integration.

DE GRUYTER BRILL

**Model-Guided Surgery: Integrating AI and Robotics via Object-Oriented Hospital Information Systems**
ID: E-DCK-170

**Viszeralchirurgie / Visceral Surgery**
**Robotische Chirurgie, Innovationen, KI, Virtual Reality (CA-Robin) / Robotic Surgery, Innovations, AI and Virtual Reality (CA-Robin)**

Sidra Rashid[1], Lukas Bernhard[1], Sonja Stabenow[1], Emily Spicker[1], Charlotte Haid[1], Carl König[1], Hedi Louise Kramer[1], Sandro Pischinger[1], Daniel Schade[1], Katarina Sliepkova[1], Johannes Fottner[1], Dirk Wilhelm[1], Maximilian Berlet[1]

[1]Technische Universität München, TUM School of Medicine and Health, TUM-Universitätsklinikum rechts der Isar, Klinik und Poliklinik für Chirurgie, MITI Research Group, München, Deutschland, München, Deutschland

**Background:**
Hospital information systems (HIS) are the primary tools medical professionals use to access and manage patient data. These are particularly critical in surgical settings, where timely access to patient information is essential for the smooth execution of procedures across pre-operative, intra-operative, and post-operative stages. However, current HIS are largely designed as office applications rather than as comprehensive systems for patient management. Their lack of modularity limits integration with modern technologies such as artificial intelligence (AI) models and medical robotics (MR). As a result, information is often fragmented across separate applications, placing additional burden on medical professionals to gather data, interpret it, and make informed decisions. To address this gap, we propose an object-oriented HIS (oHIS) conceptualized as a pragmatic digital twin (PDT) of the entire surgical patient care in a hospital. We have developed a functional research framework based on this concept to enable further investigation and to bridge the gap between reality and digital models via oHIS.

**Material and methods:**
In collaboration with an interdisciplinary team of engineers and physicians, we conducted a requirements assessment on the surgical wards of the TUM University Hospital in Munich. Based on these findings, we designed the research framework named OMNI-SYS and developed it into a functional research platform capable of bridging the gap between a model management system and real-world agents. To evaluate the framework, we simulated a clinical use-case focused on surgical pre-operative patient tracking and management. Various tracking technologies such as RFID sensors, Bluetooth localisation, or WIFI triangulation can be used to update a patient’s location during the pre-surgical workflow within oHIS. This information is linked to the corresponding patient object, allowing medical professionals to monitor the progress of pre-operative tests. For the prototype demonstration, we implemented a simplified approach using QR code scanning to update patient locations and context information. Figure 1 displays the OMNI-SYS concept within the use case of a pre-surgical patient examination.

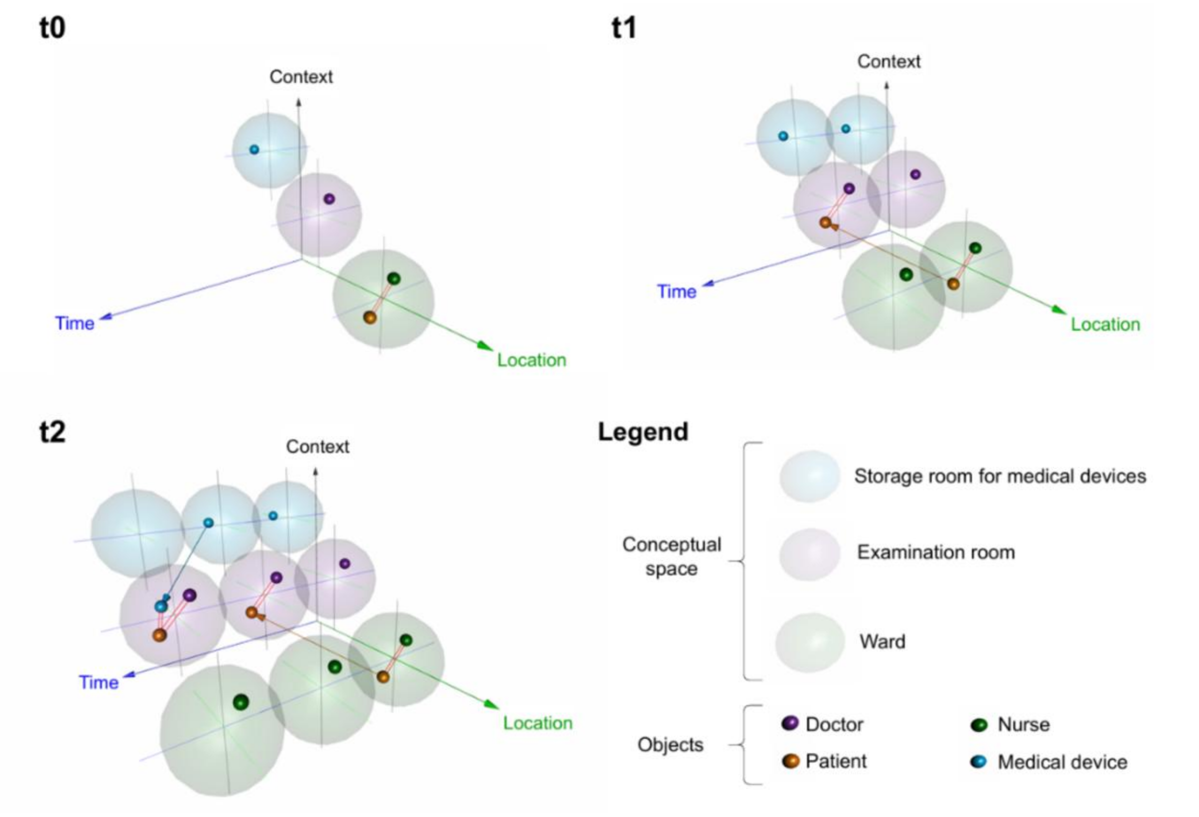

*Figure 1: Concept of object representation and object interaction in the 3-dimensional space of Time, Location, and Context / t0 (Timepoint 0): An ultrasound device (blue), a doctor (purple), and a patient (orange) are situated in the separated contextual and local spaces storage (transparent blue), examination room (transparent purple), and ward (transparent green). The patient is interacting (red lines) with a nurse (green). / t1: The patient moves to the examination room and interacts with the doctor. / t2: The ultrasound device moves from the storage to the examination room and interacts with the patient.*

**Results:**

Our analysis revealed that future-proof HIS is an under-researched topic. The integration of new technologies into clinical practice is not sufficiently prepared. Therefore, our approach could solve this shortcoming allowing for human agents, devices, models, and robots to interact in a PDT. Models can be integrated as quasi-natural objects and interact with representations of tangible objects in real-time. This approach enables surgeons and assistive medical professionals to manage their patients from one access point by integrating medical technologies and applications into oHIS. This approach enables even the future integration of new technologies that not yet exist and are still unimaginable today. Our oHIS research framework enabled a functional object representation in a simulated use case and opens the field of surgery-focused research on future-proof digital data management, AI integration, and device interconnection. Figure 2A displays the oHIS framework implementation simulating the use case of a pre-surgical patient examination. Additionally, figure 2B and 2C display an application that is built on top of the network representation of objects for tracking the pre-operative journey of a surgical patient.

DE GRUYTER BRILL

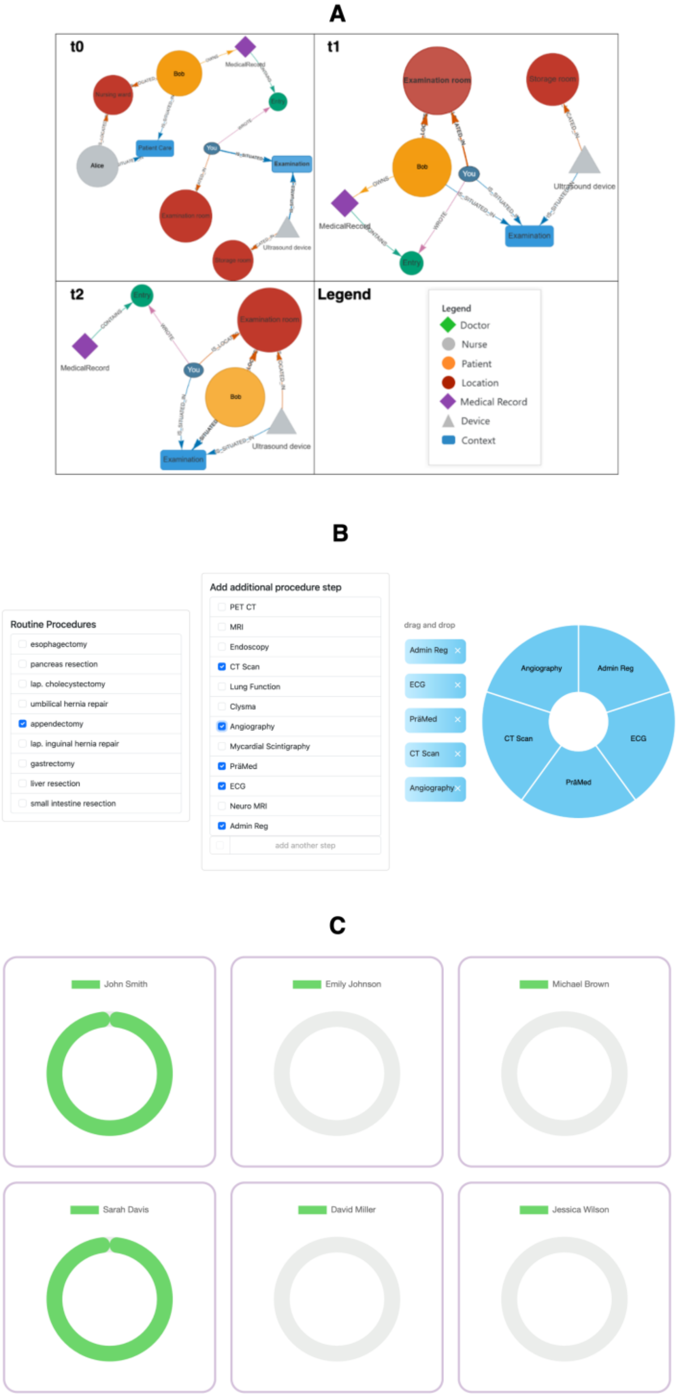

*Figure 2: Implementation of OMNI-SYS Framework in a pre-operative patient setting. (A):* Object representation and interaction in the 3D space of Time, Location, and Context / t0 (Timepoint 0): Main user (blue dot) in the examination room and examination context, patient (yellow dot) and nurse (grey dot) in the nursing ward and patient care context. Ultrasound device (grey triangle) in the storage but linked to examination context / t1: Patient moves to the examination room, interacts with the doctor. / t2: Ultrasound device moves to the examination room, interacts with the patient. *(B): drag and drop user interface that enables surgeons to define a patient's pre-operative path. (C): An overview of all patients' pre-operative path progress that tracks each patient's progress in completing test requirements before surgery.*

**Conclusion:**

oHIS could significantly facilitate the integration of future technologies like AI models and MR into surgical workflows. The OMNI-SYS framework could serve as a cornerstone for further research into this new approach. Studies on its clinical application and formalization are already planned in preparation for a possible future standard.

DE GRUYTER BRILL

### Evaluation of Ergonomics using the DEXTER® Robotic Surgery System – The ERGODEX Trial
ID: E-DCK-178

**Viszeralchirurgie / Visceral Surgery**
**Robotische Chirurgie, Innovationen, KI, Virtual Reality (CA-Robin) / Robotic Surgery, Innovations, AI and Virtual Reality (CA-Robin)**

Dolores Krauss[1], Matilda Krones[1], Lisa Abry[2], Fabiana Castronovo[3], Jennifer Eckhoff[1], Stefanie Brunner[1], Benjamin Babic[4], Michael David Mueller[3], Nico Seeger[2], Ryan Broderick[5], Christiane J. Bruns[1], Hans Fuchs[1]

[1]Uniklinik Köln, Klinik und Poliklinik für Allgemein-, Viszeral-, Thorax- und Transplantationschirurgie, Köln, Deutschland, [2]Kantonsspital Winterthur, Klinik für Viszeralchirurgie, Winterthur, Schweiz, [3]Inselspital Bern, Universitätsklinik für Frauenheilkunde, Bern, Schweiz, [4]Sana Klinikum Offenbach, Klinik für Allgemein- und Viszeralchirurgie, Offenbach, Deutschland, [5]University of California San Diego, Department of Surgery, Division of Minimally Invasive Surgery, San Diego, Vereinigte Staaten

**Background:**
The effects of complex robotic surgery on the well-being of surgeons remains widely unexplored. The DEXTER Robotic Surgery System (Distalmotion, Epalinges, Switzerland) has recently been FDA approved and introduced to clinical practice.  The surgeon console offers an open concept and can be operated in a sterile or nonsterile manner, standing, or sitting, allowing flexibility to switch between the robotic system and conventional laparoscopy at any time. Aim of this study was to evaluate surgical ergonomics during the use of the DEXTER Robotic system.

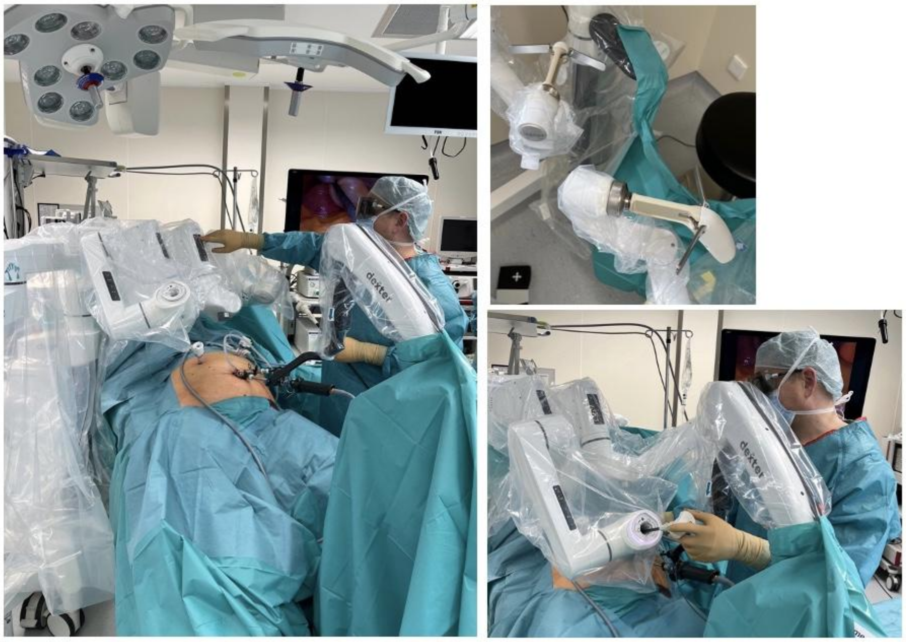

*Figure 1: DEXTER Robotic Surgery System using an open console concept as well as a laparoscopic – robotic modular hybrid approach.*

**Material and methods:**
A prospective, non-randomized multicentre trial evaluating surgical ergonomics during the routine use of the DEXTER robotic system in clinical practice was performed. Cleared study sites included the Department of General Surgery in Winterthur, Switzerland, the Department of Gynaecology in Bern, Switzerland and the Department of Surgery of the University of California San Diego, USA. A video camera was used to capture the surgeon at the console during surgery. Using video editing programs an image analyses was created showing the average posture of the surgeon and corresponding angles. Data was further compared to previous measurements(n=33 surgeries) of a closed-console robotic system using the same methods.

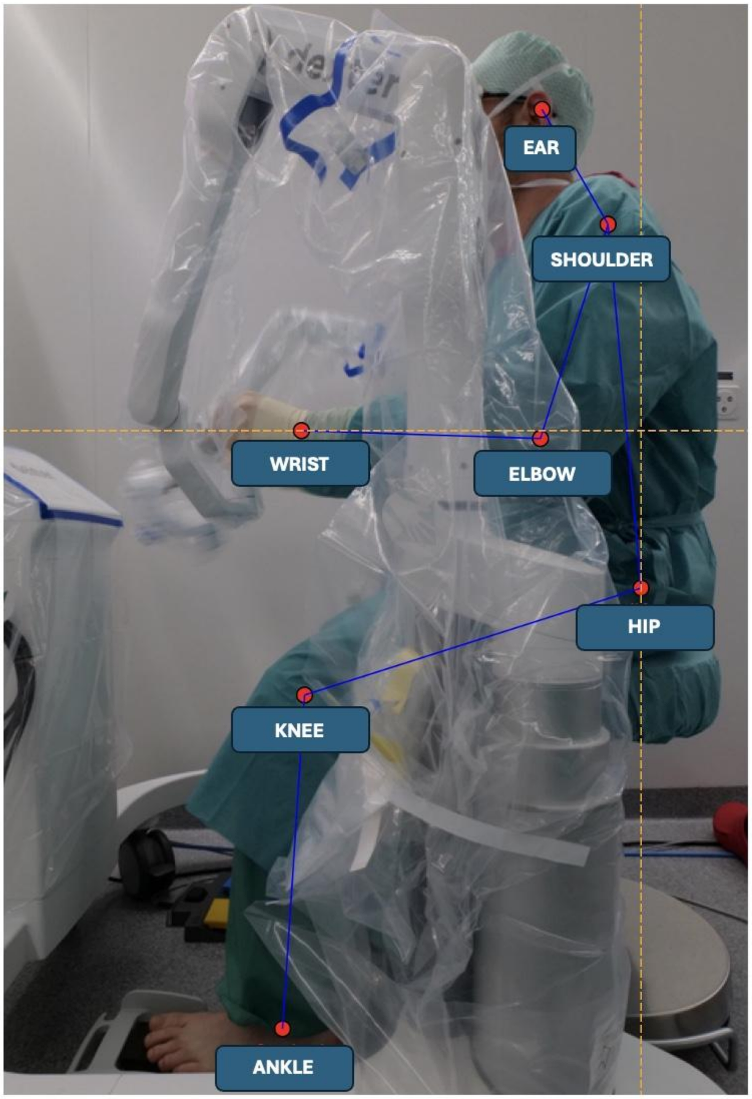

*Figure 3: Measurement of posture during robotic assisted surgery using the DEXTER Robotic Surgery System.*

**Results:**
A total of 30,848 photos from 27surgeries including 8cholecystectomies, 7 hernia repairs and 12hysterectomies from 3 different surgeons were evaluated. Results of the posture evaluation as well as comparison of data to current recommendations for a safe and healthy workspace are shown in table1. Table2 depicts the comparison of posture evaluation between an open (DEXTER) and closed console concept. Mean sound levels were 59.5dB with a mean minimum sound level of 53.9 and a mean maximum sound level of 84.5dB. Comparison to a robotic system with a closed console revealed a statistically quieter OR setting for the open DEXTER system (mean sound level 64.35dB vs. 59.5 dB; p value0.0001).

*Table 1: Evaluation of posture during a variety of surgeries using the DEXTER Robotic Surgery System.*

| DEXTER Robotic Surgery System | | | | | | | |
|---|---|---|---|---|---|---|---|
| | Knee | Hip | Shoulder | Neck | Elbow | Back | Forearm |
| Mean (°) | 105.28 | 100.73 | 31.08 | 22.18 | 120.50 | 4.48 | 5.38 |
| Median (°) | 106.24 | 101.20 | 28.48 | 22.73 | 118.01 | 4.32 | 5.13 |
| Range (°) | 92.66-115.48 | 93.53-106.3 | 16.34-50.59 | 11.54-30.08 | 101.9-143.29 | 1.89-9.3 | 2.08-14.82 |
| Recommendation (°) | ≥ 90 | 110-120 | N/A | 10-15 | ≥ 90 | 0-10 | N/A |
| Within Recommendation | Yes | No | N/A | No | Yes | Yes | N/A |

*Table 2: Comparison of posture between a robotic system with an open console concept and one with a closed console concept.*

| Posture (mean °) | Closed Console | Open Console | P value |
|---|---|---|---|
| Knee | 102.24 | 105.28 | 0.3828 |
| Hip | 99.38 | 100.73 | 0.2767 |
| Shoulder | 23.62 | 31.08 | 0.0002* |
| Neck | 33.37 | 22.18 | 0.0001* |
| Elbow | 105.05 | 120.50 | 0.0001* |
| Back | 5.37 | 4.48 | 0.2169 |
| Forearm | 5.56 | 5.38 | 0.8046 |

** a p value of <0.01 was considered statistically significant.*

**Conclusion:**
The application of ergonomic measurements during a real-life setting for robotic surgery helped reveal ergonomic advantages and challenges faced when using a new robotic technology. An open console may facilitate communication by providing a significantly quieter OR space as well as improve especially the high-risk neck posture seen in a closed console setting, making an open console favourable from an ergonomic standpoint. Identifying individual possibilities for improvement will in the future lay the foundation for a personalized training and education in surgical ergonomics leading to a healthier workspace for all surgeons and their future careers.

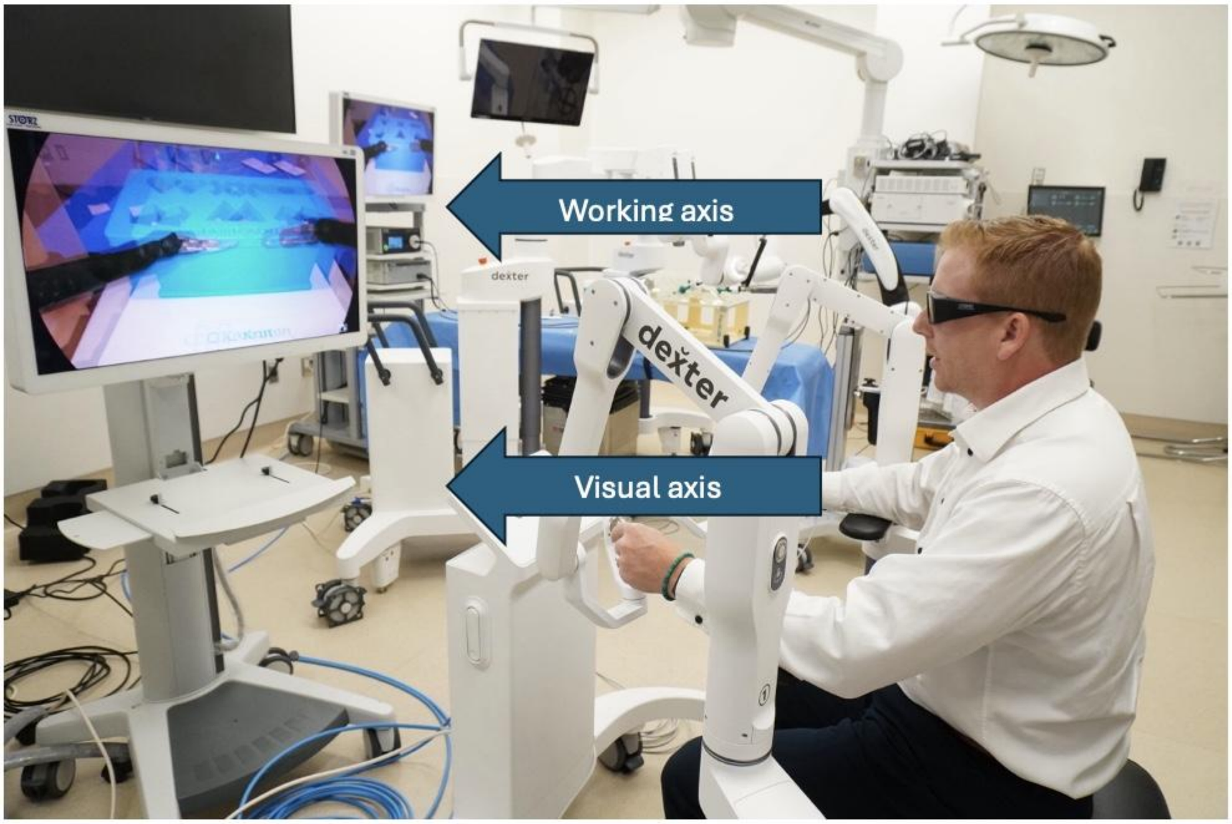

*Fig 2: The open console of the DEXTER Robotic Surgery System enhances communication and aligns the visual and working axis of the console surgeon.*

DE GRUYTER BRILL

**Outcomes of Robotic, Laparoscopic, and Open Colorectal Surgery: A Multicenter Cohort Study**
ID: E-DCK-356

**Viszeralchirurgie / Visceral Surgery**
**Robotische Chirurgie, Innovationen, KI, Virtual Reality (CA-Robin) / Robotic Surgery, Innovations, AI and Virtual Reality (CA-Robin)**

Lisa-Marie Schupp[1], Jasmin Zeindler[1], Florian Ponholzer[2], Dietmar Öfner[2], Daniel Frey[3], Stephan Vorburger[4], Sven Flemming[5], Nathaniel Melling[6], Michael Rousek[7], Tomas Poskus[8], Ovunc Bardakcioglu[9], Anas Taha[1], Katerina Neumann[10], Michael Honaker[11], Andres Heigl[1], Otto Kollmar[1], Beat Müller[12], Stephanie Taha-Mehlitz[12], Robert Rosenberg[1]

[1]Department of Visceral Surgery, Cantonal Hospital Basel-Land, Liestal, Schweiz, [2]Department of Visceral, Transplant and Thoracic Surgery, Center of Operative Medicine, Innsbruck, Österreich, [3]Department of Visceral Surgery, Cantonal Hospital Baden, Baden, Schweiz, [4]Department of Visceral Surgery, Teaching Hospital Emmental, Emmental, Schweiz, [5]Department of General-, Visceral-, Transplant-, Vascular- and Pediatric Surgery; University Hospital of Wuerzburg, Würzburg, Deutschland, [6]University Medical Centre Hamburg Eppendorf, Department of General, Visceral and Thoracic Surgery, University Medical Center Hamburg-Eppendorf, Hamburg, Deutschland, [7]Military University Hospital Prague, Department of Surgery, Prag, Czech Republic, [8]Department of Visceral Surgery, Faculty of Medicine, Vilnius University, Vilnius, Lithuania, [9]Division of Colon and Rectal Surgery, Kirk Kerkorian School of Medicine at UNLV, Las Vegas, Vereinigte Staaten, [10]Department of Surgery, Dalhousie University, Halifax, Nova Scotia, Kanada, [11]Department of Surgery, East Carolina University, Brody School of Medicine, Greenville, Vereinigte Staaten, [12]Clarunis, University Center for Gastrointestinal and Liver Diseases, St. Clara Hospital and University Hospital Basel, Basel, Schweiz

**Background:**
The surgical approach in colorectal resection has long been a focal point of clinical research due to its crucial and significant influence on postoperative outcomes and long-term prognosis. Over time, minimally invasive surgery is becoming more common due to its favorable clinical outcomes over open surgery. Despite the benefits of robotic surgery seen compared to laparoscopic surgery, heterogeneous multicenter research with large patient numbers comparing all three modalities across diverse indications is limited. This multicenter cohort study aims to compare the outcomes of patients undergoing robotic, laparoscopic, or open colorectal surgery.

**Material and methods:**
Therefore, we performed a retrospective cohort study of 7147 patients from 13 centers in Europe and North America who underwent colon anastomosis between January 2012 and December 2020. The primary outcomes of the study included operative time, hospital stay, comprehensive complication index (CCI), complications, and anastomotic leakage rates. Additionally, analyses for neoplasia and diverticulitis subgroups were performed.

**Results:**
Robotic surgery was associated with significantly longer operative time but shorter hospital stay and the lowest overall complication rates. Compared to open surgery, robotic surgery procedures had a 54% and

50% lower risk for complications and anastomotic leakage, respectively. Additionally, patients undergoing robotic surgery had lower CCI compared to open surgery but similar outcomes compared to laparoscopic procedures. Subgroup analysis of neoplasia and diverticulitis patients showed favorable outcomes for robotic surgery.

**Conclusion:**

Despite longer operative time, robotic surgery was associated with reduced morbidity and shorter hospitalization rates compared to both laparoscopic surgery and open surgery. This multicenter study showed the outcome of 13 centers performing colorectal surgery and provides a large-scale real world database across different indications. The reduction in hospital stays, complication rates and anastomosis leakage rates optimizes resource utilization and reduces the financial burden on the healthcare system, even though the cost of robotic surgery is currently one of the biggest challenges. Certainly, as a retrospective analysis, this study has its limitations, but it has shown that minimally invasive surgery, especially robotic surgery, is associated with significant benefits for patients. Further prospective and well-designed studies are needed to validate the results and explore the long-term and cost-effectiveness of robotic surgery compared to laparoscopic surgery and open surgery.

DE GRUYTER BRILL

**Preoperative Surgical Evaluation of Pancreatic Cancer Using VR – a Randomized Study**
ID: E-DCK-395

**Viszeralchirurgie / Visceral Surgery**
**Robotische Chirurgie, Innovationen, KI, Virtual Reality (CA-Robin) / Robotic Surgery, Innovations, AI and Virtual Reality (CA-Robin)**

Haluk Morgül[1], Karl Eisenträger[2], Kaya Saribeyoglu[2], Uli Fehrenbach[2], Matthäus Felsenstein[2], Lea Timmermann[2], Wenzel Schöning[2], Johann Pratschke[2], Andreas Pascher[1], Thomas Malinka[2], Benjamin Struecker[1], Igor Maximilian Sauer[2], Moritz Queisner[2]

[1]Uniklinik Münster, Münster, Deutschland, [2]Charité –Universitätsmedizin Berlin, Berlin, Deutschland

**Background:**
Virtual Reality (VR)-based technologies have the potential to enhance volumetric rendering of both anatomic and pathologic structures. While the positive impact of VR has been demonstrated in medical training and education, its implementation in routine clinical practice remains limited. In this study, we compared the diagnostic performance of VR with that of conventional 2D imaging in the evaluation of pancreatic cancer.

**Material and methods:**
12 cases with PDAC in different stages (resectable, border-line resectable, locally advanced) were identified using contrast enhanced bi-phasic CT scans. Participants were then randomized into 2 groups (group A: using a conventional 2D DICOM-viewer; group B: using Medicalholodeck® VR-software on Oculus 3 head mounted display hardware). The accuracy of the answers, timing, and inter-rater agreement were investigated.

**Results:**
5 surgeons were randomized into each group with similar professional experience (A: 15 years [6-21]; B: 18 years [10-32]). Evaluation time per case was slightly shorter in VR without significance (A: 327s; B: 313s). Group B showed the highest inter-rater agreement on assessments of the superior mesenteric artery (κ=0.67 vs. κ=0.54), whereas others showed the highest agreement in resectability (κ=0.61 vs. κ=0.13) and celiac trunk assessment (κ=0.64 vs. κ=0.19). Moreover, the conventional group showed better accuracy compared to the VR group (A: 0.85 vs. B: 0.79).

**Conclusion:**
Although using VR, surgeons were faster in surgical evaluation, the accuracy was limited. Furthermore, the overall inter-rater agreement was higher in conventional group.

DE GRUYTER BRILL

**Enhancing Laparoscopic Surgery via Audification of Surgical Tasks Using Vibroacoustic Sensing**
ID: E-DCK-87

**Viszeralchirurgie / Visceral Surgery**
**Robotische Chirurgie, Innovationen, KI, Virtual Reality (CA-Robin) / Robotic Surgery, Innovations, AI and Virtual Reality (CA-Robin)**

Nazila Esmaeili[1], Alfredo Illanes[2], Axel Boese[3], Michael Ghadimi[4], Jannis Hagenah[1 5]

[1]University Medical Center Göttingen, Department of General, Visceral and Pediatric Surgery, Center for Digital Surgery, Göttingen, Deutschland, [2]Otto-von-Guericke-University Magdeburg, Faculty of Computer Science and Research Campus STIMULATE, Magdeburg, Deutschland, [3]Otto-von-Guericke-University Magdeburg, INKA Innovation Laboratory for Image Guided Therapy, Magdeburg, Deutschland, [4]University Medical Center Göttingen, Department of General, Visceral and Pediatric Surgery, Göttingen, Deutschland, [5]Fraunhofer Research Institution for Individualized and Cell-based Medical Engineering (IMTE), Lübeck, Deutschland

**Background:**
Minimally invasive surgery (MIS), including laparoscopic surgery, has become the standard of care for many abdominal operations. The smaller incisions and indirect access provide major clinical benefits, such as reduced pain, shorter hospital stay, and faster recovery. However, MIS also limits direct sensory feedback for surgeons. In particular, tactile and auditory information, which are essential for surgical precision, are absent. As a result, surgeons rely almost entirely on visual feedback from the endoscopic video, which itself has limitations in field of view, resolution, and dynamics. The field of Surgical Data Science has sought to address these gaps by developing tools that improve perception during surgery. Imaging and navigation systems provide real-time instrument tracking, but their complexity and cost limit widespread clinical use. Endoscopic video analysis can add visual cues, but it remains computationally demanding and is constrained by the camera's perspective. Sensor-based haptic systems measure forces and pressures directly at the tool tip, but they require instrument redesign and are not yet clinically available. Vibroacoustic (VA) sensing offers a simple alternative. When instruments interact with tissue, natural mechanical vibrations are generated at the tool–tissue contact point and propagate along the instrument shaft. These vibrations can be collected at the instrument's proximal end as a VA signal, without modifying the instrument itself. The resulting signals contain rich information that can be translated into different forms of feedback in real time. One promising application is audification, which brings the tool-tissue interactions into human hearing range. This approach could restore the missing auditory sense in laparoscopy, offering an additional perception channel that complements vision without altering the surgical workflow. The objective of this study was therefore to evaluate the feasibility of using VA sensing technology for audification of tool-tissue interactions during standard laparoscopic surgical tasks, with the aim of enhancing surgeon auditory perception in MIS.

**Material and methods:**
We designed an experimental setup to simulate laparoscopic surgical tasks using a VA sensing system (SURAG Medical GmbH, Germany). The VA system consists of a sensing unit that attaches to the proximal end of standard laparoscopic instruments and a software interface running on an iPad. The sensing unit captures vibrations generated during tool-tissue interactions and transmits them wirelessly in real time via Bluetooth. The iPad application enables visualization of the VA signal, audification into the human hearing range, and storage of data for later analysis. With this system, up to two instruments can be monitored simultaneously without interfering with the surgical workflow.
For this study, ten representative laparoscopic surgical tasks were selected, including tissue cutting with scissors, palpation, suturing, cutting with instrument interaction, clipping with hemolok, tissue separation with dissection electrode, monopolar dissection, bipolar vessel sealing, bipolar cutting with scissors, and superficial devitalization. Standard laparoscopic instruments were used, including bipolar graspers, bipolar scissors, and a monopolar hook (Karl Storz SE & Co, Germany), while electrosurgical procedures were performed with clinical units (Erbe Elektromedizin GmbH, Germany). The experiments were carried out on ex-vivo porcine knuckle, including skin, fat, muscle, vessels, and bone, as well as porcine liver, chosen for their similarity to human tissue. For each task, the sensing unit was attached to the respective instrument, and VA signals were acquired and audified in real time via the iPad during task performance. Each task was repeated 10 times to ensure reproducibility.
For real-time audification of laparoscopic tasks, signal analysis was performed using advanced processing techniques in both time and time-frequency domains, with particular focus on features relevant for audification of laparoscopic tasks. A three-step signal processing pipeline was applied: first, a background noise model was estimated by analyzing spectral properties of the data; second, noise reduction was achieved using spectral gating; and finally, interaction signals were enhanced through a wavelet-based band-pass filter.

**Results:**
Across all ten laparoscopic surgical tasks, tool-tissue interactions generated clear VA signals that were distinguishable from the background, demonstrating the feasibility of using VA sensing for audification of surgical tasks. Moreover, the VA signal of each task showed unique dynamic patterns that created distinct sound signatures, allowing cutting, suturing, palpation, and electrosurgical actions to be differentiated by listening. This confirms that audification can complement currently muted laparoscopic videos by reintroducing sound as an additional sensory modality. Further VA data analysis within each surgical task showed that there are dynamical differences depending on the tissue type, suggesting that VA sensing in combination with Machine Learning could help distinguish between tissue layers during surgery. In electrosurgical applications, the VA signals reflected dynamical changes of tissue during energy delivery, highlighting a potential use for real-time monitoring of tissue effects. These findings demonstrate both the richness of the VA data and its potential for integration into surgical practice.

**Conclusion:**
This study demonstrates that VA sensing can restore the missing auditory dimension in MIS by translating natural tool-tissue vibrations into audification feedback. The approach is non-invasive, requires no modification of surgical instruments, and integrates seamlessly into the current clinical workflow. Surgeons can continue using their familiar instruments while gaining access to an additional sensory channel that enhances perception and decision-making. The VA sensing technology has the potential to improve surgical precision, safety, and outcomes in the operating room.

DE GRUYTER BRILL

### Study of the Connection Between Constipation, Diarrhoea, and Colorectal Cancer Using Real-World Data

ID: E-DCK-156

**Viszeralchirurgie / Visceral Surgery**
**Studien in der Viszeralchirurgie / Clinical Trials in Visceral Surgery**

Ernst Wolfgang Kolbe[1], Sarah Krieg[2], Christoph Roderburg[3], Sven Loosen[3], Karel Kostev[4], Andreas Krieg[1]

[1]Universitätsklinik für Allgemein-, Viszeral- und Thoraxchirurgie der Ruhr Universität Bochum, Medizin Campus OWL, Herford, Deutschland, [2]Universitätsklinik für Inklusive Medizin, Universitätsklinik Ostwestfalen-Lippe, Bielefeld, Deutschland, [3]Klinik für Gastroenterologie, Hepatologie und Infektiologie der Universitätsklinik Düsseldorf, Düsseldorf, Deutschland, [4]Epidemiologie, IQVIA, Frankfurt, Deutschland

**Background:**
This study aimed to investigate the temporal association between chronic constipation and diarrhea with the subsequent diagnosis of colorectal cancer (CRC), in order to assess whether these symptoms represent potential risk factors or early warning signs of malignancy.

**Material and methods:**
We conducted a retrospective case-control study using real-world data from the German Disease Analyzer database. The study included 10,941 CRC patients and 54,705 matched controls without cancer, matched 1:5 by propensity scores for age, sex, and relevant comorbidities. Documentation of constipation and diarrhea was evaluated in six time intervals up to five years before CRC diagnosis. Multivariable conditional logistic regression was used to calculate odds ratios (ORs) and 95% confidence intervals (CIs) for CRC risk.

**Results:**
Constipation was significantly more frequent in CRC patients within six months prior to CRC diagnosis (OR: 3.23; 95% CI: 2.85-3.66). A similar pattern was observed for diarrhea within six months prior to diagnosis, however, the association was stronger the more frequently diarrhea was documented (at least one diagnosis: OR: 3.37; 95% CI: 3.03-3.75; at least two diagnoses, OR: 4.18; 95% CI: 3.31-5.29; at least three diagnoses, OR: 6.82; 95% CI: 4.43-10.49). No associations were identified beyond one year prior.

**Conclusion:**
Constipation and diarrhea were associated with the subsequent diagnosis of CRC only in the immediate months before diagnosis, suggesting reverse causality rather than a causal relationship. These symptoms may serve as red flags rather than risk factors, emphasizing the need for timely diagnostic evaluation, especially in the presence of persistent changes in bowel habits.

DE GRUYTER BRILL

**Dynamics of body composition during neoadjuvant therapy in rectal cancer: a retrospective study**
ID: E-DCK-291

**Viszeralchirurgie / Visceral Surgery**
**Studien in der Viszeralchirurgie / Clinical Trials in Visceral Surgery**

Maximilian Trog[1], Armin Wiegering[1], Karoline Schmitz[2], Leon Schütze[2], Ekaterina Petrova[1]

[1]Goethe University Frankfurt/Main, Department of General, Visceral, Transplant and Thoracic Surgery, Frankfurt University Hospital, Frankfurt am Main, Deutschland, [2]University Hospital of Würzburg, Germany, Department of General, Visceral, Transplant, Vascular and Pediatric Surgery, Würzburg, Deutschland

**Background:**
Neoadjuvant therapy is an integral part of multimodal treatment of patients with advanced rectal cancer resulting in overall better oncologic outcomes.  On the other hand, neoadjuvant regimen can take up to several months, leading to a considerable time gap between diagnosis and surgery. In this study, we aimed at analyzing the changes in body composition during the time of neoadjuvant therapy and their impact on surgical outcome.

**Material and methods:**
Clinical data from patients who underwent multimodal treatment for rectal adenocarcinoma from 2013 to 2022 at the University Hospital Würzburg were collected retrospectively. Skeletal muscle (SM), intramuscular adipose tissue (IMAT), subcutaneous adipose tissue (SAT) and visceral adipose tissue (VAT) were quantified as area segmented from a single transversal CT scan slide at the level of the third lumbar vertebra prior and after neoadjuvant therapy scaled to patients' height. Changes in these body composition parameters were analyzed using the Wilcoxon Rank Test. A multivariable logistic regression with backwards elimination was applied to analyze the association of changes in the body composition parameters and postoperative complications according to the Clavien-Dindo classification, while adjusting for potential confounders.

**Results:**
Overall, 166 patients were included.  The average time between pre- and post neoadjuvant therapy CT scan was 117 days. There was statistically significant reduction in median SM ($p<0.001$) and SAT ($p=0.193$), while median IMAT increased ($p<0.001$). In the multivariable logistic regression, laparoscopic anterior rectal resection (OR 0.43, CI 0.19-0.95, $p=0.038$), total neoadjuvant therapy (OR 0.43, CI 0.21-0.88, $p=0.023$), positive change in SAT (OR 0.96 CI 0.92-0.99, $p=0.028$) and SM (OR 0.89 CI 0.79 -1.0, $p=0.046$) were associated with lower complication rates. Higher body mass index (OR 1.09, CI 1.01-1.18, $p= 0.040$) was associated with higher complication rates.

**Conclusion:**
In patients with advanced rectal cancer, there is a tendency towards reduction in skeletal muscle and subcutaneous adipose tissue and at the same time increase in intramuscular adipose tissue in the course of the neoadjuvant therapy. Loss of skeletal muscle and subcutaneous adipose tissue are associated with higher risk of postoperative complications.

DE GRUYTER BRILL

**TransValid-A study (ClinTrials.gov:NCT03034473) – Initial results and prognostic role of NAR for DFS**
ID: E-DCK-321

**Viszeralchirurgie / Visceral Surgery**
**Studien in der Viszeralchirurgie / Clinical Trials in Visceral Surgery**

Johannes Martens[1], Andreas Leha[2], Timotheus Ritter-Vanfloorop[3], Johanna Kreutzer[1], Andrea Hille[4], Julie Schanz[5 6], Torsten Liersch[1]

[1]University Medical Center of Göttingen, Department of General, Visceral and Pediatric Surgery, Göttingen, Deutschland, [2]University Medical Center of Göttingen, Scientific Core Facility for Medical Biometry and Statistical Bioinformatics, Göttingen, Deutschland, [3]Cellitinnen Hospital St, Antonius Köln (Bayenthal), Department of Trauma, Hand and Orhtopaedic Surgery, Köln, Deutschland, [4]University Medical Center of Göttingen, Department of Radiotherapy and Radiation Oncology, Göttingen, Deutschland, [5]University Medical Center of Göttingen, Department of Haematology and Medical Oncology, Göttingen, Deutschland, [6]University Medical Center of Göttingen, Department of Clinical Chemistry, Göttingen, Deutschland

**Background:**
The single-arm multicenter TransValid-A study (ClinTrials.gov: NCT03034473; funded by the German Research Foundation; DFG-GZ: LI 1727/3-1) examined the efficacy of 5-FU-based preoperative chemoradiotherapy (preopCRT), quality-controlled total mesorectal excision (qTME) and adjuvant chemotherapy (adCTx; FOLFOX or 5-FU) in patients with locally advanced rectal cancer (LARC, stages ≥ II; < 12 cm above the anal verge) combined with simultaneous collection/preservation of biomaterial. We aimed to find early predictors for disease-free survival (DFS) by analyzing various clinico-pathological parameters (e.g. neoadjuvant rectal score, NAR) using uni-/multivariable COX proportional hazards regression models.

**Material and methods:**
Between August 2011 and January 2015, 19 German study centers recruited 194 eligible patients (f: 36.1%; m: 63.9%; median age: 64 y; ECOG 0 to II in 63.9%, 28.4% and 7.7%, respectively) with LARC (stages II / ≥ III: 14.4% / 85.1%) localized in the lower (41.8%) or mid (57.7%) rectum. Standardized multimodal treatment (sMMT) was performed accompanied by standardized biomaterial sampling before, during, and after sMMT (including TME-surgery), as well as during > 5-y follow-up visits. The biomaterial sampling included tumor, mucosa and blood probes. For standardized data collection recording electronic case report forms (eCRF) and secuTrial® database were used. Four risk factors out of diverse parameters [e.g. age, sex, TNM-/UICC stage, BMI, CRM status (circumferential resection margin), study site and the NAR] with strongest association to DFS in uni-variable models (by significance from likelihood-ratio-tests) were selected for inclusion in multi-variable models. From clusters of strongly correlated factors (correlation ≥ 0.65) only the representative with the strongest association was included to avoid multi-collinearity problems. The significance level was set to alpha = 5% for all statistical tests. All analyses were performed with the statistic software R (version 4.4.0; R Core Team 2024).

**Results:**
180 LARC patients had started the preopCRT according to the protocol with RT + 5-FU (92.8%), with RT + 5-FU + OX (3.3%) and RT + capecitabine (CAP, 3.9%). According to the protocol, the 5-FU adherence reached 96.9 %; 92.8% of patients received 50.4 Gy of the RT. Preliminary data showed that CRT-associated toxicities according to CTC-AE G 3 and 4 occurred in 16.6% and 2%, respectively. Subsequently, 174 patients underwent qTME-surgery with optimal, moderate and poor quality in 84.5%, 7.5% and 1.7%, respectively. A 73.6% rate of ≤ II stages (vs. ≥ III in 26.4%) pointed out a remarkable LARC response to preopCRT. Complete remission (CR; ypT0) of the primary was achieved in 20.1% and the rate of stages 0 / I was 19.5% and 26.4%, respectively. 130 patients underwent adCTx (5-FU: 60.8%, mFOLFOX: 22.3%, other: 16.9%) The 3- and 5-year DFS rates were 72.6% and 68.4%, respectively (50 events), with a restricted mean DFS of 90.3 ± 5.8 months.
The multivariable Cox regression model for DFS using the risk factors ypUICC status, ratio of LN metastases / LN, age and CRM status, revealed only ≤ I ypUICC stages to be significantly predictive for an excellent DFS vs patients with stages ≥ II (HR: 2.755; 95%-CI [1.302; 5.8]; p=0.008). On univariate analysis the 3-y-/5-y-DFS for patients with low, intermediate and high NAR-score amounted 91.8% / 88.2%, 72.4% / 68.2%, 55.3% / 50.7% (p < 0.001), highlighting the correlation between low NAR-score and good prognosis. During sMMT, 4 study centers achieved the highest biomaterial ratio (planned/ submitted) of 60% to 70%, 5 centers reached 40% to 59%, and for 10 other sites this rate was 20% to 39%.

**Conclusion:**
Multivariable COX regression models for DFS revealed that patients with stage 0 / I after 5-FU-based sMMT had an outstanding prognosis compared to those with residual stages of ≥ II. Therefore, the postsurgical ypUICC stage is a valuable indicator for determining the most suitable CTx. The role of NAR and its additional impact as prognosticator remains under debate.

DE GRUYTER BRILL

**Roux-en-Y gastric bypass versus Fundoplication for GERD in class I/II obesity - REFUGO trial**
ID: E-DCK-482

**Viszeralchirurgie / Visceral Surgery**
**Studien in der Viszeralchirurgie / Clinical Trials in Visceral Surgery**

Johanna Betzler[1], Christine Stier[1], Christoph Reißfelder[1], Mirko Otto[1], Susanne Blank[1]

[1]University Medical Center Mannheim, Department of Surgery, Mannheim, Deutschland

**Background:**
Gastroesophageal reflux disease (GERD) is a common condition affecting approximately 14% of the European population and is even more prevalent among individuals with obesity (22.6%). In patients with obesity, management of GERD is particularly challenging due to increased intraabdominal pressure and obesity-related comorbidities. Laparoscopic fundoplication is the current standard surgical treatment for GERD, while Roux-en-Y gastric bypass (RYGB) has shown effective reflux control alongside significant and sustained weight loss. In patients with WHO class III obesity (BMI ≥ 40kg/m$^2$) RYGB is already favoured over fundoplication due to the above mentioned effects on weight and metabolic comorbidites. In WHO class I/II obesity (BMI 30-39.9 kg/m$^2$) the choice between fundoplication and RYGB involves considering several factors, including the severity of GERD symptoms, obesity-related comorbidities, patient’s preference, and the potential for weight loss and improvement in metabolic syndrome. So far, there is no recommendation in the German national guidelines concerning the choice of operative technique in patients with GERD and WHO class I/II obesity due to the lack of clinical trials. The REFUGO trial aims to adress this lack of evidence by directly comparing laparoscopic fundoplication and laparoscopic RYGB in patients with GERD and WHO class I/II obesity.

**Material and methods:**
REFUGO is a multicentre, prospective, randomized-controlled, parallel-group, non-inferiority trial designed to compare laparoscopic RYGB with laparoscopic fundoplication in patients with proven GERD and class I/II obesity (BMI 30–39.9 kg/m$^2$). Ninety-four patients will be randomized 1:1 across eight high-volume surgical centres in Germany. The primary endpoint is the change in GERD-specific quality of life (GERD-HRQL score) 12 months after surgery. Secondary endpoints include subjective symptom regression (reflux symptom index, RSI), weight and BMI development, bariatric quality of life (BQL), dysphagia, development of obesity-related comorbidities, pH monitoring results, endoscopic findings, need for anti-reflux medication, and re-intervention rates. Safety outcomes include operative time, length of hospital stay, and perioperative complications. Length of follow-up will be 12 months after surgery. Non-inferiority will be assessed using a linear mixed model including centre as a random intercept and group as fixed effect.

**Results:**
As the trial is in the planning phase, no results are available yet. Recruitment is expected to be completed within 24 months after initiation, with a total study duration of 51 months.

**Conclusion:**

The REFUGO trial will be the first multicentre randomized controlled study directly comparing RYGB and fundoplication for GERD in patients with class I/II obesity. Its results are expected to provide crucial evidence to guide surgical decision-making and inform future guideline recommendations, potentially improving both reflux control and metabolic outcomes in this growing patient population.

DE GRUYTER BRILL

**PERIoperative antibiotic prophylaxis in PANCreatic surgery (PeriPANC)**
ID: E-DCK-488

**Viszeralchirurgie / Visceral Surgery**
**Studien in der Viszeralchirurgie / Clinical Trials in Visceral Surgery**

Niels Siegel[1], Sophie Weber[1], Thomas Hank[1], Darius Halm[1], Benedict Kinny-Köster[1], Max Heckler[1], Jonathan Harnoss[2], Mohammed Al-Saeedi[1], Pascal Probst[3], Christoph Michalski[1], Martin Loos[1], Julian Harnoss[1]

[1]University Hospital Heidelberg, Study Center of the German Society of Surgery (SDGC), Dept. of General, Visceral and Transplantation Surgery, Heidelberg, Deutschland, [2]Giessen University Hospital, Department of General, Visceral, Thoracic and Transplantation Surgery, Giessen, Deutschland, [3]Cantonal Hospital Thurgau, Allgemein und Viszeralchirurgie, Münsterlingen und Frauenfeld, Schweiz

**Background:**
Pancreatic resections are associated with high postoperative morbidity, with surgical site infections (SSI) among the most frequent complications. Despite the established role of perioperative antibiotic prophylaxis in reducing infectious risks, optimal regimens remain undefined, and practice patterns vary considerably across institutions. A recent randomized controlled trial investigating perioperative prophylaxis in pancreatoduodenectomy demonstrated the superiority of piperacillin-tazobactam over cephalosporins in reducing postoperative infectious complications. At our institution, the previous standard regimen for pancreatic surgery consisted of ampicillin-sulbactam. In patients with biliary stenting, a combination of ceftriaxone and metronidazole was routinely administered. Given that the extent of resection is often determined intraoperatively and that microbial colonization patterns may vary, we aimed to evaluate the clinical impact of implementing this recommendation in routine practice across all pancreatic resections at our institution.

**Material and methods:**
This single-center, prospective observational before-after study was conducted at a high-volume center specializing in pancreatic surgery from January to July 2025. Adult patients undergoing elective pancreatic resection were included. The study comprised two cohorts: a retrospective standard group receiving ampicillin-sulbactam or ceftriaxone/metronidazole (in cases with biliary stenting), and a prospective intervention group treated with piperacillin-tazobactam following an institutional protocol change in April 2025 (German Clinical Trials Register, DRKS: DRKS00036546, ethical approval: S-087/2025). The primary endpoint was the rate of surgical site infections (SSI), defined as superficial, deep incisional, or organ/space infections occurring within 10 days (±2 days) postoperatively. Secondary endpoints included procedure-related complications and infection-associated outcomes. Group differences were analyzed using appropriate statistical tests.

**Results:**
A total of 267 adults undergoing elective pancreatic resection were included in final analysis. Of these, 130 patients (48.7%) received ampicillin-sulbactam or ceftriaxone/metronidazole (standard group), and 137 patients (51.3%) received piperacillin-tazobactam (intervention group) following a protocol change in April 2025. The overall surgical site infection (SSI) rate was 8.2%, with 22 cases (11 in the standard group [8.5%] vs 11 in the intervention group [8.0%]; absolute difference, 0.5% [95% CI, −6.2% to 7.0%]; p = .898). Most SSIs occurred independently of surgery-specific complications. Rates of superficial, deep incisional, and organ/space infections were low and comparable between groups (1.5% vs 0.7%; absolute difference, 0.8% [95% CI, −1.7% to 3.4%]; p = .531).

Key surgical complications - including pancreatic fistulas (POPF), pancreatojejunostomy (PJ) insufficiency, delayed gastric emptying (DGE), post-pancreatectomy hemorrhage (PPH), and postoperative acute pancreatitis (PPAP) - did not differ significantly between groups. For example, POPF occurred in 14 patients (10.8%) in the standard group and 23 patients (16.8%) in the intervention group (absolute difference, 6.0% [95% CI, 5.6% to 12.4%]; p = .155).
Subgroup analyses confirmed these findings: Among pancreatoduodenectomies with biliary stents (ceftriaxone/metronidazole vs piperacillin-tazobactam), SSI rates were identical (15.6% vs 15.6%; absolute difference, 0% [95% CI, −17.8% to 17.8%]; p = 1.000), as were rates of PJ insufficiency (2.9% vs 3.1%; absolute difference, 0.2% [95% CI, −8.2% to 8.6%]; p = .965). Among patients without biliary stents (ampicillin-sulbactam vs piperacillin-tazobactam), SSI rates remained similar (6.1% vs 4.8%; absolute difference, 1.4% [95% CI, −4.9% to 7.6%]; p = .669), with no differences in surgical complications. Thirty-day mortality did not differ between groups in any cohort or subgroup.

**Conclusion:**
This study provides a real-world evaluation of the updated German S3 guideline on perioperative prophylaxis in pancreatic surgery. In contrast to prior randomized data comparing piperacillin-tazobactam with cephalosporins, our findings did not demonstrate a significant advantage of piperacillin-tazobactam over ampicillin-sulbactam or ceftriaxone/metronidazole. From an antibiotic stewardship perspective, these findings support the continued use of institutionally tailored prophylaxis regimens and highlight the need for further prospective studies to refine guideline recommendations.

DE GRUYTER BRILL

**Unexpected turn: finding malakoplakia instead of an expected malignant tumor in transplanted patient**
ID: E-DCK-16

**Viszeralchirurgie / Visceral Surgery**
**Transplantationschirurgie (CAT) / Transplant Surgery (CAT)**

Marie-Helen Winter[1], Hans-Georg Keul[2], Christian Mönch[1]

[1]Westpfalz-Klinikum, Klinik für Allgemein-, Viszeral-, Kinder- und Transplantationschirurgie, Kaiserslautern, Deutschland, [2]Westpfalz-Klinikum, Institut für Pathologie, Kaiserslautern, Deutschland

**Background:**
Patients after transplantation are especially vulnerable due to immunosuppressive therapy. Not only infections but also cancer poses a considerable risk (1) .

**Material and methods:**
A 66 year old patient presented with abdominal discomfort and elevated CRP-level. The patient had received a decease donor kidney transplantation in the right iliac fossa seven month prior due to terminal dialysis-dependent kidney insufficiency caused by polycystic kidney disease. In addition to immunosuppressive therapy with tacrolimus the patient was on low-dose, long-term antibiotic therapy with trimethoprim-sulfamethoxazole.

**Results:**
Due to high infection parameters in a patient with known polycystic kidney disease a PET-CT scan with $^{14}$F-FDG was performed suspecting an inflammation of the polycystic kidneys. Instead a newly occurred retroperitoneal tumor with highly malignancy suspect glucose metabolism was diagnosed. After additional diagnostic with MR scan, the case was discussed in the interdisciplinary tumor board, primary resection was recommended due to the tumor's rapid growth, size and location. The surgical exploration revealed a tumor mass that extended from the back side of the right native kidney to the transplanted kidney infiltrating the ascending colon as well. The tumor appeared highly suspicious of a malignant tumor. The intraoperative instantaneous section showed inflammation containing macrophages without evidence of malignancy. The surgical intervention encompassed a right sided hemicolectomy as well as the nephrectomy of the right native kidney. After surgery CRP-level declined adequately and the patient recovered well so that discharge was possible on the 18th postoperative day. The postoperative histological results showed a histiocytic-granulomatous inflammation. Immunohistochemically the cell infiltrate was substantially positive for vimentin and CD68. Further investigation revealed a malakoplakia originating in the native kidney with infiltration of surrounding tissues.
By the time the definite histology was available the patient had been re-admitted to the hospital due to elevated CRP-level. In the microbiological testing E. coli, the most common infectious organism for malakoplakia (2), was present in the urin. An intravenous antibiotic regime with trimethoprim-sulfamethoxazole and levofloxacin was started. Initially CRP-level was decreasing adequately. After continuing the antibiotic regime orally CRP was rising again. The antibiotic regime was changed to cefotaxime in accordance with the antibiogram. As prolonged antibiotic therapy is necessary, the antibiotic regime was finally changed to cefpodoxime before discharge of the patient who appeared asymptomatic.

**Conclusion:**
Malakoplakia is a rare disease commonly occurring in immunocompromised adult patients. The prevalence is unknown but more than 700 cases have been described in the literature (3). Presently no treatment guideline exists. The variable clinical manifestations and non-specific radiological findings impede the diagnosis. In some cases the diagnosis can be secured through a biopsy (4). Treatment includes the reduction of the immunosuppressive therapy (2) as well as long-term antibiotics, i.e. trimethoprim-sulfamethoxazole, rifampicin and quinolone (5).
Even though malakoplakia is a rare disease, it is an important differential diagnosis in immunocompromised patients. Especially patients who have received a transplant within the last two years (2) and present with a tumor and high CRP. If there are no additional indications for malignancy, a biopsy should be considered, if feasible.

1. Al-Adra D, Al-Qaoud T, Fowler K, Wong G. De Novo Malignancies after Kidney Transplantation. Clin J Am Soc Nephrol. 2022;17(3):434-43.
2. Abbasi AB, Gamino G, Zambeli-Ljepović A, Whelan AM, Roll GR, Altshuler PJ. Malakoplakia among kidney transplant recipients: case series and literature review. Frontiers in Immunology. 2025;16:1605146.
3. Institut national de la santé et de la recherche médicale. Malakoplakia [Internet]. [updated 2011]. Available from: .
4. Boo Y, Yim H, Bang JB. Malakoplakia in the recipient's native kidney after kidney transplantation. Kidney Res Clin Pract. 2023;42(1):149-51.
5. Lee M, Ko HM, Rubino A, Lee H, Gill R, Lagana SM. Malakoplakia of the gastrointestinal tract: clinicopathologic analysis of 23 cases. Diagn Pathol. 2020;15(1):97.

Abstracts – DCK 2026 – 143rd Congress of the German Society of Surgery – Leipzig, April 22–24 • DOI 10.1515/iss-2026-2002
Innov Surg Sci 2026; 11 (Special Suppl 1): s77–s269

DE GRUYTER BRILL

**Hybrid 3D Printing Enhances Surgical Planning in Pediatric Living Donor Kidney Transplantation**
ID: E-DCK-193

**Viszeralchirurgie / Visceral Surgery**
**Transplantationschirurgie (CAT) / Transplant Surgery (CAT)**

Karim Bouhadjer[1], Stefan Lazarus[2], Gabriel Salg[1], Aghnia J. Putri[1], Christoph Michalski[1], Peri Husen[1]

[1]Universitätsklinikum Heidelberg, Heidelberg, Deutschland, [2]Technische Hochschule Mannheim, Mannheim, Deutschland

**Background:**
Living donor kidney transplantation in very small children is technically demanding due to size discrepancies between adult donor grafts and limited intraperitoneal space of the pediatric recipient. Vessel caliber mismatch and potentially difficult abdominal wall closure pose additional challenges. Pre-surgical planning using conventional two-dimensional imaging often fails to fully convey these complex anatomical relationships. By using patient-specific three-dimensional (3D) printing of adult donor grafts and the pediatric recipient's pelvis, exact preoperative understanding and interdisciplinary planning is possible.

**Material and methods:**
In three pediatric recipients of living-donor kidney transplantation, patient-specific hybrid 3D models were generated to support preoperative planning. Adult donor kidneys were reconstructed from thin-slice contrast-enhanced CT scans, while the pediatric recipient's entire abdominal cavity including vasculature was segmented from MRI. Image data were imported into 3D Slicer for segmentation and refined in Blender for surface optimization. Printable files were processed with Ultimaker Cura and fabricated using high-resolution polyjet technology at 1:1 scale. All models were reviewed and validated by transplant surgeons prior to use. The life-size reconstructions were employed in intra-team simulations to evaluate graft placement, vessel orientation, and feasibility of abdominal wall closure.

**Results:**
The 3D models enabled realistic preoperative simulation of graft placement, vascular anastomoses, and abdominal wall closure. In one case, an accessory lower-pole artery was clearly visualized and guided the vascular strategy. There was no need for mesh-assisted abdominal closure, which was confirmed intraoperatively, and primary closure was feasible. All three transplantations were completed successfully without major complications. At 3-month follow-up, all patients had functioning grafts.

**Conclusion:**
Hybrid 3D printing combining donor CT and recipient MRI provides detailed, patient-specific anatomical models that enhance surgical planning in complex pediatric kidney transplantation. This approach allows prediction of graft fit, vascular strategies, and abdominal closure, thereby reducing pre- and intraoperative uncertainty. Our proof-of-concept experience suggests that hybrid 3D printing represents a safe, feasible, and clinically valuable adjunct in pediatric transplantation surgery.

DE GRUYTER BRILL

**Impaired graft survival in kidney transplants from expanded criteria donors with HLA-DR mismatch**
ID: E-DCK-220

**Viszeralchirurgie / Visceral Surgery**
**Transplantationschirurgie (CAT) / Transplant Surgery (CAT)**

Uwe Scheuermann[1], Philipp Rhode[1], Claudia Lehmann[2], Jan Kowald[3], Sophie Seiffer[1], Fabian Haak[1], Daniel Seehofer[1], Sebastian Murad Rabe[1], Hans-Michael Tautenhahn[1]

[1]Department of Visceral, Transplantation, Vascular and Thoracic Surgery, University Hospital of Leipzig, Germany, Leipzig, Deutschland, [2]HLA Laboratory, Institute for Transfusion Medicine, University Hospital Hamburg-Eppendorf, Hamburg, Germany, Hamburg, Deutschland, [3]Medical Department III, Division of Nephrology, University of Leipzig Medical Center, Leipzig, Germany, Leipzig, Deutschland

**Background:**
Human leukocyte antigen (HLA)-DR mismatches are known to increase the risk of graft rejection following kidney transplantation (KT). Especially in KT using grafts of lower quality from expanded criteria donors (ECD), the immune response plays a critical role. Therefore, the aim of this study was to investigate the impact of HLA-DR mismatches on both short- and long-term outcomes in primary ECD KT.

**Material and methods:**
A total of 537 low-immunized patients who underwent primary KT at our institution between 1994 and 2024 were included in this retrospective analysis. KT recipients were stratified into four groups based on their HLA-DR mismatch status (absent [-] or present [+]) and donor type (standard criteria donor [SCD] or expanded criteria donor [ECD]): (1) SCD-DR-, (2) SCD-DR+, (3) ECD-DR- and (4) ECD-DR+. Clinicopathological characteristics, transplant outcomes, rates of rejection as well as patient and graft survival were evaluated across the groups.

**Results:**
Rejection-free graft survival was significantly decreased in recipients of ECD-DR+ KT, with an incidence rate of biopsy-proven rejections of 8.2 episodes per 100 patient-years (P = 0.005). Notably, the incidence of T cell–mediated rejection was significantly increased in HLA-DR+ ECD KTs. In comparison with ECD-DR- KTs, ECD-DR+ KTs were associated with a significantly higher rate of graft loss (mean death-censored graft survival: 15.1 ± 1.3 years versus 10.7 ± 0.8 years; P = 0.034). After adjusting for relevant donor, recipient and transplant cofactors using multivariate Cox regression analysis, HLA-DR mismatch remained an independent predictor of graft rejection (adjusted hazard ratio [aHR] 2.631, 95%CI 1.578–4.389; P <0.001) and graft survival (aHR 3.528; 95%CI: 2.008–6.197; P <0.001) in ECD kidney grafts.

**Conclusion:**
HLA-DR mismatches are associated with significantly reduced graft survival in ECD kidney transplantation. These results advocate for the consideration of HLA-DR compatibility in allocation protocols in this donor category, to improve long-term outcomes.

DE GRUYTER BRILL

**Waiting List Mortality in Liver Transplantation – A 10-year Single-Center Analysis**
ID: E-DCK-416

**Viszeralchirurgie / Visceral Surgery**
**Transplantationschirurgie (CAT) / Transplant Surgery (CAT)**

Vanessa Muth[1], Helena Linge[1], Pia Hartwig[1], Moritz Schmelzle[1], Markus Quante[1], Philipp Felgendreff[1], Cornelius van Beekum[1]

[1]Hannover Medical School, Department of General, Visceral and Transplant Surgery, Hannover, Deutschland

**Background:**
Liver transplantation (LT) remains the only curative therapy for end-stage liver disease, yet organ scarcity continues to limit access. In 2024, 1,529 new candidates were registered for LT in Germany, but only 890 transplantations were performed, resulting in 288 deaths on the waiting list (21%) (DSO Annual Report 2024). Previous studies have shown that beyond disease severity (MELD/MELD-Na), factors such as donor–recipient size mismatch, blood group O, hyponatremia, and frailty strongly influence waitlist mortality. However, the extent to which center-specific acceptance strategies contribute to patient outcomes remains unclear. We therefore conducted a 10-year single-center analysis focusing on non-transplanted candidates, organ allocation patterns, and rejection behavior. The aim was to determine whether organ allocation and matching decisions might indirectly influence waitlist mortality.

**Material and methods:**
All patients listed for primary LT at Hannover Medical School between 2014 and 2024 were retrospectively analyzed. Patients who underwent transplantation were excluded. 213 transplant candidates died while waiting. After excluding pediatric cases (n = 15), patients being permanently listed NT (n=15), relistings (n = 84), and incomplete data (n = 6), 93 deceased adult candidates remained. Demographic, clinical, and biochemical variables were analyzed, including primary liver disease, as well as duration and status on the waiting list. Organ offers were assessed for frequency, distribution per patient, and reason for decline. We requested data from Eurotransplant to evaluate organ utilization and to determine whether organs declined at our center were subsequently transplanted elsewhere.

**Results:**
Among 93 deceased candidates, 27 (29%) had alcohol-related cirrhosis, 17 (18%) cryptogenic, 15 (16%) viral HBV/HCV, 10 (11%) PSC/PBC, 5 (5%) MASLD, 3 autoimmune hepatitis, 2 HCC, and 10 had other etiologies.
Patients with PSC/PBC had the shortest waiting period before death (median total 66 days; median listed as T 35.8 days), while HCC patients remained listed longest (median total 1030 days; median listed as T 82 days).
Only 35 of 93 (37.6%) patients received at least one organ offer (total 72 offers). 58 patients (62.4%) received none. 16 patients had one offer, 8 had two, 5 had 3–4, and 1 had 5 offers.
Of all organs declined at our center, 40 (55.6%) were transplanted elsewhere; 14 were declined due to recipient instability or donor-recipient size mismatch. One-third of candidates who received an offer had none of the offered organs transplanted elsewhere.

**Conclusion:**
The majority of patients who died on the waiting list never received an organ offer, highlighting the persistent imbalance between organ availability and recipient need. More than half of the organs declined at our center were successfully transplanted elsewhere. These organ offers, however, only affected 25% of deceased waiting list patients at our center. Although it must be noted, that information on the viability of these organs was not assessed in this study.
With regard to the MELD score being replaced by the Re-MELD-Na score it seems import to reevaluate mortality on the waiting list over the next 10 years.
In line with recent studies emphasizing frailty, sodium levels, and size-mismatch as independent predictors of waitlist mortality, our findings underline the multifactorial nature of pre-transplant patient loss. Future strategies should therefore combine optimized matching algorithms with dynamic organ assessment tools—such as machine perfusion and donor-quality scoring—to expand the effective donor pool and reduce waitlist mortality in Germany.

DE GRUYTER BRILL

### Prognostic Value of AI-Driven CT-Based Body Composition Analysis in Liver Transplantation Candidates

ID: E-DCK-440

**Viszeralchirurgie / Visceral Surgery**
**Transplantationschirurgie (CAT) / Transplant Surgery (CAT)**

Mehmet Esat Belviranli[1], Adnan Jana[1], Sophia Schmitz[1], Arzu Oezcelik[1], Ulf Neumann[1], Eugen Malamutmann[1]

[1]Universitätsklinikum Essen, Essen, Deutschland

**Background:**
The Model for End-Stage Liver Disease (MELD) score remains the foundation of risk assessment for liver transplantation, but it does not reflect body composition or functional reserve, which are increasingly recognized as significant prognostic factors.
Manual assessment of body composition based on computed tomography (CT)–is time consuming and examiner dependent. Artificial intelligence (AI)–based automated CT segmentation enables objective measurement of skeletal muscle, visceral adipose tissue (VAT), and subcutaneous adipose tissue (SAT). Leveraging this approach, we aimed to delineate sex- and MELD-specific prognostic patterns beyond established models.

**Material and methods:**
We retrospectively evaluated 294 patients listed for liver transplantation at our center. Abdominal CT scans were processed with an automated segmentation pipeline to derive indices of skeletal muscle, VAT, and SAT in native, normalized, and ratio-based forms, including the abdominal visceral adipose tissue index (AVATI), its body surface area–adjusted variant (AVATI_BSA), the abdominal muscle volume index (AMVI), and intramuscular adipose content (IMAC). Prognostic performance was assessed using Cox regression (hazard ratios, HR), receiver-operating characteristic area - under the curve (ROC-AUC), and Kaplan–Meier survival analyses. Subgroup analyses stratified by sex and MELD category (<20 vs ≥20) explored heterogeneity of effects.

**Results:**
AI-based CT body composition metrics produced strong and independent prognostic signals. In the overall cohort, several indices were associated with mortality, with HR typically in the 2.0–3.6 range (all p<0.05). VAT and AVATI indices ranked among the strongest predictors. Sex-stratified analyses revealed distinct patterns: in men, intramuscular fat indices and the ankle–brachial index (ABI; HR ≈2.1–2.3) were most predictive, whereas in women, IMAC (HR 3.7, p=0.009) and periaortic adipose tissue (PAT, HR 3.0, p=0.031) predominated. MELD-stratified analyses highlighted stage-specific drivers: in patients with MELD <20, visceral adiposity measures (VAT, AVATI) were the principal determinants (HR up to ~3.6), while in MELD ≥20, muscle-related markers dominated, with AMVI_corrected reaching HR 6.4 and muscle volume HR 5.2. Together, these findings indicate that early-stage risk is predominantly metabolic/adiposity-driven, whereas advanced-stage risk reflects sarcopenia.

**Conclusion:**
This study shows that body composition is not a uniform risk factor but interacts with sex and disease stage. Automated CT analysis complements MELD by uncovering sex-specific (e.g., IMAC and PAT in women; intramuscular fat and ABI in men) and stage-specific prognostic signatures (visceral adiposity at MELD <20 versus sarcopenia at MELD ≥20). These findings support a dynamic, individualized framework for prognostic assessment. Integrating body-composition metrics into clinical practice may refine MELD-based risk stratification, guide sex- and stage-tailored monitoring, and inform prehabilitation and nutritional strategies in liver transplant candidates.

DE GRUYTER BRILL

### Impact of Temporary Abdominal Closure on Mortality After High-MELD Liver Transplantation
ID: E-DCK-506

**Viszeralchirurgie / Visceral Surgery**
**Transplantationschirurgie (CAT) / Transplant Surgery (CAT)**

Simon Störzer[1], Felix Gronau[1], Cornelius van Beekum[1], Philipp Felgendreff[1], Nicolas Richter[1], Moritz Schmelzle[1], Markus Quante[1]

[1]Medizinische Hochschule Hannover, Klinik für Allgemein-, Viszeral- und Transplantationschirurgie, Hannover, Deutschland

**Background:**
Liver transplantation in patients with a labMELD score >35 represents a particular clinical challenge, as this population typically presents with advanced organ failure and significant systemic instability. Consequently, these patients enter transplantation with a substantially higher risk of perioperative morbidity and mortality. Despite advances in surgical techniques and intensive care management, early postoperative outcomes in this vulnerable cohort remain critical. In addition to mortality, biliary complications – particularly ischemic-type biliary lesions (ITBL) – play a major role, as they can significantly impair graft function and long-term survival. The temporary abdominal closure (tBDV) is often employed in complex intraoperative situations associated with hemodynamic instability. However, its impact on postoperative complications and patient survival after liver transplantation remains incompletely understood.

**Material and methods:**
A retrospective analysis was conducted including 142 patients with a labMELD score >35 who underwent liver transplantation at Hannover Medical School between 2007 and 2017. Of these, 60 patients (42.3%) underwent temporary abdominal closure (tBDV), while 82 patients (57.7%) received primary abdominal closure. Postoperative 90-day mortality, intensive care unit (ICU) length of stay, and the incidence of ischemic-type biliary lesions (ITBL) were analyzed. Statistical analyses were performed using Fisher's Exact Test and logistic regression. Group comparisons were conducted using mean values and t-tests. A p-value <0.05 was considered statistically significant.

**Results:**
The 90-day mortality rate was 21.7% in the tBDV group compared to 9.8% in the non-tBDV group ($p \approx 0.10$). Although this difference did not reach statistical significance at $\alpha = 0.05$, it indicates a trend toward higher early mortality among patients requiring temporary abdominal closure. The mean ICU length of stay was 30.56 days in the tBDV group and 29.03 days in the non-tBDV group ($p = 0.79$), showing no significant difference.
With regard to biliary complications, ITBL occurred in 13 of 60 patients with tBDV (21.7%) and in 14 of 82 patients without tBDV (17.1%) ($p = 0.52$; OR 1.34; 95% CI 0.58–3.12). Logistic regression analysis did not identify tBDV as an independent risk factor for the development of ITBL.

Abstracts – DCK 2026 – 143rd Congress of the German Society of Surgery – Leipzig, April 22–24 • DOI 10.1515/iss-2026-2002
Innov Surg Sci 2026; 11 (Special Suppl 1): s77–s269

**Conclusion:**
Patients with a labMELD score >35 represent a highly vulnerable cohort entering transplantation with considerable physiological compromise and an inherently higher risk of adverse outcomes. In this study, overall perioperative mortality was elevated in this group, although temporary abdominal closure (tBDV) was not identified as an independent risk factor. Similarly, no significant differences were observed regarding ICU length of stay or the incidence of ischemic-type biliary lesions between groups.
The use of tBDV primarily reflects a complex intra- and perioperative situation characterized by hemodynamic instability and severe comorbidities. It should therefore be interpreted as a marker of disease severity rather than a direct cause of adverse outcomes. These findings highlight the importance of considering preoperative risk profiles when evaluating postoperative complications and survival in patients with very high MELD scores.

DE GRUYTER BRILL

### Surgical Outcomes of Ureteroureterostomy versus Ureterocystostomy in ADPKD Kidney Transplantation

ID: E-DCK-517

**Viszeralchirurgie / Visceral Surgery**
**Transplantationschirurgie (CAT) / Transplant Surgery (CAT)**

Paula Medio Torrubiano[1], Ivan Capobianco[1], Jens Rolinger[1], Arnold Radtke[1], André Mihaljevic[1], Jens Strohäker[1], Silvio Nadalin[1]

[1]Department of General, Visceral and Transplant Surgery, Tübingen University Hospital, Germany, Tübingen, Deutschland

**Background:**
In kidney transplantation (KTx) for autosomal dominant polycystic kidney disease (ADPKD), nephrectomy is sometimes required due to limited space, symptomatic kidneys, or specific listing criteria. When nephrectomy is performed, ureteral anastomosis can be achieved via ureteroureterostomy (UU) or ureterocystostomy (UC). The choice of technique is influenced by nephrectomy timing, patient anatomy, and surgeon preference. Comparative data on the impact of these techniques on urological complications and graft survival are limited. This study aims to compare UU versus UC in ADPKD KTx with respect to surgical outcomes and graft performance to determine whether UU reduces postoperative urological complications.

**Material and methods:**
This retrospective, single-center cohort study encompassed all consecutive patients with ADPKD who underwent KTx at our center between January 2011 and September 2025. Patients who required upfront or simultaneous nephrectomy were included and stratified according to ureteral anastomosis technique (UU or UC) and nephrectomy timing. Demographic and clinical variables, including age, sex, BMI, and donor type, were collected. Outcomes of interest included urological complications, recurrent UTIs, graft function, graft survival, and patient survival.

**Results:**
Of 79 patients, 46 (52.2%) underwent nephrectomy. To reduce potential confounders, only cases with upfront (n = 10/46; 21.7%) or simultaneous nephrectomy (n = 28/46; 60.9%) were considered and cases with postoperative nephrectomy (n = 7/46; 15.2%) or ileal conduit (n = 1; 2.2%) were excluded. UU was performed in 24 cases (61.5%), and UC in 14 cases (35.9%). Mean age and BMI were comparable between these groups (UU: 57.4 ± 9.1 kg/m²; UC: 57.5 ± 8.7 kg/m²). Male predominance was observed in the UU group (62.5%) compared with UC (50.0%). No differences were found in donor type or baseline demographics. UU was more frequently used in simultaneous nephrectomy (95.8% vs 35.7%), while UC predominated in pretransplant cases (64.3% vs 4.2%; $p<0.01$). No significant differences were found between UU and UC regarding recurrent UTIs ($\chi^2=1.49$, $p=0.69$), last serum creatinine (ANOVA=2.24, $p=0.10$), or last eGFR (ANOVA=1.69, $p=0.19$). Urological complications were infrequent (UC_sim=1/5; UU_sim=2/23; $\chi^2=0.68$, $p=0.88$). Reported events included vesicoureteral reflux, urinary obstruction, and anastomotic leakage, with no clear pattern by technique or nephrectomy timing. Patient and graft survival remained excellent across all groups. In simultaneous nephrectomies, a current trend toward the preferred use of UU was observed (ANOVA=6.98, $p$<0.01), indicating an emerging institutional tendency.

**Conclusion:**
Despite the small sample size, potential confounders, UU and UC provide comparable safety, graft function, and survival outcomes in ADPKD KTx. However, UU may facilitate simultaneous nephrectomy without increased urological morbidity, which reflects an evolving institutional preference, Longer and balanced follow-up is needed to confirm any potential superiority between techniques.

DE GRUYTER BRILL

**ENTPD3/CD39 expression on T cells in gastric signet ring cell carcinoma: novel immunological targets**
ID: E-DCK-305

**Viszeralchirurgie / Visceral Surgery**
**Viszeralonkologie, personalisierte Therapie (ACO) / Visceral Oncology and Personalised Treatment (ACO)**

Resa Puffert[1], Vincent Wolf[1], Felix Gronau[1], Aina Lask[2], Franziska Köhler[1], Moritz Schmelzle[1], Linda Feldbrügge[1]

[1]Hannover Medical School, Department of General, Visceral, and Transplant Surgery, Hannover, Deutschland, [2]Charité Berlin, Department of General, Visceral, and Transplant Surgery, Berlin, Deutschland

**Background:**
Gastric adenocarcinoma (GAC) and adenocarcinoma of the gastroesophageal junction (GEJ) represent common worldwide malignancies with poor prognosis despite advancing treatment options. Signet ring cell carcinomas (SRC) represent a special subtype of GAC carcinomas, characterized by diffuse growth, poor prognosis and limited response to chemotherapy. Current guidelines for non-metastatic but locally advanced GAC/GEJ carcinomas recommend surgery combined with perioperative chemotherapy with the potential addition of targeted or immunotherapy. The treatment recommendations for SRC remain controversial, especially because of its postulated chemotherapy resistance.
Extracellular ATP and adenosine metabolism seem to modulate T cell activity in the tumor microenvironment. ATP release is triggered in hypoxic or necrotic tumor tissues and can promote antitumoral immunity. Ectonucleotidases such as CD39 (ENTPD1) or ENTPD3 mediate its hydrolyzation to adenosine which down-regulates immune cell activity by supporting T cell exhaustion and tumoral immune escape. A high expression of CD39 seems to be associated with a worse prognosis in GAC and other solid tumors. There are no published studies on purinergic signaling in SRC.
This study aimed to analyze the expression of ENTPD3 and CD39 on different T cell subtypes in patients with GAC/AEG carcinomas in their peripheral blood and peritoneal fluid. We wanted to analyze whether SRC patients show a different purinergic tumor microenvironment than non-SRC patients to understand the mechanism of chemotherapy resistance and find further potential targets for medical treatment options in SRC than chemotherapy alone.

**Material and methods:**
Between 2020 and 2023, 185 patients were undergoing surgery for GAC or GEJ carcinomas. The study cohort included 117 men (63.2%) and 68 women (36.8%) with a mean age of 62 years. Of these, 62 patients met the inclusion criteria, while 31 were SRC and 31 non-SRC tumors. Most patients had received neoadjuvant chemotherapy, mostly FLOT/FLO.
Peripheral blood samples were collected preoperatively, and peritoneal lavage was performed intraoperatively using sterile saline. Peripheral blood mononuclear cells (PBMC) and cells isolated from peritoneal fluid (PF) were stained and analyzed by flow cytometry. The expression of CD39 and ENTPD3 was analyzed on the surface of T cell subtypes. Statistical analyses included Mann–Whitney U test, with $p \le 0.05$ considered significant.

**Results:**
Peripheral blood analysis showed significantly lower ENTPD3 expression on circulating $CD3^+$ ($p<0,001$) and $CD4^+$ T cells ($p=0.020$) in SRC patients compared to non-SRC patients. Additionally, $CD8^+$ T cells in SRC patients also showed lower ENTPD3 expression, but the difference did not reach statistical significance. In contrast, CD39 expression on peripheral blood T cells did not differ significantly between SRC and non-SRC patients.
In PF, the isolated number of immune cells was low, so that results have to be interpreted with caution. Interestingly, SRC patients without peritoneal carcinomatosis showed higher ENTPD3 expression on T cells in SRC compared to non-SRC patients. These inverse findings compared to the blood samples suggest compartment-specific purinergic pathway regulation.
Patients with a higher lymph node status (>N3) showed a higher CD39 expression on CD8+ T cells, independent of SRC differentiation.

**Conclusion:**
Our data show systemic (peripheral blood) and local (peritoneal) purinergic immune cell profiles in SRC and non-SRC patients. Reduced ENTPD3 expression on peripheral blood T cells may represent systemic immune exhaustion in SRC patients, supporting tumor immune escape and progression. In contrast, the non-significant but higher ENTPD3 expression in the PF of SRC patients may indicate local compensatory response in the tumor microenvironment. Nevertheless, further analyses of a representative number of peritoneal cells and tumor surrounding immune cells are needed to elucidate whether patients with SRC may benefit from therapeutic strategies targeting purinergic signaling pathways. CD39 inhibitors, ENTPD3 modulators or adenosine receptor antagonists could represent additional therapies as some studies already postulate.
This study shows differences in the purinergic signaling pathways of the tumor microenvironment of SRC and non-SRC-differentiated GAC/GEJ carcinomas. The results may hint at potential benefits from differential (immune)therapy depending on SRC differentiation. Further studies are needed to understand the mechanisms and test modulators of purinergic signaling.

Abstracts – DCK 2026 – 143rd Congress of the German Society of Surgery – Leipzig, April 22–24 • DOI 10.1515/iss-2026-2002
Innov Surg Sci 2026; 11 (Special Suppl 1): s77–s269

DE GRUYTER BRILL

**5-FU-based treatment (MMT) in rectal cancer (stage >I): Did we have unrecognized DPYD-mutations?**
ID: E-DCK-325

**Viszeralchirurgie / Visceral Surgery**
**Viszeralonkologie, personalisierte Therapie (ACO) / Visceral Oncology and Personalised Treatment (ACO)**

Lara Neidhart[1], Yasmin Haskan[1], Sascha Dierks[1], Paula Liersch[1 2], Johannes Martens[1], Julie Schanz[1], Andreas Leha[1], Torsten Liersch[1]

[1]Universitätsmedizin Göttingen, Göttingen, Deutschland, [2]Universitätsmedizin, Frankfurt am Main, Deutschland

**Background:**
The prospective multicenter TransValid-A trial (ClinTrials.gov: NCT03034473; funded by the DFG; GZ: LI 1727/3-1, 05/2011 to 01/2015) assessed the efficacy of 5-FU-based preoperative chemoradiotherapy (preCRT), quality-controlled total mesorectal excision (qTME) and adjuvant chemotherapy (adCTx; FOLFOX or 5-FU) in locally advanced rectal cancer (LARC, stages ≥ II; < 12 cm above the anal verge). Due to the recommendations of the European Medicines Agency (EMA, in April 2020) post-hoc *DPYD*-testing was performed to evaluate the impact of unrecognized *DPYD*-mutations on 5-FU-associated adverse events (AEs) and survival.

**Material and methods:**
Cryopreserved blood samples of 108 pts with LARC [median age: 62 yrs, f: 38,0%, m: 62,0%, stages II / ≥ III: 17,4% / 82,6% in the lower (42,2%) or mid (57,8%) rectum], who had started preCRT, were tested for DPD-mutations, *DPYD**2A (c.1905+1G>A; IVS14+1G>A; rs3918290), *DPYD**13 (c.1679T>G; rs55886062), the polymorphism c.2846A>T (rs67376798) and Haplotype_B3 (c.1236G>A; c.1129-5923C>G). Multimodal treatment (MMT) consisted of preCRT (RT: 50,4 Gy, 28x1,8 Gy; 5-FU: 1.0 g/m²/d, d1-d5 civ and d29-d33) followed by qTME and adCTx [5-FU: 0.5 g/m²/d, d1-d5, repeat on d29; or FOLFOX (FA: 0.4 g/m² 2h iv; OX: 0.1 g/m² 2h iv; 5-FU: 2.4 g/m² 46h civ, repeat on d15). AEs were reported according to NCI-CTC-AE criteria (version 3.0). Survival was calculated using the Kaplan-Meier estimator, logrank-tests as well as Cox proportional-hazards regression models. *DPYD*-testing was performed according to well-established methods blinded to clinical data.

**Results:**

*DPYD*-testing revealed 100% WT for *DPYD**2A, *DPYD**13 and the polymorphism c.2846A>T. In 9 (8,3%) pts [ypUICC stages 0 (3x), I (1x), IIa (1x) IIIb (3x) and IVa (1x), respectively], a *DPYD*-mutation was detected (8x Haplotype B3, 1x D/V). In three cases RT was reduced to 2%, 3.6% and 10.7%, respectively, according to CTC-AE grades 1 to 3. 5-FU was applied completely in 6 mutational pts and had to be reduced to 10% and 22% of the aimed dose in only 2 pts. AdCTx was omitted in 3 *DPYD*-mutational pts (2x stage 0, 1x stage I), while 5-FU could be administered to 2 pts (1x stage 0 with 5% dose reduction; 1x stage II) and FOLFOX to 4 pts (3x stages III, 1x stage IV), respectively. Long-term follow-up (Median: 69,6 months after qTME) showed that *DPYD*-mutational pts had (in trend) a lower OS

[mean: 51.15 ± 8,1 months (95%-CI: 35,3; 67,0)] vs *DPYD*-WT pts [mean OS: 62.61 ± 2.2 months (95%-CI: 58,32; 66,89); p=0,132].

**Conclusion:**
*DPYD*-testing in LARC-pts revealed unrecognized Haplotype_B3 and D/V mutations in 8.3%. Its impact on MMT-efficacy and survival remains unclear, but in clinical routine investigators should be well aware about *DPYD*-polymorphisms. Further results will be presented in detail at DCK 2026.

DE GRUYTER BRILL

**Underutilization of CRS, HIPEC & PIPAC in Peritoneal Mesothelioma Across a German Nationwide Cohort**
ID: E-DCK-345

**Viszeralchirurgie / Visceral Surgery**
**Viszeralonkologie, personalisierte Therapie (ACO) / Visceral Oncology and Personalised Treatment (ACO)**

Veronika Müller[1], Matthias Hornung[1], Jens Werner[1], Hans Schlitt[1], Miklos Acs[1]

[1]Universitätsklinikum Regensburg, Klinik und Poliklinik für Chirurgie, Regensburg, Deutschland

**Background:**
Peritoneal mesothelioma poses a unique challenge in cancer treatment. Though rare, it is the most common cause of malignant peritoneal carcinomatosis (1). For this disease cytoreductive surgery (CRS) in combination with hyperthermic intraperitoneal chemotherapy (HIPEC) has been identified as the most effective and therefore designated treatment option (1,2). Patients that do not qualify for CRS can benefit from the laparoscopic installation of pressurized intraperitoneal aerosol chemotherapy, PIPAC (1,3), as well as palliative chemotherapy (1).
This retrospective cohort study aims to provide detailed information about the epidemiology and incidence of peritoneal mesothelioma in Germany over the years 2019 to 2024 and to give an overview of current treatment practices.

**Material and methods:**
Data collected by the German Institute for the Hospital Remuneration System, InEK, was analyzed by age, sex, treatment with HIPEC, PIPAC or peritonectomy, ICU stay, type of hospital, and length of hospital stay. Additionally, the Patient Clinical Complexity Level, PCCL, was utilized to categorize arising complications. Data was selected based on its categorization as "peritoneal mesothelioma" by the International Statistical Classification of Diseases and Related Health Problems 10. The treatment options were identified by the assigned operation and procedure codes.

**Results:**
In total 3394 cases were recorded during the years 2019 to 2024 in Germany, with an average annual incidence of 0.68 per 100,000 inhabitants. The mean length of hospital stay was 8.8 days, however with a pooled standard deviation of 11.12 there was a large variation recorded within this data. On average 57.77% of patients were male. The median PCCL was 1 with most categorized as PCCL 0 (31.86% - 42.83%). The median age was 65 to 74, the same as the peak incidence (22.87% - 35.08%). 15.88% of patients had an ICU stay. Most patients were treated at maximum care hospitals with over 1000 beds (26.52 - 35.56%). Only 9.37% of patients received HIPEC treatment. 15.03% received some form of peritonectomy (this included total and partial). In the analyzed time PIPAC was only performed on 4.60% of patients. Accounting for coding two procedures in one patient, just 20.36% of all patients received surgical therapy with just 15.76% being subjected to CRS and HIPEC.

**Conclusion:**

This study depicts the incidence and epidemiological data of peritoneal mesothelioma in Germany between the years 2019 and 2024. It was demonstrated that despite HIPEC and CRS being classified as the best treatment option (1,2) only a small percentage of patients received HIPEC or peritonectomy. The same phenomenon is also identified for PIPAC, a method that has shown great benefits for patients deemed as irresectable (3). The reasons for this could be a lack of consideration of these procedures, however more research is needed on how to mitigate this.

References

1. Rau B, Köhler F, Bachleitner-Hoffmann T, Hacker U, Lehmann K, Piso P, et al. Peritoneales Mesotheliom und Pseudomyxoma peritonei. 2025 Mar; Available from: https://www.onkopedia.com/de/onkopedia/guidelines/peritoneales-mesotheliom-und-pseudomyxoma-peritonei/@@guideline/html/index.html
2. Cashin PH, Jansson Palmer G, Asplund D, Graf W, Syk I. Peritoneal mesothelioma in Sweden: A population-based study. Cancer Medicine. 2019 Oct 1;8(14):6468–75.
3. Cortés-Guiral D, Kranenburg O, Sgarbura O, Van Der Speeten K, Taibi A, Hübner M, et al. PIPAC Pharmacologic and Clinical Data. Journal of Surgical Oncology. 2024 Nov 1;130(6):1337–48.

DE GRUYTER BRILL

### Functional characterization of mutation-associated neoantigens arising from nonstop mutations in EGA

ID: E-DCK-397

**Viszeralchirurgie / Visceral Surgery**
**Viszeralonkologie, personalisierte Therapie (ACO) / Visceral Oncology and Personalised Treatment (ACO)**

Maria Garcia-Marquez[1 2], Loreen Sophie Rudek[1 2], Martin Thelen[1 2], Eugen Bauer[3], Kerstin Wennhold[1 2], Ali Yazbeck[1 4], Christiane Bruns[2], Birgit Gathof[3], Axel Hillmer[4], Hans Schlößer[1 2]

[1]Center for Molecular Medicine Cologne, University of Cologne, Faculty of Medicine and University Hospital Cologne, Cologne, Deutschland, [2]Department of General, Visceral, Thoracic, and Transplantation Surgery, University of Cologne, Faculty of Medicine and University Hospital Cologne, Cologne, Deutschland, [3]Institute of Transfusion Medicine, University of Cologne, Faculty of Medicine and University Hospital Cologne, Cologne, Deutschland, [4]Institute of Pathology, University of Cologne, Faculty of Medicine and University Hospital Cologne, Cologne, Deutschland

### Background:

Nonstop mutations are nonsense mutations that replace a stop codon by a sense codon leading to a continued translation into the 3’ untranslated region (3’ UTR) of a gene. Due to disruption of normal translation termination, an abnormally elongated protein is generated. Nonstop transcripts are often eliminated by the nonstop decay (NSD) pathway. But if the elongated protein is folded, it can be misfolded, destabilized, or dysfunctional. Novel peptide sequences derived from nonstop mutations may result in neoantigens. Nonstop mutations have been identified in various cancer types, including colorectal, lung, and breast cancers.

### Material and methods:

Tumor mutational burden and nonstop mutations were detected by whole exome sequencing (WES) in a cohort of patients with esophago-gastric adenocarcinoma (EGA). These nonstop mutations were used for neoantigen prediction. Transcripts were generated beyond the affected stop codon up to the next stop codon or the end of the transcript (extension). The patient’s human leukocyte antigen (HLA)-genotypes were determined and in combination with the extended transcript sequences, HLA-binding peptides were identified using the “Immune Epitope Database” (IEDB). The predicted peptides were synthesized, and they were used to stimulate patient-derived lymphocytes in vitro, aiming to determine whether nonstop extensions are immunogenetic and represent promising targets for tumor-specific T-cell responses. FluoroSpot was selected as a functional assay to assess immunogenicity of predicted neoantigens in vitro.

### Results:

We performed WES of 72 treatment-naïve EGA patients and identified eleven patients with nonstop mutations. The frequency of nonstop mutations in our cohort (15.3%) was consistent with findings based on “The Cancer Genome Atlas” (TCGA) database (19.0%). Analysis of the tumor mutational burden in our cohort revealed that nonstop mutations are more prevalent in highly mutated tumors. Microsatellite instability was overserved in five out of eleven (45,5%) patients with nonstop mutations. To check if the nonstop-mutation-derived neoantigens induce immune responses, we selected four patients, determined the nucleotide sequence of the transcripts affected by the nonstop mutations, and synthesized the predicted nonstop-mutation-derived peptides. We did not detect endogenous interferon-gamma (IFN-γ) responses upon stimulation of peripheral blood mononuclear cells (PBMCs) with nonstop peptides in FluoroSpot assays. In one patient, isolated tumor-infiltrating T cells co-cultured with antigen-presenting cells (APCs) and nonstop peptides exhibited enhanced IFN-γ secretion, indicating a specific immune response. Furthermore, PBMC-derived T cells expanded with low-dose IL-2, IL-7, and IL-15 in the presence of APCs and nonstop peptides revealed antigen-specific expansion of $41BB^{+}CD69^{+}$ T cells, further supporting neoantigen-specific T-cell activation.

**Conclusion:**
Taken together, while endogenous responses to nonstop peptides are not widely detectable in the peripheral blood of EGA patients, antigen-specific T-cell responses can be elicited and expanded ex vivo. This suggests that nonstop mutations may represent immunogenic neoantigens in certain EGA patients with implications for personalized immunotherapy.
Due to the threshold of functional T-cell assays for assessing mutation-associated neoantigen-specific immune responses, we are performing T-cell receptor (TCR) sequencing of T cells expanded with nonstop peptides to gain a deeper insight into the immunogenic potential of nonstop mutations.

DE GRUYTER BRILL

### Prognostic Factors in Pseudomyxoma Peritonei Patients Undergoing Cytoreductive Surgery and HIPEC

ID: E-DCK-427

**Viszeralchirurgie / Visceral Surgery**
**Viszeralonkologie, personalisierte Therapie (ACO) / Visceral Oncology and Personalised Treatment (ACO)**

Jan Philipp Ramspott[1], Anna Kolmhofer[2], Lukas Schabl[3], Philip Schredl[3], Klaus Emmanuel[3], Jaroslav Presl[3], Dino Bekric[4], Andreas Pascher[1], Daniel Neureiter[4], Tarkan Jäger[3]

[1]University Hospital Muenster, Department of General, Visceral and Transplant Surgery, Muenster, Deutschland, [2]Salzburg Cancer Research Institute – Laboratory of Immunological and Molecular Cancer Research, Department of Internal Medicine III, Salzburg, Österreich, [3]Paracelsus Medical University Salzburg, Department of Surgery, Salzburg, Österreich, [4]Paracelsus Medical University Salzburg, Institute of Pathology, Salzburg, Österreich

**Background:**
Cytoreductive surgery (CRS) combined with hyperthermic intraperitoneal chemotherapy (HIPEC) has been shown to significantly enhance the long-term prognosis for patients with pseudomyxoma peritonei. However, approximately 20% of these patients experience tumor recurrence and progression and may not derive benefit from repeated CRS-HIPEC procedures, thereby necessitating meticulous patient selection. This meta-analysis examines the potency of perioperative predictive markers as indicators for appropriate patient selection, aiming to identify those most likely to benefit from CRS-HIPEC.

**Material and methods:**
A systematic literature review and meta-analysis were conducted in accordance with the Preferred Reporting Items for Systematic Reviews and Meta-Analyses (PRISMA) guidelines. The inclusion criteria required histologically confirmed pseudomyxoma peritonei and studies assessing the prognostic significance of clinical-pathological, (immuno-) histological, and molecular parameters on the efficacy of CRS-HIPEC, as measured by overall, disease-free, and progression-free survival. Statistical analysis was performed using R (Version 4.4.3).

**Results:**
Sixteen studies involving 4,009 PMP patients were included in the analysis. Age, sex (male), completeness of cytoreduction score, peritoneal cancer index, tumor markers (CEA, CA 19-9, CA-125), tumor grade (high), histological subtype, and Ki-67 were significantly associated with prognosis prediction.

**Conclusion:**
Clinical-pathological and (immuno-) histological parameters were significantly associated with prognosis in pseudomyxoma peritonei patients undergoing CRS and HIPEC. Data on molecular prognostic factors remain limited. Given the evolving landscape of oncological research, further studies incorporating molecular biomarkers are critically needed to refine patient selection criteria and optimize outcomes for CRS and HIPEC.

DE GRUYTER BRILL

**Comprehensive Nationwide Analysis of Outpatient Care for Peritoneal Metastasis in Germany**
ID: E-DCK-8

**Viszeralchirurgie / Visceral Surgery**
**Viszeralonkologie, personalisierte Therapie (ACO) / Visceral Oncology and Personalised Treatment (ACO)**

Derya Danisman[1], Veria Khosrawipour[2], Shiri Li[3], Carina Jaekel[4], Wolfram Trudo Knoefel[1], Tanja Khosrawipour[1]

[1]Universitätsklinikum Düsseldorf, Chirurgische Klinik, Düsseldorf, Deutschland, [2]University of Environmental and Life Sciences Wroclaw, Department of Surgery, Wroclaw, Polen, [3]Presbytarian hospital New York Weill Cornell University, Department of Surgery, New York, Vereinigte Staaten, [4]Universitätsklinikum Düsseldorf, Klinik für Orthopädie und Unfallchirurgie, Düsseldorf, Deutschland

**Background:**
Peritoneal metastasis (PM) is a complex clinical condition associated with high morbidity and requiring interdisciplinary management, even in inpatient settings. However, data on the outpatient management of PM are scarce. This study provides, for the first time, a nationwide analysis of outpatient care for patients with PM in Germany.

**Material and methods:**
Claims data were obtained from the medical service authorities of the Association of Statutory Health Insurance, covering all patients who received outpatient treatment specifically for PM between 2014 and 2021, including the COVID-19 pandemic period. Analyses focused on regional incidence, prevalence, and age and sex distribution.

**Results:**
A total of 69.6% of patients were managed primarily by general practitioners, 22.8% by oncologists/hematologists, and 8.2% by gynecologists. Between 2014 and 2021, the nationwide incidence of PM increased by 5.3%, reaching 19,359 cases. Significantly higher regional incidence rates were observed in the eastern German states (formerly the German Democratic Republic). A wide range of diagnostic and therapeutic procedures were ordered and performed in the outpatient setting.

**Conclusion:**
Outpatient management of patients with PM is complex and involves extensive diagnostic and therapeutic interventions, the majority of which are coordinated by primary care providers. It remains unclear whether the expertise and resources available in primary care are sufficient to meet the diverse needs of these patients throughout the course of their progressive disease. Further research is needed to better understand and optimize outpatient management strategies for PM.
